# Supplementary material for: Understanding factors influencing utilization of HIV prevention and treatment services among patients and providers in a heterogeneous setting: A qualitative study from South Africa
Source: PLOS Glob Public Health. 2022 Feb 3;2(2):e0000132. doi: 10.1371/journal.pgph.0000132 (PMC10021737; doi:10.1371/journal.pgph.0000132)
Supplement: S1 Data — (ZIP) [file pgph.0000132.s001.zip › Supplementary information/IDI_Clinic attendee_QA001.pdf]

Full Participant ID: QA001
Participant Type: Female
Location: Clinic A
Date: 16 July 2020
Start time: 10:49
Primary interview language: English

Label Key
I = Interviewer
P = Participant
N = Notetaker

I: Do you agree to be audio recorded?

P: Yes

I: Thank you. We are going to start like this, as we explained that we will be conducting research and you signed the informed consent and you also signed the audio consent form. I just want to remind you that the information that we share here is confidential and we are going to record this because I can't capture everything in writing so audio will also help us to capture things that we miss during the interview.

P: Okay

I: Okay, can you tell me more about yourself?

P: My name is (xxx name of the person) I stay in XXX (Name of Area), I have two kids. I live with my sister; I live with my sister in XXX (Name of Area). Err I'm 32 years old, yah.

I: Alright, are you married?

P: No. I'm not married

I: Okay. So, can you please tell me how long you have lived in that area?

P: Err is not because I'm from XXX (Name of Area) so I came to XXX (Name of Area) to stay there so that I can be err near the workplace. Now I'm staying in XXX (Name of Area), I work in, I (laughs), I live in, I work in XXX (Name of Area)

I: Alright

P: Yes

I: So, you work around here?

P: Yes, I work around XXX (Name of Area)

I: Okay, Err I heard you talking about XXX (Name of Area), so grew from there or?

P: Yes, I grew up in XXX (Name of Area).

I: Okay, that's where you can say you have been staying there?

P: Yes

I: Okay and you just said you haven't been staying in XXX (Name of Area) for long.

P: Yes

I: It is for how long maybe, if you know or if you can estimate?

P: mmm 1 year – 2 years

I: 1 year – 2 years you've been staying in XXX (Name of Area)

P: In XXX (Name of Area), yes.

I: So, you attend this clinic because you work around here?

P: Yes.

I: Alright. Can you tell me how long have you been visiting this clinic?

P: It has been many times, yah it's been, from last year, yah from last year when I started working this side.

I: Okay, can you also please speak up so that we can capture you on the audio

P: Okay

I: Okay, you said you have been visiting this clinic from last year

P: Mmhh (yes)

I: So, have you ever visited any other clinic in the area?

P: No, I didn't, this is my first one

I: Okay this is your first clinic?

P: Yes

I: Can you tell me, what do you like about this clinic?

P: The service here is very, very good. The nurses here know how to speak with people, they accommodate you with everything, they don't shout, they don't, they ask you nicely or if you are ill, what do you want and so on.

I: Okay, so have you ever, since you said you haven't attended any other clinic so do you think in other clinics, they don't treat patients well or?

P: Yah, I've been to other clinics like I said I come from XXX (Name of Area). There's a different between XXX (Name of Area) and here.

I: Okay,

P: Mmhh (yes)

I: What's the difference?

P: In XXX (Name of Area) maybe it's because is rural I don't know. The service is too slow, we wait for too long, even if you came sick you can even get well while you are on the queue

I: So, you come sick

P: Yah (yes),

I: And you get well?

P: You come sick, you can even get well on the queue there and decide to leave because you stayed too long.

I: Okay, what are the dislikes in this clinic, I mean what do you dislike in this clinic?

P: The only problem I have in is clinic, is too small, that is the only problem I have, it is too small like it is crowded.

I: Okay, could you tell me whether you are HIV infected or not?

P: I am, I'm drinking medication. I am on treatment.

I: Okay, you said you are on treatment

P: Mmm (yes)

I: How long have you been on treatment?

P: From last year February

I: Okay, Last year February

P: Mmm (yes)

I: Have you ever defaulted?

P: Never

I: Okay, so, tell me here, can you please tell me what you think are the major factors affecting your health at the moment?

P: Since I've been in this treatment, I don't see anything wrong with it like I'm normal. It doesn't do anything to my body or otherwise, I'm fine. It doesn't affect me in anyway.

I: So, in other words, there's no any other major issues that are affecting you other than the factors that would be caused by the treatment?

P: No, ha-a, I'm fine with it. The only thing I can see, I'm getting big like I'm gaining weight

I: So, do you think you can reach the obesity level?

P: I think so, but I will make a plan (laughs)

I: Okay, do think these factors are also affecting other people that you know?

P: Err not that I know of, I don't know.

I: Okay, you don't anyone who has been affected

P: ha-a (no), they cant tell you, you know people with their statuses they cant even tell you, even if you say me I'm on medication I'm HIV what, what, you can only speak for yourself. Other people, they are too private with their status.

I: Alright

P: Mmm (yes)

I: So, you don’t know

P: I don’t know

I: Alright, lets move forward, in your experience, err when it comes to the services, I mean the delivery of the health services here in this facility, can you tell me your experience there?

P: I don’t have any complains about the service. The service is, for me is good, I can’t complain about it.

I: Okay, can you describe the services that they offer you that you experienced? It can be good; it can be bad.

P: No, it’s good. For my side is good

I: I mean can you tell me your experience like when you come here to get the services how do they deliver it to you, how do they do things in your experience?

P: In my experience, when I enter the premises we wait in the queue err social distance and the, they even come and explain err the chronic stand this side, and the sick people, and the child and then when we enter the door they screen us and with a smile like they talk with us in a polite way and then we, they screen us and they register us, we sit down on the chair err we wait for your time to come to the vitals if you are coming like everything is fine for me

I: Okay do you always get what you want when you come here?

P: Always, I don’t have any complains

I: Okay

P: Mmm (yes)

I: So, you have never come here and leave without

P: Without my medication or something that is short, no.

I: Okay, have you ever seen any people being turned back because they… for other services?

P: They can turn them back if maybe their medication is not there or maybe the clinic has some emergency that’s how I see it.

I: And how often does that happen?

P: Ahh, not always when I’m here, I don’t see it often when I’m here. Just early in that days or that month

I: Okay

P: Mmm (yes)

I: You’ve been saying that err the service here is good, you like it, can you tell me what are the good things, I mean positive features in this facility or the facilities that you visited?

P: Mmm, there’s, the nurses here, the SR’s here they, like they, even if you don’t understand they make you to understand, even if you talk some other languages that maybe you can’t hear but for me I see them, they can accommodate you with your language they try by all means to accommodate you.

I: Okay, and then the most challenging issues?

P: The most challenging

I: in the facilities that you have visited?

P: Err like my, my own, the place I come from err XXX (Name of Area) I'll talk about XXX (Name of Area) my first, it is because we used to one language neh

I: Mm-hh (yes)

P: when someone comes like talking other language is very difficult because we are used to like Sotho, so if someone comes with Zulu or Venda it is very hard to accommodate that person (giggles)

I: Okay, and then here?

P: Here *hai* (no) they all, I think around here they all, they all know the other languages

I: Okay

P: Yes

I: Alright, I'd like you to tell me about your experience when it comes to getting HIV care here

P: In my own experience, err, ahh everything is, like when is my date I come and even if it's my time to take the blood I do or maybe it's my time only to take the medication I get my medication then everything is fine for me

I: So, you don't see any problem, you don't encounter any problem?

P: No,

I: Alright, so when you come here you don't stand long queues, you don't do anything, you don't wait?

P: Huh-uh (no) I've never stand like 2 hours maybe on the queue outside waiting for them to come and register us, no

I: Okay

P: Mmm (yes)

I: So, it's in and out?

P: It's in and because *vele* (for sure) chronic we can't wait for that long

I: Do they turn people because the blood has been collected already, is after hours?

P: No, I've never seen anyone being turned back because of something is not there maybe err because of maybe that medication is not there or something but, in my experience,, I've never seen turned back because of the blood or what

I: Okay,

P: Mmm (yes)

I: Okay, what are the things in this facility or this clinic that you think or that you would like them to improve or that you think should be improved?

P: Eish, I don't know if they can improve err ahh like maybe they can extend the clinic. That's the only problem because it's too small, about the service huh-uh (no). (noise on the

background) Yah I was talking about the service; the service is good. The only problem I have is the space because we can't be too many in a small space

I: Okay, let's go or let's move forward. And now we are going to talk about HIV prevention. So, in your own understanding, in your own understanding not in anyone else's understanding, what do you understand about HIV prevention?

P: You mean the medication?

I: Prevention

P: Oh, HIV it's a…

I: HIV prevention, I mean what do you understand there about HIV prevention?

P: It's a, it's a method that err make one another not get infected

I: Alright, can you tell me different types of HIV prevention services that you know?

P: Err, using condoms, and that's it

I: So, you only know using condoms?

P: yah, using condoms

I: Okay, mmm what do you think are the some of difficulties you may experience accessing HIV prevention services?

P: Err, I don't think there noth… there's nothing difficult to get the access of the HIV prevention because they are free, you can go to the clinic and get some if you cant afford to buy some.

I: Alright

P: mmm (yes)

I: I'd like to know that err let me ask you this. Do you use condom?

P: Yah I do, yes, I do.

I: okay can you tell me why do you use them?

P: mmm so that I cannot infect other people

I: Okay

P: mmm (yes)

I: That's all?

P: And to protect myself

I: Okay, how often do you use them?

P: Since I've been in treatment, I use them every day (giggles) every time when I'm doing the deeds (laughs)

I: Okay

P: Yes

I: Can you tell me where you get the condoms?

P: In the clinic

I: All the time you get them from the clinic?

P: yah because they are free. I cannot buy something that I know is for free

I: Okay, what are other places that you can get condoms? I mean the places where you can get condoms.

P: At the shops you can buy

I: mm-hh (okay)

P: yah at the shops

I: Okay, what do you think would prevent you from using condoms?

P: (Silence for 20 seconds) I don’t think there’s nothing that can, it can, I don’t think there's nothing that can (knocking at the door)

I: Okay somebody is knocking at the door lets attend (paused the recording). Sorry for the interruption lets continue. Should I repeat the question?

P: Yes

I: I mean if you were to find yourself not using condom what do you think would prevent you to use the condom?

P: If I plan to have a baby maybe I can stop using a condom

I: Okay, is that all?

P: Mmm (yes)

I: Can you please explain what the universal test and treat is?

P: is when they find, if they test you, they find you positive (background noise – interruption – paused)

I: Okay before the interruption you were still explaining what universal test and treat is.

P: Oh yes, the universal test and treat is when they, when they test you and then they find you positive you start the treatment as soon as possible. Not like before when they have to wait for the CD4 counts and what so on

I: So, what are the some, what are the some of advantages of universal test and treat?

P: What are the what?

I: Advantages of UTT which is universal test and treat. Okay what’s good about universal test and treat

P: The, the, I think the thing about, the good thing about this is that like me when I find out that I was HIV I was pregnant so in a way it helped my baby yah it helped my baby and myself so that I can know and live healthy.

I: Okay, and what are the disadvantages of the UTT?

P: Sometimes you are going to drink the medication without knowing your CD4 count and then only to find out your CD4 count is fine to start the treatment already, so sometimes, hai

(no) I think, I think somehow is bad in a way because you cant drink the treatment not knowing your CD4 count

I: Okay

P: So, yah

I: Alright, has there been any changes to the way health information or health service been delivered since the immediate ART began that has changed the way you look after your own health?

P: No, I've been living a normal way like before I've never changed any diet of how I look at myself. I'm still the same way

I: Alright

P: Something changed again about using condoms every time when I'm doing the deeds

I: Okay

P: And yah, and my body wise because I think is the medication or so, I don't know

I: So, do you do something about the body changing or you just letting it be?

P: I just let it be

I: Alright, okay what are the issues have you experienced that prevented you from accessing or taking ARV's?

P: The issues?

I: Mmm (yes)

P: No, I don't have any issues

I: Okay what do you think would happen if one continues taking ART?

P: Is going to happen?

I: Mmm (yes) if you keep on taking ART

P: You keep on taking them?

I: Mmm (yes) you keep on taking your medication what do you think will happen?

P: Ahh, I don't know

I: So, they didn't tell you what happens when you take the medication

P: Huh-uh (no) they didn't. some other things they don't explain we just have to think. I don't know I don't want to lie; I don't know.

I: So, the just told you that take the medication

P: Yah, since I've started ahh they have just said take the medication they don't tell us *ore* (that) if you stop or if you don't stop this is going to happen, I don't know

I: So, you are trying to tell me that you don't even know what will happen if you stop?

P: If I stop, I can only think neh?

I: Okay, what do you think?

P: If I stop, it means I’m going to die

I: Okay, that’s all you thinking?

P: Mmm (yes)

I: So, they just tell you that you are HIV positive, take your medication

P: Mmm (yes)

I: Nothing else?

P: Huh-uh (no)

I: You don’t know why you are taking the medication?

P: Serious I don’t know. For the viral load and what’s so on, CD4 count (laughs) we must take the medication for the CD4 count and my viral load so that it cannot drop yah, it cannot go down something like that

I: Okay

P: Mmm (yes)

I: I think you should go to your health promoter and then you ask about these things, what happens if you continue taking medication

P: Mmm (yes)

I: What happens if you stop taking medication

P: Okay

I: They will explain properly or when you collect your medication you should ask them

P: Okay

I: Err okay, can you tell me, like since accessing the facilities for HIV prevention err, could you explain hoe your life has been impacted?

P: Mmm it has changed a lot because *nna* (me) before I’m not that condom person but now I have to use it every time when I do the deeds and it affects me because sometimes it makes me dry

I: Okay

P: Yah and I don’t feel the thing that I’m doing like normally (laughs) before, I don’t know if it’s in my head or what, but it affected me badly

I: Alright, okay.so can you explain the HIV prevention services, do you think they have been helpful to you?

P: Do you mean the condoms? Yah they have been because there's like, in fact *ke batla oreng* (what do I want to say) even if it’s for HIV prevention neh there's other err illnesses like STI and what so on so when I use it at least I’m saving myself from other things

I: Alright

P: Mmm

I: So, that how you think it has been helpful to you?

P: Yes

I: You are saying is not helpful to you only for HIV but also for STIs?

P: Yes

I: Alright. It is time for us to close this part of the interview.

P: Okay.

I: But before we do, is there anything else about this topic that we haven’t discuss that you feel it is important to you and you want to say it?

P: Huh-uh (no) I’m fine with everything

I: You can ask any question

P: Huh-uh (no)

I: Alright now we have come to an end of our discussion. Thank you very much for your participation. If you have any question about your study participation please contact us, there are numbers on the ICF that you signed, the Informed Consent Form there are relevant people to contact. Thank you very much.

Time Ended: 11:50
