# Supplementary material for: Understanding factors influencing utilization of HIV prevention and treatment services among patients and providers in a heterogeneous setting: A qualitative study from South Africa
Source: PLOS Glob Public Health. 2022 Feb 3;2(2):e0000132. doi: 10.1371/journal.pgph.0000132 (PMC10021737; doi:10.1371/journal.pgph.0000132)
Supplement: S1 Data — (ZIP) [file pgph.0000132.s001.zip › Supplementary information/IDI_Clinic attendee_QA002.pdf]

1 PARTICIPANT IDENTIFICATION NUMBER: QA002

2 RESEARCH ASSISTANT: XXXXXXX (NAME OF RA)

3 TYPE OF THE PARTICIPANT: CLINIC ATTENDEE

5 LANGUAGE: ENGLISH

7 I. Can you please tell me more about yourself?

8 P. I don’t understand.

9 I. About yourself.

10 P. I am not that person that talk too much my sister, like what do you like to know be specific to the question.

11 I. Maybe how old are you, where are you coming from, are you married do you have any children?

12 P. Okay.

13 I. Yah (yes)

14 P. I am from here XXX (Name of Area), I am not married and I am 40 years old. Mmm (yes) what else?

15 I. I you coming here XXX (Name of Area)?

16 P. Yes.

17 I. Do you have any children?

18 P. Yes I have one.

19 I. Is it a girl or a boy?

20 P. It’s a girl.

21 I. How old?

22 P. 12 years old.

23 I. Okay, can you please tell me how long have you lived in this area?

24 P. This area?

25 I. Yah (yes)

26 P. Since 2013

27 I. How long have you being visiting this clinic?

28 P. It’s a first time.

29 I. It’s the first time?

30 P. Yah (yes)

31 I. Okay, have you visited other clinic in this area?

32 P. No.

33 I. What did you like about this clinic and what did you dislike about this clinic?

34 P. As I have told you that is the first time in this clinic, I haven’t seen anything wrong in this place, probably when times goes on. I will answer the next question.

36 I. Meaning that is the first time.

37 P. The is nothing that I can say.

38 I. Could you tell me whether you are HIV infected?

40 P. Yes I am.

41 I. How long have you being infected with HIV?

42 P. I don’t know because I came today I found that I am HIV positive.

43 I. You are not on ART, or you are not on medication?

44 P. No is the first time I am going to take it.

45 I. Could you tell me what are the major factor affecting your health right now?

46 P. Mmm (yes) *ake* sure (I am not sure) I am not sure about that.

47 I. You mean very thing is fine

48 P. Yes.

49 I. do you think this factor affecting other people?

50 P. Ahhh which one the one I am having.

51 I. Yes.

52 P. Yes I think so

53 I. You don't see anyone affecting with this factors or you are just thinking about it?

54 P. Just thinking about it my sister.

55 I. Okay. Can you tell me your experiences of health delivery from health care facility?

56 P. Mmm (yes) laughing I don't answer that one, I am not used to come to the clinics and all the staff. Actually I am ignorant to things like this*, uya understander* do (you understand me)

57 I. Yah (yes). what are the some of positive features in this facility that you have visited and what are the most challenging features in this facility that you may have.

58 P. Mmm (yes) as have said I don't come to this facility my sister, like now I am going to focus on those things.

59 I. So today you don't have any challenge?

60 P. No I don't have.

61 I.Okay, Can you tell me about your experience about getting HIV care?

62 P. My experience?

64 I. I don't have any experience of this one.

65 P. Because it's your first time?

66 I. It's a first time

67 I. What are the things you would like to improve in health services in your health facility?

68 P. To improve?

69 I. Yes.

70 P. For sure I have to take my medications so that I can see some of the things, Actually I don't want to see some of the things if I don't take my medication, I don't know my answer is clear on you or what?

71 I. No I understand because you are still new.

72 P. Yah (yes).

73 I. what do you understand about HIV prevention?

74 P. Prevention meaning? (Noise at the background) *u e preventa so* (preventing it)

75 I. Yes.

76 P. *Uri kenwa de philise* (To drink my medication)

77 I. Do you use kind of prevention now?

78 P. For sure I have to use it now.

79 I. Can you tell me the different type of HIV services?

80 P. No I don’t nothing about that.

81 I. You don’t know nothing on that?

82 I. What are the some of difficulties you may experience in accessing HIV prevention?

83 P. Eish my sister I don’t know those things.

84 I. You don’t know nothing?

85 P. Sometime is good not to lie about things you don’t know.

86 I. Do you use condoms?

87 P. Sometimes.

88 I. why do you use condom?

89 P. Like you like to know why today I am here, I have cheated my girlfriend you know so that’s the other reason that me to come and test, I only find out that I am positive and she negative.

90 I. How often do you use condom?

91 P. Monthly or weekly?

92 I. What did you do maybe and how often did you use condom, every day when you have sex or?

93 P. Yes I do use condom my sister (Noise at the background)

94 I. Where do you use get the condom from?

95 P. Like what?

96 I. Where did you get them from the condom?

97 P. Where do i get?

98 I. The condom from?

99 P. I don't understand the question.

100 I. Where do you get the condom from? Do you buy them or?

101 P. Yah (yes) I buy it. (Noise at the background)

102 I. What will prevent you from using the condom? And what will prevent you from getting the condoms?

103 P.Okay, I can't answer that my sister.

104 I. can you explain what the universal test and treat is?

105 P. I don't know the universal test.

106 I. You don't know?

107 P. Yah (yes)

108 I. Has the any challenge in health service ever since the immediate ART the way you look after your own health (Noise at the background)

109 P. No the is nothing.

110 I. The is nothing?

111 P. Yes.

112 I. Issues that can prevent you from taking the ARVS? (Noise at the background)

113 P. Come again my sister.

114 I. What if any issues you have experience that may be preventing you or accessing taking the ARVS

115 P. I don't understand such question.

116 I. What can prevent you from taking the treatment?

117 P. What can I say the reason of taking the ARVS is to prevent the HIV from getting up, actually to reduce? Is that right?

118 I. Yes.

119 P. Okay,

120 I. What do you think if one continue to take ART or stop to take their medication for HIV?

121 P. I don't know my sister.

122 I. you don't know?

123 P. I don't know.

124I. Since accessing the facility of HIV prevention services could you explain how your life has being impacted?

125 P. No I don't.

126 I. What happened today when you find out that you are HIV positive? What is your reaction on that?

127 P. Sometime I see on TV when they talk from those shows I didn't feel maybe and I didn't know anything you know, maybe I get shocked as I am listening to the people that talked from those show that HIV is doing one two three you understand. I got shocked but I am strong.

128 I. Do you think you can take this medication from today?

129 P. Yah (yes) I already have them on my pocket I am going to take them today late.

130 I. Do you know any of the side effects of this medication you are having?

131 P. They have told me that they will be some but I must not take it seriously but if they become bad I must come and see the guys here.

132 I. Can you explain the HIV prevention services that you think have being helpful to you?

133 P. (Laughing) eish my sister I can't answer that.

134 I. I mean the nurses that were helping you today when you test neh, they were helpful or not?

135 P. They were helpful.

136 I. How?

137 P. Like they explain everything to me and what I must not do.

138 I. What did they tell you?

139 P. They tell me that as I am going to start the medicine I mustn't stop I must make sure that I drink a lot of water and eat healthy and follow up what they have told me every day, I mustn't forget my time that I am going to take my treatment and must always make sure I am doing the right thing, and make sure that now I must use a condom if I feel like I want to have someone next to me.

140 I. Do you think you are going to follow up what the nurses have told you today?

141 P. Yes I will my sister I will and I will make sure and safe other people's lifes.

142 I. We have come to the end of our section if you have anything to add you can say it, may if you have something to say about the clinic or the nurses you can add before we close our section.

143 P. No I don't have anything to say or anything to ask my sister. I am okay.

144 I. You the service that they have offered you today was perfect?

145 P Yes sure.

146 I. Okay thank you very much for taking time in your study the time is 13:48 thank you so much for your time.

147 P. Okay dankie sister (thank you sister)
