# Supplementary material for: Understanding factors influencing utilization of HIV prevention and treatment services among patients and providers in a heterogeneous setting: A qualitative study from South Africa
Source: PLOS Glob Public Health. 2022 Feb 3;2(2):e0000132. doi: 10.1371/journal.pgph.0000132 (PMC10021737; doi:10.1371/journal.pgph.0000132)
Supplement: S1 Data — (ZIP) [file pgph.0000132.s001.zip › Supplementary information/IDI_Clinic attendee_QA003.pdf]

Full Participant ID: QA003
Participant Type: Female
Location: XXX NAME OF CLINIC
Date: 16 July 2020

Label Key
I = Interviewer
P = Participant
N = Notetaker

I: Do you agree to be audio recorded?
P: Yes, I do
I: Okay, I'd like to remind you that the information you share here is going to be confidential, what you will say will not be connected to you, we are going to use a PID Number, PID is QA003.
P: Okay.
I: Okay let's start here, can you tell me more about yourself?
P: I am 40-year-old lady, born and bred in XXX (Name of Area), I have 12-year-old girl and I work for the department of education.
I: Okay, are you married?
P: I'm engaged.
I: Alright, can you tell me how long you have lived in this area?
P: It's plus or minus 6 years
I: So you.ve been here
P: Come again?
I: You've been here for a very, very long time
P: Yes
I: Since you are in XXX (Name of Area), are you still staying in XXX (Name of Area) or you are staying in XXX (Name of Area) now?
P: Now I reside here
I: and it has been
P: More than 6 years
I: Okay let's pause there's interruption
I: Sorry for the interruption, have you visited other clinics in the area?

P: No, it is the first time

I: For the past 6 years

P: It is the first time

I: Alright, what do you like about this clinic?

P: It’s near my house so I decided that I want to come here

I: And something that you don’t like about it?

P: I haven’t experienced anything as of

I: Okay, do mind telling me if you are HIV infected or not?

P: Is it not confidential? Is not supposed to be confidential?

I: It can be, that is why I am asking do you mind

P: No, I don’t mind if it will be

I: It won’t come back to you

P: No, yoh your question, that question, I’m not

I: Okay what about that question

P: It is a confidential question; you don’t ask a person that question

I: But we said whatever we are going to discuss is going to remain confidential

P: But ahh I think you are answered, I am not

I: Okay, can you tell me what are the major factors affecting your health right now?

P: My health now?

I: Yes

P: I take precautionary measures of my health; I try to do everything that. Are you talking about HIV part of the Corona virus?

I: Everything that might be affecting, issues that might be affecting your health, anything

P: I do take precautionary measures

I: Not limited to anything?

P: On every part when it comes to HIV I try as much as I can to use protection, I don’t know if I have answered the question, like I am health conscious if I can say that?

I: So, there are no factors affecting your health?

P: No, not that I know of

I: Okay, so are they any factors that you know and might affect other’s health?

P: I can’t say that I don’t know. It depends on the person or the immune system of that person, I don’t have an answer for that one

I: Okay, can you tell me, like in terms of service delivery in this facility or from healthcare facilities, can you tell me your experiences there?

P: Like I’m saying I’ve never been to a clinic this is my first time but so far I haven’t seen anything that I can complain about but from what I’ve heard from other people err people are treated bad at public clinics but I haven’t experienced or been in that situation

I: Okay, can you tell me what some of positive features that you have experienced today since it was your first time?

P: Err, I got the help that, I got what I came here for and I think I came here around about 11H00 and I’m done, and some people they came here at, early in the morning so *nna* (me) I’m satisfied so far

I: Okay in other words you can’t tell me any of other challenges that you experienced?

P: The challenge, okay for now what I can say the challenge is *akere* (because) we know there’s this epidemic about the corona whatever, whatever

I: Yes

P: I don’t see social distancing happening around. I’m not sure maybe I’m too conscious but I did not see social distancing happening because we are seated on the same bench with other people which I don’t know what they are here for, so that is the only

I: The only thing?

P: Yes

I: Okay I wanted to ask something, since you were here from 11 what are the things you would to improve about the health services in this facility?

P: Nothing (noise on the background) nothing

I: Just like that “nothing” (noise on the background) alright, I heard you talking about being conscious on your health, can you tell me, what do you understand about HIV prevention?

P: HIV prevention?

I: Mmmhhh (yes)

P: It’s knowing your partner’s status that the first prevention and using protection at all times even if it is difficult. What else? If you are positive you take your medication that is being cautious

I: Alright, can you tell me different types of HIV prevention?

P: You abstain, condomise, you become faithful to your partner

I: Those are the ones you know?

P: Only ones I know

I: Okay, tell me, what some of the difficulties that you may experience in accessing HIV prevention?

P: Like in terms of in terms of going to the clinic?

I: anything like when I want to get my HIV prevention services that might stand on my way

P: I think they should have their separate room for people with HIV. They must not be, what do I want to say? I think there must be a separate room *ya ore okay this a room ya batho ba eleng for medication ya* (for people who went for HIV medication) HIV or about HIV related

issues, they must be separate so that people can have their privacy if *ba kopakopana* (they mix) with other, like you to this room you go to that room, you go to like everybody *wa bona ore this o tlesetse ke motho wa* (can see you came to do what) what's your company? Ya Aurum or whatever

I: Aurum

P: Automatically they assume that this one it means o positive so they should have privacy

I: Oh okay, so the stigma can

P: Yes, yes

I: That's what you are trying to say

P: Yes

I: Okay, can I ask you something like do you use condoms?

P: Sometimes

I: Why do you use them?

P: (Laughs) I said sometimes so it means I only use it depending on the situation

I: Okay, what might be the situation if you can explain?

P: If you are cheating then you have to use a condom. If you have a partner err you stay with partner maybe my husband is difficult to use condoms because maybe let's say for example we've been together for 20 years I can't say to my husband let's use protection because there's otla naganela (will think) that I'm cheating or something or whatever that is why or maybe I'm sick or whatever but maybe if I cheat or go out then I know that I have to use protection

I: Alright, so where do you get the condoms from?

P: Clinics, you buy them

I: Okay so you can't tell me of any other places that you can get condoms other than clinic and buying?

P: No

I: Okay, you only know clinics and buying?

P: Clinics, at schools, hai uh-uh I don't know

I: Is it easy to access condoms

P: Yes

I: Okay, alright do you know what we call universal test and treat

P: Universal Test and Treat?

I: Yes, they call it UTT

P: is it not when you test for HIV and then treatment is it not by the process whereby you going to be taking your ARVs or whatever, is it not that?

I: It is testing they find you positive

P: They give you treatment

I: Immediately

P: Okay

I: Yes. Alright, since accessing the facility for prevention services, could you explain how your life has been impacted?

P: err, I've seen people with HIV and it triggered my mind it has taught me not to judge people who have HIV and Aids because you will never know ore where my partner is and what he's doing wherever he is. I must never judge and I must never discriminate that this one is HIV, has HIV I must treat him/her like this, like we are all human beings we are not, even if we use protection it is not hundred percent safe ore you won't get it

I: Okay?

P: That's what I know

I: Okay, in all the prevention services that you've been accessing or having can you tell me. How have been these HIV prevention services been helpful to you?

P: It is helpful because I came here with my friend wanting to help him and he got the help. So, I am satisfied

I: And to you HIV prevention services

P: Prevention services?

I: Yes, how are they helpful to you?

P: It's all. It depends on my attitude towards this issue like I said I always have to use protection so it take you back to the attitude that you have towards HIV, if you haven't been affected then at the later stage if you do not take precautionary measures then you will become infected with it

I: Okay, so that's how it has been helpful to you

P: Yes

I: Alright, it seems like we didn't take time or much of your time like I said that it might take around 30 – 60 minutes

P: Okay

I: But, it is time for us to close this part of interview but before we do so is there anything else about this topic that we haven't discussed that you feel it is important to say?

P: Hai, I'm okay

I: Are you sure?

P: I've got nothing to add, it is just that err your company is doing good things and then must keep on helping especially black disadvantaged people who have no knowledge about HIV/AIDS

I: Alright, now we have come to an end of our discussion. Thank you very much for your participation, if you have any questions about the study participation then you can contact us.

P: I will do that.
I: Thank you very much
End Time: 14:00
