# Supplementary material for: Understanding factors influencing utilization of HIV prevention and treatment services among patients and providers in a heterogeneous setting: A qualitative study from South Africa
Source: PLOS Glob Public Health. 2022 Feb 3;2(2):e0000132. doi: 10.1371/journal.pgph.0000132 (PMC10021737; doi:10.1371/journal.pgph.0000132)
Supplement: S1 Data — (ZIP) [file pgph.0000132.s001.zip › Supplementary information/IDI_Clinic attendee_QA004.pdf]

1 PARTICIPANT IDENTIFICATION NUMBER: QA004

2 RESEARCH ASSISTANT: XXX (NAME OF RA)

3 DATE: 16-07-2020

4 CLINIC NAME: XXX (NAME OF CLINIC)

5 TYPE OF THE PARTICIPANT: FEMALE

6 LANGUAGE: ENGLISH

8 I. Now we can start, can you please tell me more about yourself?

9 P. I am XXXX (Name of the participant), I am 35 years old lady, I am from XXX (Name of Area), I am staying here around XXX (Name of Area).

10 I. How old are you?

11 P. I am 35 years old.

12 I. Are you married or not?

13 P. No I am not.

14 I. Do you have any kids?

15 P. I have seven years girl and five years boy.

16 I. Okay, can you tell me how long have you lived in this area?

17 P. I have being here since 2003 yes 2003

18 I. Since 2003?

19 P.Yes mam.

20 I. How long have you being visiting this clinic?

21 P. Since 2010 yes 2010.

22 I. Have you visited other clinics in this area?

23 P. Yes I have visited somewhere around XXX (Name of Area) it’s called XXXX (Name of clinic).

24 I. What do you like about this clinic and what do you dislike about this clinic?

25 P. I can't say there is something I dislike about this clinic since I compared all the clinics around Tembisa this one is the best. Because they does really cares if you see the person calling and reminding about the date of coming and collecting your medication, it does shows that person really cares about you.

26 I. Could you tell me whether you are HIV infected and if so how long?

27 P. Yes I am on ART since 2010 still taking my medication until now.

28 I. Are you on treatment yes?

29 P. Yes I am on treatment.

30 I. Can you tell me what are the major factor affecting your health right now?

31 P. Mmm (thinking) I back your pardon?

32 I. The major factor that affecting your health right now.

33 P. No now I can't say I have other problems.

34 I. It's only HIV?

35 P. It's only HIV but last year I was infected by TB but I did finish the treatment.

36 I. So now you are no longer….

37 P. No I am no longer taking TB treatment.

38 I. So you are fine right now?

39 P. I am fine right now.

40 I. Okay, Do you think this factor is affecting other people that you know as well?

41 P. Yah (yes) I think so.

42 I. How if you can explain?

43 P. Mmm (yes) if I can say if I am HIV positive I come here at the clinic before they give me the medication they take me to counseling, and they counsel me about everything that can happen if I am taking the medication. The side effects anything that could happen if I am taking the medication.

44 I. I heard that you mention about the side effects could you please elaborate maybe if you can mention one or two?

45 P. It’s like the side effects depends on the kind of medication you take. Because we don’t take the same medication, other side effects depends on the medication I am taking. They explain that the one I am taking now they can make my legs so thin.

46 I. Okay.

47 P. Or they can turn my eyes into yellow. But thanks God those didn’t happen to me, I still have my body the way it was.

48 I. Could you tell me your experiences in terms of service delivery from health care facility?

49 P. Yes their health delivery its good cos they explain everything, even last of last week the clinic was closed, but before it closed I got the message that the clinic is closed you can go take your medication where you see and they are so good.

50 I. When they refer you to other clinic did you find your medication or not?

51 P. Thank god it wasn’t my date of taking my medication they were just remind me if it was my date I must go somewhere to take my medication.

52 I. Okay, what are some of positive features in the facility that you have visited? And what are the most challenging features in the facility that you have visited?

53 P. I back your pardon?

54 I. What are the some of the positive features in the facility that you have visited? And what are the most challenging features in the facility that you have visited?

55 P. Ohhh this clinic is too small neh but they occupied a lot of patient because most of the people come this side but this clinic is small but they are trying by all means to cater for all of us.

56 I. Can you tell me about you experience about getting HIV care?

57 P. Yes getting HIV care is the biggest experience I ever had in my life cos I now know how to take care of myself, I now know what to do and what not to do. I know what to do if I want to have kids, I know how to keep my boyfriend safe because I am positive he is negative so I am being the kind of support to him that he must make sure that he get tested each and every six month.

58 I. I heard that if you want to have kids there are procedure that you have to follow, what procedure do you have to follow when having HIV or taking treatment?

59 P. Yah (yes) if you want to have kids you must first check for your viral load because it's risky to have kids when your viral load it's too high, and you even go check for your CD4 because those things works together if your viral load is too high and your CD4 is too low so is not good time for you to have the baby or to fall pregnant.

60 I. Okay, what are the things you would to improve about health service in the health facility?

61 P. Aaah (thinking) I think they can give them more nurses cos sometimes you come to the clinic they tell you or you will find the board written that today we have only two nurses but when you check the patient are too many in side. Some they even complain I think if they can give them more nurses or trying to make this clinic little big. So that they can occupy us all.

62 I. Okay, What do you understand about HIV prevention?

63 P. Mmm (yes) HIV prevention what I understand about it is aaah (thinking) it prevent the flow of virus in there in your blood. But it can even take it down that to the extend if you go to test your viral load will come undetected. That mean the medication has work for you.

64 I. Okay in most cases when you have sex do you use protection kind of condom or whatever?

65 P. They always tell us to use protection.

66 I. Could you tell me the different type of HIV prevention services?

67 P. Mmm (yes) I am not sure if I know any but if you are asking me about the medication I know is or four groups of type of medication, like me know I was taking that one day one pill. But if I can check my CD4 comes up and down and they gave me medication that I take in the morning and at the afternoon.

68 I. What are the some of difficulties you may experience in accessing HIV prevention service?

69 P. I back back your pardon?

70 I. What are the some of difficulties you may experience in accessing HIV prevention service?

71 P. Mmm (yes) some other patients neh they won't take their medication seriously and they blame that to the nurses like if somebody becomes rude if you are not taking your medication the right way it doesn't meant that person is rude it mean that person does care about you. But we must be grateful for that. But other people they can say why are you telling me what to do.

72 I. Do you use condom?

73 P. Yes mam.

74 I. Why do you use condom?

75 P. I am trying to protect myself and other ones.

76 I. So how often do you use the condom?

77 P. Every time I have sex.

78 I. So where do you get the condom from?

79 P. I get them at the clinic or I buy them.

80 I. What will prevent you from using the condom and what will prevent you from getting the condom?

81 P. What will?

82 I. What will prevent you from using the condom and what will prevent you from getting the condom?

83 P. There is nothing that can prevent me from using the condom, and what can prevent me from getting the condom is if the anywhere were I can get them.

84 I. So you mean every time you always use protection?

85 P. Yes mam.

86 I. Can you explain what are the universal test and treat is?

87 P. The universal test?

88 I. And treat.

89 P. Can you please explain that question?

90 I. What is the universal test and treat? UTT? Is to initiate test and treat.

91 P. Treat for HIV, neh?

92 I. Yah (yes)

93 P. Mmm (yes) it must be treated if it doesn't treated it becomes worse, and you infect the other one that you are living with and if you doesn't treat your HIV you become somebody. People wont know like this days HIV is no longer a taboo and it's no longer a secret. We share we tell each other so that we motivate each other, if they are HIV and they are hiding and you heard someone I must go and test and get my medication.

94 I. Okay, what are the some of the advantages of UTT and what are the some of disadvantages?

95 P. The advantages of UTT is people must know their status but if you are afraid of telling each other but try to tell one person you trust so that he can be the support. To remind you when to take your treatment and when to go collect them.

96 I. Has the be any changes to the were health information or health service have been delivered since the immediate ART began? That have change the way you look after your own health?

97 P. No the has never being any changes, nothing has ever change, but what has change is only my viral load and my CD4 my physical appearance has never change. (Noise in the background)

98 I. What if any issue if you experience preventing you from accessing or taking ARVS?

99 P. They have never being any issue that can prevent me from taking the ARVS.

100 I. So you mean that every time you come here you get your ARVS?

101 P. Yes mam.

102 I. Or you don't skip your medication?

103 P. I don't skip my medication.

104 I. Okay, what do you think would happen if one continues to take ART or stop taking their medication?

105 P. They will get more sick, they will get sick cos when you started the medication then stop that virus become stronger to your body. It can even kill you.

106 I. Since accessing the facility for HIV prevention service would you explain how your life has change?

107 P. Come again?

108 I. Since accessing the facility for HIV prevention service would you explain how your life has being impacted?

109 P. Mmm (yes) my life has being easy since accessing the HIV services cos I can even talk about it with others. But at first I was afraid, I was even afraid coming to the clinic taking my medication. I was even afraid of other people like my people from my neighborhood will see

me. But now I am free I am doing ever thing so feely. Because I am not the only one infected by the HIV virus.

110 I. Could you explain the HIV prevention services you think have being helpful to you?

111 P. The HIV?

112 I. Could you explain the HIV prevention services you think have being helpful to you in this clinic?

113 P. Mmm (yes) I think that rule eight thing I think has being helpful. Because I was sick before I come to the clinic, but they were there for me encouraging me to take my medication, encouraging me not to lose hope, encouraging me I am not the one infected by HIV. They were so good so good.

114 I. Meaning the… they never discourage you?

115 P. They never discourage me, they were like my sisters. They were telling me everything that why they even told me to bring my kids to do the test. Do you still remember that time when they said all kids must get tested neh? They support me and they say even if your kids tested positive is not the end of the world, they must take treatment just the way like you they will be fine, but thanks god my kids were not infected. But even from birth they never get infected.

116 I. What can you do in that case when you come to do testing for your kids and find one of your kids is HIV positive, what could happen to you maybe if you can elaborate?

117 P. I would feel bad but I will have to accept. Because there is nothing I will do, i will just take that kid is my kid. And make sure I am there taking the medication for my kid and everything will be okay.

118 I. Okay, thank you very much we have come to the end of our section of this interview. if you have anything to add in this discussion that you think it is important you are free to talk about everything you want to.

119 P. Mmm (yes) I think this interview is the best thing neh cos those nurses they don’t know if they are doing the wrong thing or the right things there. Just they are here to work, most of them they are not here to work they are here to help us, cos if you are sick see and talk with you. Some of them they even take your contact and say viginia how are you, how is the medication when are you coming? Ohh if you are sick do this and this you will be okay. That’s way I am saying this people they are so supportive. I love them.

120 I. Is that all that you can add?

121 P. Yes.

122 I. Thank you very much for your participation in this study time is 13:09, thank you very much.

123 P. Thank you.

GROSSARY UTT= UNIVESTAL TEST AND TREAT

HIV= HUMAN IMMUNODEFICIENCY VIRUS

ART= ANTI- RETROVAL TREATMENT
