# Supplementary material for: Understanding factors influencing utilization of HIV prevention and treatment services among patients and providers in a heterogeneous setting: A qualitative study from South Africa
Source: PLOS Glob Public Health. 2022 Feb 3;2(2):e0000132. doi: 10.1371/journal.pgph.0000132 (PMC10021737; doi:10.1371/journal.pgph.0000132)
Supplement: S1 Data — (ZIP) [file pgph.0000132.s001.zip › Supplementary information/IDI_Clinic attendee_QA005.pdf]

1 PARTICIPANT IDENTIFICATION NUMBER: QA005

2 RESEARCH ASSISTANT: XXX (NAME OF RA)

3 DATE: 17/07/2020

4 CLINIC NAME: XXXX (NAME OF CLINIC)

5 TYPE OF THE PARTICIPANT: MALE

6 LANGUAGE: ENGLISH

9 I. Qualitative interview time is 09: 53, location is XXX (Name of Clinic) PID QA005. Now we are going to start with our discussion please feel free to answer any question I am going to ask you.

10 I. Can you please tell me more about yourself?

11 P. My name is XXX (Name of the person) ahhh I work for XXX (Name of Employer) as a meaty functioning , I am very jolly person very open minded person. I love challenges that's me. The rule of life that I use most its meet one step low for every reaction they have a opposite but equal reaction so what means what happen on the right side must also happen on the left side so that the equation must balance that the rule in life.

12 I. So can you please tell me are you married or not?

13 P. Yes I am mam.

14 I. Do you have any children?

15 P. Yes, I have four daughters.

16 I. How old are you?

17 P. I am 38 years old.

18 I. Can you tell me how long have you lived in this area?

19 P. Mmm (yes) this area it's being about four to five years since I have being living in this area but I come from XXX (Name of Area). But from XXX (Name of Area) to here it's being five years now.

20 I. What most did you enjoy about this area?

21 P. Its peace of mind.

22 I. Peace of mind?

23 P. Yes.

24 I. How long have you being visiting this clinic?

24 P. Okay, it is my second time visiting the clinic but not as a patient but visiting a clinic it is my second time coming here.

25 I. In your first time in this clinic can you explain your experience in this clinic?

26 P. Mmm (yes) everything the staff was professional, the service was good I cannot or complain about anything, everything was on far.

27 I. How was the service maybe?

28 P. The service was good.

29 I. Have you ever visited other clinic in this other clinic?

30 P. In this area no, but by the time I was staying at XXX (Name of Area)I once visited theXXX (Name of clinic) clinic. And I had bad experience.

31 I. Can you please tell me about the bad experience you have had at XXX (Name of Clinic)?

32 P. At, XXX (Name of Clinic) clinic we were there to collect some medication for my wife. And she had an appointment of 8:00 oclock she was only attended at 15:30 in the afternoon. And no explanation was given what so ever and I was frustrated about that yes very frustrated about that.

33 I. But they end up giving you the medication?

34 P. Yes she got her medication around 16:00. From we have being there from 8:00 she had appointment of 8:00 but she got her treatment about 16:00. So for me that was not good that was the bad service and the was no apology given to us no explanation it took so long or anything.

35 I. What do you most like about this clinic and what did you most dislike of this clinic of XXX (Name of Clinic)

36 P. As I said before they have treated us with professionalism since we are here I am happy with the way that they are doing their things. They are what can I say they know what they are doing. There are what can I say everything is in order. They follow the right processes so I am happy with the way they do things around here.

37 I. Could you please tell me whether you are HIV positive if so how long?

38 P. Yes I am positive, HIV positive I tested it was 2002. Yes on the 10th of February I tested positive for HIV.

39 I. Since 2002 did you experience any side effects of this ARTS medication?

40 P. Yah (yes) at first I started taking my ARVS it was difficult yah (yes) I had drowsiness yes but now everything life is back to normal. It’s a norm to me. Everything its fine I don’t wanna lie. I don’t have those dowdiness and everything.

41 I. So you mean you take your medication daily or you do skip you medication?

42 P. No I drink daily.

43 I. Okay, Can you tell me what are the major factor affecting your health right now?

44 P. Aaah (thinking) despite the pandemic we are facing right now, the is nothing really that I can say, because I am nonsmoker, I don't drink , I exercise so it's just this covid. Just it give us a scare a judge scare. Especially if you know that you are chronic anything is possible with this covid.

45 I. Have you ever drink or smoke in your life before?

46 P. Yes I used to drink but I never smoke in my life.

47 I. What makes you to stop or to quit smoking?

48 P. No I never smoke before. Yes to stop drinking yes due to my status I wanted to live a healthy life I decided to quite a habit of drinking yah (yes) so far so good.

49 I. Do you think this factors that is affecting you is affecting other people as well?

50 P. Yah (yes) maybe they suffer from drug abuse due to stress and anxiety. And if you don't have support system in place so it is really difficult for others. So I have my family I have my wife and kids and that help me a lot I am strictly focus. I know what I need to do and I know how to maintain this healthy life and provide for my family.

51 I. The first time you tested positive did you get the support from your family members or your friends or whoever?

52 P. Okay, the first time I tested positive it took me time to accept the that I was positive, then yah (yes) it took me time the fact that I was positive so I cannot say I was getting support. Because they never knew my status so they couldn't support what they don't know so but I was positive in life but awake changes. Like they were surprise why this changes in life why this why that, why I am exercising why I have stopped drinking , why I am more responsible why yah (yes) even though I got support even if they didn't know what is going on. (Noise in the background)

53 I. Okay, (Noise at the background)

54 I. Can you tell me your experience in terms of service delivery from health care facility?

55 P. So far so good I didn't experience so much but one experience it was last month with Rwyp (inadible) so I had a blockage I couldn't pee I went in it was 02:00 in the morning so I have seen the Doctor and he assisted me and the problem is they put on a tube and they said I must come in 24 hours to remove so that they can refer me to the specialist, okay fine I went after 24 hours and fortunately they said I must reopen a new file, it didn't make sense to me why should I open a new file because they already had a file my file but they said I must open a new file. And then I asked and say if I open a new file who gonna fund for that new file they said I am going to fund that new file, and then I refused to

open a new file you know to cut the mater short I was not happy with the way they handle everything. But at the end of the day they assisted me they took off the tube. Then after I went to waterfall make an appointment to see one of their specialist. The urologist the and then I went there and everything was okay. But that part of wanting me to pay for another file it didn't make sense on me because I already have I file I didn't understand why going to the hospital and open for a new file. Of which I had an exiting file that was bad for me.

56 I. So what was your diagnosed I had you taking about tube and everything?

57 P. Okay, when I went to the specialist what we could pick up was a miner infection on top of my bladder and yes he treated it and I take a treatment of it and he said in two month if it doesn't get better, I need to go back to him so a can go to theater and see what is the main course for that. But so far I haven't experience any difficulties and no discomfort as well.

58 I. Earlier I heard you saying you never disclosed your status to your family or your friends so what makes you not to disclosed your status?

59 P. Aaah (thinking) by that time I think it was fear of the unknown the stigma and then you don't know how the people will treat you as long or as much as years is growing you find that this is a chronic diseases, is manageable so nothing to be feared. In that time I feared like they will treat me differently. They will see a different me, they won't treat me as me they will treat me as this other person who is infected I didn't want that in my life.

60 I. Can you tell me about your experience in getting HIV care?

61 P. The experience has being great I never had any problem no side effects no anything since I have taking my medication only the dowdiness when I started with the ARVS. But after three four month everything was back to normal, it is everyday thing. Normal and normal.

62 I. What are the things you would like to improve about health service in your health facility?

63 P. Aaah (thinking) if it were possible I believe if a were employed with their calling not with the sake of getting paid. It will make life easier for everyone, because if you are doing that with the goodness of your heart not for salary it make life easy for everyone even for yourselves. Think about going to work you spend nine hours at work and not happy what it kind of life style is that. But if you do something that you enjoy it makes things easier for you and it makes life easier to everyone around you as well.

64 I. Have you ever a crossed with a nurse who is working maybe with the purpose of money?

65 P. Hence I said with my experience that at XXX clinic (Name of Clinic) yes I felt like they were doing us a favour. Like they were not there to carry out their responsibility, is like when they are there they are doing us a favour helping us, and I felt disrespected and I felt devalued in a sense that even though I was not the patient there but I even recruited one of their nurses some people they are here just because they want money, and if you can report them and they lose their money maybe they are bread winners we don't know but think about it if you report them because they don't wanna do what they are getting

paid for, think about it you have being there by 8:00 just to get the medication. You stayed the whole day just to get the medication you get your medication at 16:00. It doesn't make sense, why giving me an appointment of 8:00 knowing that you are going to give me my medication at 16:00, what is the point of that.

66 I. Okay, now we are going to talk about the HIV prevention.

67 I. What do you understand about HIV prevention?

68 P. Okay, about HIV prevention you need to condomised so that they won't be a spread and spread the virus and come to terms living this it is my diseases I don't wanna share it its mine. How I contracted it it doesn't matter and now it's mine I don't wanna share it. So whenever I had sexual intercourse I make sure that I use protection.

69 I. Can you tell me the different type of HIV prevention services?

70 P. Aaah (thinking) what I now of is its condom that I know of , and if you are exposed to it the is that is raped if you are exposed within 72 hours the is drug that they can give you so that they can break the chain but after 72 hours the is nothing they can do, yah (yes) I only know of those two nothing else.

71 I. What are the some of the difficulties you may experience in accessing of HIV prevention services?

72 P. As I have said knowledge is power because lot of people they lack knowledge so they say why positive why me, why not spread by doing so you are killing the society and even you are killing yourself. Because the more you are sleeping around you don't know the other person's status you don't know the viral load for that person. You don't know anything so in a sense you are killing yourself it's like committing a society in a way.

73 I. Have you ever across with someone with HIV positive who is spreading the virus not letting people to know that he or she has that virus?

74 P. Lot of people use to do that it break my heart you know why people must share but certain things are not to be shared. Especially the virus is not to be shared if you are diagnosed with it is best to treat it, to maintain it and keep it to yourself. I believe in it I don't believe in infecting other people and creating more cases of HIV. I believe if lot of workshop are lent and awareness are made before people maybe they will think out to the box. And they say no I am positive and I was not born positive then someone gave me this and I am gonna give to somebody else. And somebody give it to somebody and then it's a chain reaction. But if proper awareness it's made. And you know that if you were not careless you got the diseases you maintain it you treat it and you keep it to yourself. And will live in a peaceful normal life. This life will be good.

75 I. Have you ever attended maybe any kind of awareness you know?

76 P. Yah (yes) I was an organization at XXX (Name of Area) that was teaching about HIV, AIDS that time I found that I was positive, and it plays a major roll to me to understand the importance of not spreading of HIV and the importance of healthy life style yah (yes) I think it help me a lot.

77 I. Can you please tell me do you use condom? And why do you use them? And how often do you use condom? And where do you get them from?

78 P. Yes I do use the condoms and I buy condoms, mostly I don't use the government one because (Noise at the background)I think I am allergic to that lubricant that they use on the government one, after using my skin is like and before I had rash and then I didn't understand why. And I consulted and I know that I had that because of that lubricant they are using. So wherever I had sexual intercourse I buy myself a rubbers, yes.

79 I. What happens if you don't have money for condoms and lubricants?

80 P. I don't play. No condom no play.

81 I. Meaning you don't do it if you don't have money?

82 P. No,no,no.

83 P. No condom no play.

84 I. What could prevent you from using condoms?

85 P. Aaah (thinking) as much as I am aware of my status, and I don't wanna share it will be difficult to me to sleep with another person without protection. It will be very difficult, I don't see myself sleeping with another person without protection.

86 I. What could prevent could prevent you from getting condoms?

87 P. Aaah (thinking) maybe if I don't have money to buy condom, lack of financial problems maybe yes. But anyway I am well paid I am on medical aid, and money it's not an issue at this time. It's not an issues to buy condoms,

88 I. Can you explain what is universal test and treat is?

89 P. The universal?

90 I. The universal test and treat is?

91 P. I don't understand the question.

92 I. The first day you tested positive what was the steps.

93 P. I was sick I went to it was XXX hospital (Name of hospital), I was staying at XXX (Name of Area) buy that time I tested positive and they took me to counseling, they explain everything and after they show me the results I said wow this is really happening. I couldn't believe it, I stayed three month then I

went again after three month to do the test again it came positive again. I went back again after six month it came positive and then I through this is reality this is my life now, then I started making adjustment and taking my treatment since that day still today I am okay.

94 I. Meaning, you don't take treatment the first day you were diagnosed with HIV?

95 p. Okay, they gave me the medication and they never explained and I never knew that the medication they gave me was the ARVS, at that stage they never told me but after six month I knew what I was taking. So it makes sense to me that why I was dizzy and everything it make sense and they explained that later not on the same time when I took the medication.

96 I. What is the advantage of taking treatment and disadvantages of it?

97 P. I cannot say there are disadvantages, the advantages is that you can live longer based on how you want it and adjust to everything. It doesn't restrict you from drinking it doesn't restrict from smoking it's a personal choice. So the advantages is you can eat whatever you wanna eat but its up to you to eat healthy. And maintain a healthy lifestyle.

98 I. And what if any issues have you experience from preventing you from accessing or taking the ARVS?

99 P. So far I had none, I had none, nothing will prevented me nothing so far.

100 I. What do you think would happen if one continue to take ART or stop taking ART medication?

101 P. Okay, I know if you stop and you sleep around without condom your CD4 could get low, and the viral load could go up. And you can be very sick and you can even loss your life (Noise at the background)

102 I. Since accessing the facility of HIV prevention services could you explain how your life has being impacted?

103 P. All I can say at first it was a mountain to tell but now it's a norm, like taking tablets for a headache or making breakfast it's an everyday thing. It's easy now because I have accepted the fact that I cannot change my status. Even though my viral load it's an detectable I still know that I have the virus and I cannot expose myself to any other infections that may bring harm to my health.

104 I. Can you explain the HIV prevention services you think have being helpful to you?

105 P. Yah (yes) the condoms yes, the condom they play a major role in my life as a prevention. Because if I wasn't preventing maybe my CD4 should went down and my viral load should have went higher. So but know I am happy.

106 I. Now is time to end our discussion but before we do that, is there anything you like to add in our discussion?

107 P. I do believe people as I have said I think more awareness is needed, most of our people they are still scared because of the unknown if they are scared of the virus, because they don't know about it, and they don't even know how to manage it. I think if the awareness has being done and then it can help. Even the stigma it can help with the stigma as well. Because lot of people who turned to spread the virus there are those who don't know. How to manage it and how to live with it. If you reach a point and you know how to manage it and how to live with it. I think it's everyday it's like flu now. Its manageable it is manageable.

108 I. Okay, thank you very much for taking time to participate in this study, is time to close our section. Time its 10:28. Thank you very much for your participation.

109 P. Mmm (yes) thank you so much.

GROSSARY

HIV= HUMAN IMMUNODEFICIENCY VIRUS

AIDS= ACQUIRED IMMUNODEFICIENCY SYNDROME

UTT= UNIVESAL TEST AND TREAT

ARVS= ANTIRETROVIRAL DRUGS
