# Supplementary material for: Understanding factors influencing utilization of HIV prevention and treatment services among patients and providers in a heterogeneous setting: A qualitative study from South Africa
Source: PLOS Glob Public Health. 2022 Feb 3;2(2):e0000132. doi: 10.1371/journal.pgph.0000132 (PMC10021737; doi:10.1371/journal.pgph.0000132)
Supplement: S1 Data — (ZIP) [file pgph.0000132.s001.zip › Supplementary information/IDI_Clinic attendee_QA006.pdf]

Full Participant ID: QA006
Participant Type: Female
Location: XXX (Name of Clinic)
Date: 17 July 2020
Start time: 10:09am
Primary interview language: English

I: Date 17 July 2020, time 10:09am, place XXX (Name of Clinic), participant number QA006, type of the interview qualitative, name of the interviewer XXX (Name of RA). Do you agree to be recorded?
P: Yes.
I: Okay, thank you. Alright, I like to remind you that the information we share here is confidential as I have already explained.
P: Yes.
I: And whatever you say here won’t be connected to you because the information that guaranteed here will be the using the PIDs.
P: Okay.
I: So remember that there is no right or wrong answer.
P: Okay.
I: Err…do you have any question before (someone talking from the background) sorry for the interruption. I was saying, do you have before we begin?
P: No.
I: Okay Err…time started; I have already said but time started 0, 10:00, 10:09. Okay. Tell me more about yourself? Can you please tell more about yourself?
P: Uhm!
I: Everything and I would like you to speak-up for the audio.
P: Should I also include err…why am I here for err…?
I: Just tell me everything about yourself, everything about yourself? Like a background check and who am I talk to?
P: Well my name is (XXX name of the participant)
I: Yes.
P: I stay here (XXX participant address)
I: Alright.
P: A international performance Artist but that is not what I came here for.

I: Okay can you speak-up?
P: Actually, am pregnant so I just came for Antenatal (ANC)
I: Alright, Okay, you said how old are you?
P: Am 25 (years old).
I: 25 Okay and you been staying here in XXX (Name of Area)y. How long have you been staying here?
P: Err… is been 2 months now.
I: 2 months?
P: Yes.
I: And where were you staying before here?
P: I was staying XXX (Name of Area).
I: Okay.
P: Yes.
I: So, are you married?
P: No but am engaged.
I: Alright that is nice. Congratulation.
P: Laughing, thank you.
I: Okay, do you have any kids ?
P: Yes, how many? Three
I: (Laughing together with participant)
P: Yes, three.
I: Three
P: Yes, actually three but
I: Three because of this one that you are...
P: Three, the other ones are twins, so that makes them three, actually I have two
I: Okay, you have three, this one is the fourth one .
P: Yes, this one is fourth one.
I: Okay, err…how have you been visiting this clinic?
P: Yes I just started today. This is by first day actually (this is my first day visiting clinic).
I: Okay, you have never come to this clinic before?
P: Yes, I have never came to this clinic before.
I: Okay.
P: So this is my first time coming to this clinic.
I: So you have 2 months, you have never?

P: Yes.
I: Okay have you ever visited any other clinics accepts this one?
P: I have.
I: Okay, where?
P: Err…XXX Clinic (Name of Clinic) around XXX (Name of Area).
I: Yes.
P: Yes, I have visited that one, I also visited XXXX Clinic (Name of Clinic).
I: Alright.
P: Yes.
I: XXX (Name of Area) is the one at XXX (Name of Area)i, not far from XXX (Name of Area).
P: Not far from XXX (Name of Area).
I: Oh alright okay, if I may ask?
P: Yes.
I: What do like about this clinic?
P: What do like about this clinic?
I: Yes.
P: Err…okay I cannot say a lot about it because is my first time today (17 July 2020) but so far I can see that the service is not poor from the okay, I how shall I put it? Okay compare to the other clinics for me this one is better, the services is better, compare those ones. Because I remember the last time I went to XXX Clinic I got there early in the morning around eighty and I left at 15:30pm.
I: Wow.
P: So it was bad for me, it was very very bad for me.
I: Okay.
P: So at least here I can see that from the time I came here in the morning until now. They have already you know.
I: Okay, alright, and what is that you do not like about this clinic?
P: The queueing outside maybe is because is COVID-19
I: (Laughing together with the participant).
P: The outside queueing ooh! No. that’s what I don’t like
I: Okay.
P: But the Nurses seem to care because they do come to check-up on us, ask that err…are you okay? What are you here for? You know yes.
I: Alright. Yes, the queues is because of COVID-19

P: Yes.
I: Alright, could you tell me whether you are HIV infected or not?
P: I am (HIV Positive).
I: Alright and if so how long?
P: Mmm (Thinking) when did I find out? When I went to XXX Clinic
I: Please speak-up?
P:Ooh, it been like I have to take blood today its been like six months now.
I: Okay . Yes. It has been six months?
P: It has been six months now.
I: Alright are you on treatment?
P: Yes am on ART.
I: Okay, when did you start it?
P: I started six months back, May, June, July. When was it? Okay what month is this? Maybe January.
I: July now so. Ooh. So you started immediately.
P: Yes, I started immediately after.
I: Oh, six months, is still six months also?
P: Yes.
I: Okay, okay, can you please tell me how do you think are the major factors affecting your health right now?
P: Uhm (Thinking).
I: Okay sorry about the interruption.
P: Okay.
I: Yes. so before the interruption I was asking what do you think are the major factors affecting your health right now? Or at this moment?
P: A this moment?
I: Yes.
P: I think maybe is pregnancy.
I: That’s all?
P: Yes that’s it.
I: Alright so you think?
P: Yes.
I: its only pregnancy?
P: Yes for now is only pregnancy.

I:Okay
P: Rather than this COVID-19 thing, rather than this COVID-19 thing outside.
I: Okay.
P: Because since I came yesterday they said I must go back home because some of the things needed here, remember err...I used to go the XXX Clinic, so had to, I got a referral to come to this clinic.
I: Okay
P: Because I relocated am staying here now. So I had to go back and its cold, like its cold.
I: Okay
P:Yes.
I: Alright, I heard talking about pregnancies affecting your health right now.
P: Yes.
I: Do you mind telling me how is it affecting your health?
P: Yoo! Am not good like am not good.
I: Please share with me?
P: the vomiting.
I: Yes.
P: The laziness.
I: Yes.
P: Yoo! I don't know to put it right but is affecting me in many ways.
I: Alright.
P: Maybe is just my body is not to used to...or its been a long time not being pregnant maybe. I don't know but right now this time my pregnant its affecting me a lot because there is a lot that is happening, I went to the sooner the other time and they told me err… am actually fabrics (fibroids) on my womb and stuff. So maybe that's one of the thing that are making me feel this way.
I: Okay.
P: Making my pregnancy like difficult sometimes am having like pains on my stomach, sometimes I cannot sleep at night you see all those unbearable pains.
I: Okay and have you, have ever experienced things that you are experiencing now on your previous pregnancies?
P: No. I haven't
I: Alright.

P:I haven’t.
I: Okay these factors do you think they are affecting other peoples that you know?
P: Yes, I think so, if maybe they are having same problem that am having right now.
I: Okay.
P: Like having Fabrics (Fibroids) on their womb maybe they are experiencing same problem that am experiencing right now.
I: Alright, alright I would like you to tell me about your experiences in.
P: Okay.
I: Service deliver.
P: Okay.
I: Not just in this facility but in the facilities can you tell me your experiences?
P: Facilities, you mean in this clinic?
I: Yes, they that you attended
P: And Yhoo! My experiences were very bad.
I: Yes, tell me about them?
P: how so?
I: Feel free start from the beginning?
P: Okay well when I was pregnant with my first baby right.
I: Okay.
P: They used to tell me bad, they used tell me that am young and you know all those things from the clinic. And all those, the queue things is something that is very bad for me I don’t like queuing for long time.
I: Okay.
P: So when I was pregnant I went to Ethafeni Clinic.
I: Okay.
P: You see there right.
I: Okay.
P: I only went once until I gave birth.
I: You never went back?
P: I never went back until I gave birth that is the only time I went back there. Then they asked me why didn’t come for check-ups and those things. And I told them that I didn’t like the treatment that you gave me. So that’s why I was home all this time. So I was going to the Doctor
I: Okay.

P: To get the supplements because I was already having my maternity case (Maternity book).
I: Okay
P: Book and my second one right the challenge that I was having on I was going to the same clinic right I went back there and I find that err…the person, okay the Nurse was the same Nurse. (Laughing) Ooh!
I: You mean the same Nurse who treated you bad?
P: Other time it was the same Nurse who treated me bad the other time
I: In another clinic?
P: No, same clinic.
I: Okay .
P: I went back to
I: XXX Clinic. Alright
P: Then I run away to XXX Clinic and XXX their problem is time.
I: Okay
P: They will tell you that appointment is at 08:00am, then I will get the at 08:00am and I will leave very late. So they don’t care whether you are pregnant or not.
I: Okay.
P: Yes.
I: Alright. So the time you were going attending clinics or these healthcare facilities. Were you going there for pregnancy or you also go there for something else?
P: No, I only went there for pregnancy, I don’t want to lie, I even uhm…yes I only went there for pregnancy
I: Okay.
P: That the only time I got the clinic, when I feel sick I just go maybe to the Doctor and come back because hate like queueing and waiting for long time at the clinics. I just hate clinics let me just honest.
I: Okay.
P: I just hate public clinics.
I: Okay I believe that in the clinics that you attended.
P: Yes.
I: They might be some positive features, what are this?
P: Okay
I: What are some of the positive features those facilitates you attended?

P: Okay, they probable like good Nurses that I don’t know but maybe I was not lucky I just got those ones who are actually rude and stuff. But yes, maybe there are good Nurse there because some peoples they go there and say, yes, its fine. So but for myself it was never fine for me.
I: Alright, so you are saying the treatment, they way you were treated.
P: Yes.
I: Err…the time
P: Yes.
I: The queuing and all were the most changing things for you?
P:Yes it was. And also, the Nurses. Okay just one Nurse the problem with one Nurse because that one Nurse was working for err…2 years.
I: Okay.
P: Okay when I was my first baby 2017 and then it was 2018.
I: Alright.
P: So, I just got the same Nurse working at that certain department ANC
I: Okay, what are the other most challenging features you experienced in the facilities that you attended? Any other…
P: Any challenges?
I: Yes, any other
P: The toilets.
I: The toilets?
P: The toilets at XXX Clinic.
I: Yes.
P: They are very dirty, is not hygiene.
I: Alright.
P: They are not hygiene that the only challenge from that facility, like that specific clinic.
I: Okay and the other one?
P: The other one no, besides that I already complain
I: Okay, this one (XXX Clinic)?
P: I cannot say much about this one because is the COVID-19 is probable this and this is my first time. I cannot say much about it.
I: Alright, alright, okay. So, I would like you to tell me more about your experience getting HIV care?
P: HIV care?

I: Yes.
P: Okay, since I started with my, with my err…with the medication right.
I: Okay.
P: Yes. Okay first month I went there right, and everything was fine. And second time I came, yooh! You know one of the Nurses, like I don’t know maybe if they didn’t want to work or something, that’s am saying maybe myself maybe am not lucky like good Nurses most of the time.
I: Okay.
P: Because when I went there I was supposed to get my medication, then first time it went all well now they took my blood and what not.
I: Okay.
P: I came for the second time now they told me the results but then the Nurse was also rude to tell me like everything, I mean don’t really understand everything about HIV am new on this.
I: Okay
P: Would like to know what do they mean about the viral load, undetectable or whatever they were just explaining to me
I: Okay.
P: I don’t t really understands so the Nurse was bit rude.
I: Okay.
P: Towards that right.
I: Yes.
P: When I came for the third time, third time they just gave me medication for two months, they did ask me what was wrong. And they did call me two weeks after my medication and say okay how is medication treating you?
I: Yes.
P: You know are having any problems. You know then okay told them that okay its fine, still fine the only thing that am experiencing is just rush on my face that was it.
I: Okay.
P: They say okay its normal, but it will just go away okay.
I: Yes.
P: Fine then I came for the third time, when I came for the third time. Then, I wanted to ask again because want fine I think I was an infection or something like that. Then, I asked the Nurse, okay what do I do if I have the infection or maybe because for the

first time, I wasn't experiencing this kind of the problem. So is it because of the medication or what? And then, she was like okay your infection has nothing to do with the medication.
I: Okay.
P: Okay, so what could be the problem? Then, she said like I don't know.
I: Yes.
P: Can't you check, or you know what the problem with me is?
I: Yes.
P: She was like no am not going to telling you anything Sis (Sister or Madam) I don't have time right, did you check the time? Then, I said yes I did check the time and its half past two I want to go home, here is your medication. She gave me two right.
I: Yes.
P: For two months and she gave me the date (return date for collection of medication) come back on this date. That's it.
I: Alright.
P: So what is that.
I:Yes, I hear you.
P: I couldn't understand why she doing this to me or maybe we are boring then because we are already infected and the don't wanted infected peoples. I don't know.
I: I cant answer that.
P: (Laughing)
I: I can't for them.
P: Yes that. So yes. That's it.
I: Alright thanks you. So what are the things you would like to improve about this facility or other facility?
P: You know what I would like right?
I: Yes.
P: I would like them to have specific peoples who are working with err…specific category of sickness or like you see here right they chronic peoples are on one side like you took me from the line you said pregnant you are going the side for ANC and I did not know I just waiting there on the line and they didn't ask us, they didn't do anything.
I: Yes.

P: I wish they could maybe change that. You know just let us know when you get here they must tell you, when you get here they must tell you that you must go this side what are you here for just go this side so that you don’t queue on the wrong line
I: Yes.
P: Yes. That I would like them to change otherwise just.
I: So nothing else that you think can be?
P: Can be improved rather than to get here but from other clinics there is a lot like.
I: Okay tell me about it?
P: XXX Clinic their toilets need to be you know maybe that’s where we take the infection from we don’t know at least need to be hygiene, its clinic need to be hygienic all the times they must keep it clean. The glass were they, you see they give you a glass when you are going to the toilet.
I: Yes.
P: The urine and stuff you will get them there on the same water they don’t even change you can see right that err…they don’t even change.
I: Alright.
P: That too.
I: But you said that’s a lot?
P: Yes that’s a lot.
I: We have time, I want you to tell me everything.
P: And the Nurses, I just wish right .
I: Yes.
P: The peoples who are working at the clinic like the Nurses they just like follow they dreams sometimes I feel like this person at work you know .
I: Yes.
P: Working and after work its gonna paid and go home that’s it.
I: (Noise at the background) Am sorry about the interruption again lets continue. Now were going to talk about the HIV prevention.
P: Yes.
I: Okay what you understand about HIV prevention?
P: Mmm (thinking) okay according they have explaining to me.
I: Please speak up?
P: Err… what they have explained to me right?
I: Yes.

P: They said that HIV prevention uhm… doesn't   make HIV to go away.
I: Yes.
P: But it suppresses the virus right. That's what they told me. So, what I understood
I: Prevention or treatment?
P: Okay HIV prevention oh my bad. (Laughing).
I: Okay. You can start over?
P: Okay what do I understand about HIV prevention?
I: Yes.
P: Is like you asking me what is HIV prevention?
I: Yes.
P: (Laughing) Okay err…HIV prevention is about preventing yourself from getting HIV
I: Yes.
P: You using err…certain err…contraceptives maybe protection like condom, can use that to protect yourself from getting uhm…HIV.
I: Okay.
P: Uhm…making sure that you don't someone's blood.
I: Okay.
P:If you know that someone uhm…has okay if, if someone is having blood like somewhere you cannot touch with your hands you use like gloves to prevent like getting HIV because you might never know if this person is infected or not.
I: Alright.
P: Yes.
I: Other than using condom?
P: Yes.
I: And not touching someone blood. What are other types of HIV prevention?
P: Err… okay am not sure about this one but am sure that err…probably immediately finding out that you are have had err….an intercourse.
I: For the interruption should you were saying something?
P: Yes. I was saying something, I was saying that err…we on prevention, right?
I: Yes.
P: Am saying that am not sure about this one but right uhm…that after having intercourse maybe after find out that you have had an intercourse with err… an infected person you can always go the nearest clinic so that maybe they can give you something for that. But I don't know for how many hours, minute or days.

I: Alright.
P: Or something like that. Those are other ones.
I: Do you know the name of that something?
P: No, I don’t it, actually I was about to ask?
I: Ask who? Me?
P: From the Doctor now of what is that?
I: Alright. Okay, please do ask?
P: Okay.
I: But are different types of.
P: But what I understand is that he can only got infected through blood, anything that has to soothing do with blood.
I: That’s what you understand?
P: Yes. Not, really blood, salver as long.
I: (Someone talking from the background) Sorry for the interruption.
P: Okay.
I: I was saying when you to the Doctor.
P: Yes.
I: You can ask every question that you need to understand everything about HIV prevention .
P: Okay.
I: HIV treatment. Ask everything do not be afraid
P: I just wish I get an understanding Nurse this time again.
I: Okay what do you think are some of the difficulties you may experiences when accessing HIV prevention services?
P: Mmm (thinking) please repeat the question?
I: I mean what do, what do you think err…are some of difficulties?
P: Yes.
I: Like what can be difficult for you to accessing HIV prevention services?
P: Mmm (thinking and laughing) Should I really say it?
I: Say it.
P: Laughing,
I: I wanna hear it.
P: Okay what do you mean accessing?
I: Like having access

P: Yes.
I: I mean something that can stop you from getting HIV prevention services
P: Like there nothing that can stop me for getting HIV prevention from getting HIV prevention.
I:Nothing.
P: There is nothing. I can always come to the clinic and get prevention.
I: Okay, okay if I may ask, do you sue condoms?
P: Yhoo! Yes we do sometimes (laughing).
I: Okay you don’t use them sometimes?
P: Not sometimes, everyday we use condoms, but sometimes you know we do forget. We don’t forget actually but.
I: Just do not you.
P: Intentionally but we just do not use it yes.
I: Okay.
P: I think the reason why am pregnant again now because if I was you know stable like using condoms, I wouldn’t be pregnant maybe right, even though is not 100% but maybe the risk of being pregnant
I: Okay, can I ask you something?
P: Yes.
I: Why do you use condoms?
P: (Coughing) okay according to me I use err…condoms to protect myself from getting err…infections.
I: Okay.
P: Not specifically like HIV but err…STIs (Sexual Transmitted Infections) as well and from prevent myself from getting pregnant again because I have, I didn’t want to
I: Yes
P: To be pregnant like, I didn’t want to plan to be pregnant like this time.
I: Alright,
P: (Coughing) Maybe is Corona(virus)
I: Sanitize,
P: (Laughing) Yes.
I: Please, sanitize before we can continue. Okay this condom that you use where do get them?
P: We buy them us, us we buy the condoms.

I: Okay
P: Because the last time I, I used like condoms from the clinics the one that I get from the clinics I think I was allergic to them.
I: Okay.
P: So every time after using that one after intercourse maybe I was bleeding. I used to bleed so.
I: Alright.
P: When I went to the Doctor the Doctor said (that) try changing the protecting that I was using. Then I started buying them.
I: Okay other than buying condoms. Where other place that you get condoms?
P: (Thigh) condoms, condoms are everywhere you get them from the clinic.
I: Okay.
P: From the err…Spazashop, pharmacy sometimes you know yes.
I: Okay. What do you think can stop you from using the condoms?
P: No, at this moment nothing can stop me (laughing with interviewer). Maybe if I wasn't err…HIV positive, maybe I would say, I can stop but now nothing can never stop from using protection (condoms).
I: Alright, alright and then what do you think would prevent you from getting condoms?
P: What could prevent me from getting condoms? How so?
I: Yes, what can stop you from getting condoms, you need condoms and you cannot have them. What do you think can prevent you?
P: From oh your question is…don't understand it.
I: I want to understand if now you want to get condoms
P: Yes.
I: And you cannot get them. What do you think standing on your way?
P: Money.
I: Okay.
P: if that what you mean because always buy them. So, if I don't have money obviously I won't able to buy, to get condoms. Then again, I have to go the clinic get those ones but obviously because am allergic to those ones as well. I would not be able to get them.
I: alright
P: You are not recording, are you? Oh okay.
I: Thanks, you. You thought it sopped? Is still recording.

P: Okay.
I: Alright can you explain what is Universal Test and Treat (UTT) is?
P: Universal test and treat?
I: Yes.
P: UTT.
I: Yes.
P: I know that uhm…isn't they, the err… the okay you know when you test for, for HIV testing.
I: Yes.
P: There is that the first one, the UTT is not the second one the confirmation for. Eish I don't know.
I: You do not what the universal test and treat? Okay also think when you go to the Doctor ask about this things.
P: Okay.
I: Okay universal treat is when you test, and you start the treatment
P: Okay.
I: Immediately you don't have to wait for CD4 count to drop.
P: Start the treatment immediately.
I: Yes, what they are doing now.
P: Okay.
I: You know, that right?
P: Yes. No, I did not know the term UTT.
I: Yes.
P: Universal testing what, what. It was similar to those when they explain those err…you see my the first-time doing HIV testing.
I: Okay
P: What do they call the first one?
I: Mmm (Thinking).
P: Ehen talking my blood.
I: Its rapid test.
P: Yes.
I: Yes, okay what are the advantages of UTT?
P: The advantages of UTT is that if you start immediately uhm…probable the chances of you of getting more infected can be slim than maybe testing today and mmaybe you

getting the HIV positive and you don't start treating yourself. Then, wait for maybe the time you start getting sick, you start getting symptoms you know.
I: Okay.
P: Yes.
I: And some of the disadvantages?
P: The disadvantages is that obviously when you start your treatment your body is not used to the treatment. Its like a drug to you.
I: Okay.
P: Sometimes you vomit, sometimes you feel otherwise your body the dizziness yes.
I: Okay.
P: Those disadvantageous.
I: Alright, thank you.
P: The facts that there is possibility that they may not treat you well.
I: Alright.
P: Oaky UTT.
I: Sorry for disruption.
P: Okay.
I: Continue
P: Yes, I was done.
I: Alright, has there been any changes to the where health information or health services has been delivered since immediate ART?
P: Okay, is there?
I: Any changes err…to the way health information has been delivered to you? (noise at the background) Sorry for interruption again, so I was saying, is there any change way health information or health service has been delivered to you since the immediate ART? Like it has, did it change the way you look at your on health, the way you look after your on health?
P: Yes, yes it did.
I: How did (so)?
P: Uhm…okay there are some of the things that they advise not to eat after like taking, after you have started with your ART.
I: Yes.
P: Like err…one of those thins am must maybe err…stop drinking a lot of coffee .
I: Yes.

P: Err…I must stop err…eating ginger, no not ginger, what is it? Gallic, they said is not good for when am taking like, when am on ART. I don't have to eat those things.
I: Yes. Alright
P: Those two I must stay away from them and I was drinking a lot of coffee before. So for me it was a big challenge, it a bit challenging because I had to.
I: Okay. Uhm…what issues has you experienced, experienced that prevented you from accessing you from ARVs or taking ARVs?
P: What, what?
I: Issues Have you experienced that stop you from accessing ARVs or made you not to take ARVs?
P: Err…there are no issues because (someone talking from the background)
I: Uhm…sorry for the interruption should I repeat the question or?
P: Yes please.
I: I was asking, what do you think can be the issues that just stop you from taking ARVs or preventing from accessing ARVs?
P: Okay at this moment there is nothing that can stop me like really because at the end of the day my help. So I have to take care of myself.
I: Alright.
P: Yes.
I: So, what do you think it would happen if one continues to take ART?
P: Uhm…(laughing) it will lower the risk of err…making the HIV virus like worse.
I: Okay.
P:Yes.
I: And then, it will lower the, the viral load as well. It can lower the viral load and it can also make you like your CD4 count like stay stable at the correct place.
P: Okay.
I: And what will happen if you stop?
P: You will die (Laughing together with interviewer) uhm….it very risk. You might die because you are making it, its like you are making it worse. If you don't know, okay it better if you don't know but if you know.
I: Okay.
P: I think its can me more worser because now you know that there is something wrong with you
I: Okay.

P: And you are not taking care of it
I: Alright.
P: So your viral load can go way million, trillion what, what. (laughing together with interviewer) higher
I: Okay.
P: Yes.
I: Alright since accessing the facilities for HIV prevention services, can you explain how your life has been impacted?
P: Repeat the question?
I: I mean since accessing the HIV prevention.
P: Yes.
I: In the facilities.
P: Yes
I: So could you explain how your life has been changed? Impacted could be good or bad.
P: Okay err…from the bad side (laughing) I was not used to the timing like taking my medication right.
I: Okay.
P: So I had to get used to the time maybe if they say I take my medication at eight I have get used of taking medication at eight. And sometimes I would like to forget to take them at that time. And remember after eight maybe at nine. And if I don't have my medication with me sometimes maybe I went to mommy place just for today maybe I decided to sleepover and I didn't bring my medication with. That's where the problem like because like I will be skipping taking may medication.
I: Okay
P: And then on the good side like is only affected me for that period of time. I had challenges for first two weeks. Then, after that, it was easy for my body to adjust to medication. So there is nothing wrong is like normal life, back to my normal life, normal life.
I: Alright.
P: Yes.
I: When it comes to preventing?
P: Yes.
I: Err… the prevention service the one that makes me, the one makes from.

P: Getting HIV.
I: Or infection
P: Infection.
I: So can you tell me? Could you explain?
P: The good and the bad as well.
I: Yes.
P: Okay the bad side is that uhm…I was probably not err…using that protection.
I: Speak up please?
P: I was actually (laughing) okay probably I was not like use protection, the protection. So uhm…it different like we have sex now like protection now. Like compare to that time I was not using protection.
I: Okay.
P: The intercourse its different.
I: How different?
P: How different?
I: Yes.
P: (loudly laughing) I can't say that (laughing). Okay well I cannot answer that one.
I: Alright, no problem. Okay, can you explain the HIV prevention services. Do you think it was helpful to you? How do you think it has been helpful to you?
P: They has been helpful to me because uhm… from…
I: From?
P: From the period I started taking my medication until now.
I: Yes.
P: The, the difference that is see is like I used to have maybe, when am having like the infection it could take time like to heal.
I: Okay.
P: But since I started with my medication is no longer that bad. So I could see that at least the medication has, its fighting somewhere.
I: Alright, okay, you spoke about the treatment helping you and then about the prevention.
P: Okay about the prevention is has help a lot because okay most of the time maybe like I could have like err…sex without using protection (condoms) and I think err…. it's cool.
I: Okay.

P: Forgetting about the HIV because most of the time in the mentality I could tell myself that okay am not going to have sex without err…using protection because I know am gonna be HIV or am gonna be pregnant.
I: Okay.
P: But it help me because I know not about that.
I: Okay.
P: There is also some things that called uhm…Sexually Transmitted Diseases (STDs).
I: Alright.
P: Yes.
I: Okay it time for us to close the session.
P: Okay.
I: So but before we do so. Is there anything about this topic that we have been discussion and you feel is very much important to say?
P: Yes.
I: Okay please?
P: Okay when it comes to uhm…when it comes to HIV right.
I: Yes. I feel like, when it comes to HIV I feel like we should have uhm…each and, no not each and everyone but they should be a specific group right.
P: Okay.
I: Of peoples maybe one Nurse working err…specific group of peoples to help them because its I, I feel like is not easy for one to, to overcome the whole situation even if they already gave you the. What do they call it? The
P: The counselling.
I: The counselling okay.
P: They give you the counselling right .
I: Okay .
P: But is not easy even after counselling is not easy for one just like start. You know you have that positive mind to say am just taking my medication it going to be okay. Its not easy this thing needs yooh! Its torturing.
I: Okay.
P: You know its very, very torturing for that matter.
I: Okay.
P: so maybe one, one Nurse or Doctor or whoever or whoever who was counselling must make follow-up on those kinds of peoples. I feel like that's why of the other

reasons why peoples stop taking their medication. They will start first day, the second day, and then maybe the medication is taking time to adjust to their bodies. And they just leave them.
I: Okay. Alright
P: So they don’t care sometimes. If you came like this month (July 2020) they ask you are taking your medication and they say yes (and you say yes). Okay they give you another pack another one you know.
I: They take your word for it.
P: So the what?
I: They take your word for it?
P: Yes, they take your word for it not easy am not saying that they should force peoples to take their medication, but they must try to find other ways to make it easy. For the patients to take their medication because is not easy like its trauma (tic)-very traumatic
I: Okay.
P: Its traumatizing get that you are HIV to start with, after they just told me that am HIV positive like today.
I: Okay.
P: I felt like it was the end of my world.
I: Okay.
P I tried to be fine, to act stronger and but I could feel like you know what I feel like ma dying now. I started like from same day after testing. I started taking my what do you call?
I: Okay.
P: UTT the one just ask me about.
I: Okay.
P: I started taking my medication like immediately.
I: Okay.
P: And it wasn’t, it wasn’t good because my body is not used to, to that kind of the drug (HIV treatment mediactaion).
I: Okay.
P: You know. So if, if it was somebody else maybe he/she was going to say no what am not gonna take that. This thing making crazy. Sometimes you just dream.
I:Okay.
P: Bad things at night, at night because of the medication.

I: Alright.

P: So its not.

I: Alright.

P: Follow-up, they need to follow-up with their patients whether they are talking their medication and how it is treating them. And make sure if possible test (ask politely them to find out) them just to see if they are taking their medication correctly.

I: Okay.

P: Because sometimes you skip because you know iesh if am taking these. Okay imagine if I was on the state, I wasn't able to share with my partner right.

I: Okay.

P: Right need to take my medication every day. I just tested positive today and I have to start taking my medication. And haven't disclose to my partner.

I: Okay.

P: Do you understand?

PI: Yes.

P: We stay together. So what I was going to do because it was going, it was going to be very difficult for me take my medication even. I have to hide it, because he doesn't know am thinking maybe if I can tell my partner gonna act this way. Do you understand?

I: Yes, yes.

P: Yes, so for my side it wasn't that bad okay due to some reasons maybe I cannot say. It was my first person. So, I would say, I wasn't busy (sleeping around) before, haven't dated before. Its was the first person I ever sleep with.

I: Okay.

P: So maybe that's it was easy for my partner to understand that. Okay you are HIV positive how come?

I: Okay.

P: Yes, understand that so maybe.

I: Alright. So now we have come to the end of our discussion. Thanks for your participation

P: Okay.

I: If you have any question the or about the study participation please contact us.

P: Okay.

I: Thank you very much.

P: Thanks you.

I: And time is 11:27am.

Glossary

Antenatal=ANC

Fibroids are abnormal growths that develop in or on a woman's uterus. Sometimes these tumours become quite large and cause severe abdominal pain and heavy periods

Sexual Transmitted Infections=STIs

Universal Test and Treat =UTT

Sexually Transmitted Diseases =STDs
