# Supplementary material for: Understanding factors influencing utilization of HIV prevention and treatment services among patients and providers in a heterogeneous setting: A qualitative study from South Africa
Source: PLOS Glob Public Health. 2022 Feb 3;2(2):e0000132. doi: 10.1371/journal.pgph.0000132 (PMC10021737; doi:10.1371/journal.pgph.0000132)
Supplement: S1 Data — (ZIP) [file pgph.0000132.s001.zip › Supplementary information/IDI_Clinic attendee_QA007.pdf]

Full Participant ID: QA007

Participant Type: Male

Location: XXX Name of clinic

Date: 17 July 2020

Primary interview language: English

Label Key

I = Interviewer

P = Participant

N = Notetaker

I: Thank you for agreeing to be part of this interview. I'd like to remind you that every information that will be displayed here will be confidential, it won't be connected back to you. So, remember that there's no wrong or right answer, and also remember to speak up so that it can audible on the audio recorder. Do you agree to be audio recorded?

P: Yes

I: Thank you very much, do you have any question?

P: No

I: Before we can begin?

P: No

I: Okay, let's begin. I'd like you to tell me more about yourself.

P: How do I feel?

I: I'd like you to tell me more about yourself

P: Like what?

I: Everything, who are you, how old are you? I just want to know you; I want to do a background check on you

P: I'm (xxx name of the person) I'm coming from XXX (name of province).

I: Alright,

P: Yes

I: And how old are you?

P: I'm 40 years old

I: And are you married?

P: I'm married, I've got 4 kids

I: You've got 4 kids

P: Mmm (yes)

I: Okay, can you tell me how long have you lived in this area?

P: In XXX (Name of Area)?

I: Mmm (yes)

P: Is now 20 years

I: 20 years

P: Yah (yes), since 2000

I: Since 2000 you’ve been staying here?

P: Yes

I: Okay, and how long have you been visiting this clinic?

P: I started visiting here 12 of 12, 2012.

I: Okay triple 12

P: Yah (yes)

I: 12 December 2012

P: Yah (yes)

I: Alright, have you visited other clinics in the area?

P: No

I: Only this clinic?

P: Only this one

I: So, you don’t know how other clinics are like?

P: No, only this one

I: Alright, so tell me what do you like about this clinic?

P: No, this clinic I like because is near to where I’m staying

I: Uh-huh (okay)

P: Mmm (yes)

I: Only that?

P: Only that

I: For services wise?

P: Is good, is good

I: Okay, and then what is that you don’t like about this clinic?

P: There's nothing that I don’t like. They respect the patients; they give right pills to the patients and they treat good.

I: Alright so can you tell me whether you are HIV infected?

P: (not audible 03:20)
I: Can you tell me whether you are HIV infected?
P: Whether?
I: You are HIV infected or not?
P: Yes, I can tell you, I am HIV infected.
I: Alright, you are a HIV positive
P: Mmm (yes)
I: And then is for how long?
P: Is now 8 years
I: 8 years
P: It is supposed to be 8 years because I started in 2012, 12 of December
I: Alright, alright, and then are you on treatment?
P: Huh (Pardon)?
I: Are you on treatment?
P: Yes, I take the treatment
I: Okay, since when?
P: Huh (pardon)?
I: Since when, when did you start your treatment?
P: In 2013 January, 16 of January the time I started to take my first ARV tablets
I: Okay
P: Mmm (yes)
I: 2015?
P: 2013, after 2012,
I: December then January you started
P: Go home, they gave me only the, they said that they can only give me vitamins,
I: Okay
P: Then they said I should come back on 16th of January
I: Alright
P: Then I come here they started to give me treat… that tablet you remember yah (yes)
I: Okay what do you think are the things that are affecting your health right now?
P: What are?
I: Are the things that you think that are affecting your health right now?

P: No, I don’t know. I think I am alright (Not audible 05:04) I don’t think there's other thing disturbing my life

I: And not only on HIV but in everything in your health

P: No, I’m well, since I started taking this tablet then I’m well

I: Alright, so do you think there are other any factors that you know of that are affecting people that you know, that are affecting their health?

P: Like this thing of Covid 19

I: Uh-huh (yes)

P: Yes, I’ve heard people talking about those things and I’ve never ever meet the person with it

I: Okay

P: Yes, even now I’m still living without it, yes but there's more people that have this sickness and have to be very safe, not next to other people, wash hands and do what they need

I: Alright, when it comes to service delivery in this facility

P: Here?

I: Yah (yes) what is your experience?

P: No, I think, I think there’s nothing that I can talk about this clinic because I’ve never ever come here find the tablets, they say the tablet is not there

I: I’m talking about good or bad service delivery

P: Service delivery

I: Yes, in general, it doesn’t matter whether is good or bad but just tell me service delivery in general in your experience

P: No, is good

I: How is it good, tell me about it? Tell me everything

P: That’s why I said there's no day I will come here and find that maybe there is nothing, no tablets, no medicine and other things. If you come here, you get your things and then you go back. There's nothing that I can say maybe one I came here, and they told me that there is no tablet. Always when I come here, I find my medicine and go back home

I: Okay, do you only come here to collect HIV treatment?

P: Only

I: Only, you never came here for any other sickness?

P: No

I: Okay when you check when others are here and you are waiting for your treatment, how do they do things?

P: How do they?

I: How do they deliver service when you are observing, when you are checking others while you are busy waiting for your treatment?

P: I don’t know, I cannot say I know, I don’t know. According to myself I good that’s why I like to come here

I: That’s why you are always here

P: Come here

I: Okay, you said that in this facility there’s no day that you will come and not get tablets or not get your treatment. Is there any other good thing about the clinic except for getting the treatment all the time when you come here?

P: I cannot comment much about that because I only come to take this tablet only

I: But any other good things that you see when you are at the clinic?

P: Err old person or disabled come first

I: Okay

P: Is a good thing

I: Alright, any other thing?

P: Other things, their clinic is clean

I: Okay

P: And also, they treat us very good

I: Alright,

P: Mmm (yes), is all about that, that’s all about it

I: Okay, so, can you please tell me more about how you get HIV care here?

P: How I get?

I: Your HIV care

P: Here?

I: Mmm (yes)

P: When I come here, I have to queue like other people, they collect our cards, and then they go fetch our files then they call us in order, then they give us the medicine, then we go back. They write on the file, I don’t what they write on the file, but they write on the file then we go back, no problem.

I: Okay,

P: Mmm (yes) then after 3 months they were taking blood but now they take blood once a year

I: Alright

P: They give you tablets, 11 months then the last month of the year they take the blood

I: Alright, so last month of the year

P: Yes, the time we were taking 3 packets of tablets they were taking blood continuously after 3 months, 3 months, but now because the tablet is one they take blood once a year but now I came here they said I must come to take blood on the 15th of January

I: Okay

P: Mmm (yes)

I: Alright, so tell me here what are the things you would like to improve in this facility?

P: In my life or here?

I: Yah (yes) here in the facility

P: Err, the building is nice, the building is fine, they got security, I don't know other things they can improve the clinic with but security is there, the building is good, it is clean you see

I: Okay you don't how they can be improved?

P: No

I: So, what do you think are the challenges?

P: Here?

I: Yah (yes)

P: The challenge is only a queue when I come here I can find maybe I'm number 50 then I must wait until maybe 50 people, they must be served (noise on the background, not audible 12: 23). Only challenges

I: And then how do you think that can be improved?

P: I don't know because people are many

I: Okay

P: Yah (yes) and they need to be served like other people

I: Alright, can we talk about HIV prevention now

P: HIV prevention?

I: Yah (yes), so what do you understand about HIV prevention?

P: Is only if you can abstain, or you can use condoms to prevent HIV

I: Alright,

P: Mmm (yes)

I: Okay, I can hear that you are talking about types of prevention services; of HIV prevention services which is abstaining and/or using condoms, are there any other different types of HIV prevention services that you can tell me?

P: No, I don't know about it, is only to use condom and abstaining only

I: Alright, what could be some of the difficulties for you to access or for anyone to access HIV prevention services?

P: You repeat?

I: What could be some of the difficulties that can make one not to access HIV prevention services?

P: It is just because maybe other people they say they want to have a child, they want to married or other people they just want, they know about, they have HIV positive, they want to infect other people so that they cannot be killed alone, they must die with other people. You see the other people are like that so if you do like that it means that (not audible 14:41). If you are infected don't infect other people.

I: Okay, do you use condoms?

P: Always, (not audible 14:59)

I: If I may ask, why do you use condoms?

P: Just because I don't want to re-infect myself

I: Okay

P: Mmm (yes)

I: How often to you use them?

P: How?

I: Often do you use them; how often do you use condoms. When and when do you use condoms?

P: Err only when I'm at home

I: Only when you are at home

P: Yah, at night and then when we go to the bed, I make sure I've got some of the condoms with me so that I cannot re-infect my wife and not re-infect myself also

I: Alright, where do you get these condoms?

P: Right here

I: At the clinic?

P: Mmm (yes)

I: Okay

P: You see now I've got 6 packets (not audible 15:56) I've got maybe one packet left then I collect others

I: Okay, other than getting condoms at the clinics where are other places where you can get…?

P: If I don't have enough, I have to go buy

I: Okay

P: Only

I: Okay

P: Mmm (yes)

I: So, in other words condoms you can get them at the clinics or buying because I heard you say you have to buy, if you don’t have condoms you have to buy and you ended up saying only. So, buying and clinic are the only places where you can get condoms?

P: Huh (pardon)

I: Buying at the shops and clinics are there only places where you can get condoms?

P: No

I: Where else can you get them?

P: I can go to hospital

I: Okay

P: I can get it to other people those coming from government

I: Okay

P: I can also get it from mobile clinic

I: Alright

P: Mmm (yes)

I: Mmmm okay, how is it to access the condoms, how is it?

P: To use it?

I: Huh-uh (no) to get them, how difficult is it to get the condoms?

P: It is not hard, is simple because spend nothing to get it unless if you are going to the shop but if you are not going to the shop, this is free

I: Alright

P: Mmm (yes)

I: Err what would prevent you from using condom?

P: What?

I: What would stop you from using condom?

P: No, there’s nothing that can stop me from using condom

I: If you were to find yourself not using condom what will be the reason?

P: I am not going to have a reason of not using it

I: Alright

P: Mmm (yes)

I: And then what would be the reason to stop you from getting the condoms? If you need them then find that you can’t get them what would be the reason not to get them?

P: To get them?

I: Not to get them (condoms), what would be the reason not to get the condoms?

P: I don’t have the reason because if I don’t have the condoms I must go to the nearest clinic and I find them

I: Alright, can you please explain to me what the Universal Test & Treat is?

P: What?

I: Universal Test & Treat, what is it?

P: I don’t know

I: You don’t know it; you’ve never heard of it?

P: Universal?

I: Test & Treat (UTT) that thing of being tested and get the treatment right away, immediately

P: I don’t know, I thought maybe you talk about that thing they use it

I: So, can you tell me what’s good about being tested now and then they find that you are positive then they initiate you on the treatment same time?

P: Is very hard

I: What’s good about it?

P: Is very hard

I: How is it hard?

P: You cannot believe that you are in the row of those people who are going to take the tablet every month and you are going to live from this the whole of your life. It is going to be hard to you, it is going to give you stress. The good thing is only that they can take you to counselling. They are going to counsel you and give you some ideas, how can you do, you see? Don’t worry, you are not alone, other people are doing this, they’ve got many years living with this thing then you come and recover from yourself

I: Okay

P: Mmm yes

I: Okay, since you have started ART, since you have started taking your medication has there been any changes to the way they’ve given you the information?

P: Huh-uh (no), change of what, of life?

I: How you look after yourself?

P: No, the people don’t know about my status, you can maybe say those people they know,

I: No, I mean you, on yourself

P: No, nothing changed, you eat nicely, you sleep nicely, you recover nicely

I: So, before you started treatment were you doing the same thing?

P: Like?

I: Like I’m saying before you started the treatment, you were living a certain life, after you have started the treatment, is it still the same life as after you have got the information? So, I want to know

P: There's a change

I: What are the changes there, what changed?

P: The changes start that they must know that the cond- (not audible 20:47)

I: You must do what?

P: You must use it always when you are going to have sex

I: You mean condom?

P: Yes, and you must make sure that everyday you drink one tablet at night

I: Uh-huh (yes)

P: That's the changes of your life.

I: Okay, Anything else?

P: Nothing else, you get yourself they you were, nothing is going to be difficult

I: Alright, tell me, what are the issues that you experienced that stopped you from getting HIV treatment, I mean from getting ARVs or drinking them?

P: To stop?

I: That have stopped you or made you not to get HIV treatment, I mean the ARVs?

P: If I don't?

I: What are the issues that you experienced, that you came through, what made you not to get HIV treatment?

P: I don't understand

I: Okay let me rephrase the question

P: Mmm (yes)

I: What have you experienced, that prevented you from accessing ARVs?

P: What did I experience?

I: Yes, what have you experienced?

P: From medicine?

I: No, from getting them, what stopped you from getting the medication?

P: Err I don't know; I don't think there's something that can stop me to get the medicine

I: Okay, you haven't…

P: I know is like battery

I: Is like what?

P: Battery, when is flat you must charge it

I: Okay

P: If you don't charge it, it will shut down

I: Alight

P: So, you must always have a tablet to charge yourself, I don't know whether I'm wrong or write (giggles)

I: Okay, okay

P: Mmm (yes)

I: And then what do you think will happen if one stops taking treatment?

P: Stopping?

I: Taking treatment, if one will stop taking treatment

P: It is going to be hard; it's going to be hard

I: How hard, what do you mean is going to be hard?

P: You are going to suffer from our lives

I: Tell me about it

P: We are going to lose our body

I: Uh-huh (yes)

P: We are going to be shameless

I: We are going to be?

P: We are going to be shameless; we are going to start to go with 2 bars or 1 bar

I: What are 2 bars and 1 bar?

P: We going to start go slowly then and getting old little bit, little bit, little bit after maybe 2-3 years you gone

I: What is 2 bar – 1 bar?

P: When I'm collecting this one is like I'm collecting my batter charge (laughs)

I: But I want to understand 2 bar and 1 bar

P: 2 bars, when I'm not drinking this thing the battery goes down little bit, little bit. If I stop drinking these tablets I'm not going to live, I'm not going to survive as I know it

I: Okay

P: Because (not audible 25:35) with the problem of the legs those legs were swollen, everything was difficult for me, my body was paining, there was no, I was powerless at that time. When I start to come here, they gave me medicine, I pick up little by little, now I'm good, I can go to work, I can do other kind of job. Like any other person

I: Alright, and then what do you think will happen if one continues taking medication?

P: If?

I: If I keep on taking my medication what will happen?

P: No, I'm going to, it is going to make me to be strong, yah (yes) you continue life because if you do not take it, I'm telling you brother you not going to live. Now I've got, it's almost 8 to

9 years drinking this tablet, if there was no this thing, what do you think about my life and I came here feeling painfully or my body was on pain then maybe is about 8 years to 9 years now, what do you think about me on (not audible 27:14) maybe I'll be in grave long time ago but now I'm still alive, taking care of my kids, taking care of my wife, that's all.

I: Okay, since you have started getting HIV prevention services could you please explain how your life has been changed?

P: What?

I: Could you please tell me how your life has been changed since you have started…

P: This treatment?

I: No, getting HIV prevention services

P: It is too much, my life has changed

I: How, how has it changed?

P: I know that if you are using condom you can infect other people. I know that if you can just do it without condom like maybe I do it at home with my wife, we re-infect ourselves. I don't like that thing

I: Alright

P: Mmm (yes)

I: So, before…

P: Using this thing?

I: Before you were not using

P: I was not aware of that thing because I was not part of them, I was just here enjoying our lives, go up and down, do that thing of not using condoms then after that when I (not audible 28:45) I was infected, then I came here at the clinic they told me about this dangerous disease and I started to understand it and use right thing that they want. You must understand what are they talking about because if you cannot understand you cannot say (noise the background, not audible 29:09) those people don't want having a child or having a wife or what, they talking truth. Let's say you are doing this thing of sex, you must use condom, either you are positive, or you are not positive

I: Alright, can you kindly explain to me the HIV prevention services you think have been helpful to you?

P: Can I?

I: The HIV prevention services that you think have been helpful to you, can you please explain those ones?

P: I don't have many but what I think these things help our nation

I: Which HIV prevention services are they helpful to you?

P: Like what?

I: Types

P: Types of medicine?

I: huh-uh (no) prevention services that you are using that are so helpful to you

P: I don’t understand this one

I: Like you said condoms

P: Condoms are helpful to me

I: So, are the condoms only that you think are helpful to you?

P: No, with this medicine because you can use a condom without the medicine, you must drink the medicine always

I: Alright

P: Yah (yes)

I: Okay, now it is time for us to close this part of interview but before we do so, do you have anything in this topic that you feel it is important to say and we haven’t discussed it?

P: About?

I: About this topic that were talking about, about HIV?

P: No, nothing

I: Are you sure?

P: Nothing, what can I say? It is just that you must keep on doing this job because I think it is going to help many people, because other people don’t believe that there is a thing called HIV, and they don’t even believe they can be infected by this thing just because they are not infected but if they can be infected they wont believe that there is a thing, HIV is. Like us the time were still young boys we were still doing everything but when time comes then this thing is a trouble, it is going to kill us. That’s why you see us coming here always drinking this medicine. Other people passed away because they didn’t want to come here and make a queue, they don’t want other people to know that they are infected of which there is no problem because it is my life. What about, HIV is not the only one disease, there are many diseases that kill people but many people are shy to tell some other people that I am infected with HIV/AIDS because they hide themselves so that they can come and infect other people. It is not good that thing

I: Alright

P: Mmm (yes)

I: Okay, thank you very much. Now we have come to an end of our discussion and thank you very much for your participation. If you have any questions about your study participation just contact us. Thank you very much.

Time ended: 13:28
