# Supplementary material for: Understanding factors influencing utilization of HIV prevention and treatment services among patients and providers in a heterogeneous setting: A qualitative study from South Africa
Source: PLOS Glob Public Health. 2022 Feb 3;2(2):e0000132. doi: 10.1371/journal.pgph.0000132 (PMC10021737; doi:10.1371/journal.pgph.0000132)
Supplement: S1 Data — (ZIP) [file pgph.0000132.s001.zip › Supplementary information/IDI_Clinic attendee_QA008.pdf]

1 PARTICIPANT IDENTIFICATION NUMBER: QA008

2 RESEARCH ASSISTANT: XXX (Name of RA)

3 DATE : 17-07-2020

4 CLINIC NAME : XXX (Name of Clinic)

5 TYPE OF THE PARTICIPANT: FEMALE

6 LANGUAGE: ENGILISH

7 I. We are going to start with our discussing, can you please tell me more about yourself?

9 P. I am outgoing person, yes.

10 I. Okay.

11 P. I am outgoing person I like to go out, I like to have fun. I am freely and nice person I great everyone. Mmm yes I guess that it.

12 I. Where are you from?

13 P. Where I am from, XXX (Name of Province and Area)

14 I. I mean around here maybe?

15 P. XXX (Name of Area).

16 I. Are you married?

17 P. No

18 I. Do you have any kids?

19 P. Yes yes I do have one.

20 I. It's a girl or a boy?

21 P. It's a girl.

22 I. How old?

23 P. She is one.

24 I. Can you tell me how long have you being living in this area?

25 P. How long?

26 I. Yah (yes)

27 P. Since 2013 its was seven years.

28 I. Okay, How old are you if I may ask?

29 P. I am 20.

30 I. 20?

31 P. Yes.

32 I. How long have you being visiting this clinic?

33 P. How long?

34 I. Yes.

35 P. I started visiting this clinic when my mom medical Aid got expired, which was three month ago. Which it was three or four month ago I think then I can here yah (yes).

36 I. (Noise at the background) how was your first day at the clinic?

37 P. They were well coming, at first they well come me they teach me how things works. They saw me is not like going to the Doctor or going to the pharmacy and get your medication you have to relay and staff and staff but then it was like that.

38 I. Meaning you started your medication with the Doctor not the public?

39 P. Yes.

40 I. Okay, have you visited other clinics in this area?

41 P. No.

42 I. Why not?

43 P. This one is closer.

44 I. What do you like about this clinic and what do you dislike about this clinic?

45 P. What like is that anyone here is nice, the thing that I don't like is that I was waiting on the line for long time, and they didn't tell me or us to go in, so I guess is because of this pandemic that is going on then I guess that's way, but besides that they are nice people they great everyone, they are nice people Mmm (yes).

46 I. Could you tell me whether you are HIV infected if so how long?

47 P. Yes I am since 2018 its being two years now.

48 I. So can you please take me through the process since you are diagnosed with this HIV?

49 P. At first it was hard but I talk to my mom about it, I talked to my mother and she said is not the end of the world, you still have a lot of life a heard of you, yah (yes) I started doing those things that the Doctor was telling me do, he said I must exercise to keep healthy I must not eat everything for my body to be feet and healthier again and every time I go to the Doctor I make sure everything is fine even through it wasn't my intention to be infected but it happens that's how life is.

50 I. Are you on treatment?

51 P. Yes.

52 I. How long?

53 P. Yes its being two years now.

54 I. Can you tell me what are the major factor affecting your health right now?

55 P. Affecting my health I have got eye problems if you can see, my eyes gets red it was from my dad side I think, (Noise at the background) but I don't have any allergies or any symptoms.

56 I. By the time you started with HIV treatment did you encounter maybe any side effects?

57 P. No. it was okay. And it is still okay even now

58 I. Do you think this factor affect other people as you know as well?

59 P. Yes it does, it does affect them. It affect a lot of people.

60 I. How?

61 P. People are scared to go out, people are scared to get tested. Or prevent those kind of things you know, I think its affects them that fear is always there, I don't know what to say next.

62 I. So have you come across with someone who is afraid to get tested before?

63 P. Yes I have my friend, she is pregnant now she is pregnant as well, she like I don't wanna go to the clinic I am going to be tested and staff, and I said no if you get tested you get treatment you and the baby you are safe. Everything is going to be fine it's not the end of the world. I said I did the same I got pregnant and had HIV at the same time and here I am still standing and I am still strong.

64 I. Can you tell me your experience of service delivery from healthcare facilities?

65 P. My?

66 I. Experience.

67 P. Its good.

68 I. Maybe how if you can elaborate?

69 P. My experience (Noise at the background) I am running out of words. Ahhh (thinking)

70 I. You are well come to say anything you want to say.

71 P. Mmm (yes) it's being okay they are nice people like I sad, my experience was okay felt well come. And they didn't judge me yes I don't feel judged and I felt like I belong in the facility. I feel like I belonged here.

72 I. What are the most challenge features in this facility you have visited?

73 P. What's challenging?

74 I. Yah (yes)

75 P. The challenges?

76 I. Yes I had earlier saying that you stand a long time on the line?

77 P. Ohhh yes that was the first time and the second time they were quicker, today I don't know what's happening (Noise in the background)

78 I. Can you tell me about your experience getting HIV care?

79 P. My experience?

80 I. Yes.

81 P. I make sure I take my medication every night one hour before I sleep and I eat healthy and I don't go around telling people that you know I am HIV, Because people can judge you because of this virus, they can look at you and say ooh (yes) she is HIV. She likes things those kind of staff.

82 I. Have you being see anybody or anyone you know being judged about this virus?

83 P. *Nna (me*)I haven't, I only saw on face book they post they say what's happening what is the viral load. Why is the HIV rate is so high and staff and staff and staff. I am like no those things happen that's how life is.

84 I. So in your life since starting this treatment or being diagnosed have you being judged?

85 P. No,No. I never across those.

86 I. Meaning it's your mom and Dad?

87 P. My mother and mother and my sister, my sister always forces me to eat healthy because I like eating sweets, she judged me when I eat sweets she like no eat fruits and veggies and that's it, at first she was shocked but now she is fine, she accepted and we are okay now.

88 I. What are the things you would like to improve about health service in your facility?

89 P. I want them to be a bit quicker I guess since today I was waiting for long time in the line, for them to be a bit quicker and you understand that the are lot of us who suffer of chronic illness they should know at this day a lot of people are going to come, they should make their process be more faster I guess.

90 I. If you can tell me what can makes the nurse to be quicker?

91 P. To be?

92 I. The nurses to be quicker?

93 P. Firstly to stop going around, one thing I noticed about them is that before we get in they room around they talk they laugh, what if some is dying inside or someone how has the illness we don't know and they are in there they are busy rooming around, so that's the thing that I hate, so patient must come first and then you can do your taking and your things later.

94 I. Do you think you can raised this point to the nurses or whoever who is responsible for this clinic?

95 P. No, they just how they are, they like to talk.

96 I. Okay, how do you know or think about HIV prevention?

97 P. HIV prevention?

98 I. Yes.

99 P. Know a lot.

100 I. Can you please explain.

101 P. Before I tested positive they use to teach me at school, they say we must always use condoms, and stay away from boys which I have being doing, and go tested with your boyfriend before have sexual intercourse with the person so you know your status.

102 I. That's all you know?

103 P. Mmm (yes)

104 I. Can you tell me the different types of HIV prevention services?

105 P. I don't know.

106 I. Okay. What are the some difficult experience in accessing HIV services.

107 P. What are the?

108 I. Difficulties.

109 P. forgetting your medications, always forget so I set an arlam to remind me very night or my mom reminds. My mom reminds me and say take your medication. I think that's the most difficult part cos I always forget to take my medication.

110 I. So how long did you forget taking your medication?

111 P. How long did I forget?

112 I. Mmm (Yes)

113 P. A few nights and my mom will say did you take your medication, and I say ohhh I forgot and yah (yes) that's it.

114 I. Did you use condoms?

115 P. Yes I do.

116 I. Why did you use them?

117 P. To protect my significant other from getting the virus, even though my viral load is very low, even though I am not danger to society but is to protect my significant other so that they doesn't get infected, and whoever who comes to me I tell them I have HIV do you have HIV if he has we must use condom because it's important to use condom yes it's important to use condom.

118 I. How often do you use condom?

119 P. How often?

120 I. Yah (yes)

121 P. I only use it once and it blasted I don't know how?

122 I. Where did you get it from?

123 P. The condom?

124 I. Yes.

125 P. I was going to XXX (Name of Area) to school they gave us condoms when we geos out, so I just took them and put them in my bag. When me and my boyfriend says lets to this and this, he even told me about his status I didn't ask him about his status I don't ask him that I am like okay here is the condom and them we use the condom, I didn't even check if the condom is okay.

126 I. Earlier you said that condom blast?

127 P. Yes.

128 I. What did you do after?

129 P. What did I do?

130 I. Yes.

131 P. I stopped having sex, I never had sex I just told myself that I will stay away from boys, I will stay away from doing that staff and focus on my future and focus on my child.

132 I. Can you explain what are the process and universal Test and Treat is?

133 P. The?

134 I. Universal Test and Treat is? Or can you please take me to the process when you tested HIV positive what did the nurses do or the Doctor do.

135 P. I cryied I was shocked, I cryied because I always use condom and I was pregnant at the same time and I said how did this happen.

136 I. So did you start your medication as soon as you find out you are HIV positive?

137 P. Yes.

138 I. What are some of advantage of UTT and what are the some of disadvantages?

139 P. What is UTT?

140 I. Universal Test and Treat?

141 P. The advantages is you get to know your status and the disadvantages is that when people when they walk around and they know you they will be like ohh are you the one that tested positive infant of everyone this thing is rude. Some people are very sensitive like me I am very sensitive person.

142 I. Has the any changes to health service in information since immediate Art?

143 P. No, no changes.

144 I. The is no changes?

145 P. Yes.

146 I. What if any issues you have experience that may prevent or stop you from accessing ARVS.

147 P. What can prevent me?

148 I. What can prevent you from taking ARVS?

148 P. What can prevent me?

149 I. Yes.

150 P. Getting healed, something that can take this virus from my body so that I can be fine and feel that confident again.

151 I. So what can prevent you from accessing or taking HIV treatment?

152 P. HIV treatment?

153 I. Yah (Yes).

154 P. I don't understand.

155 I. What can prevent you from taking the treatment?

156 P. What can prevent me from taking the treatment?

157 I. Yes.

158 P. Nothing.

159 I. So you can take your medication anytime anywhere?

160 P. I make sure I take my medication at home.

161 I. Why at home?

162 P. It's safer there.

163 I. Why if you may explain?

164 P. Because when I go to visit, like when I go to visit my cousins my sibling and my aunt. I just make sure I come back at home to take my medication or I hide the medication and go to the bath room and take my medication and then go out. Because I don't what them to know that I have a virus in my body.

165 I. Why do you prevent them to know that you have HIV?

166 P. You know when you live people can talk about you so you can never trust anyone, so I only trust my family there are the ones who are closer to me, they are the one who reminds me to take my medication. There are the ones who motivate me to keep going.

167 I. What do you think if one continue or stop taking their medication?

168 P. Stop taking the medication?

169 I. Yes.

170 P. With that I don't know, but I guess your viral load will go high or go to second phase of HIV which is AIDS. Yah (yes) and then you die.

171 I. Then for the one who continue to take ART?

172 P. The one you stay healthy, and nothing happened actually, nothing happens if you make sure you take medication and if you make sure every day this time I am taking medication, if you make sure you eat healthy and your mind is always on point, but mine is not on point sometimes. But if you make sure yours is always on point so that you don't forget to take your medication.

173 I. Since accessing the facility of HIV prevention service, could you explain how your life has being impacted?

174 P. It has change a lot, I couldn't go to school because I was embarrassed I finish my matric in

2018 that when I got into the virus. I finish my matric I got pregnant and I have to stay at home and look after a child because she was young, yah (yes) I think that's the worst I ever being. But I have heard people around who are helping me like my sister who was always there when I needed her.

175 I. I heard that you have a child, she is negative or positive?

176P. She is negative.

177 I. Okay.

178 P. Mmm (yes)

179 I. Could you explain the HIV prevention service you think has being helpful to you?

180 P. Helpful to me?

181 I. Yes.

182 P. Taking Arvs like when I go to the Doctor I will always get vitamin c , here they gave me vitamin b3. I don't know to prevent TB I don't know what for, but vitamin c to boost my immune system for me to keep going but even b3 its okay. I can feel that I am strong now and nothing has happen to me.

183 I. So you are saying the multivitamin you are getting here it's not the same like the one you use to get from the Doctor?

184 P. No. I was getting vitamin c at the Doctor but when I got here I they change (Noise in the background) they explain that vitamin b3 is for your immune system and staff, then the other tablets they explain is for preventing TB because since your immune system is very weak I have to take them for a full year so I have to finish the course so it's being three month now since I have being taking the b3 and TB prevention.

185 I. So we have come to the end of our section if the is anything that you want to add you can add before we close.

186 P. People should get tested and never be ashamed this is life it happen to everyone, you must get out. Life goes on time wait for no one, because the whites will say time waits for no man. You must get up move on, if you are positive get your treatment you will be fine.

187 I. That’s all you want to say?

188 P. Mmm (yes)

189 I. Thank you very much for your time to participate in our study, time is 13:30. Thank you very much.

190 P. Okay.
