# Supplementary material for: Understanding factors influencing utilization of HIV prevention and treatment services among patients and providers in a heterogeneous setting: A qualitative study from South Africa
Source: PLOS Glob Public Health. 2022 Feb 3;2(2):e0000132. doi: 10.1371/journal.pgph.0000132 (PMC10021737; doi:10.1371/journal.pgph.0000132)
Supplement: S1 Data — (ZIP) [file pgph.0000132.s001.zip › Supplementary information/IDI_Clinic attendee_QA011.pdf]

Full participant ID: QA011

Participant Type: Female

Location: XXX (Name of Clinic)

Date: 2020-07-20

Primary interview language: English

(Recorder did not record for 20 minutes)

P: [Background noise] there is no…. for now, I can say nothing that is affecting me for now.

I: for now?

P: Yaa (yes) it's just all these general sicknesses like flu like other people otherwise none the less I don't have.

I: Okay, sicknesses like flu since is the flu session.

P: Yaa (yes), yes.

I: Ooh okay, that's normal, do you think these factors affect other people that you know as well?

P: Yaa (yes)... yes.

I: Aah can you please elaborate on that?

P: Mmh(yes) Like my (laughing) my aunty… (interrupted by interviewer)

I: Aah(yes).

P: They have flu too, my cousins they have flu at times meaning like the one that you are asking me is the one that uhm which one the major factors?

I: Eeh is that one for just eeh factors affecting other people that you know as well?

P: Yaa (yes).

I: So, they also suffer like from sicknesses [background noise] such as flu?

P: Yes.

I: Ooh okay, so now we are moving on to our next part which is experiences with public healthcare facility facilities. [background noise and paper flipping].

P: Okay.

I: Now we are going to talk about health care in general.

P: Okay.

I: So, can you tell me your experiences in terms of service delivery from health care facilities?

P: Like facilities meaning this clinic or?

I: The clinics around all over in general, clinics in general.

P: Okay, some uhm as for me this one that I'm attending uhm they deliver but problem is it shortage of nurses sometimes.

I: Mmh (yes).

P: But others other clinic I don't know about them, whether they deliver or what because but news that I watch people are complaining a lot about health facilities in other places.

I: Yeah (yes).

P: You know, so generally uhm we are facing a lot of shortate shortage of staff and some other medication, it's hard to get other medication because others they are distance from those facilities. If they can bring clinics generally like near the people sometimes other people, they have to use taxis and they don't have money to…

I: Yes.

P: to go to clinic so that's the problem.

I: Its understandable mam, so what are some of the positive features in the facilities that you have visited?

P: The positives?

I: Yes.

P: Okay, what I can say is that uhm in this place I found that uhm they can be nurses and then at the same time they can be a family sometimes you learn to share things with them you know… (interrupted by interviewer)

I: You learn what?

P: You learn to share things with them, and they make you feel at home, you know then you go to clinic its not like they will use the stigma that you have to label you but you just feel happy when you come here. So that’s the only thing I can say about it I just found family instead of aah nurses. Just because I call them nurses, but I found friends too.

I: Yes, yes and is easy to open about…. (interrupted by participant)

P: Anything that I have… (interrupted by interviewer)

I: Yes yes.

P: Any problem that I have, any secret that I have I can open up to them.

I: Yes.

P: And I can even call them or talk to them you know generally like friends.

I: Yes.

P: Yaa (yes) that’s.

I: That’s good and what are other most challenging features in the facilities that you have visited? Most challenging eeh features.

P: Mmh(yes), most challenging is to wake up in the morning…

I: Yes.

P: And then standing in the queue like you wake up round about five o’clock to come to the clinic so that they will attend to you and the clinic open by eight o’clock, so can you imagine you wake up by five just to be inside.

I: Mhm Mhm mhm (yes yes yes).

P: Yaa(yes) that’s most challenging for me.

I: Yaa yaa yaa (yes yes yes), can you tell me about your experience getting HIV care?

P: (sigh) My experience?

I: Like how is it? Uhm…

P: For the first time?

I: Yes.

P: For the first time it was difficult for me, it was difficult eeh but when times goes by it wasn’t really difficult so but later-on I I have get used to…

I: To it?

P: To it.

I: Okay, I I hear you say it was difficult at first, how how difficult was it? Can you share some instances whereby you find difficulties?

P: Uhm, you know when you come to the clinic you think this is the queue for HIV pills, basically it’s not its everyone’s you know.

I: Mhm.

P: You look people maybe they are looking me, you are scared…

I: Mmh.

P: You even feel like not coming to the clinic anymore.

I: Okay.

P: So, it was challenging for me for the first time, but I had to learn in a harder way that I have to come to clinic at the end of the day.

I: At the end of the day?

P: But now I’m really enjoying it.

I: So, what you are basically are saying is that it is like they are catero… categorizing you? HIV people this side.

P: You know sometimes they will say uhm critical I don't know condition or whatever.

I: Ahaa (yes).

P: So, you will see that okay this people they are for Sugar Diabetes, now HIV whatever, you understand? So that's was the problem for me [background noise].

I: [Background noise continues] Ooh okay, okay okay. So, did your things get better with time?

P: Yaa (yes) when times goes by things get better.

I: Ooh okay.

P: Because everybody they attended the same way, we are just the same in a same way.

I: Ooh okay, okay. So, what are the things you would like to improve about health Services in this facility?

P: Uhm, to improve?

I: Yes.

P: Uhm, to have more staff and like to expand the place because the place is too small.

I: Yaa, yaa (yes yes).

P: The clinic is very small it's what I can say but…

I: Yaa (yes).

P: And it have a lot of people and there's lot of people who are coming from different places.

I: Yeah (yes).

P: So that's the problem.

I: That's the problem.

P: Yes.

I: Ooh okay, now we are coming to the part which ask eeh about knowledge of HIV prevention, so what do you understand about HIV prevention?

P: Mmhm (thinking), what do I understand about the HIV prevention?

I: Yes.

P: Uhm, basically you saying if I was not HIV how do I prevent myself?

I: EYaa (yes), along those lines.

P: Okay, so what do I understand is that either you condomise or you abstain?

I: Condomise or abstain?

P: Or abstain.

I: Yes.

P: That's the preventions.

I: That's the preventions.

P: That's I know.

I: That you know, not any other… no any other prevention?

P: Aaah (yes).

I: Condoms only?

P: Yes.

I: Okay, can you tell me the different types of HIV prevention services?

P: Okay, different types of prevention services?

I: Yes.

P: HIV prevention uhm is what I can say the first the first uhm answer that I told you is that you prevent, you condomise you use a condom.

I: Condom use.

P: Yes, and then either you abstain.

I: Abstain yes.

P: Yes, you can abstain from from it.

I: Yes.

P: And then uhm what I have nuna is just that those pills I don’t know them, I don’t know after that you drink, I don’t know what are those I forgot them.

I: Ooh okay, the pills you drink after?

P: They said that you drink them after sex ((cell phone ringing)) is just that the general information but I don’t know is it real or…

I: Okay okay.

P: I just forgot.

I: [background noise car passing by] I understand there is “PREP’’ and the is “PEP’’.

P: Yaa yaa (yes yes).

I: [background noise continues] okay as we go on there is no anything?

P: Yaa (yes).

I: Okay try to focus uuh, what are some of the difficuls you may experience in accessing HIV prevention services? What are the difficulties maybe that you can come across, say maybe you’re coming for condom collection or any other prevention services that you may come for?

P: Eeh if you are coming to the clinic as a lady you want to protect yourself from uhm HIV and coming just like you want to come and collect condoms its very hard.

I: It’s very hard?

P: Its very hard for a lady to just come and pick condoms.

I: How how hard is it? Please share that.

P: (laughing) aah on my general knowledge you think that people they are just looking you…

I: Mhm (yes).

P: And they can't mind their business and they are asking condoms it could didn't be a problem they will give you the condom at the end of the day but there will be questions like "why do you need the condoms?'' so it's very hard…

I: Yaa yaa (yes yes).

P: To go and access them so it's better you go and buy them in a shop and too if even you go to the shop like the person sales person will be looking you like this lady is buying condoms.

I: Mhm, mhm.

P: So that's the problem.

I: Yaa (yes), so if a lady is like when when a lady comes for condom collection it's like eeh they they they like to engage in more sex… (interrupted by participant)

P: Yes.

I: Safe sex all the time.

P: it's what that's the stigma.

I: Ooh! Okay.

P: Yaa (yes).

I: Okay, so that may prevent you from practising condoms hey?

P: Yes.

I: Okay okay okay, so our next question is based on condoms again.

P: Okay.

I: So, do you use condoms?

P: Yes.

I: Why do you use them?

P: I use them just because of my… like eeh my since I'm HIV and then and my partner too since he is HIV… (interrupted by interviewer)

I: Yes.

P: So, we are using basically condoms so that our viral load and our CD4 count could be eeh…

I: Supressed.

P: Supressed.

I: Okay.

P: So that's it.

I: Okay alright alright alright, and then how often do you use them?

P: I can say is every day of my life.

I: Every day of your life?

P: Yes, it's a lifetime thing that I have that I'm facing right now.

I: That's good, where do you get them from?

P: I buy them in Clicks.

I: At Clicks?

P: Mhm (yes).

I: Ooh you buy them?

P: Yes.

I: So, what other places can you get them from?

P: I can get them from the clinic.

I: Yes.

P: But I I can’t go to clinic, I better buy them.

I: Since you said “you feel like eeh people maybe are looking at you” …(interrupted by participant)

P: Yes.

I: In a different way when a lady comes and collect condoms.

P: Yes, so when I’m buying, I’m just buying and that’s my choice and the person won’t ask me questions.

I: Yes.

P: “Why are you buying the condoms?’’ for the what I’m just buying them and take them home.

I: Yes, yes yes, so what could prevent you from using condoms?

P: Nothing.

I: nothing? Okay, what could prevent you from getting condoms if you think?

P: Basically nothing, nothing I can come to clinic I will get them.

I: Yes.

P: Nothing will prevent me from getting them…

I: Okay.

P: Unless I forget about the people and their peer pressure outside them whatever…

I: Yes.

P: And… (interrupted by interviewer)

I: Yes.

P: So, I will get them.

I: You will get them, you will always get them.

P: Yes.

I: Okay, can you explain what the universal test and treat is?

P: I don’t understand that.

I: You don’t understand that?

P: Mhm (yes).

I: Okay.

P: What is universal test, universal test is what?

I: Okay, if I may explain it to you eeh briefly its when you come for an HIV test, you test now you get your result, you go home having your medication at the same time.

P: Okay.

I: Yes, so do you understand anything about that?

P: I understand.

I: Basically, it’s what we call immediate ART, ART immediate ART.

P: It’s okay.

I: Okay?

P: I I don’t know about that?

I: You don’t know about that?

P: But I have faced that challenge before.

I: You faced that… (interrupted by participant)

P: Because I’m I came and test uhm when I was pregnant, I tested for the eeh first time.

I: Yes.

P: And then I found that I’m HIV, at the same time uhm okay I was given the medication… (interrupted by interviewer)

I: Medication okay.

P: So, that I will start the eeh treatment as soon as possible.

I: Yes.

P: You know? And then I started the treatment as soon as possible but eish it was very hard.

I: It’s very hard.

P: It was very hard.

I: Okay, okay how hard was it? Why do you say it was hard?

P: To accept again.

I: Okay.

P: To accept uhm that ooh now I’m HIV, ooh what? I have to drink this medication you be like holding the medication and looking the medication, looking at yourself and looking back.

I: Yaa, yaa (yes yes).

P: So…

I: It was…

P: It was difficult to just accept that.

I: Okay, okay so the next question would be what are some of the advantages if you think of that to test and treat? You see it says UTT? UTT stands for Universal test and treat.

P: Yea (yes) test and treat.

I: Yes, so what do you think are some of the advantages of it?

P: The advantages of it is that if you start the treatment immediately…

I: Yes.

P: It’s gonna be fine because it helping your health and everything.

I: Yes.

P: You know? So, that’s the advantage of it.

I: Okay, okay.

P: You know, if they test you now and then you find out that you are HIV not like eeh come back next month and then we gonna start no, immediately you find out that you are HIV start using the medication immediately too.

I: Immediately.

P: Yes.

I: That’s nice, and what do you think are some of the disadvantages?

P: The disadvantages is that eeh for the first time the for the first time if you never drink the medication it has side effects.

I: Yes, yes.

P: First of all…

I: Mhm (yes).

P: You may find it difficult, if you drink it for the first time it might made you like others, they are vomiting…

I: Mhm mhm (yes yes).

P: Having bad dream…

I: Mhm.

P: sweating the whole night, it will be like you are sick already, you know?

I: Mhm, mhm (yes yes).

P: So, that’s the disadvantage about it.

I: Yaa yaa yaa (yes yes yes), any other disadvantage you may think of?

P: Mmm (thinking), aye(no) besides those ones I don’t know.

I: Okay, okay nice [background noise] has there been any changes to the way health information or health services have been delivered, since immediate ART, UTT do you remember?

P: Mhm (yes).

I: Began that had changed the way you look after your own health?

P: There have been changes, you know like uhm you have to like medication before there were a lot now it’s one.

I: Yes, yes.

P: Now its one and uhm so now the only thing is that they have never changed the the rules of how to take out, you continue that thing.

I: Mhm (yes).

P: Yourself which is you have to take care of yourself and then you listen to your to the person who is attending to you.

I: Yes.

P: You, do you understand?

I: Yes.

P: And then you listen carefully and drink that your medication I think that’s it.

I: Yaa yaa okay so, (clears throat) what if any issues have you experienced that prevent you from accessing or taking ARVs? Do you have any issues that you have experienced that may prevent you from taking or accessing eeh ARVs?

P: No.

I: Okay.

P: No I have…. (interrupted by interviewer)

I: So, so it’s been easy?

P: It’s been easy.

I: Accessing them?

P: Yes, its just come to the clinic [backkground noise] they give you the pills [background noise continue] then your {not audible 00:17:44}.

I: And how about taking them?

P: Taking them its been easy too but at the same time, you know time because eeh you tell yourself that you gonna drink them by nine o’clock and you finds that’s that’s maybe you are in a party or family gathering…

I: Mhm (yes).

P: And then its nine o’clock…

I: Yes.

P: So, you have defaulted the time…

I: Yeah, yaa (yes yes).

P: Because when I get back even its eleven o’clock, I have to drink them.

I: Yes.

P: So, that’s the pro…the…

I: The the an issue.

P: Yes.

I: The small issue.

P: Yes.

I: Okay, so what do you think would happen if one continues to take ART?

P: Its aah ART it’s like ARVs?

I: ARVs yes ARVs.

P: Okay.

I: So, what do you think will happen if you if you continue say maybe you yourself you continue taking ARVs? What do you think is gonna happen to you?

P: What gonna happen to me, I will live long.

I: You will live long.

P: Yes.

I: Yes.

P: I will be healthier, and no diseases no diseases can come upon me because I think this thing suppressing any sickness.

I: Yaa (yes).

P: That will come my way.

I: And the immune system is…

P: Is is getting stronger each and every day.

I: Yes, yes okay, so how about you stopped taking the medication ARVs?

P: Iyo!(surprised) I will be sick, and my my CD 4 count will be low and I will start having many challenges and sicknesses.

I: Mhm (yes).

P: From different side.

I: Yaa (yes).

I: So, I don't think I will stop taking them.

I: Yes, okay we have come to bevi…behavioural change and the question is since accessing the facilities for HIV prevention services, could you explain how your life has been impacted?

P: Since I have been coming like to clinic and taking the medication…(interrupted by interviewer)

I: Yes yes.

P: Eeh since I have been coming to a clinic and taking the medication its been its been (laugh’s) I can say its eeh its like a hobby now.

I: It’s a…yaa.

P: Because I know that every month or after two months I have to go to clinic.

I: Yes.

P: So, now its that’s my lifestyle now so I can take it like my hobby of coming to clinic and going now.

I: That’s nice, can you also explain the HIV prevention services you think have been helpful to you?

P: Yes, HIV prevention services have been helpful to me like condom as well as I said… (interrupted by interviewer)

I: Yes, yes.

P: It suppress down.

I: Yes.

P: The viral load you know?

I: Yaa (yes) the ART.

P: So, yes so and I think none the less that’s it.

I: Yaa okay, yaa now we have come to the end of our session, its time for us to close this part of the interview, but before we do, is there anything else that (clears throat) is there anything about this topic that we haven’t discussed that you feel is important to say?

P: No.

I: Is there any way there way you like to add something?

P: No, what I can say is that eeh people should get tested always and they have to know their status each and every time, so that we will fight this thing together you know? If I can if we can find the vaccine I will be happy.

I: Yes.

P: That’s it.

I: That’s that’s a strong message.

P: I will be just happy.

I: Okay okay, aah anything else? That’s it.

P: That’s it thank you.

I: Now we have come to the end of our discussion, thank you for your participation, if you have any questions about your study participation please contact us. Thank you.

P: Thank you.

I: And the time now… (paper flipping)

P: Half pass ten…half pass

I: 10h35, thank you again.

P: Yes.

GLOSSARY

HIV – Human Immunodeficiency Virus

ART- Antiretroviral Therapy

UTT- Universal Test and Treat

PREP- Pre-Exposure Prophylaxis

PEP- Post Exposure Prophylaxis
