# Supplementary material for: Understanding factors influencing utilization of HIV prevention and treatment services among patients and providers in a heterogeneous setting: A qualitative study from South Africa
Source: PLOS Glob Public Health. 2022 Feb 3;2(2):e0000132. doi: 10.1371/journal.pgph.0000132 (PMC10021737; doi:10.1371/journal.pgph.0000132)
Supplement: S1 Data — (ZIP) [file pgph.0000132.s001.zip › Supplementary information/IDI_Clinic attendee_QA012.pdf]

PARTICIPANT IDENTIFICATION NUMBER: QA012

RESEARCH ASSISTANT: XXX (Name of RA)

DATE: 2020-07-20

Facility NAME: XXX (Name of Clinic)

LANGUAGE: ENGLISH

I: My name is (XXX Name of an interviewer) and the time is 11 o'clock I am conducting an interview at (XXX Name of a clinic). I am... before we continue, and do you have any question?

P: Mmm (No)

I: Alright eh whatever that we will be discussing here we will remain confidential. We will be using English and whatever that you will be saying could you please raise your voice so that we can be able to record everything that you're saying.

P: Yes Sir

I: Yes, Could you please tell me more about yourself?

P: About myself?

I: yes, how old are you?

P: I am 44

I: Alright where you from?

P: originally?

I: yes

p: originally?

I: Yes yes

p: I am from (XXX Name of a Province).

I: Alright and then currency where do you stay?

P: Currently I stay here (XXX Name of a place)

I: Alright around (XXX Name of a place)

P: Yeah around (XXX Name of a place)

I: Alright, are you married?

P: yeah, customer.

I: And do you have any children

P: One

i: Alright how old is your child ?

P: Ehh he is turning 12.

I: Aright, How long have you been living in this area?

P: Its been since from 2016, four years

i: Four years

P: Mmm (Yes)

I: Alright, And then how long has he been visiting this Clinic?

P: I started last year.

I: Oh last year...mid last year?

P: I started some time last year... let me check first

I: Alright.

P: [opening a hand bag] i just want to be sure.

I: Have you visited...

P: I was transferred here from last year January.

I: Alright. From which clinic were you from before?

P: I was from, actually i was not attending at the clinic i was attending at the hospital.

I: Alright.

P: So they transferred me here.

I: Have you ever attended any other clinics except here?

P: Other clinic?

I: Yes

P: Yeah

I: Which one was it?

P: (XXX Name of a clinic)

I: (XXX Name of a place)

P: Mmm (yes)

I: Okay, when as it?

P: Mmm (thinking) my las visit was ... eish i dont know, i dont remember.

I: Alright, What do you like about this clinic?

P: the service is good.

I: Yeah...When you talking about services which one are you talking about?

P: Like you don't you don't you don't... When they started opening ok, to start to work 8 o'clock.

I: Yes

P: You don’t stay for long.

I: Alright, They attend you imminently

P: They attend you imminently and they are fast.

I: Alright, what do you dislike about this clinic?

P: What i dislike about it, its the fact that, how can i put it... even before COVID (COVI19)

I: Yes

P: We queue for long outside.

I: Alright

P: My concern is when it rains, where are we going to stand outside there and when its really hot out there, like extremely hot.

I: Yeah

P: You cant stand outside there, and there is no toilet outside.

I: And inside the facility?

P: They are only inside and you must wait until they open.

I: Alright,

P: and sometimes I come here like today at 6:00 if i am not mistaken 5:30.

I: 5:30, Except the queues is there any other issue or any other things that you dislike about the clinic?

P: Ah for me... for me its ok.

I: can you tell me about your HIV status? Are you are you testing in this clinic? Do you know your HIV status?

P: Yes, I am HIV positive.

I: Alright.

P: I have been HIV positive since 2008.

I: Since 2008, So do you attend here to take treatment? Are you taking any treatment?

P: Yeah i am taking treatment.

I: For how long?

P: I have started taking treatment since from 2013.

I: Alright, So how are the services here? how do you access them?

P: Here its easy, that’s why i am saying i started at the hospital because I was sick.

I: Alright

P: And I was attending clinic at the hospital and then they referred me here because of my Viral Load.

I: Alright

p:... has picked up

I: What are the other factors that affect in your health right now?

P: nothing

I: Alright

P: The health that is giving me problem is my high blood.

I: [laughing] Your high-blood did you get any treatment for your high blood?

P: Yes i did, they gave me something to treat the high blood.

I: Ok, Do you think this factors affect other people as well? The high blood ?

P: Yeah i think so, i think so because of, i think the medication that we taking, at first i didn’t have high blood. But i think because of the medication that i am taking.

I: Yeah

P: I think the medication is the one that is causing...

I: High blood?

P: High blood yes.

I: Alright, i hear you. So you spoke about your partner. Having a partner

P: Yes

I: Is he also attending here?

P: He is negative.

I: oh alright, is he supportive?

P: Yes

I: Oh, Good, Can you tell me about your experience in terms of service delivery in this facility?

P: when you mean service delivery now so?

I: I am talking about medication, Availability of medication.

P: Oh I didn't have any with my medication I always get my medication on time.

I: On time, alright.

P: I have never come across of them telling me that they dont have medication.

I: Aright, Any challenges that you faced before you come here for visiting? Except the queues

P: Mmm (No)

I: No challenges?

P: Yes

I: Alright, what would you improve about this facility?

P: What?

I: If You had powers to improve this facility. What would you improve?

P: [laughing] i would make shelter for the people outside, that are standing outside.

I: Alright, i hear you.

P: And maybe the mobile toilets

I: The mobile toilets

P: Yes, Outside because unless if someone can come early and open early

I: Yeah

P: So that we can access the toilets

I: Yes

P: And the shelter is more important, summer is coming now.

I: Yes

P: For those who don’t have cars, when it rains, yes.

I: Ok, What do you understand about HIV prevention?

P: Eish i understand a lot yoh,

I: Take me through like someone who doesn’t know about it.

P: [laughing], eh HIV Prevention neh (right)?...

I: Mmm (yes)

P: Always use a condom.

I: Alright

P: and you must know your status always.

I: Yes

P: Don’t look at me and and think I’m negative or... without knowing my status. So knowing your status is very important.

I: And then, what are the other preventions that you know of? it's only condom

P: Its Condom and abstain.

I: Alright, abstain is number one right?

P: [Laughing]

I: All right How accessible are the condoms?

P: Yeah they are accessible, they... usually when you go to the rooms here

I: Yeah

P: To... what do they call it?

I: Eh which rooms are you referring to?

P: Inside here.

I: The ones

P: They do have condoms

I: Alright, alright. So how often do you see people taking them?

P: I don't know because you go one person at the time.

I: One person at the time?

P: Inside there

I: Alright. Can you tell me about the way how how how user-friendly are the place where they put the condom? is it confidential enough for you to access them or you feel like

You just want to be alone when you access them?

P: I just want to be alone when accesss them.

I: Alright, well if you don't mind me asking and do you use a condom?

P: Yes I do, as i have said my partner is...

I: Negative

P: Yes negative

I: The reasons why you are using a condom. Can you give me the reasons why you are using a condom?

P: I don't want to infect him

I: Alright, And then are you able to use it in a good way?

P: Yes we do.

I: In a good way? Any challenges?

P: Mmm (no)

I: Alright, good. Eh what do you understand universal Test and Treat? Eh you don't know anything about it?

P: No.

I: UTT?

P: I am clueless about that.

I: Alright its about a process where by you test a person today and you initiate the person today. You give the person today the ART. You give the person same day treatment.

P: Same day?

I: Yes, Without waiting for the viral-load.

P: But i have never done that.

I: alright, take me through your process. How did they initiate you?

P: I was pregnant, then I... thats when tested.

I: And then you started...

P: If i can remember correctly, usually they use to test us when you are pregnant.

I: Yes

P: And then the results came positive and then i told my partner and then he went went and test, they were negative. After three months he tested again negative and mine was positive.

I: Mmm (ok)

P: S o i started to take medication 2013, and i found out that i am positive 2008.

I: Alright

P: I think my viral load just went down, dropped and my CD4 count was low

I: mmm

P: That when i took my.... i started the ARVS

I: Alright.

P: And then now my viral load is undetectable.

I: Ok, Eh when a viral load is undetectable, what does it mean?

P: It means that the virus is there but its suppressed

I: Oh suppressed, ok alright. Thank you for the clarity, eh do you have any challenges that you are experiencing when you are taking ARVs?

P: I have never had issues, me i have never had issues but before you take the ARVs they tell you how are they go like. You will have night dreams and bad dreams, sweat and what what. I have never experienced that.

I: Alright, Eh what happens if someone stop taking their treatment? [silent] you dont know about it?

P: I do but...

I: you dont know how to explain it?

P: I dont know how to explain it. That person will have AIDS, HIV and AIDS.

I: Oh, whats the different between HIV and AIDS?

P: AIDS is different from HIV but is caused by

I: HIV?

P: Its a one disease, immediately when they say you are fully blown.

I: Alright now i get it

P: Thats when you dont take your medication, thats why your medication is very important.

I: alright

P: And you taking them same time evryday, every night before i sleep.

I: Mmm ok

P: So i dont , i dont make that mistake of not taking my medication, even my child know that i must take my medication.

I: Mmm (alright), So if someone eh each the HIV and AIDS level is there any other solution? What do you think should be done? Do you have... are they offering any services in this clinic when you are HIV and AIDS positive?

P: Which clinic ? This one

I: Yeah this clinic yes

P: They counsel you first...

I: Oh they counsel you?

P: Yeah you go through counselling

I: Alright,And then they initiate you again? And they they give you treatment?

P: Yes

I: Ok, alright , alright. Could you explain how your life have been impacted? How did it change or how ?

P: Mine didnt change, i am still living a normal life.

I: Alright, ok. Eh do you think the HIV prevention services is helpful to you?

P: To me yes.

I: And the youth out there are they aware of this services?

P: They are aware but most of them they are afraid

I: Oh they are afraid.

P: Mmm (yes)

I: whats there to be afraid of? Is it the clinics or the communities out there?

P: The community

I: Alright, whats ...

P: Of what people would say

I: Alright, How did you overcome that situation on yourside?

P: Ah, My family was therefore

I: Your family

P: Their support

I: They know about your status?

P: Actually when i am saying my family i am saying my partner.

I: Ok

P: And my child

I: Alright, they are supportive

P: Yes

I: Do you have any question so far?

P: No

I: Alright

P: The only question that i am having is when are we getting ah treat... not like permanent treatment.

I: oh for HIV?

P: Yeah

I: Eh

P: That will be the question that i am having.

I: We don’t know so far [laughing] so fa they are still busy but i will escalate the question to my superiors. As soon as i know the answer you will be the first one to know. But then so far, the ARVs are doing wonders right?

P: A great job.

I: oh yes, that is good that’s good. So is there any other supportive eh group or counselling that you receive here in the clinic?

P: here?

I: yes.

P: ai me on my side mhm mhm. [thinking]

I: But is there for.

P: (cuts in) … I don’t know.

I: other people. You don’t know about it?

P: mhm (no) remember I told you at the first time I said I came here last year?

I: yes. So.

P: I was transferred from hospital, at hospital they use.. There were groups, supporting groups.

I: yes yes yes.

P: and then when they saw that my viral load is up and then that’s when they discharged me from the hospital to come here to the clinic.

I: Alright. So eh I didn’t to tell you about the UTT. Jah the UTT is when you test someone today and then you treat the person today. Reason being is that, eh before

people used to be tested today and then they have to wait for a certain period of time for them to... I mean for their viral load.

P: to go down.

I: yeah to go down. So now they initiate the person immediately, yes do you… there's a different between the previous system and this one.

P: this one, okay.

I: Yeah. Do you see any advantages from that one?

P: yeah I think it's good.

I: In which way?

P: in a way that if your viral load is down you get sick and then.

I: and then accessing them. Do you feel like accessing.. clinics accessing accessing people out there is simple. Like initiating a person today and then tomorrow. Are they normal… do they normally come here to collect their treatment, or they have to make follow ups on them always?

P: when they initiate?

I: yes.

P: I don't know.

I: okay.

P: That one I don't know.

I: Alright.

P: but me I collect mine.

I: you collect your?

P: Yeah they give me a date and then I come back, give me a date.

I: Alright. So we are about to come to an end of our interview. Do you any question or any other things that you would like to say?

P: mhmm (thinking).

I: anything.

P: what I would like to say is if it's possible neh (right), when like you don't withdrew blood neh it is fine to send your partner to collect and you go to work. You don't miss time to go to work time and again and time and again.

I: eehh withdrawing blood?

P: if am not…like today I was withdrawing blood, it's fine I came myself. So it is fine if my partner will come September to.. For my results and medication.

I: yes you can be able to have someone who can come here to collect your medication for you but am not sure about the results of your blood.

P: okay.

I: you need confirm with your nurse, your doctor.

P: no the results of my blood I can come and take them am saying for.

I: treatment?

P: treatment.

I: yes.

P: because you see when you miss work time and again time and again, sometimes it's.

I: yeah so When you talking about work, is there any other things that come between work and your health?

P: my health comes first, that's why.

I: so eehh.

P: last week I was here Tuesday they were closed and I came again, when, Wednesday.

I: are they giving you any letter or something that shows. That prove that you came to the clinic today, I mean a letter to show your manager at work.

P: Yeah, if you ask a letter they give you.

I: Alright, at the clinic?

P: hmm.

I: Alright. Free of charge?

P: hmm.

I: Alright, okay. Thank you. I think we covered all the important points that we needed from this interview. Thank you very much, thank you for your time.

P: okay thanks

I: there time is 11:23
