# Supplementary material for: Understanding factors influencing utilization of HIV prevention and treatment services among patients and providers in a heterogeneous setting: A qualitative study from South Africa
Source: PLOS Glob Public Health. 2022 Feb 3;2(2):e0000132. doi: 10.1371/journal.pgph.0000132 (PMC10021737; doi:10.1371/journal.pgph.0000132)
Supplement: S1 Data — (ZIP) [file pgph.0000132.s001.zip › Supplementary information/IDI_Clinic attendee_QA013.pdf]

TYPE OT INTERVIEW: QUALITATIVE

DATE: 20 JULY 2020

INTERVIEWEE: CLINIC ATTENDEE

INTERVEIWER: XXX (Name of RA)

PI: Okay participant number QA013 participant male, XXXX Clinic (Name of Clinic), interview date ehh 20 July 2020, interviewer XXXX (Name of RA). Let`s start.... Mmm... Okay. Thank you for agreeing to be part of this interview. Thank you for your time, ehh we going to be starting the interview. Ehh as I`ve said my name is XXX (Name of RA), thank you for agreeing to be part of the interview. The purpose for this regulation is for to allow us to, do you agree to allow us to record this session?

QA013: Yes, yes.

PI: Oh okay. So we are interested to hear about your experience when you access or provided with healthcare related to HIV interventions care here in this clinic at XXX (Name of Clinic). You don`t have to answer questions if you don`t want to neh....

QA013: Yes...

PI: The interview will take approximately 30-45 minutes.... I want to remind you that the information that you gonna be sharing with us here its gonna be confidential neh, what you say will not be connected back to you. Nobody will know cause the information gathered during the interview will be combined with the other interviews. No one will know who said it, when it was said or where it was said. There are no right or wrong answers, we are interested in what you think and your experiences please feel free to ask any question when it is unclear…

QA013: Okay…

RA: Do you have any questions before we begin?

QA013: Ahh not yet..

RA: Not yet…. Okay start time is 11:36am. Please tell me more about yourself.

QA013: Ahh myself I`m a good person like I like socializing and then socializing and one I like to talk with somebody, to share our experiences and sort off.

RA: How old are you?

QA013: 35 (years old)

RA: You are 35. Okay. Where are you from before you came here? Where are you from?

QA013: XXX (Name of Province and Area).

RA: Oh you from XXX (Name of Area)?
QA013: Yes…
RA: Are you married?
QA013: No.
RA: You not yet married?
QA013: Not.
RA: Do you have a partner though?
QA013: Yes, I got a partner.
RA: Do you stay with your partner?
QA013: No
RA: you don`t stay with your partner. You stay alone?
QA013: Yes
RA: Do you have any children?
QA013: No
RA: You don`t have any children. Okay can you tell me how long have you been living in this area.
QA013: Ahh since, since 2007.
RA: Since 2007.
QA013: Yes until now.
RA: So you`ve got 13 years living in this area.
QA013: Yes.
RA: So how long have you been using this clinic?
QA013: Ahh since 2013.
RA: Since 2013
QA013: Yes
RA: Is there any other clinic that you have used before, other than this one?

QA013: No

RA: Only this one?

QA013: Only this one.

RA: What do you like about this clinic?

QA013: Ahh first like you know it’s a treatment that they give you in this clinic is very good like if you ask something they give you everything you ask about it. Like if maybe you sick they can ask and then they gonna give you medicine and said okay here do this and do this so that`s why I`m usually coming this side.

RA: So what is it that you don`t like about this clinic?

QA013: Yahh Sometimes you find there because some people are not same people, some people you find it they got attitude you understand so that the problem that you have especially not all the sisters or whatever but some you find that there is no respect or whatever they just harass some people. You find you got adult people, adults then they just shout nje. I don`t know…

RA: Oh shouting of the adults?

QA013: Yes, yah adults so you have to respect other people and then they gonna respect you so that’s the problem that you have.

RA: Okay, can you tell me, are you HIV infected?

QA013: Yes

RA: Yes, okay so how long have you known your status?

QA013: Ehh since 13, 2013 I think until now…

RA: Since 2013

QA013: Yes

RA: Now its how many years?

QA013: Almost 7 years.

RA: So are you on treatment?

QA013: Yes

RA: How long have you been on treatment?

QA013: I started there on 2013. 7 years yes.

RA: Okay, lets get clear then. You tested in 2013?

QA013: Yes.

RA: And you started treatment same time in 2013?

QA013: Yes

RA: Oh okay, so its how long?

QA013: 7 years

RA: 7 years on treatment?

QA013: Yes

RA: So can you tell me what are major factors that are affecting your health right now.

QA013: Ahh so far there`s nothing. Its only that taking the treatment is going okay, for me there is no problem.

RA: There`s nothing that affects you?

QA013: No

RA: Oh okay,… so as you said there`s nothing that affects you, is there anything that you think affects other people when taking treatment?

QA013: Yah, you find some another people when you take treatment they come in rash or whatever or allergic with something that’s the problem but for me ah no the treatment is going okay.

RA: Oh okay.

RA: Okay can you tell me about your experience when it comes to service delivery when it comes to healthcare facility.

QA013: Ahh like for me its okay.

RA: When it come to service delivery in this clinic?

QA013: The only thing that I have the problem is time because you find that you come early around 5 o`clock but you end wait until 12 o`clock.

RA: Mmmm

QA013: But maybe its because there`s too much people or whatever? But that service people you find that you want to go to work but you end up not going to work and then you never provide or issue us with a letter from to commend at work, some you gonna give you but some you gonna refuse to give you.

RA: Okay…. So what are some of the positive features in the facility that you have visited?

QA013: Ahh its fine for me its fine eh…. Everything like it come, like I said everything like the service you find like when you come you get it. So that the pro….

RA: And what are the most challenging features you find in the facility?

QA013: Ah only thing is the time.

RA; Its`s time?

QA013: Yes, but maybe you understand because you find you got lot of people.

RA: Okay

QA013: Yes

RA: Can you tell me more about your experience when getting HIV care in this facility.

QA013: Ah… The experience first if to come here I find two, two sisters and then I asked them to can you, I got this problem can you help me, you welcome me nicely and help me with everything that I need.

RA: So you get help positive help from the sisters?

QA013: Yes…….

RA: So what are the thing that you would like to see improve about the health services in this facility?

QA013: Ah what is that only time arrange it about time.

RA: Okay if they can speed up?

QA013: Yah and maybe old people or adult people they can give them first service and then us maybe later because you find you got people like 60 years still on the que, standing a lot of the time almost for 12 to 4 to 6 hours but if you said okay adult people 60 years or pensioners can help first and us, we can wait there`s no problem.

RA: So now we are gonna talk about HIV prevention, your knowledge when it comes to HIV prevention.

QA013: Okay…

RA: What do you understand about HIV prevention?

QA013: Is that you always have to protect yourself to prevent and always use condoms, and then when you got a partner tell your partner about your status. Then you have to be clear to your partner about your status with your partner. That is what I can say.

RA: Can you tell me the different types of, of HIV prevention services that you know.

QA013: Ahh no. Use condom and then… I just know that you can prevent yourself by use condom and then you can circumcise but it doesn`t mean that you gonna prevent the, this but you gonna have to have one partner and know your status.

RA: Do you use condoms?

QA013: Yes I do.

RA: How do you use them and when do you use them?

QA013: I do you use them.

RA: Why do you use them?

QA013: To prevent other people and then to not to infect other people like to prevent your partner because sometimes if you keep quiet you can spread the disease to other people.

RA: How often do you use them? How often do you use the condoms?

QA013: Ahh for me it`s every time…

RA: Where do you get your condoms?

QA013: Ahh usually I`m getting like on the medical clinic like. You find that you put it on the outside there door and then you cross and then you pick it.

RA: Oh you get them from the clinic?

QA013: Yes

RA: Are there any other places where you get them besides the clinic, where you get your condoms?

QA013: No.

RA: There`s nowhere else?

QA013: No.

RA: So what prevents you or stop you from using the condom?

QA013: Ah no this one ahh.. I use them!!!

RA: What is it that can make you not to use the condom?

QA013: Ahh… I don`t know.

RA: You don`t know?

QA013: Yeah I don`t know.

RA: So what would prevent you from getting the condoms? Ukuwathole uyabona?

QA013: Ahh never because I make sure I`ve got it because I go to the clinic and got them.

RA: So ahh what do you…..

RA: Can you explain what the universal test and treat is.

QA013: The universal test and treat….

RA: Yes universal test and treat….

QA013: To how, to know your s… But this one I don`t know it, I don`t understand it.

RA: Okay, so universal test and treat is whereby we test you now and when you are, when your results are positive we give you the treatment now.

QA013: Okay.

RA: When you tested how did it happen?

QA013: I tested and then he said he gonna give me the tablets after that I gonna come back to the clinic. First when I tested, I tested on private doctor and he tell me and then he give me the tablet there and there and then he told me how to use it and then I came to the clinic and then I asked the sisters and then they tell me about everything I need.

RA: Yah that`s the universal test and treat, like we test you now like it happen to you. We test you today if the results are positive, we start you on treatment there and there neh.

QA013: Okay

RA: Yes. So what do you think of… So what are some of the advantages that you know about, what is good about this universal test and treat?

QA013: Because now you know your status and then you get, you get help there and there because sometimes when you get your results like you go home you find some people doesn`t want to come back and then some people just keep quiet end up doing things that affect other people.

RA: Mmm

QA013: Like you find some people got anger, some people are said okay I got this disease because I talk to, I infected with you who and the you go and fight that person. But if you get help there and there you find out that you know you got help and then there is no anger there because you know if I do this and then she or he this one who tested you is gonna explain you that thing is gonna help you with this and this and you gonna understand everything.

RA: Okay so what are some of the, the disadvantages, what is wrong about testing now and treating now?

QA013: Advantage because you know your status.

RA: Disadvantages...?

QA013: For me disadvantages… I don`t think, if you understand to like to prev…. to get everything like treatment like you get everything there`s no disadvantages if you find disadvantages you didn`t except you didn`t allow yourself to, first thing you have to except your status first and then I don`t think you gonna be have a disadvantage.

RA: Okay is there any change to the health services or health information has been delivered since you, your immediate ART began? Change the way you look at your own health?

QA013: Yah… Okay first I take treatment since 2013 until 2018 and on the 18 when my results came they said there`s no any, any symptoms that we, I have that. The sister said you still have to take the tablets then you`ll see. But on the, this year when the results came ahh the sister did tell me anything.

RA: Mmm

QA013: She didn`t tell me my results just write down the tablets, so I don`t know whether I still don`t have the disease or I still have the disease I don't know. So I waiting for the blood on December my results is gonna is gonna come on January so I will and see.

RA: Okay. So I heard you say in 2018 they said you don`t have the disease?

QA013: In my blood.

RA: In your blood?

QA013: Yes.

RA: What happened then, did you stop the treatment, or you continued?

QA013: I continue until now, I still continue.

RA: Okay yah because what happens is the, the tablets work on that so what they wanna do they suppress the virus so it`s a good thing if the virus is not showing in your blood.

QA013: Yes

RA: So you continue taking your tablets so that the virus can be suppressed neh.

QA013: Yes

RA: So even if the blood doesn`t show the increase of HIV, you still continue taking your treatment like make sure of that you don`t stop.

QA013: Okay

RA: Because that what the treatment is for to make sure that it destroys the virus. Neh.

RA: So are there any issues that you have experienced, that have prevented you from accessing your ARVs?

QA013: No.

RA: There are no issues?

QA013: There are no issues.

RA: What do you think would happen if on continues to take ART or stops taking their medication?

QA013: Ahh like if you stop taking your medication and then you can maybe you can be good for 3 to 6 months and then you find that later like it attacks you it`s gonna come and make you sick and not able to come, not able to walk maybe sometimes you gonna die because you stop it. And then and then when you come back and take the tablet that tablet is gonna maybe is gonna fail to help you.

RA: Mmm….

QA013: Like that because you have to continue maybe until the doctor tells you maybe you can stop or what.

RA: Okay so we are going to part four.

RA: Since accessing the facility for HIV prevention, could you explain how your life has been impacted?

QA013: Noh for me, my life is still the same. Because I still working because before when you go to test first time I saw not sick. I was like this and I still like this. It didn`t change anything.

RA: Okay.

RA: So can you explain the HIV prevention services you have, had been helpful to you?

QA013: Yes they been helpful to me because like some times you gonna prevent you on other diseases you understand. And then you stay with a positive every time, everything is normal, for me I don`t think maybe if I didn`t take the tablets maybe I would be dead now but now I still here, I`m fine so there`s no anything that I complain.

RA: Okay is there anything you wanna add, subtract?

QA013: Ahh for now no.

RA: Okay now its time for us to close this part of the interview. But before we do so, is there anything about the topic that we have discussed that you feel like is important to say?

QA013: The important thing that you want to know like the patients how do you feel about the HIV

because everything is preventable, everything is gonna help others if want to change som thing to do more and then for me I think its everything is good.

RA: Okay now we have come to the end of our discussion. Thank you for your participation. If you have any questions about the study please don`t hesitate to call us and thank you.

QA013: Okay.

RA: Okay now the time is 12h03

END
