# Supplementary material for: Understanding factors influencing utilization of HIV prevention and treatment services among patients and providers in a heterogeneous setting: A qualitative study from South Africa
Source: PLOS Glob Public Health. 2022 Feb 3;2(2):e0000132. doi: 10.1371/journal.pgph.0000132 (PMC10021737; doi:10.1371/journal.pgph.0000132)
Supplement: S1 Data — (ZIP) [file pgph.0000132.s001.zip › Supplementary information/IDI_Clinic attendee_QA014.pdf]

1 PARTICIPANT IDENTIFICATION NUMBER: QA014

2 INTERVIEWER: XXX (NAME OF RA)

3 TRANSCRIBER: XXX (NAME OF RA)

4 CLINIC NAME: XXX (NAME OF CLINIC)

6 TYPE OF THE PARTICIPANT: MALE CLINIC ATTENDEE

7 LANGUAGE: ENGLISH

8 I. Good afternoon again, Okay my name is XXX (Name of RA) I would to thank you of agreeing to take part in this interview. And for the purpose of the regulation please confirm that we audio record you.

9 P. Yes I do.

10 I. We are interested about hearing about accessing health services related to HIV prevention in this clinic. Which is XXX (Name of Clinic) clinic, you don't need to any answer questions if you don't want to. The interview will take approximately 30 to 45 minutes, I want to remind you that the information you share is confidential. What you say will not be connected back to you, while the information with this interview will be combined with other interviews no one would know who said it when it was said and how it was said. There are no wrong or right answers, we are interested in what you think and your experiences. Please feel free to ask me any questions if something it's not clear.

11 I. Do you have any questions before we begin?

12 P. Yah (yes) I have.

13 I. What is your question sir?

14 P. If you come here maybe you get the problem who am i supposed to consult to ask something like I am treating like bad or something is wrong who is in charge or to be responsibility for that, maybe if you have a problem?

15 I. Thank you so much for your question sir, to answer you to this clinic or in every clinic there is always a clinic manager.

16 P. Yes.

17 I. Whenever you feel like you have like compliant or suggestions that you want to make you can also contact the clinic manager. What you have to do is to ask for the clinic manager, you have the right to be directed to the exact person which the clinic manager.

18 P. Okay I will do.

19 I. Yes

20 I. Is there any other question?

21 P. Yes, since I come here to attend some staff so I got a problem actually my wife was coming here and they make something wrong because they miss something like a they mix blood several times, even if is not blood ticket for her it's not right, so when she come here then someone you know was in charge here. And the other one you must come the following days, then when she comes here there was no one here. So they said they didn't take her blood.

22 I. So she was not assisted in anyway?

23 P. She was assisted but they take a long process, everything of here its mixed up, its ups and down.

24 I. Okay, Okay. Hence I was saying.

25 P. Someone for the first time she made a mistake for her because maybe she didn't download everything of her document you see, and then after she left, and then after the following days she comes no one she knows about this.

26 I. Okay.

27 P. And then they have to right again for another things, and this thing is back to who didn't take the blood and she mix her blood.

28 I. Ohhh (yes)

29 P. Mixing the blood yes you understand what I am taking about.

30 I. Okay if I may get you well.

31 P. What I must supposed to do when it's like that?

32 I. Okay hence I was saying there is always a clinic manager in every clinic I think the right thing to do is to consult the clinic manager and explain your complaint to her. She will have the solution to your problem.

33 P. Okay.

34 I. Thank you, are you happy?

35 P. No yah (yes) I am happy.

36 I. Okay, the time now is 13:25 participant ID QAO14, Participant type its male interview language is English, and clinic name its XXX (Clinic Name).

37 I. Sir can you tell me more about yourself?

38 P. Okay, I am (xxx Name of the person) i was living in XXX (Name of Area) but I am moved by three months ago and I live at XXX (Name of Area) right now.

39 I. XXX (Name of Area)?

40 P. Yes XXX (Name of Area).

41 I. But is still in XXX (Name of District)?

42 P. Yes, around XXX (Name of District).

43 I. Okay, and how old are you?

44 P. I am 37 years old.

45 I. Okay, where are you from originally?

46 P. From XXX (Name of Province).

47 I. XXX (Name of Province)?

48 P. Yes XXXX (Name of province) province.

49 I. Are you married?

50 P. No I am not married.

51 I. Do you have any children?

52 P. Yes I have.

53 I. How many are they?

54 P. Two.

55 I. (Coughing) thank you so much for sharing such information with us.

56 I. Can you also tell me how long have you lived in this area?

57 P. This one?

58 I. Yes.

59 P. Alberton, I have lived about one year and half and I move to XXX (Name of Area).

60 I. Okay. So how long have you being visiting this clinic?

61 P. I think three month or four month (Noise at the background) from January yah (yes) from January.

62 I. January this year?

63 P. Yes.

64 I. 2020?

65 P. Yes.

66 I. Have you visited other clinics in this are accept this clinic which other clinics have you visited?

67 P. No, not yet.

68 I. Not yet? Only this clinic?

69 P. Yah (yes) only this one

70 I. Okay.

71 I. What do you like about this clinic?

72 P. I like this clinic because it's very clean some other staff are very patient to other person but some others have some attitude but not all of them maybe one or two.

73 I. Okay.

74 P. Yah (yes)

75 I. So I hear you say some other staff have some attitude can you elaborate what kind of attitude?

76 P. When you come you don't know what's going on and then you ask, they will say stay there and you said know I want to ask, and how can I stay there I want to ask.

77 I. Okay.

78 P. They say go and ask to other clinics you can see that we are working here. You understand so that attitude you have to listen before you say something to me. Don't say you are working here. Go and ask the toilet or something special for me. They think maybe you want to jump the line or something, you can be the last man on the line but if you have a problem right now I have to go to the sisters and ask something, or you want to say my grandfather is collapsing right now.

79 I. Mmm (yes)

80 P. They will say no don't come in you understand.

81 I. Mmm (yes)

82 P. But outside there is something happening you see so I don't want this attitude but you have to listen to them, and what he is asking for.

83 I. Yes.

84 p. You see.

85 I. No its clear, it's very clear.

86 I. So could you tell me if you are HIV infected? Are you HIV positive?

87 P. This thing its confidential.

88 I. It is confidential however most of our questions are based on people who are HIV positive so like if I ask something like ARVS if you are HIV negative you don't know how to answer me. But if you are positive you must have knowledge as well to answer me.

89 P. Yah (yes)

90 I. Yes. And remember I insure you that this is between us, no one else will get to know about your status.

91 P. Yah (yes)

92 I. The reason why I am asking is the sake of the study, I think you are right now,

So make sure if you would like me to repeat the question again.

94 P. Which question?

95 I. The same question that I ask whether you are HIV infected.

96 P. Yes I am HIV.

97 I. Okay, are you in any treatment?

98 P. Yes

99 I. Since you are HIV positive?

100 P. Yes.

101 I. What treatment is that?

102 P. I collect the ARVS.

103 I. The ARVS?

104 P. Yes.

105 I . So you are on ARV medication?

106 P. Yes.

107 I. Okay, for how long have you being for ARVS?

108 P. From January.

109 I. January which year?

110 P. This year.

111 I. So you are roughly seven month on treatment?

112 P. Yes.

113 I. Okay, can you tell me what are the major factors affection your health right now? Accept for HIV what else affects you health?

114 P. I am okay nothing is affecting me right now.

115 I. Okay, so the is nothing that affects your health right now?

116 P. Yes.

117 I. So do you think other people have other factor affects their health right now?

118 P. Some people they are affected with the affect like HIV because they drink alcohol and they don't drink treatment that way or sometimes they ignore it because he or she is affected

119 I. Okay, if I may ask the question again you think this factors affects other people you know as well (Noise at the background)

120 P. No I don't have any one.

121 I. Not even one at home or friends?

122 P. No one.

123 I. Okay, sir now we are going to talk about health care in general.

124 I. Can you tell me your experiences of service delivery from health facilities from health care clinics? How do you find the experiences in terms of service delivery ?

125 P. In service delivery some others they are right some others the is not right because if you work with people sometimes you get like disturbing in your mind, sometimes you shout most of the time they all human and they are all fathers and they come to the clinic so I don't understand sometimes what's happening you see. But especial I am taking for them because they are scared to ask. (Noise at the background)

126 I. Okay.

127 P. But you kind like I am a father, and they stand here for more than four hours you understand and it's like they get tired sometimes you see the is no shelters here.

128 I. Mmm (yes)

129 P. In some other clinics the is no shelters and they are standing on the sun more than four hours.

130 I. Mmm (Yes)

131 P. But me I can try. Let say if she must come late and you gonna take him infrant of the people but if you see on the line you can tell, but you talk with the people don't mind this is an elder person.

132 I. In other words you are saying elder people or disable people must be given first preference?

133 P. Yes.

134 I. In terms of accessing health care.

135 P. Yah (yes)

136 I. I get you well my friend.

137 I. So what are the some of positive features in the facility that you have visited, what is about other clinics that you have visited. What is good about it?

138 P. No I like to come to clinic some others guy, or some nurse or Doctor they want to know what do you want and you can't stand here for more than four hours after you come this side and then they know who must do this and this, when the guys when they come on the line you come they ask one by one. So that you this thing you don't have you must go home to fetch it and then come again so that you understand if don't have ID but you stand to the line for four hours. And when they need ID you didn't have why you didn't ask us before, you understand.

139 I. Mmm (yes)

140 P. If maybe I am going around town or somewhere I fetch and come and I check it, and is anyone in the line you understand so that even the line is gonna be fast and now it's a season of covid- 19.

141 I. Mmm (yes)

142 P. You understand.

143 I. Yes.

144 P. So you avoid for more people crowded you understand?

145 I. Yes. I get your point my friend.

146 I. What are the most challenging features in the facility that you have visited?

147 P. The challenging of, can you repeat again.

148 I. What are the most challenging features in the clinic that you have visited?

149 P. Sometimes they take time to attend a patient to see some other staff like they ignore you they will be busy phoning. Now and after that they go to the lunch you see.

150 I. Mmm (yes)

151 I. Does that also happen in this clinic in XXX (Name of Area)?

152 P. No, not on this clinic but some others.

153 I. Okay.

154 P. Yah (yes)

156 I. Can you tell me more about your experience of getting HIV care, how do you find it? Maybe you come here for your HIV medication what kind of treatment that you get?

157 P. From me?

158 I. From the clinic, how are they treating you when you are here? When you come for HIV care for your medication particularly?

159 P. No they treat me alright because they ask me what are the side effects from as you have being starting the treatment and the is no something effects. And there is infection or side effects from me as from now.

160 I. Okay. And what are other things you would like to improve about health services in your health facility, what do you want improved here.

161 P. No, what I want to improve is even if you would like to get so work, like even if you want to get to the toilet you understand. (Noise at the background)

162 I. Mmm (yes).

163 P. You understand. Maybe they must put some did you see that board.

164 I. Mmm (yes)

165 P. If you want a drink or you want the toilet you understand. If people feel comfortable at the clinic, don't say if I come inside I want the toilet and why you come here because there is a line outside on the toilet but the is no toilet outside and when I pee outside its wrong you understand.

166 I. Yah (yes)

167 P. Yah (yes)

168 P. Its natural, cos when you need I toilet its natural, if are need a drink its natural. I need water right now.

169 I. Okay.

170 P. Course it's time for me to drink water, like when I need the tablets but they have a problem when you come inside they block you no we are working here. And they don't know how to listen to me they just say no just wait outside, most of the clinics are like this.

171 I. So you want them to improve ?

172 P. Yes. But they must listen to the person what are you looking for.

173 I. I understand.

174 P. You say no I am asking for the toilet or if I want to follow this line this line is for sickness people or this one is for the collecting people.

175 I. Mmm (yes)

176 P. Or I never join the line, after that you have to join this line.

177 I. Okay.

178 P. You understand.

179 P. Or I have to jump this line so they must update everyone so that if you want to join this clinic you must join this line, when you come to do this you must come to this line so that you must not get confused.

180 P. Okay I understand

181 I. In other word what you are asking for is for the clinic to have a marshall ?

182 P. Exactly, like at the bank see like at the bank you say no I want to do like opening the account. And they direct you to the right place, and that thing is gone makes everyone to be quick and go home so that to avoid more people, you understand.

186 I. Yah (yes) it's clear sir.

187 I. Now we are going to talk about HIV prevention.

188 P. Yah (yes)

189 I. What do you understand about HIV Prevention?

190 P. HIV prevention the first thing is to abstain, it is your opinion, you have to prevent to use condom.

191 I. Use a condom yah (yes)

192 P. I have to use condom for protection.

193 I. Okay.

194 P. Yes.

195 I. So can you tell me the different types of HIV prevention services?

196 P. Sorry can you repeat again.

197 I. Can you tell me the different types of HIV prevention services?

198 P. Different types of?

199 I. HIV prevention services.

200 P. Is to up stain and use condoms

201 I. Yes

202 P. I think is all that is to use condom.

203 I. Okay.

204 I. So what are some of the difficulties you may experience in accessing HIV prevention services, what may stop you to access HIV prevention service?

205 P. Sometimes when you do like having sex you have to control, don't drink things like drunk you understand and use your mind and take something like simple you understand.

206 I. So for me to understand you better if you are drunk or take too much alcohol and then it might make you not to access HIV prevention service difficult.

207 P. Yes.

208 I. What else you can say?

209 P. According to that prevention for?

210 I. According to the difficulties you may experience in accessing HIV prevention services?

211 P. You have to keep on having condoms on your place.

212 I. Okay.

213 P. Whenever keep on having condoms, you don't have to be scared taking out the condom because it's your life be protect yourself and others.

214 I. Alright.

215 I. So the next question it's about condoms.

216 P. Yes.

217 I. Do you use condoms?

218 P. Yes I use condom.

216 I. If I may ask why do you use them?

217 P. I use them to protect me myself and those I am busy doing with them.

218 I. Okay.

219 P. Yes.

220 p. And prevent for making pregnancies.

221 I. So how often to you use them?

222 P. Sorry.

223 I. How often do you use condoms?

224 P. To use condom ?

225 I. Yes how often? Like daily like sometimes?

226 P. Every daily basic not sometimes.

227 I. Okay.

228 P. On a daily basic when I am doing I have to protect myself.

229 I. Okay. So where do you get the condoms from?

230 P. I get from the clinics sometimes even at work they have condoms, even in the shop I get it from there.

231 I. Okay, what will prevent you from using condoms what can stop you from using condoms?

232 P. What can stop me from using condom?

233 I. Yes

234 P. I think maybe when I agree with my wife that we want to make a baby and then maybe we can stop and check everything if we are the right condition to stop using condom and have a baby or something.

235 I. Okay

335 P. According to my wife.

336 I. Other than that the is nothing?

337 P. The is nothing.

338 I. Okay.

339 I. Now what can prevent you from getting condoms, what get prevent you from getting condoms?

340 P. I think the is nothing can stop me from getting the condom. As long as the government is still placing everywhere, most of the people they are busy they don't have time at work, maybe I want to do sex then I don't have money. I have to buy the condoms but those condoms from the government the is no using money you just take them just for free.

341 I. Okay.

342 I. Can you explain what the universal test and treat is, do you know anything about the universal test and treat?

343 P. No I don't know.

344 I. Let me give you an idea what it is, universal test and treat is when you come to the clinic to do for HIV testing, you test if your results are positive they gave you medication ARVS today.

345 P. Yah (yes)

346 I. That's what we call universal test and treat you understand.

347 P. Yes sir

348 I. So what are the some of advantages of UTT, Of that universal test and treat. What do you think is good for test and treat at the same time.

349 P. Yah (yes) I think it's good it's to know year status you have to test. So that you can know your life what you are right now.

350 I. Okay.

351 P. Sometimes most of the time you get flu you get some infection in your life and in your body you have to know what's going on, you understand.

352 I. Okay.

353 P. So that you know your status.

354 I. Okay.

355 I. And what do you think is bad about universal test and treat? A disadvantages of it UTT?

356 P. What is a??

357 I. What do you think could be the disadvantages?

358 P. Yah (yes) it could be advantage if you test, or people who test me can expose me and say this guy its positive I will be feeling uncomfortable. Cos it's my confidential.

359 I. Mmm (yes)

360 P. You understand and I have to, or if you test me you have to know your story, to wash your hands and put your gloves.

361 I. Mmm (yes)

362 P. You see, but to protect me and even me to protect you. So that we don't escalate for everyone.

363 I. Okay, has the be any changes to health information or health services has being delivered since immediate ART, that you change to look for your own health?

364 P. Such a, the is nothing you can say the is something bad or.

365 I. Okay.

366 I. So have you notice any changes?

367 P. No.

368 I. Okay.

369. I. What issues have you experience that prevented you from accessing or taking ARVS? Do you find any problem taking ARVS or getting them?

370 P. No (Noise at the background)

371 I. So you are telling me that is easy to access the ARVS?

372 p. Yes is easy to access ARVS because they is not separating us, and then people are scared to say this line is for ARVS but if they can say go to the room so that people cannot be scared to get she or his treatment.

373 I. Okay.

374 I. So what do you think could happen if one continue to take ART, if you like yourself if continue to drink your ARVS what happens to you?

375 P. If I continue to take ART.

376 I. What do you think it will happen to you? In terms of your health.

377 P. Ahhh esh (thinking) I don't know maybe if you drink some things and flushes out some other certain time.

378 I. Okay, what I am trying to ask here is that if you continue you keep on taking your ARVS. What happens to your body? You always taking your ARVS.

379 P. Like what happens?

380 I. In terms of your health.

381 P. As for right now or for in future.

382 I. Already you are taking ARVS neh

383 P. Yah (yes)

384 I. Right, let's say you stop taking ARVS what will happen to you?

385 P. Okay, no I think when I am stop it my CD4 maybe is gonna be not right?

386 I. Mmm (yes)

387 P. Because my body is not right, something like my body is gonna change. Default something like that changes and those powers, you understand and then appetite not gonna be alright. And food and many things could happen or change.

388 I. But if you continue taking them what will happen? If you does stop taking them?

389 P. I think my body is gonna be alright if the Doctor says no you can stop because he check some other times.

390 I. Since accessing the facility of HIV prevention could you explain how your life has being impacted? Ever since you have starting for your HIV medication, do you think your life has change? In any way?

391 P. If like?

392 I. The question is since accessing the facility of HIV prevention?

393 P. Yah (yes)

394 I. Could you explain how your life could be impacted ? like what is the changes since you come here for HIV medication or condom and staff? How your life change?

395 P. No my life is like it change a little bit because I can take care of myself. Because I do alcohol but now I am limited and then I need to know the time to drink my medication. And I have to avoid some other things for my health.

396 I. Okay.

397 P. Like I am not smoking anymore, and I am drinking alcohol on a weekend not on a daily weekend but on that time.

398 I. So you are trying to say your life has changed in such a way that you consuming less alcohol now?

399 P. Yah (yes)

400 I. Okay. Not like before when you drink a lot?

401 P. Yah (yes)

402 I. Okay.

403 I. Can you explain the HIV prevention service you think has being helpful to you?

404 P. For?

405 I. I mean like, can you explain the HIV prevention service you think has being helpful to you, like taking condoms and using ARVS how it helped you?

406 P. Yah (yes) same thing because my body is still okay I have that power like before and not to felt for other one. For use condoms or.

407 I. Yah (yes)

408 P. Yes.

409 I. Okay, so they have being very helpful?

410 P. Yah (yes) very helpful.

411 I. Okay.

412 I. Sir it's time for us to closed this part of the interview but before we do is there anything else, in this topic you feel we don't discuss that you feel it's very important, you think the is anything we didn't touch that you would like use to talk about?

413 P. Yah (yes) I would like to say like something .

414 I. What is it sir?

415 P. I would like to know *kuthi (that)* why the is condoms ? I Saw some people they say they want to buy some condom those ones from government which is not perfect I want to know. Because they say that if you use this one you are in danger it can blast. You have to buy on a shop trust condom its maybe R8 rand or and you see. And this one it's not like comfortable for the people who don't have money.

416 I. So your question it's basically like is using government condoms save?

417 P. Like its unsafe 100 percent you understand? What's that using condom of government and that one of shop. Its still the same or because they are running a business or something, what's the different?

418 I. Government condoms are always the safest, why I am saying this even the health world organization has approved of this condoms. The reason why the condom being distributed to the clinic is or in the hospitals is not all people have money to buy condoms, so is to help those how don't have money and get them free, so the is no difference between the two condoms. If you use the clinic one or one from the pharmacy is still the same.

419 P. Okay.

420 I. They all provide protection.

421 P. Okay.

422 I. I hope now you are happy?

423 P. Yah (yes) I am happy.

424 I. So it's even advisable if you don't have money to buy go to the clinics and fetch free condoms.

425 P. Yah (yes)

426 I. They perform the same duties, you understand?

427 P. Yah (yes)

428 I. Okay now we have come to the end of our discussion, thank you for your participation. If you have any question about our study please contact us. I would like to thank you.

429 P. Okay thank you

430 I. The time is 13:56.

Glossary

Default= stopping taking medication

HIV= Human immunodeficiency virus

ARVS= Antiretrovirals

ART= Antiretroviral therapy
