# Supplementary material for: Understanding factors influencing utilization of HIV prevention and treatment services among patients and providers in a heterogeneous setting: A qualitative study from South Africa
Source: PLOS Glob Public Health. 2022 Feb 3;2(2):e0000132. doi: 10.1371/journal.pgph.0000132 (PMC10021737; doi:10.1371/journal.pgph.0000132)
Supplement: S1 Data — (ZIP) [file pgph.0000132.s001.zip › Supplementary information/IDI_Clinic attendee_QA015.pdf]

Full participant ID: QA015

Participant Type: Female

Location: XXX (Name of clinic)

Date:20 July 2020

Primary interview language: English

I: Alright, eh time is 14:09. Eh participant ID is QA015. Participant is a female 44 years old. Thank you very much for your time, my name is *(XXX Name of the interviewer)* and I will be your Interviewer for the day.

P: Ok

I: Yes, can you tell me more about yourself?

P: Ok My name is *(XXX Name of a person*) I am 44 years old…

I: Yes

P: I am married, with two kids I am not working at this present moment.

I: Yes

P: Eh I am a very quiet person, who like to socialize with people

I: Yeah

P: Yeah but I normally come to this clinic this clinic after three months I think, yes after three month. Yeah as I'm taking the chronic medications for high-blood.

I: Alright

P: Yeah

I: So you mentioned that you have kids, how old are they?

P: The first born is 18 and the 2nd born is 6 years

I: Alright, How often do you come to the clinic?

P: Once in three months.

I: Once in three months…

P: Mmm

I: Alright, How long have you been living in this area?

P: Mmm (*Thinking*) About 5 years from now.

I: Ok, Is there any other clinic that you once visited?

P: Yes (XXX Name of a clinic)

I: Alright (XXX Name of a clinic), Alright What do you like about this (XXX Name of a Clinic)?

P: Nothing much except that I am staying in (XXX Name of a place).

I: Owk, then what do you dislike if… what do you dislike then?

P: They are taking long, you stand for long hours. Today I came about 10:00 in the morning, I even went back home and come again but people are still there were they where, where I left them, so problem it's the queue and I don't know weather its short of staff or what but its taking long. I don't know why but this thing has been going on for long time now. Its not a new thing that was caused by lockdown or anything like that, no its been going on forever.

I: Alright, eh could you… eh I'm going to ask you a personal question about your HIV status. How often do you test?

P: Test... yoh almost every time when I come to the clinic mostly.

I: Alright

P: But I went to the hospital last month, yeah that's the last time I tested.

I: How were your results?

P: Negative

I: Alright, And then what do you understand about HIV prevention services that you know of?

P: I know that you have to protect yourself with HIV as you know people are sick, people are getting sick. Now we have Corona which is killing people, so its nice to be safe in most of the time.

I: Alright

P: And HIV, I wont say it kills because you have to protect yourself to prevent it that's it.

I: Yes, So what are the prevention services that you know of, which are provided in the clinic?

I: Services yes, which are provided in the clinic?

P: Prevention of what?

I: Prevention of HIV. Protection or prevention

P: Only condoms

I: Only condoms

P: Yeah

I: How accessible are they?

P: No they are accessible,

I: Alright, Which one? Which condoms are they providing is it females or male?

P: Both

I: Males and females, alright. How often do you see people going out having condoms from the clinic?

P: Not most of the time

I: Ok. Meaning they are not always taking them

P: No

I: Alright. Are there any factors affecting your health right now?

P: No

I: Ok, Do you know other people who are affected by any factors?

P: Any?

I: Anything, who's affected Healthwise close to you?

P: Healthwise in terms of?

I: any sickness

P: Any sickness, Yeah I have a sick cousin?

I: Is he or she coming to the clinic or the clinic is coming to her?

P: No, shes going to the clinic.

I: Alright, is it user friendly?

P: I don't know.

I: Alright you don't know, alright; in terms of service delivery how do you find… how to find it?

P: As I said this clinic is very good and is helpful except as I said that the queue is taking long and the service.

I: Ok, Alright. Meaning there are more positives than negative.

P: Yeah

I: What's good about this clinic? Except being good, what else that you picked up at this clinic that is good?

P: In this clinic when you are sick, they do provide with medicine, they even have the physio, yeah their services is good otherwise.

I: Alright so, what do you understand about the… which information do you have about HIV? After being tested, after being tested positive in general what do you understand about HIV?

P: I don't know anything except that, you have to take your medication if you are positive.

I: Yeah

P: For you to get better

I: Alright, eh Do you know anything to do with UTT the method of testing today and then being initiated today? have you heard about it?

P: No.

I: Its only the prevention part?

P: Yeah, You were asking about the UTT. What is UTT?

I: UTT It's a method were you test a person today and you treat the person today. Before they use to delay starting ARVs.

P: Alright

I: Due to suppression of… yeah back then they use to delay they were not initiating them same day. Yeah so now today, these days you test positive today. They initiate and start treatment same day

P: What I know before is that we use to… when you going for a test for HIV, maybe if you go lets say I am going for a test for HIV.

I: Yes, I: Yeah so now today, these days you test positive today. They initiate and start treatment same day

P: They will test you before you go in.

I: Mmm (yes)

P: And then when you tested negative, they will take you for counselling but these days I see this tents all over.

I: Mmm mmm (Yes)

P: Hospitals, Clinics and everything they are doing test without counselling the people, is that safe?

I: According to the department of health those people are qualified counsellors.

P: But they do not counsel you.

I: Eh I don't know about that, but they are… I will make follow up.

P: Ok

I: Yes, So eh do you also receive counselling here in this clinic?

P: I never come for counselling.

I: Alright, Ok. Do you also access condoms here in this clinic?

P: Yes

I: OK and then which other prevention services do you access? That you are using

P: Nothing

I: Alright, its only condoms.

P: Yeah.

I: Alright, do you have any question before we continue?

P: No

I: Alright so since you came here today with your husband… is he your husband?

P: No

I: Who is he

P: I came alone.

I: Oh you came alone

P: mmm (Yes)

I: Alright, I thought you said he is in a queue.

P: I said I am after that guy.

I: Ohh Alright, alright. Ok so, eh What do you understand about ARVs? In a distance

P: Eh I don't know much because I am not using them. If I was using them it was going to be easy to answer that question.

I: Mmm

P: but I know that they help CD4 Count or something like that.

I: Alright

P: For when they are low or high.

I: Alright.

P: They are preventing actually, the virus to spread.

I: Alright.

P: Its only, yeah that's the only things I know.

I: You mentioned something to do with accessing condoms in this clinic…

P: Mmm (yes)

I: Ehh how often do you use them? Do you use them all the time?

P: I use to.

I: Oh alright

P: Before I fell pregnant.

I: Alright [laughing], eh so before when you were experiencing … when you were using them did you face any challenges?

P: No

I: Alright, okay. We have come to the end of our discussion, is there any other question or any input, something that you would like us to talk about?

P: Nothing as much as said that I have a problem that people that are doing HIV test on the clinics or in the hospitals these days they don’t do counselling.

I: Alright

P: So that thing it can affect people because sometimes you wont know that you are positive but if people are just testing you without letting you know what are you facing, your challenges in the testing. It can damage people.

I: Alright

P: It can give stress.

I: Yeah

P: I can fall down by heart attack so I don’t dispute people testing but they must do them with the procedure of counselling so that I could face whatever I could get that it could be tested.

I: Alright, Thank you very much and thank you very much for your time.

P: Okay

I: Time is 14:24
