# Supplementary material for: Understanding factors influencing utilization of HIV prevention and treatment services among patients and providers in a heterogeneous setting: A qualitative study from South Africa
Source: PLOS Glob Public Health. 2022 Feb 3;2(2):e0000132. doi: 10.1371/journal.pgph.0000132 (PMC10021737; doi:10.1371/journal.pgph.0000132)
Supplement: S1 Data — (ZIP) [file pgph.0000132.s001.zip › Supplementary information/IDI_Clinic attendee_QA016.pdf]

TYPE OT INTERVIEW: QUALITATIVE

DATE: 20 JULY 2020

INTERVIEWEE: CLINIC ATTENDEE

INTERVEIWER: XXX (NAME OF RA)

RA: Okay time 13:40 interview participant number QA016 a female from XXX (Name of Clinic). Ah thank you mama for agreeing to have the interview with us and thank you for your time. And again, do you agree to be part of this interview, this recorded interview?

QA016: Yes…

RA: Could you please speak up a bit.

QA016: Yes.

RA: Okay, so we are interested to hear about your experiences in accessing HIV health services to, HIV interventions in this clinic, eh you do not have to answer questions if you don`t want to ehh the interview will take approximately 30-45 minutes and I want to remind you that the information that you share remain confidential. What you say cannot be connected back to you, while the information is being gathered during this interview will be combined with other interviews no one will know who said what or where it was said. There are no wrong or wright answers neh. We are interested in what you think and your experiences neh. Please feel free to ask questions when you not clear, something is not clear neh. So before we begin do you have any questions?

QA016: Mmm….

RA: Please speak up so we can hear clearly...

QA016: Ah No questions.

RA: Alright, so we are starting I will ask you just your background information, tell me about yourself.

QA016: My name is (###### #######). I`m from XXX (Name of Province), I`m stay number XXX (Name of Area). I stay because I`m working there. Ahh I have children but not stay with me, they stay with the grans (grandparents) with my mom. I`m honest people, I like to share things with the other people.

RA: How old are you?

QA016: 43 years.

RA: How old are you?

QA016: 43.

RA: You are 43 years old?

QA016: Yes.

RA: So, I heard you say you are from XXX (Name of Mpumalanga).
QA016: Yes
RA: Are you married?
QA016: No.
RA: You not married.
QA016: Yes
RA: So, I heard you talk about children, how many children do you have?
QA013: Three children.
RA: How old are they?
QA016: First born is 27 years, second born is 20 years and the last born is 14 years.
RA: okay, so tell me how long have you lived in this area?
QA016: Mmm from 2013(year)
RA: Mmm
QA016: When I`m from XXX (Name of Area).
RA: Okay
QA016: I come to start work here from 2013 and started clinic that time.
RA: Okay so, you started attending the clinic that time?
QA016: Yes.
RA: Okay. So, you started attending the clinic when you arrive here in 2013?
QA016: Yah the clinic from XXX (Name of Area), they referred me to this clinic.
RA: Oh, they transferred you from XXX (Name of Area) to this clinic?
QA016: Yah
RA: Have you visited any other clinic in this area?
QA016: In XXX (Name of Area). No.
RA: Only this XXX (Name of Clinic)?
QA016: Yah
RA: So, what do you like about this clinic?

QA016: Ahh I like their services; their service is good they treat people nicely. They help to you nice, they ask you, if you come you say you have headache they ask when it start, where do you feel maybe is it nausea they ask many things they ask.

RA: Mmm…

QA016 People they give you medication.

RA: So what it that you don`t like about this clinic?

QA016: Since I didn`t see anything wrong.

RA: Mmm..

QA016: I`m happy.

RA: You happy with the way they operate?

QA016: Yes.

RA: So there`s nothing that you don`t like about this clinic?

QA016: Yes.

RA: Okay. So, we gonna ask you personal question now. Can you tell me whether you HIV positive or not?

QA016: I`m HIV positive.

RA: You HIV positive.

QA016: And sugar diabetic too.

RA: Oh, so you attend two chronic in this clinic?

QA016: Mmm(yes)

RA: Okay so , how long have you known that you are HIV positive?

QA016: Mmm since 2008(year).

RA: 2008.

QA016: Yes

RA: So that`s how many years?

RA: That`s 12 years now?

QA016: Mmm(yes)

RA: So you said you sugar diabetes as well?

QA016: Mmm…(yes)

RA: How long have you been on sugar diabetes medication?

QA016: Mm in 2009 or late 2008.

RA: So, it's the same time with your ART?

QA016: Yes.

RA: So can you tell me, what are major factors affecting your health right now?

QA016: Baba….(pardon)

RA: What are major factors, what is affects your health now?

QA016: Sugar diabetes is affecting my health than HIV.

RA: Mm….

RA: So, do you think these facts affect other people as well?

QA016: Yah, like my child, he affected because they feel pain all the time when I say my sugar is up or my sugar is down like that.

RA: Mm….

QA016: They feel maybe I will go and leave them.

RA: Oh okay.

QA016: Yah. I feel it affects them…

RA: It affects them in a bad way neh?

QA016: Yah…

RA: Okay now we are going to talk about general health, health in general neh…

RA: Can you tell me your experience in terms of service delivery from this healthcare facility.

QA016: The service?

RA: Yes the services that you get from this clinic. What are your experience with the services you get from this clinic?

QA016: When I collect my treatment?

RA: Yes, how is the service?

QA016: The service I think its fine because if my sugar level is not up, they give me treatment for 3months.

RA: Mm…

QA016: But if my sugar diabetes is up, they give me for one month and then they going to check

what`s going on with my sugar levels if I maintain right, they give me 3months or 2months.

RA: Oh Okay

QA016: They check actually, they want to know what`s going on with you and they tell you to do things, right things for your health.

RA: Mm…(Sorry okay)

RA: What are the most positive features the facility, in the facility that you visited? What is positive about coming to this facility?

QA016: I don`t understand.

RA: Okay so if you come to this facility what is positive like what is right that, that you see when you visit this clinic?

QA016: When I come to this clinic even when I come to collect my treatment, they don`t just give me treatment, they ask me how do, you feel…

RA: Mm…

QA016: And tell them how do I feel…

RA: Mm…

QA016: If there`s something maybe they don`t understand or maybe need something else they give me that thing.

RA: Okay

QA016: So, with that one I say its positive for me.

RA: Okay and then what is the challenging? What the challenging that`s not right that you see here?

QA016: Mm… The thing is not right to see maybe is from the people, you see the other people maybe they come from the clinic to collect treatment and they don`t use it. This thing, the thing is me, you see I use treatment someone will come and collect treatment and put at home not use and me when I come to collect treatment I didn`t get that treatment because other people take it home for nothing because they don’t use it.

RA: Mm…

QA016: She`s sick but don`t use treatment…

RA: Okay so, if I hear you, you say people that take medication but not using it? That`s the challenge in this clinic.

QA016: Yah, that`s the bad thing.

RA: Okay so, who`s taking this medication? The other clients or the staff?

QA016: The other clients.

RA: Not the clinic staff?

QA016: Yes

RA: Okay, can you tell me about your experience when getting HIV care. Ma uzolanda itreatment yakho yeHIV about your experience. (When collecting your treatment for HIV tell me about your experience).

QA016: The medication of the HIV?

RA: Mm…

QA016: When you come here they say if the treatment they give you is not good for your body, maybe you see something for you breast maybe growing up you have to tell them, this treatment is not good for me they change and give you the other treatment. That`s the experience that I have.

RA: So, what are the things that you would like to see improve in the, about the health services in this facility?

QA016: Yah… I don`t know what are you saying…

RA: What would you want to see change so that the service can be improved, so that izobangcono(better) service in the clinic.

QA016: Is like the dates, when they give us to come to collect our treatment it seems like they give to much people one date, like you see now there are many people here now. But now I think maybe it`s Corona(virus) that is why people are too much like this because…

RA: Mm…

QA016: You need the service like I can`t say more, but I like if they give people different dates.

RA: Oh okay. So now we gonna come to HIV prevention.

RA: What do you understand about HIV prevention?

QA016: I understand about HIV prevention like, condomise.

RA: Mm…

QA016: Mm… To don`t touch someone’s blood..

RA: Mm… Okay can you tell me about; can you tell me the different types of HIV prevention services.

QA016: Ahh… I don`t know, all I know you don`t, you to use condom and that one to not touch anyone’s part to touch, to help someone who is getting hurt you use gloves.

RA: Mm… What are some of the difficulties that you may experience in accessing some of the HIV prevention services?

QA016: The different what are you saying?

RA: Yeh the different difficulties that you experience when you accessing HIV prevention services.

RA: Like the difficulties that you may face when you want to get condoms?

QA016: Condom is free…

RA: Mm… So there are no difficulties there?

QA016: No

RA: Okay gloves, to help someone is hurt as you`ve said?

QA016: Yah gloves sometimes its difficult because we, you on the road, you don`t have gloves on our pocket so when someone get hurt and you need to help that thing will be difficult because you don`t have gloves and you want to help you see you end up to touch.

RA: Okay… Do you use condom?

QA016: Yes

RA: Why do you use them?

QA016: To prevent cause, to prevent someone even me because when you have sex without a condom the HIV is going up up….

RA: Mm….

QA016: It multiply itself.

RA: How often do you use condoms?

QA016: Ayy let's say maybe I`m not too much to sex maybe 3 or 4 a month maybe 3 or 4

RA: Okay. Where do you get them condoms from?

QA016: Clinic

RA: You get your condoms from the clinic? Are there any other place you get condoms from?

QA016: Most of the time is from clinic…

RA: Mm…

QA016: But also in a Tarven there`s condoms there…

RA: Okay…

QA016: Some other shops they have free condoms just to say why don`t you take.

RA: What would prevent you from using condoms?

QA016: Prevent you, prevent from pregnancy…

RA: What can stop you from using condoms?

QA016: Mm… Nothing

RA: Nothing that can prevent you from using condoms.

QA016: Yes

RA: What would prevent you from getting condoms? What would stop you from getting condoms?

QA016: Maybe if the free condoms is finished.

RA: Mm

QA016: No free condoms at all, maybe is finish at all the world maybe I don`t have money to buy.

RA: Mm…

QA016: But I think if there is no money to buy no sex.

RA: Okay

((Laughter))

RA: Okay, so there is this thing. Can you explain what universal test and treat is? Do you know what is universal test and treat is?

QA016: No.

RA: So that is the, the process whereby you test now and then you get the treatment now. Unlike the olden days where you test and go and come back and then only start treatment at a later stage. So nower days we`ve got this universal test and treat, its whereby if I test today and I found out that I`m HIV positive I start treatment now, so that is universal test and treat neh mama.

QA016: Okay

RA: So what do you think maybe the advantages of this process.

QA016: Yah it`s good to the people because let say maybe someone gets, get tested and maybe that HIV is long time stay in your body so it good when you test now and get treatment now.

RA: Mm okay. What do you think might be the disadvantages of this test and treat?

QA016: The disadvantages, sometimes the test is say you are positive when you go to take blood and go to the laboratory maybe it comes not, no positive so that`s the disadvantage because you would have started treatment for nothing yah….

RA: For nothing okay mama…

RA: Has there been any change to the way health information or health services had been delivered to you since the immediate ART had began when you look at your own health?

QA016: Mm…

RA: Has there been any changes to the way health information or health services had been delivered since your immediate since uqale amaphilisi neh khona ichnge oyibonile mawuthola usizo eclinic? MAwunikwa usizo eclinic?

QA016: Yah khona ukuqala ampills eHIV or kuphi? Kukhona usizo(Yes there’s help).

RA: Mm…

QA016: Mm(yes) until kuqhamuka(there was) diabetes now but for HIV only ngibonile usizo lapho ngiye ngabaright nomzimba wami uyewaba right until diabetes ifikake ngachanger konke but still I`m fine(..for HIV only I saw help there I became alright even my body became right until diabetes comes and changed everything …)

RA: Okay, what or are there any issues since you started that prevented you from accessing or taking you ARVS? Kukhona mhlampe. Is there any experience that prevented you from accessing or taking ARVs?

QA016: Into engangisiza?

RA: Kukhona am? Is there any experience khona mhlampe oye wahlangabezana nakho experience izinkinga eziye zaku vimba ukuthi ungathathi amapills?

QA016: No.

RA: Okay

QA016: Azikho eziye zangivimba and azikho engizazivumela ukuthi zingi vimbe (There has been no challenges and I won`t allow any challenges to stop me from taking my pills).

RA: Mm okay so you won`t allow any to stop you from accessing the treatment?

QA016: Mm (yes)

RA: Okay, what do you think would happen if one could stop taking ATVs or stop taking their medication?

QA016: If you stop to take your medication you going to die.

RA: Mm, if you continue taking your medication?

QA016: You are alive. Maybe many years.

RA: Mm… Oh okay, since accessing the facility for HIV prevention services could you explain how your life has been impacted.

QA016: Mm…

RA: Since accessing this facility for HIV, since coming to this clinic for HIV prevention services could you explain how your life has changed.

QA016: Ah since my life is okay, is going nice everything is going right all the things I saw before taking treatment are gone I`m fresh like the other people there`s nothing wrong.

RA: Oh okay, can you explain the HIV health services you think have been helpful to you.

QA016: Mm this treatment was helpful to me.

RA: Mm…

QA016: It help to not multiply my HIV.

RA: Okay it is time for us to close this part of interview but before we do so is there any part about this topic that we have discussed that you feel it is important to say?

QA016: Mmm…. No.

RA: No everything is okay?

QA016: Mm(yes)

RA: Now we have come to the end of our discussion, if you have any question about the study please you can contact us and thank you for your time.

RA: Time is 15H05. Interview ended thank you.
