# Supplementary material for: Understanding factors influencing utilization of HIV prevention and treatment services among patients and providers in a heterogeneous setting: A qualitative study from South Africa
Source: PLOS Glob Public Health. 2022 Feb 3;2(2):e0000132. doi: 10.1371/journal.pgph.0000132 (PMC10021737; doi:10.1371/journal.pgph.0000132)
Supplement: S1 Data — (ZIP) [file pgph.0000132.s001.zip › Supplementary information/IDI_Clinic attendee_QA017.pdf]

Full participant ID: QA017

Participant Type: Female

Location: XXX (NAME OF CLINIC)

Date:21 July 2020

Primary interview language: English

I: Time is 10:19 participant QA 017, Participant is a female at (XXX Name of a Clinic). Good morning?

P: Morning

I: How are you?

P: I'm good, and you?

I: I am good thanks.

P: Eh My name is (*XXX Name of an Interview*) and I'll be your interviewer for the day.

P: My name is (*XXX Name of a person)*

I: Alright, eh I`ll like to request you to speak out loud so that our recorder here can be ableto record you well.

P: Okay

I: Before we go forward eh… can I, I am asking for your permission to record this interview.

P: No problem

I: Alright, Eh Could you please tell me more about yourself?

P: My name is (*XXX Name of a person*), Yeah, I am 38 years old.

I: Alright

P: Mmmh

I: How old are you?

P: 38 years, 1982.

I: Alright, tell me more more about your education?

P: I finished school at grade 11.

I: Oh Grade 11.

P: Mmmh yes, now I'm working as a cleaner at (XXX Name of a hospital).

I: Alright

P: Mmm yes

I: Alright where are you staying currently?

P: (XXX Name of a Place)

I: Where are you originally from?

P: From (XXX Name of a place)

I: Oh alright thanks

P: Yeah

I: Okay, How are you finding easy with this? COVID19? How do you feel about it?

P: It's not easy, but it's because I'm working at the hospital. Yeah.

I: Oh yes

P: Yeah

I: Alright, how long have you been working there?

P: from 2008.

I: From 2008?

P: Yes.

I: Alright And then how long have you been visiting the clinic?

P: It's been I found out that I am HIV on January. I visit it, its three times from now.

I: Which year? January which year?

P: This year (2020)

I: Okay, did you visit other clinics?

P: No , it's only this one.

I: Alright What do you like about this clinic?

P: Their good service, Everything.

I: Mmm (ok), Which services are you accessing?

P: Side of me, especially about HIV.

I: Alright

P: Yeah. They are good on that.

I: How often do you visit?

P: It's only, now it's three times.

I: Three times a week or three times a month?

P: Eh a month. It was January, May.

I: Okay

P: And now July…

I: July

P: Yes its three. Yah

I: Alright, alright. So what do you dislike about this clinic?

P: There are some people they know how to talk. There are some people they don't know. Especially the one they wait for us on the door.

I: Alright

P: Mmm yes.

I: Is it's only about the way they treat you?

P: Yeah, it's only the way they treat us. Others they treat us right. Others they are not the same.

I: Alright, So what else do you dislike about this clinic except the way they treat you?

P: Nothing for now.

I: Alright.

P: Yeah.

I: You spoke about working in a hospital.

P: Yes.

I: Which job are you doing?

P: I am cleaning.

I: You are cleaning?

P: Yes

I: Alright so how do you… how how safe is it?

P: Its safe because safe because they give us full PPE.

I: Alright

P: Yeah, Every time when you go in we go in seven o'clock. They give us FULL PPE Like nurses, doctors.

I: Alright

P: Yes

I: Are you familiar with the way this environment is in this clinic since you are working in the hospital?

P: Yeah.

I: You are familiar?

P: Yeah.

I: Do you find this clinic safe as the hospital?

P: NO, no.

I: What you think can be done or improved?

P: Our… the shoes we wearing? When you go inside the hospital we have the shoes…

I: Ohh (ok)

P: That we were in on the top of these shoes.

I: Alright,

P: We don't walk with this shoes here. The mask; the head; that Gloves; the apron, everything.

I: Alright.

P: Mmm

I: Its safe.

P: Yeah

I: Alright, so eh. You spoke about accessing ARVs in this clinic.

P: Mmm (Yes)

I: How long have you know your status?

P: From January.

I: From January

P: Yeah, It was, its only 7th month. I think from January.

I: Alright.

P: Yeah because it's only seven months.

I: Yeah, take me through that they How did you get tested? And when did they initiate you?

P: I get tested because I was here to check for what they call it like (lithumba) lumb , I came For HIV tests.

I: So there was a Pimple.

P: yes.

I: Alright. And then after that testing that the after tasting that day?

P: After testing that day they gave me counselling and then they take the blood again and then they gave me the pill.

I: Alright

P: Mmm

I: So how was the counselling?

P: It was good

I: How long did it take?

P: it was this situation, at home I have a brother whos HIV, I Have a sister from another mother (Aunt) whos HIV positive.

I: Alright

P: Mmm (yes).

I: They are Also accessing treatment?

P: Yeah.

I: Same clinic?

P: No, not Here at home.

I: Oh at home.

P: Yeah

I: Alright, Who are you staying with?

P: I'm not saying anyone but I have a boyfriend.

I: Where is he staying at?

P: He is staying at (XXX Name of a place)

I: Alright

P: He is coming sometimes this side.

I: Is he aware of your status?

P: Yes I did tell him. He did went to check after I went to check, then he get it is not positive. He's still negative.

I: Alright

P: Yeah

I: Is he supportive?

P: Yeah, he's Supportive. Until now. I don't know if but he is supportive.

I: Do you have any other factors that are affecting your health?

P: No. It’s Just HIV.

I: Alright, there any other people around you, Except your siblings? Your brothers?

P: No, I don't know about Other people.

I: Can you tell me about your experience in terms of service delivery in this clinic?

P: My experience?

I: Yeah
P: Like, how was it when I come here?
I: Yeah. Even if it can be a story or information about the process, or the procedure that they're using to deliver services?
P: That's why I told you, other people. They talk to us nicely. Others they didn't know how to talk.
I: Alright
P: Yeah, they're not the same maybe today was you will find someone who will talk to you to you nicely. But next time, find someone Why are you here? Why are you late? There is no sister.
I: Alright and then when it comes to accessing your pills…
P: Mmmh
I: Your Treatment, are they always on time.
P: Yeah.
I: It's sufficient?
P: Eight o clock because I'm Knocking out at work like seven o clock. I drink the pills 8 oclock im at home.
I: 8 Oclock evening?
P: Yes, Evening.
I: So do you receive any consulting?
P: Now since I received the consulting here at the clinic yes?
I: You Nolonger receive any counselling?
P: Yes.
I: Alright, I hear you, So what other things that you'd like to improve about this health services?
P: For now I don't know because at the first time I was confused but now I don't know.
I: Alright
P: Yeah now I don't know I: Alright

I: So, what do you understand about HIV prevention?

P: I Understand that it prevent the cells, anything in your body.

I: Alright and then what Do you understand about things that you can use to avoid getting HIV

P: It was only a condom

I: Its only condom?

P: I only know condom.

I: Alright, what happens if you find yourself experiencing a condom bast?

P: I'll come back to the clinic.

I: Have you ever experienced that before?

P: For now? I didn't. I didn't.

I: You Didn’t it?

P: Yes.

I: What were the services that you think they might offer you if you're in such position?

P: I don’t know if I’m HIV they give me something or what I don’t know.

I: Are you using a condom?

P: Yeah.

I: Is it always user friendly?

P: Yeah, since that time I'm using a condom

I: Alright Do you get access to condoms easily?

P: No Because sometimes I take the condom at work if there is condoms.

I: And at the clinic do you have access to condoms?

P: No, I didn’t see condoms.

I: So which condoms Do you prefer Males or Females?

P: Males

I: So here in this clinic, you haven't seen any Condoms?

P: No

I: What to do. Think about that. Do you think what do you think about not seeing them? what comes to your mind? About this place?

P: It means always we must tell them can I have condoms. when you ask something they will shout at you. They will tell you if you don't see there was nothing.

I: Alright, Do you know lubricant ?

P: No

I: it's only the condoms that you know Off?

P: Yeah.

I: So what do you understand about universal testing and treat?

P: Universal test and treat… what is that?

I: It's it's a process whereby when someone is tested positive today,

P: Yeah…

I: They initiate the person Today.

P: I don't understand.

I: Okay. Since now I explained it can you be able to Picture it, testing a person today and when that person is positive?

I: Like me they test us today. And they with with that thing like a pregnancy test?

I: Yes.

P: Yeah, they test us today. They give me the results now. Is that it?

I: Yes

P: Okay.

I: Yeah. And then and then the treatment When did you receive it?

P: Same Day.

I: What do you think about that? Is it good? Oh,…

P: it's good.

I: It's good.

P: Yeah.

I: In which way?

P: You don't waste any time to take a month you don't take your treatment. It's good. To take same time.

I: Yeah. So, what was what are they good at? what good things about ARVs? the advantages of taking ARVs?

P: There are no any. They are good. Like disprings (XXX name of pills).

I: There are no any challenges that you're experiencing when you're taking?

P: Since I take these pills, I didn't have any challenges.

I: Alright, what could happen if you stopped taking ARVs?

P: You default

I: You default?

P: Mmm

I: Take me through what do you mean by default?

P: Like yourself will be weak. Everything will change on your Body.

I: Alright, And then when a person defaults What? Is there anything that can be done to the person?

P: Yeah, sometimes they give them treatment that old treatment, you remember to start afresh.

I: Oh to start afresh.

P: Yeah.

I: And then after that?

P: After that, I don't know but I know they give him that treatment or the old treatment then he can recover or she can have.

I: Alright, and then what are the good things about taking the treatment?

P: To protect Body, Everything. To Protect yourself.
I: How often do you come here for blood collection?
P: Eh For now it’s a second time.
I: How's the service?
P: It was good
I: The results when do you get them?
P: I didn't get the results
I: Alright.
P: So I don't know why but I didn't get it you it was it the time hours? Yeah. Because the time I was here for the results. They told me that the doctor was not here.
I: Alright.
P: Mmm
I: Ok and then since then you haven’t seen a doctor?
P: No.
I: Ok, I understand, how did your life happen to be impacted since you started coming to the clinic?
P: Impacted how?
I: The effects how did it change?
P: Nothing's change.
I: Everything is still the same?
P: Yeah it’s still the same.
I: Alright. the HIV prevention services are they helpful?
P: Yeah. They call you. They remind you about the treatment. Every time they call me even to yesterday they did call.
I: What are they saying when they call you?
P: They just asked you, are you taking the treatment right on time?
I: Who's calling you?

P: I don't know. It’s that sister.

I: Alright, at what time?

P: Yesterday, she call me around 16h00.

I: How do you feel about different cause?

P: I feel, it's great. It's great. They remind you, it's very, it's great.. Just remind me.

I: And then… since you started coming to this clinic, are there any difficulties that you're experiencing between the clinic and your work?

P: No

I: When you come here for a visit, how do it do you take a sick leave or sick leave

P: Yeah, Sick leave

I: They are not complaining?

P: No, they're not complaining.

I And then do you receive any letter or something too?

P: Yeah, they give you a letter.

I: Oh, Ok give it to your boss?.

P: To my manager.

I: How is the situation at home?

P: Its great

I: Its good?

P: Yeah, nothing has changed

I: Ok, Is there any question?

P: For now I am ok.

I: Alright, do you have anything else that you would like to share with us about your experience in this clinic story or something that you witnessed in this clinic?

P: No, for now, it was good for me, especially when I found out from HIV. They treat they treat me nicely. They give me everything Counselling and everything. It was good.

I: And then when you coming to the clinic to collect your treatment how are they dividing you from other people, take me through that process?

P: No, they did not divide. They don't divide people, we go in a line with everyone high blood, everyone.

I: Ok

P: They don't divide like HIV, everyone. And then at the end, at the end, they know that they will call your name and you go to that Sister, you do your thing and go out like that.

I: Alright

P: Yeah.

I: So there is confidentiality.

P: Yeah.

I: Okay.. Is there anything that you think can be done better in, patterning that?

P: They must take..they must have a meeting to treat the people.

some they treat the people nicely, some they don't the people.

I: Oh the way they treat you.

P: Jah.. Jah

I: How often do you see people complain in this clinic?

P: They are complaining every day because when you can come out you see the line here, but when you come in that lady she will shout "we didn't say you must come here at six o'clock, we are knocking in eight o clock is not our problem".. Things like that.. and we come here in the morning to be, you see, but they shout at us. You come late, they shout.

I: So, the working hours in this clinic are they okay?

P: For them.. for us when you come here from six until seven they open eight o'clock they will shout.. Is not okay.

I: What do you think..

P: At least they can open seven o'clock, you see. when we come here six o'clock, seven o'clock we know they are open.

I: Alright. Then before COVID-19 the situation was like this with the queue?

P: No, it wasn't like this.

I: How was the service?

P: It was..It wa.. it was hey I don't know. It was full inside and full outside. There was no line neh, but it was full full full. I don't know.

I: Alright. So did you happen some other day to come here and go home without being helped or assisted?

P: No. No, no. I didn't, no.

I: So normally they help everyone?

P: For me since I'm coming here they help me, I don't know about other people, but they help me.

I: So if it's fully packed as you said, What do think can be done?

P: That's why I said at least, if they can open seven o'clock maybe it will be better. You come here six o'clock since you are here, Seven, eight, nine it is like there's no or what I don't know.

I: So about the lump..lump that you spoke about, how is it since you started the treatment?

P: You know this lump started When? When I was home, I told doctor about this lump, it was in here it was on my vagina on a side. When I was at home, I tested I was okay that time. every year, it's comes, every year. Then one day at work I was working in theater, you know theater? When doctors with their needle, they just put.. Throw it any plastic then you come with the glove, that glove is not the strong like this one for COVID-19 you just put it and take it like that.. when you take it like that it prick you that time, then they take it too casualty, I was okay. This year when I come here, When I have that lump, they say am HIV positive. then I don't understand is that needle or what? But it's okay life goes on.

I: So since then, since you said it kept coming back every year.

P: every year, I don't know since I started this treatment next year it will come back, I don't

know.
I: But now it disappeared?
P: It disappeared.
I: Alright. Okay. What do you understand about the STI and STDs?
P: I don't understand about that.
I: STI stands for sexually transmitted infection.
P: Okay.
I: Then STDs stand for sexual transmitted disease.
P: Okay.
I: You don't know anything about those?
P: Mhmm
I: Okay there are eeehh... diseases or infections that you get when you having sex without using a condom.
P: Okay
I: Yes with a person who is not circumcised or the person who slept with another person
P: Okay.
I: And come to you with that infection. Yeah.
P: Okay.
I: So those are STDs and STI.
P: Okay?
I: Yes.. So, did you have information about STDs and STIs.
P: No.
I: Okay. Take me through this process of HIV. How do you think HIV is transmitted?
P: About the scratch. Maybe you have scratch a blood can connected like that.
When you sleep with someone I don't know how that one.
I: When you sleep with someone...sexually sleeping with someone?

and then what else do you know?

P: Is that.

I: Only those two?

P: Hmm.

I: And then the needle, you spoke about needle?

P: Yes. A needle, that needle was having a blood. that's why they take me to casualty. That time They give me a treatment. It's a treatment from, it was for six months. Yeah. ARV for six months, after that they say I must come back to take.. what you call, a blood again, they take me blood, they give me results, but still now January I find out am HIV positive.

I: how did you feel about it?

P: For me It was shocked. But I just say to myself, it's okay because life goes on. I can't do research, when? Why? You see? it's waste of time, let me take the pill and life goes on.

I: What convinced you or motivated to take the pills?

P: because there's someone who has HIV at home, and then he is still alive and life goes on.

I: When you get time, you should ask one of the nurses about your question that about the consent that you had..you said you don't know how if you sleep with a person you get infected .

P: Okay.

I: You don't know how?

P: Yeah

I: They will elaborate. They'll tell you more about it.

P: Okay no problem. I will do that. I will do that.

I: Do you have any other question?

P: No.

I: alright. If you don't have any other question then do you have anything else that you want to say before we?

P: Am fine. Am happy.

I: Alright. we have come to the end of our interview. Thank you very much.

P: No problem

I: Yeah, if you have any other question about the study or any other questions, feel free to contact us neh.

P: Okay.

I: Yes, I will share the information, the contact details.

P: Okay no problem...

I: Yes. So the time is 10:46
