# Supplementary material for: Understanding factors influencing utilization of HIV prevention and treatment services among patients and providers in a heterogeneous setting: A qualitative study from South Africa
Source: PLOS Glob Public Health. 2022 Feb 3;2(2):e0000132. doi: 10.1371/journal.pgph.0000132 (PMC10021737; doi:10.1371/journal.pgph.0000132)
Supplement: S1 Data — (ZIP) [file pgph.0000132.s001.zip › Supplementary information/IDI_Clinic attendee_QA019.pdf]

1 PARTICIPANT IDENTIFICATION NUMBER: QA019

2 FACILITATOR: XXX (Name of RA)

3

4 CLINIC NAME: XXX (Name of Clinic)

5 TYPE OF PARTICIPANT: MALE CLINIC ATTENDEE

6 LANGUAGE: ENGLISH

7 TIME: 11H05

8 DATE: 21 JULY 2020

8 I: Good morning again.

9 P: Morning morning, how are you?

10 I: Ehh my name is XXX (Name of RA). I would like to thank you for agreeing to take part in this interview today. For purposes of regulation can you please confirm that you allow us to audio record you?

11 P: Yes, I confirm by saying my name, my name is (xxx name of person). *Ehh* I would like to respond in any question that you have.

12 I: Thank you very much Sir. We are interested in knowing about your experiences when accessing services related to HIV intervention services in this clinic which is XXX (Name of Clinic). You do not have to answer questions if you do not want to. The interview will take approximately 45 to 60 minutes. I want to remind you that the information you share is confidential, what you say will not be connected back to you. While the information gathered during this interview will be combined with other interviews no one will know who said it, where it was said or when it was said. There are no right or wrong answers. We are interested in knowing in what you think and your experiences. Please feel free to ask me any question if something is not clear. Do you have any question before we begin?

13 I: No, I will take the one from you.

14 I: Ok thank you. Participant ID is QA019, Participant type is male clinic attendee, ahh the place is XXX (Name of clinic), Interviewer is XXX (Name of RA) and the time now is 11h05. *Ehh* Sir, can you tell me more about yourself?

15 P: *Mhhh* like saying my name, my name is (xxx name of the person) *ehh ehh* I am living here in Alberton in town. Ehh I would like to say that I…I am coming in this clinic just because I am ill.

16 I: *Ohh* ok. So, sir can you tell me *ehh* how old are you?

17 I: I am 45.

18 I: 45, and then please tell me about your education level?

19 P:*Ehh* I finished my matric ’94, year 1994. *Ehh* I had *ehh* short courses I have done. So, at this moment I am not working anywhere.

20 I: Ok can you please share with us what courses, those short courses that you have just mentioned what are those short courses you have done?

21 P: I have done Health and Safety, I have done Fire Fighting, I have done Security Course, I have done *ehh…ehh* Machine Operator by the name of *ehh* CMC (…) machine…

22 I: Ok…

23 P: That is an industrial machine.

24 I: Yah.

25 P: Yes.

26 I: *Ohh* ok, so where are you from originally?

27 P: Originally, I am from XXX (Name of province) *ehh*…

28 I: XXX which is XXX (Name of province)?

29 P: KwaZulu Natal yes.

30 I: Ok.

31 P: *Ehh* XXX (Name of Area)…

32 I: Ok…

33 P: District yah.

34 I: Ok, so where are you staying currently?

35 P: Currently I am staying at XXX (Name of Area).

36 I: At XXX (Name of Area)?
37 P: Yes.

38 I: That is here in XXX (Name of District)?

39 P: Yes, here in XXX (Name of District) yes.

40 I: Ok, are…are you currently working?

41 P: Not working as I have said before.

42 I: *Ohh* you not working. How are you finding things with COVID-19 ? Do you find it easy?

43 P: Talking about…talking about COVID-19 19?

44 I: COVID-19 19 lockdown *ehh* how are things going for you? Are you happy with things? Which are your experiences with COVID-19 19 lockdown?

45 P: *Ehh* I have learnt so many things about that because *ehh* we…I must say that *ehh* it is better to take *ehh* regulation or it is better to…to…to follow instruction…

46 I: Yes…

47 P*: Ehh* when the government or all the people who are instructing you to follow the proce…to follow the protocol of COVID-19 yah.

48 I: Ok, ok but do you…do you find things easy?

49 P: Me I find it very difficult because there is no place to go, there is nowhere to go so you must have to…you must have to be in one place so that you can survive on to that *ehh* pandemic.

50 I*: Ohh* I understand, now let us talk about your clinic visits (Clearing throat). How did you know about this clinic?

51 P: *Ehh* XXX clinic is a clinic that I…I know it very long time ago, since I was staying there by the squattercamp, XXX (Name of Area) Squattercamp, while I was staying there for a very long time. I used to use this clinic, so I am used to this clinic than other…other clinics.

52 I: Oh ok.

53 P: Yah, that is why I am always coming here.

54 I: Oh ok. So, can we talk about what brings you here in this clinic today?

55 P: Sorry?

56 I: What have you come here for today in this clinic?

57 P: Eh I must say that I…I am here for a follow up about my sickness that I have. I was here last on the 22nd June. So now I am back again.

58 I: Ok.

59 P: Yah.

60 I: Yah ok, so do you get good service from the clinics, from the clinic do you get good service?

61 P: As I said before yes, I do get better service from this clinic. That is why I…I recommend it…

62 I: Oh ok.

63 P: The most because when I came in here, I find a good welcome as a patient under them and then they try by their all means to…to respond on what you are suffering from or what you are…what problem you have. Yah they are very…

64 I: Helpful.

65 P: Yah, I had a good attendance and helpful yah.

66 I: Oh. So (sound of moving vehicles at the background) do you think there is…is there anything that the clinic can improve?

67 P: Yah I would say that they had *ehh* like this thing of Corona virus yah. I would say to them they must have to improve by helping the queue marshal. I saw him holding things the way that he…he needs an assistant anyway.

68 I: Ok…

69 P: Yah, she is the only person whom I see she is working very hard. She controls so many *ehh* areas alone, alone. So, like for instance this thing of social distancing, helping people where they are going, helping people how must I help you. Yah there are so many…and screening the staff. Yah she has a pressure on to that.

70 I: So, she does all that by herself?

71 P: By herself at all yah.

72 I: So, our take here is that at least if the clinic can find another person to assist her.

73 P: Another helper to help her yah. That is where I see that the clinic must improve.

74 I: Oh ok…

75 P: Yah, and then inside the clinic I am not sure…I am not sure about what is happening but the occupants pertaining the…the protocol of *ehh* of *ehh* corona virus ehh situation they are holding it by the way it is supposed to be because they are not allowing so many people inside. They had that number and social distancing inside the clinic…inside the clinic that is why there is a queue outside, just because they avoid that.

76 I: Yah.

77 P: They avoid…they avoid the corona virus to attack us.

78 I: So, I hear you saying inside the clinic (Clearing throat) there are no queues, all the protocols of covid-19 are being followed like social distancing and not allowing *ehh* to many people in one place at the same time…

79 P: Yes.

80 I: So how about the queue outside? Do they control the queue? Do you guys have someone who maybe encourages social distancing?

81 P: Yah, I would say that we as patients outside as we are queueing in there, we talk about that. Yah we normally talk about that. We say that lets…let us get the distance. We saw that the helper is alone there then we try to talk about this to avoid the corona virus, what to do…what to…most of the time wha- what must we do so that we can find ourselves helping this disease.

82 I: Ok, so do you think that everyone outside there in the queue are complying with *ehh* with the protocol of social distancing?

83 P: I saw them the most they are wearing the mask.

84 I: Yah…

85 P: I took like as if ever we are not 100% in that social distancing, by wearing a mask we are doing it as it is because it is like when the…when the *ehh* the minister XXX (Name of person), Minister of Transport, said that we need 100% in the taxi you can imagine if we are standing in a queue wearing a mask is very important.

86 I: Yah…

87 P: So, I balance that. I saw that well is like we are sitting in a taxi because in a taxi when it says 100%, we are…we are touching each other in there. So, the most important thing is to wear a mask. So, by answering your question…yes, we are complying in to that *ehh* protocol. Yah.

88 I: Oh, I understand. So how long did you spend…how long do you spend time in this clinic? How long do you maybe spend time?

'89 P: I don't care about time; I care about my life.

90 I: Yah…

91 P: Just because what is happening what we are facing now we are facing the Corona virus. So, the way things are happening is not normal like before. I…I expect that *ehh* the thing will be like the way it is. I will reach there.

92 I: Since you are saying things are not like before, how were things before?

93 P: No, people were just getting, getting inside without *ehh*…were just getting inside and sit in the bench, when the bench are full which mean that you cannot…you cannot stand. You just sit on the bench because the line was going *ehh* fast. So, this time that thing of…I think the nurses they had their own time this thing to sanitize, wearing gloves, removing glove end wearing another glove so that is why I am saying that nowadays and before are not the same.

94 I: Yah. So, were you happy about the time that you spend in the clinic? Are you always happy about the time that you spend in this clinic?

95 P: *Ehh ehh* when i…when I compare or?

96 I: Yah, before and even now. Are you happy with the time?

97 P: Yah, I would say…I would say not every day. Not every day but I am sure they will attend me.

98 I: Ok, ok. (Sighing) I heard you *ehh* I heard you sharing your age with me and you also told us where you come from so the next question will be are you married?

99 P: Yes, I am married,

100 I: Ok, and do you have any children?

101 P: Yes, I have.

102 I: How many are they?

103 P: Five of them.

104 I: Five of them.

105 P: Yah.

106 I: Ok, ok that is nice. So, can you tell me how long have you lived in this area?

107 P: Ekurhuleni?

108 I: Yes, Alberton specifically.

109 P: Alberton as a…as a town?

110 I: Yah.

112 P: *Mhh* so many years I would say. So many years. Eighteen years.

113 I: Eighteen years?

114 P: Yah, from 2002.

115 I: So, you are a citizen here, like you were born in here.

116 P: It is like that yah,

117 I: Ok. (Laughing) so how long have you been visiting this clinic?

118 P: *Ehh* not often, not often. But *ehh* maybe a year twice or three.

119 I: Oh, roughly three years?

120 P: Yah, no I mean to say in a year maybe once or some other years not coming.

121 I: Oh ok, my question is like how many years have you been visiting this clinic? If you can count the years.

122 P: (Thinking) I am not the person who is always sick but if I can count by numbers, I would say five times.

123 I: Five years.

124 P: Yes, five times or eight times because this thing of flu will allow me to come here.

125 I: Ok, so you are not sure about years like how many years.

126 P: Yah I am not sure about years.

127 I: Ok ok, so have you ever visited other clinics in this area?

128 P: In XXX (Name of Area)?

129 I: Yah.

130 P: Or XXX (Name of District)?

131 I: Or XXX (Name of District) or any other clinic other than XXX Clinic.

132 P: Yah I have been in the…*ehh* XXX (Name of Area)…I mean (Finger triggering) *ehh* XXX (Name of Area). I was there in XXX (Name of Area) to test HIV. I have been in *ehh ehh* XXX (Name of Area) yah.

133 I: Ok, so you know a lot of them?

134 P: Yah.

135 I: Ok, so what do you like about this clinic?
136 P: *Ehh* about this clinic?

137 I: Yes.

138 P: Like I said…like I said before their welcome. The way they welcome patient. Yah it is very interesting just because if ever they had…I mean they even advice you what to do' I mean they spend the time with the patient, I must say that'

139 I: Ok.

140 P: That is why I used to come here in XXX (Name of Area) ehh *ehh* XXX (Name of Area)Clinic…

141 I: It is very good.

142 P: For me, I do not know other people.

143 I: Alright, and then what can you say you dislike about this clinic?
144 P What I dislike about this clinic?
145 I: Yes.

146 P: *Mhh* (Thinking) sometimes I used to see as if they are short of staff, yah.

147 I: Ok ok.

148 P: Yah this is what maybe it takes time sometimes we find ourselves sitting more the whole day just because of that I used to see that because they are short of staff.

149 I: Ok, so since you mentioned *ehh* shortage of staff what do you think they must do to improve on that part?

150 P: Yah, to have enough staff and then to have more staff, yah I would say that.

151 I: Ok, thank you. Ok, so now I am *gonna* ask a sensitive question *ehh* can you tell me whether you are HIV infected?

152 P: I am not HIV infected.

153 I: Ok, if I may ask, does sir understand what I mean when asking this question in such manner?

154 P: Say it again.

155 I: Ok, can you share with us…maybe I can put it lightly so that you can understand it. *Ehh* can you share with us you HIV status?

156 P: *Ehh* by saying that I am positive or negative?

157 I: Yes.

158 P: Hey, I make sure every year that I test my status for HIV. It could be 4 times or 5 times a year.

159 I: Yah…

160 P: Even this year I have been in it 3 times if I am not mistaken, 2 or 3 times. I had the last check ehh on the…on *the ehh* what… let me check it for you. On 13th…

161 I: Of June?

162 P: Of July, which means last Monday, yah last Monday.

163 I: Oh, your last check?

164 P: Yah my last check was negative.

165 I: Can you share with us your results for the 13th of July?

166 P: My status?

167 I: Yah your status.

168 P: My status says that I am negative.

169 I: You are negative?

170 P: Yes.

171 I: Ok, that's good news. *Ehh* can you tell me what are major factors affecting your health right now?

172 P: Hmm (…) *Ehh* I would say well even though I am not *ehh* infected, but it is the fear.

173 I: The fear of being infected?

174 P: The fear of being infected by the Corona virus.

175 I: Oh ok.

176 P: That' why I used to screen myself so that I can see my temperature how it is go about, and then keeping myself *ehh* secured. Social distancing, wearing masks, sanitizing, washing hands and not liking to be in the group of people like clusters. *Ehh I* do not want to find myself in that. Yes, I do but I try to protect myself by using that social distancing and wearing mask and sanitizer at the same time.

177 I: Oh ok.

178 P: And then *ehh* what is affecting my health for now, right now is that they said to me it is an eczema.

179 I: Eczema?

179 P: Eczema yah, this is what I am here for.
180 I: Oh ok, just to get medication for it.

181 P: Yah, I feel better than last week as you see me yah.

182 I: Ok. So even last week you were here for the same problem?
183 P: And and on the 22nd I was here.

184 I: For the same problem?

185 P: Yah, it is about a month now. So, I am here to take instruction what to do now because they gave me medication. They said that I must use it and it is finished so I am back to them so that I can get rid of this infection.

186 I: Ok, so do you think these factors, what you just mentioned that you are suffering from, does it affect other people that you know as well

187 P: No no.

188 I: Oh ok.

189 P: The only person who is suffering from this thing…ahh in my family? Or you just saying…

190 I: No, it could be your friends your family or whoever.

191 P: No, it is only me.

192 I: It’s only you?
193 P: Yah it’s only me’

194 I: Ok. Now we going to talk about healthcare in general. Can you tell me your experiences in terms of service delivery from this healthcare facility?
195 P: Service delivery?

196 I: Yes.

197 P: Oh, I will know from today if ever there is something different *ehh* but their delivery yah is super yah. It is very good.

198 I: Oh ok, no complaints about it.

199 P: Yah because I will ask them something like *ehh* like *ehh* prescription. Maybe If ever, they give it to me not give it to me, but I will say the service delivery is good. Yah I will not comment about it.

200 I: So, in other words you are saying whenever you come into this clinic you always *ehh* get what you… you have come for.

201 P: Yah.

202 I: So, what are some of the features, positive features in the facilities that you have visited. So, it means other than this clinic, the other clinics that you have visited what are the…what are some of the positive features? What is good that you have seen in other clinics that you have visited except XXX clinic?
203 P: The positive features?
204 I: Yes.

205 P: Yah, they are average I will not count one by one look *ehh* similar to this one *ehh* yah yah I would say. What I have said to you about this clinic even in other clinic yah some other things you get some other things you will not get. So, I will not which ones. There is some medication for this Eczema that I am suffering from and then they said into that clinic they will not help me they have not got *ehh* they just give me something that I would say they not sure of, because they said to me 'go and try this thing'. They did not say this is the medication for what you are suffering from. They said to me 'go and try this'.

206 I: Oh…

207 P: Which means I turn my back with no hope. That is why I run to XXX (Name of clinic). I get help here they do not say go and try this. They say to me take this medication you will be healed.

208 I: Ok. So, what other things you would like to improve about the health services in your clinic…in this clinic? What do you want to see being improved in this clinic?

209 P: Maybe that is a second question…I mean that is the very same question you asked. I talked about…I talked about employ more maybe employ another staff where it is short, if ever they are short. And then they must have to have *ehh* a helper.

210 I: The assistant.

211 P: Yah.

212 I: Except the shortage of staff and the queue marshal, getting an assistant, is there anything else you can add on that?

213 P: They must place water outside so that the people can drink.

214 I: Yah yah yah.

215 P: As we are having that problem of his I have seen that maybe if ever they can supply those people, I saw last week one patient was wearing a dirty, dirty dirty mask. Maybe if ever, they had those maybe to help those people who are not affording to buy mask. Maybe yah they must contribute that.

216 I: Ahh now we are going to talk about HIV prevention. What do you understand about HIV prevention?

217 P: *Ehh* HIV prevention is very important to everyone, for the sake of the lives of the people. So, people must always use ehh condoms if ever we are to prevent this disease to spread yah.

218 I: Yah, so can you tell me the different types of HIV prevention services? (Huge sound of a moving car)

219 P: Condomise…

220 I: Yah.

221 P*: Ehh* one-night stands, you must avoid *ehh* drinking *ehh* so that the person who might have to find himself having this thing in such a way that he did not know how to…how did he or she get that he must be *ehh* in a positive mind yah.

222 I: Ok, I heard you mentioning *ehh* condom use *ehh* you said one-night stand. Can you elaborate on one-night stand?

223 P *ehh* one-night stand is something that is happening *ehh* unaware. Like for instance you were drunk while you are doing this thing of sleeping with someone, you were drunk. And then you were not holding yourself, you were not holding your feelings. You must have to always think if that you must have a condom before you have *ehh*…

224 I: Oh, simply put you mean one must always avoid having sex under the influence of alcohol without using protection.

225 P: Yes.

226 I: Oh, ok I get you now. So, what are some of the difficulties you may experience in accessing HIV prevention services? What do you think can be a problem when you trying to get HIV prevention services?

227 P: Hey I do not think there is because *ehh* what I look around even inside the clinic if you are getting inside the clinic there is some…there are condoms. There are condoms who are always there to help people not to be infected with HIV. So, it is for everyone. Health and safety are for everyone.

228 I: Alright. Sir if I may ask you, do you use condoms?

229 I: No, I do not use condoms.

230 I: You do not use condoms?

231 P: Yah.

232 I: Why don’t you use condoms?

233 P: Because I am married to one person.

234 I: Ok…

235 P: Yah and then I am focusing on her. That is why I use to always check my status so that I will have to know about myself. So that I can report immediately in the house that if ever I am infected to prevent not *ehh* getting sick yah.

236 I: Ok since you mentioned that you do not use condoms *ehh* I would just like to…I would like to ask you this question, do you know where you can get condoms? I understand that you do not use them, but do you know where you can get them?

237 P: Yes, I know where I get it.

238 I: Yah.

239 P: In the clinic I can get condoms and, in the municipality, ehh places you do get condoms, yes.

240 I: Ok. So, what…what would prevent anyone or you yourself from using condoms?

241 P: What prevent me?
242 I: What would prevent you from using condoms?

243 P: Hey, actually I am not a womanizer I would say. I am not a womanizer, so I am not even thinking of that because I am in the house.

244 I: Ok, it is like you ‘stay indoors’.

245 P: Yah I am ‘indoors’.

246 I: (Laughing) Ahh can you explain what universal test and treat is? I understand *ehh* you shared with me your HIV status, which is negative, but I would like to know if you know anything about universal test and treat.

247 P: No, I do not know nothing about that only what I know is that they just punch (prick) your finger and then they had the kit (testing device) that is next to you and then they pour the blood they sunk the blood in that sort of thing that is transparent glass. And then they pour it into the…

248 I: That device.

249 P: Yes, and then there are two lines that shows…that will determine your status, whether you are positive or negative. If there is one line you are negative. If there are two lines it means that you are positive. This is what I know.

250 I: Ok. So, after there are those two lines which means you are positive, what happens next? What do they do to the person?

251 P: *Ehh* first of all before you do that, they will give a counselling…

252 I: Yes…

253 P: And then after that hey finished about that and then they would ask you whether you have decided to get tested or you *gonna* go. You just give them the authority. Yah they take advice from you and they write it down all what you want. Then you sign then they take you blood test. If you are negative, they tell you you are negative. You see it by yourself they show you. if you are positive, they say if you are positive, they will arrange the medication for you.

254 I: Alright, thank you for sharing such information with us. So has there been any changes to the way *ehh* I think this is irrelevant. Ok ok, now let us move to behavioral change. Since accessing this facility for HIV prevention services can you explain how your life has been impacted?

255 P: Since?

256 I: Since you came in this clinic for HIV prevention services, like you collecting condoms if you are collecting *ehh* can you explain how your life has been impacted?

257 P: For me if I am a person who are not always ‘indoors’ I would say something different. But as I am not the person who use those condoms I am looking after myself.

258 I: Alright I get your point, since you are not using condoms and also you are HIV negative ahh…

259 P: I am always looking after myself.

260 I: I get you. Sir I think we have come to the end of this interview but before we do is there anything about this topic we have left, that we have not discussed that you feel is important to mention or to say. Do you think we have left anything behind that is important for us to touch on?

261 P*: Mhh* I am not just because I have been looking around like I have been in those clinics like XXX (Name of Area) they had that *ehh* testing stations that are on the street. And then yah I saw that the…the HIV and AIDS campaign is doing well for our health around that place, I must say.

262 I: So, it is like you are recommending them for a great service.

263 P: Yah because of the vendors that I used to see.

264 I: Ok ok, is there anything else?

265 P: No.

266 I: Thank you. Now we have come to the end of our discussion. Thank you for your participation, *ehh* if you have any questions about your study participation please contact us. I would like to thank you again.

267 P: Ok thanks.

268 The time now is 11h40.

GLOSSARY

Gonna : going to

Stay indoors : stick to one partner
