# Supplementary material for: Understanding factors influencing utilization of HIV prevention and treatment services among patients and providers in a heterogeneous setting: A qualitative study from South Africa
Source: PLOS Glob Public Health. 2022 Feb 3;2(2):e0000132. doi: 10.1371/journal.pgph.0000132 (PMC10021737; doi:10.1371/journal.pgph.0000132)
Supplement: S1 Data — (ZIP) [file pgph.0000132.s001.zip › Supplementary information/IDI_Clinic attendee_QA021.pdf]

Full participant ID: QA021

Participant Type: Female

Location: XXX (Name of Clinic)

Date: 20 July 2020

Primary interview language: English

Name of facilitator/interviewer: XXX (Name of RA)

I: My name is (xxx name of interviewer), thank you for agreeing to an interview today. For the purpose of regulation, can you please confirm that you allow us to radio (audio) record you.

P: Yes.

I: Audio record, okay yes. Participant type is Female, and then pid is (paper flipping) QA021, participant type (interview done at xxx name of clinic) and then today's date is 20th July 2020.

P: Okay.

I: And then start time is 11h23. Okay before we begin do you have any question you want to ask me?

P: No.

I: Okay, can you please tell me about yourself?

P: Mmh ooh I don't know why ijoo.

I: Okay.

P: Can I explain so I don't know I have done (laughing) ahh I don't know, I don't know to ahh...aah my name is (xxx name of person) and I'm 23 yaa, during this curfew right now .

I: Where are you from?

P: (sigh) (xxx name of place).

I: Okay, are you married?

P: No.

I: Okay, do you have any children?

P: Yaa.

I: How many?

P: One.

I: Okay, can you tell me how long you have have lived in this area?

P: Aah around three years to four four years.

I: Okay, how long have you been visiting this are… uhm this clinic?

P: Since since last year.

I: Okay, so how did you know about it? Who told you about this clinic?

P: Uhm, first time I get hear I found that it’s a clinic which is close to me.

I: Okay.

P: Its close to me.

I: Have you visited other clinics in this area?

P: No.

I: What do you like about this clinic?

P: Iyoh! (sigh) I don’t know, I don’t understand, aah ask. The working type I mean, agh I don’t know. Nothing I could say. (laughing)

I: Okay, and then what do you dislike about this clinic?

P: Uhm, I think anytime when we are here yoh, you take too much time aah I don’t (do not) think (it’s not possible) ahhh…I don’t (do not) know.

I: So, if they take too much time don’t (do not) they and apologize or they just leave you like that or they don’t come and explain anything to you, why they are taking time?

P: Aah no.

I: Could you tell me about, can you tell me whether you are HIV infected or not?

P: *Ah*, I’m not.

I: (Paper flipping) Can you tell me your experience in terms of service delivery from health care facilities?

P: My what?

I: Your experience, about the service deliveries?

P: *Uhm*, I don’t (do not) know, I don’t (do not) know, I don’t (do not) know.(laughing)

I: *Eeh*, what are some of the positive features in this facility that you have visited?

P: Like what?

I: Like maybe when you come to the clinic…

P: *Mhm*(yes).

I: What services do they render normally here?

P: Think *ko… ko baneng* (at the children) is better. Yah yah its better.

I: Okay, I will repeat the question, what are some of the positive features in this facility that you have visited?

P: Family planning *yah*, I have visited family planning.

I: And what are most challenging features in the facility that you have visited?

P: *Aah* I encountered …I don’t (do not) know (laughing) what can I say. At the scale at EPI (Extended Program of Immunisation) the *yah* (laughing)

I: What are the things you would *eeh*, okay, can you tell me about your experience getting HIV care?

P: The what?

I: What are some of the things you would like to improve about health services in your health facility?

P: *Uhm, yoh*! I don’t know.

I: The things that maybe you see are not happening…

P: In this?

I: *Mhm*, maybe that you could like in the future to happen maybe.

P: *Mmh*, the is no labour what what here, never seen labour what do you call (laugh) ((page flipping)) *mmh* (laughing) *yoh* like saying that thing on you, the labour ((page flipping)) ((Quite for 11 seconds)), *uhm* a labour.

I: When you say “labour” you mean like labour ward or?

P: Yah a labour ward.

I: Okay, now we are going to part three of knowledge of HIV prevention neh (isn’t)? What do you understand about HIV prevention?

P: *Uhm, uhm*.

I: Like how do you understand that HIV, HIV how is it prevented or how can you prevent HIV, you can prevent getting HIV?

P: Condomising and what abstain. (laughing)

I: Can you tell me the different types of HIV prevention services?

P: The different…

I: The different types of HIV prevention services?

P: *Uhm*.

I: Different things that people use to prevent HIV?

P: (Sigh), just know condomising.

I: What are some of the difficulties you may experience in accessing HIV prevention services? Other things that can prevent you or that maybe can, that can prevent you from getting HIV?

P: HIV…

I: Yes, the services of HIV.

P: *Uhm*, I don’t (do not) know.

I: Do you use condoms?

P: Yah.

I: Why do you use them?

P: *Aah*, just for protection and I don’t (do not) know. It protects me lots of things.

I: How often do you use them?

P: *Uhm, iyo* (sigh) I don’t (do not) know.

I: Where do you get them?

P: Here at the clinic, sometimes at the clinic.

I: And where (which) other places can you get them beside the clinic? Are there any other places here where you can go and collect condoms?

P: You just buy there is no I don’t (do not) know where you collect, you just buy you don’t (do not) collect.

I: What would prevent you from using condoms? What is it that can stop you from using a condom?

P: *Eeh*, I don’t (do not) know.

I: And what can prevent you from getting condoms?

P: *Aah*, I don’t (do not) know.

I: Can you explain what the universal universal test and treat is? Where, okay you go to the clinic they test you *neh*, after they test you and then they give you treatment. So, can you explain what the what you understand about the universal test and treat.

P: *Uhm, iyo* don't (do not) know, *aah* (laughing).

I: Okay now we are going to part four behavioural change. Since accessing the facilities for HIV prevention services, could you explain how your life has been impacted? How your life has been affected?

P: *Aah*, (whispering) what can I say? I don't (do not) know.

I: Can you please raise up your voice.

P: *Joo*! I don't (do not) know, I don't (do not) know. *Eish, Arg* I don't know *iyo*, what can I say? *Aah*.

I: Can you explain the HIV prevention services you think have been helpful to you?

P: *Mhm, mmmhm* (sigh) like *aah* what, any advice *yoh*, (laughing) I don't know (laughing). *Yo*h, abstaining, condomizing.

I: So how do they help you, when you say you are condomizing, how did it help you from getting HIV?

I: *Yoh*, (laughing) don't (do not) know.

I: It is time for us to close, this part of the interview, but before we do, is there anything else about this topic that we haven't discussed , that you feel is important to say? Anything that you could like to add? (background noise).

P: Something I want to add?

I: Now we have come to the end off our session of our discussion. Thank you for your participation. If you have any questions about……. if you have any questions about your study participation, please contact us neh?

P: Okay.

I: And then thank you for your time and then end time is 11h36. Thank you.
