# Supplementary material for: Understanding factors influencing utilization of HIV prevention and treatment services among patients and providers in a heterogeneous setting: A qualitative study from South Africa
Source: PLOS Glob Public Health. 2022 Feb 3;2(2):e0000132. doi: 10.1371/journal.pgph.0000132 (PMC10021737; doi:10.1371/journal.pgph.0000132)
Supplement: S1 Data — (ZIP) [file pgph.0000132.s001.zip › Supplementary information/IDI_Clinic attendee_QA022.pdf]

Full participant ID: QA022

Participant Type: Female

Location: XXX (Name of clinic)

Date: 20 July 2020

Primary interview language: English

Name of facilitator: XXX (Name of RA)

Name of transcriber: XXX (Name of RA)

I: The time is 11:54 and participant ID is QA022 and we are at (Name of the clinic XXX) and the participant is female and the type of participant is attendee and today’s date is 20 July 2020. My name is (Name of the interviewer) I’ll be conducting the interview. [Flipping page] Ehh thank you for agreeing to be interviewed and ehh for the purpose of re… the regulation can please confirm that you allow us to audio record this session.

P: Yes.

I: And Ehh as we started I explained about what is the study about and then you signed the consent form that you are giving us consent so that we can record this. Ehh mnca we are interested to hear about your experience accessing, providing the health services ehh related to HIV interventions in this clinic (Name of the clinic XXX) mnca ehh you do not have to answer any questions that you do not feel comfortable answering, ehh the interview will take a period of 45 to 60 minutes. Ehh are you ohk with that?

P: Yeah. (yes)

I: Ehh I would like to remind you the information you share is confidential. What you say will not be connected back to you, while the information gathered during the interview will be combined with other interviews, no one will know who said it? when it was said? Or where it was said. Mnca there are no right or wrong answers. We are interested in what you think and your experience. Please feel free to talk and ask me any questions if something is unclear you can ask for clarity. Ehh do you have any questions before we start this session?

P: No ((very softly))

I: Alright, and then can you please try and speak as loudly as you possibly can sinces we have voices on the other side. Ehh can you please tell me about yourself, holistically, if a person asks who you are? Describe yourself holistically.

P: Okay, ai no I should like mention who am I, who ar…who am i?

I: No you can skip if you do not feel comfortable, if you do feel comfortable it is fine.

P: Okay, *kenna (Name and surname of the participant XXX), like ho disciber nna like, the way keleng kateng?* (I am (Name and surname of the participant XXX), and describing me like, the way I am?)

I: Who are you? where do you come from?

P: Like *ketswa* (I'm from) like eish *ketlareng* (what can I say)? Like *ketshwantse ke buwe like lehore ketswa ko kae?* (must I say like where I come from?) and stuff like…

I: Yes everything like if I had to *kere* (say) I want to know about (Name of the participant XXX)…

P: Yah (yes), yah (yes) *ke tshwantse kethome* (I need to start) *like from scratch*

I: Yes.

P: Okay *kenna* (I am (Name of the participant XXX) *ketswa mo* (I am from, (Name of the place XXX) like ahh, *ke kgotse like kesena batswadi* (I grew up like without parents) *so like I can say hore (that)*, I am a strong women, I'm caring, I'm mhhh like *ke* (I'm)… like *kegona* (I can) like *ketlareng ke explaine jwang* (how can I explain) this thing, like *ke etsa ntho hore e etsagale* (I make things happen). Yes *keba k… kebatla ntho hore iyirahale* (I'm k… I want things to happen) like *yairahala* (they happen) *so like ke…* (I'm)…*like ohh eish! Eish! cKeyatshaba* (I'm afraid) hmm (thinking) I'm loving neh, I'm caring, I'm supportive hmm, I'm hard worker mmh yah (yes).

I: And your age.

P: I’m 23

I: Mhm

P: Ahm (thinking) I’m not married, ((laughing)) I’m single, I don’t have kids ahhh (thinking)

I: Do you have siblings?

P: Yah, I do have one brother that I’m staying with now, yah (yes)

I: Okay, ehh can you tell me how long ehh have you lived in this area?

P: Since 1997.

I: Okay and how long have you been visiting this facility?

P: Since last year October.

I: Last year which is 2019.

P: 2019 October.

I: Okay, and ehhh have you visited other clinics in this area?

P: Around (Name of the place XXX)? Yah (yes)

I: (Name of the place XXX).

P: (Name of the clinic XXX), once.

I: Okay, and ehh what do you like about ehh (Name of the clinic XXX)?

P: Like *ba… di nurse tsa mo ke di* (they… the nurses here they are) hardworkers yah (yes)

I: And ehh what do you dislike about this clinic?

P: ((Whispering)) dislike about this clinic, like *hore emisa nako etelle* (to make us wait for long) sometimes.

I: Okay, ehh could you tell me whether you are HIV infected or not.

P: Negative.

I: You are HIV negative.

P: Yes

I: Okay, Ehh can you tell me what are the major factors affecting your health right now?

P: Uhmm (thinking) its High blood and Cholesterole.

I: And then do you think these factors ehh affect other people that you know as well?

P: Yah (yes), my aunt.

I: Okay, [sound of page flipping] can you tell me your experience in terms of services delivery from health care facilities.

P: Hai! No.

I: Uhmm, your own experience with regards to service delivery, from the health care facilities as you said you visited the other clinic and theres… and then how is the service delivery.

P: Mhm But they *keng?* (what)?, they both, like *batshwarana* (treat us) like the w… like *gotshwana* (the same) both ehh *nthweng ena* (kinda like) ehh… *keng* (what) clinics from (Name of the clinic XXX) and (Name of the clinic XXX) *hahona* (the is no) like, I can not tell *hore honale* (that there’s) diffent *kapa jwang* (or what).

I: So how is the treatment?

P: Yah (yes) is good.

I: Ehh… what are some of the positive features in the facility that you have… that you have visited?

P: Pardon.

I: What are some of the positive features in the facility that you have visited, and what are the most challenging features in the facility that you have visited.

P: ((Whispering)) the was challenge, I never came across.

I: Can you tell me about your experience getting HIV care. (silence for six seconds) ehh what are the things?... okay because this one you did mention that you are HIV negative so ehh what are the things you would like to improve about health services in ehh this facility or in your facility?

P: In this…Clinic

I: Yes.

P: Area, like *habaitse bare ka… keng… ka nako eso like tshwantse bare… barefe attention ka nako e barefileng yona* (when they at… what…at a certain time) like (they are suppose to… give us attention at that time that they allocated to us) cause they don't, like *okile wabona* like (can you imagine*) barefe* (they allocate) this, like *nako eso (certain time) but otlotsamaya setso hole late* (you will leave late). *Nako eo setse efitile* (That allocated time would have passed), yes.

I: So punctuality its an issue?

P: Yah (yes)

I: Is there anything else besides punctuality?

P: No.

I: Ehh… mnca (preparing to speak). Now I would like to know about ehh your knowledge of HIV prevention neh, ehh what do you understand about HIV prevention?

P: I can say HIV prevention its when you take your treatment maybe.

I: Okay.

P: You take your ARV's.

I: Is that how you understand it, like when they talk about HIV prevention?

P: Yah (yes)

I: Can you tell me different types of HIV prevention services, do you know of any HIV prevention services?

P: Like medication? ARV's

I: Mhm (yes)

P: Yah (yes)

I: How do they… how…how do they prevent HIV? Like ARV’s how do they prevent ehh HIV?

P: *Booster* (boost) your…your immunion (immune system).

I: Okay. Ehh what are some of the difficulties you may experience in accessing HIV prevention services? Some of the difficulties that you can experience to access the HIV service prevention…HIV prevention services.

P: Skip ((participant whispering))

I: ((laughing)) Pardon

P: Lets skip ((laughing))

I: Okay ((laughing)) ehhm do you use condoms?

P: Yes.

I: And ehh… the reason why do you, what d…, why do you use condoms?

P: To prevent from infections, like STI’s and st…yah (yes) like tipple (TB) yah (yes)

I: And how often do you use condoms?

P: Everytime when i…

I: Everytime when?

P: When I have sex.

I: Okay, and ehh where do you get them from?

P: We buy them from (Name of the store)

I: Okay and what other places can you get them from?

P: Clinics, yah (yes) Hospitals yah (yes).

I: Ehh what would prevent you from using condoms?

P: Not to get disease and stuff and can prevent also like not to have a child.

I: Okay, but what would prevent you from using them? is there anything that can stop you from using condoms?

P: No ((softly particint responds))

I: Okay, and then ehh what would prevent you from getting condoms? What would stop you from getting condoms? Is there anything that can stop you from getting condoms?

P: No ((softly the participant responds))

I: Okay, Can you explain what the Universal Test and Treat is? Have you ever heard of Universal Test and Treat?

P: No.

I: They… they abbreviated, they say UTT.

P: No, I haven't.

I: You've never heard of that. Okay, UTT. Okay, ehh UTT is the new strategy that was introduced which was… it is last year? Year before last? ((unsure)) to… when you get tested, you start ARV's, haven't you heard of that? That if you get tested and you become HIV positive, you no longer have a long waiting period of starting ARV's, they start you…

P: Immediately. ((Talking at the same time))

I: On ARV's yes, so haven't you heard of that?

P: No.

I: Okay. Alright, ehh has there bee… a…, has there been any changes [Page flipping] so we gonna talk about behavioural changes, mnca ehh since accessing the facility for HIV prevention services could you explain how your life has been impacted?

P: Pardon?

I: [laughing] Okay. Since accessing the facilities for HIV prevention services, could you explain, how your life has been impacted? Have you ever accessed the HIV prevention services in this facility?

P: No.

I: Okay, ehh can you explain the HIV prevention services you think have been helpful to you, not in this facility but *wena* (you) as a human being, as a lady, a 23yr old. Can you explain the HIV prevention services that you think has been helpful to you.

P: To cond…co… [laughing] condomize

I: Condomize [laughing] okay, alright ehh, it is time for us to close our session. Ehh the part of this interview we are done but before we do, is there anything else about this topic that we haven't discussed ehh that you feel *gore* (that) ehh we need to talk about or anything that we left out that you think *gore* (that) we should have touched, that you feel its important to say?

P: No.

I: Mnca, alright ehh now we have to come to the end of our discussion. Thank you for participating, if you have any questions about the study participation, please contact us.

P: Okay.

I: Thank you and the end time is 12:09pm
