# Supplementary material for: Understanding factors influencing utilization of HIV prevention and treatment services among patients and providers in a heterogeneous setting: A qualitative study from South Africa
Source: PLOS Glob Public Health. 2022 Feb 3;2(2):e0000132. doi: 10.1371/journal.pgph.0000132 (PMC10021737; doi:10.1371/journal.pgph.0000132)
Supplement: S1 Data — (ZIP) [file pgph.0000132.s001.zip › Supplementary information/IDI_Clinic attendee_QA023.pdf]

PARTICIPANT IDENTIFICATION NUMBER: QA023

RESEARCH ASSISTANT: XXX (NAME OF RA)

CLINIC NAME: XXX (NAME OF CLINIC)

TYPE OF THE PARTICIPANT: Clinic Attendee

DATE: 20 July 2020

LANGUAGE: English

I: My name is (xxx name of interviewer), thank you for agreeing to an interview today. For purpose of regulation, can you please confirm that you allow us to audio record the session?

P: Yes, I do.

P: we are interested to hear about your experiences accessing or providing health services related to HIV interventions in this clinic (xxx name of clinic).

P: I am.

I: You do not have to answer questions if you   do not want to.

P: Yes.

I: This interview will take approximately 45 to 60 minutes. I want to remind you that the information you share is confidential. What you say will not be connected back to you. While the information gathered during this interview will be combined with other interviews, no one will know who said it, when it was said or where it was said. There are no ‘right’ or ‘wrong’ answers. We are interested in what you think and your experiences. Please feel free to ask me any questions if something is unclear. Do you have any questions before we begin?

P: No question.

I: Type of participant is male participant, pid number 0 eeh is QA023, age of participant is 39 years old, is a male, we are at (xxx name of clinic), today’s date is the 20th July 2020. And then start time is 12h26. Can you please tell me more about yourself?

P: Eeh I'm (xxx name of person), I'm from (xxx name of country) and then I'm 39 years old. Eeh I test for HIV positive in 2019 and then I started with treatment eeh 2018 August. So yaa now I'm close to two years taking treatment. [background noise] yes so that's all. [background noise continue], yes I am married to a woman, we have three children's, but we never made children after we got positive, so our children are safe yes.

I: Oh, so in other you are saying you and your wife are both…

P: Yaa we are both HIV and positive, but our children's are they are HIV negative.

I: Can you tell me how long you have lived in this area, (xxx name of place?

P: How?

I: How long you have lived… (interrupted by participant)

P: Twenty years almost I have been here in 2000 January, so is nineteen years to twenty years eeh.

I: How long have you been visiting this clinic?

P: Eeh since this lockdown, I think for months back because I used to take my treatment in (xxx name of country), so yaa I started visiting this clinic four months back lockdown.

I: So, how did you know about this clinic?

P: Eeh nothing bad, eeh mmm yes like I will say I started four months back, when I come they give me treatment and go back things like that, so yaa nothing else.

I: So, you have been visiting this clinic for how long? You come to this clinic for how long?

P: I think this is my eeh eeh this lockdown was my first time yes.

I: Have you visited other clinics in this area?

P: Yes, yeh (xxx name of clinic), (xxx name of clinic) eeh in (xxx name of place). [background noise, people talking at the back].

I: What do you like about this clinic?

P: Eeh about clinic, eeh how I’m I gonna say? Eeh eish   the way they (nurses) help people, they (nurses) are active and yes. Anyway, on my side they give me what I wanted at that time so I’m happy with that.

I: And what do you dislike about this clinic?

P: Aah anyway I didn’t   dislike anything here. So far I’m so happy, so far I’m happy.

I: Okay, before I heard you say that you are HIV infected, is that correct?

P: Yes.

I: And then you said it’s for how long?

P: For almost three years but eeh I started treatment 2018.

I: Ooh you are taking treatment neh?

P: Yes.

I: And then it’s how long   that you have been taking   that treatment?

P: One year eight months almost.

I: Can you tell me what are major factors affecting your health right now?

P: No nothing, I’m here I never get sick to come and get treatment, I just decided to come and start treatment so yes I never feel anything different.

I: So, do you think that   there are other factors that affect other people that you know?

P: Yes I can see people with rash, eeh or getting sick but I can’t say that is because of HIV positive because the are many outside there that can cause that thing but yes that’s why I can’t be sure with that yes.

I: Can you tell me your experiences in terms of service delivery from healthcare facilities?

P: Eeh facility like here in clinic? Eeh for me like I said so far so good for me. Eeh service delivery for me is still good. I never been here for long time so I started for

months back, when I come they give me what I want then I just go back. So, that’s why is good so far.

I: What are some of the positive features in these   facilities that you have visited?

P: Like I didn’t   get well.

I: Must I repeat the question?

P: Yes.

I: What are some of the positive features in these facilities that you have visited?

P: Yes like positive, like like I said the people in this clinic they are our sisters are very active, are lovely and they are for my side they give me what I wanted in time so that’s more positive for me.

I: And what are the most challenging features in these facilities that you have visited? The most challenging one.

P: The most challenges, (sigh) I can’t say that I had challenge, I never had a challenge.

I: Can you tell me about your experience getting HIV care?

P: HIV care, eeh like eish I don’t understand whether.

I: Okay I will repeat the question…

P: Yes.

I: Can you tell me   about your experience getting HIV care?

P: Yes as I said eeh I came here as a temporary just to ask for medication coz (because) (because) coz of this lockdown I couldn’t   to (xxx name of country) and get eeh medication, so eeh I’m I   I   I’m still good ,nothing. I   have   no ifection (infection) nothing if I ask for what I want they give me in time yes yes   just like that.

I: Ooh! So, since lockdown you were were not taking your treatment?

P: Yes since lockdown I’m taking treatment, since for eeh I can say   four… I started during four months four months back. Eeh I came to this clinic and then sister (xxx name of person) give me what I want.

I: Okay, yes since I heard you that you said “since lockdown…’’

P: Yes.

I: You didn’t manage to go to your home…

P: Yes to my home, to my homeland yes.

I: So, you were not taking medication by that time or you you…

P: I’m taking.

I: No, want I want to say is that by that time you were having more medications, or you were not taking any, they were finished or?

P: they were finished yes.

I: So, in other words you were not taking your medication by that time when they were finished, until you came here today?

P: Ye... no no I I have been I have been coming here to this clinic since the lockdown.

I: Ohoo!

P: Yes.

I: What are the things you would like to improve about health services in your health facility?

P: Eeh, its difficult to answer that one, you must eeh eeh think you are well trainete (trained) for your job, eeh I can’t add anything (laughing) you know yes.

I: Okay, now I want to know your knowledge of HIV prevention neh?

P: Yes.

I: Yes, what do you understand about HIV prevention?

P: Mmm (thinking), like wearing condom each levels have sex, eeh when you need to have sex you need to wear a condom yes, things like that.

I: Can you tell me the different types of HIV prevention services?

P: Ei yes eish, let’s say having too many wives or girlfriends eeh, using condom always when you need to have sex and you see like you don’t need to use help somebody if he is blooding (bleeding) and then just like that yes.

I: What are some of the difficulties you may experience [door bang] in accessing HIV prevention services?

P: The difficulties experience, eeh I don’t I don’t (laughing) I don’t understand.

I: Okay, what are some of the difficulties you may come across when you want to access…

P: When I need to access someone…

I: For HIV prevention services, the services for HIV prevention in this clinic?

P: In this eeh eeh clinic.

I: Yes. (door bang)

P: Mmm (thinking), no I never… no I never had something like that. {background noise}

I: Do you use condoms?

P: Yes, yeh.

I: Why do you use them?

P: Mmhm (surprised)?

I: Why do you use them?

P: I just to … eeh just for prevetion coz my wife is HIV positive, I am also HIV positive and then I understand that eeh our blood is not the same, so if we keep eeh eeh how shall I say it? If we keep we having sex without condom, we can maybe eeh end up getting sick things like that.

I: How often do you use them?

P: How?

I: How often do you use the condoms?

P: in fact no I don’t understand, can you explain what’s that.

I: Like how many times do you use them?

P: Once, one condom for one round.

I: Where do you get them from?

P: Sometimes I get them from clinics, sometimes I used to buy if they get finished.

I: Where do you buy them? What are other places where you can get them?

P: I can buy them in a filling station we used to have condom and in the spaza shops they keep condoms so you buy anywhere.

I: What would prevent you from using condoms?

P: treve (preve)…

I: What can stop you from using condoms?

P: Aah, sometimes if I need to have a child, eeh nothing can stop me but so far, we have three children’s then we are fine with children.

I: And what would prevent you from getting condoms?

P: Nothing.

I: Can you explain what the universal test and treat is?

P: Universal test like?

I: Like When you go to the clinic neh?

P: Yes.

I: They do test you after that they give you treatment.

P: Yes.

I: Yes, so I wanted you to explain what universal test and treat is. [paper flipping]

P: Eeh, no I didn’t get well but when I go to clinic eeh eeh I tested positive, ehm I didn’t take treatment at the same time I eeh eeh eeh coz I got po.., I got tested 2017 and then started treatment in 2018, so still eeh almost one year without taking treatment but anyway I was still well, I didn’t get sick I just decided to come back and take a treatment.

I: Oh! By that time when you were testing your CDfo…CD4 count it was high or they just?

P: It was high.

I: Oh!

P: Very high.

I: Okay.

P: Yes.

I: And then what are some of the advantages of universal test and treat and what are some of the disadvantages?

P: Advantage of eeh, the advantage is when you you start the treatment when you are still well, then eeh you will be well for the whole life if you don’t stop treatment, but if you know that you are HIV positive and then you don’t start with treatment then you start when you are sick and then that eeh mmm dangerous because you can end up eeh having TB things like that and then your life will be critical that time.

I: Has there been any changes to the way health information or health services have been delivered since immediate ART, where you take your medication neh, began that have changed the way you look after your own health?

P: No, my has been same, like as I said I didn’t get sick, nothing went wrong in my body or in my health I just decided to come and take my treatment. So, I can see other people having rash things like that, getting more darker you see? But my look still the same.

I: What if mm (thinking) okay, what if any, issues have you experienced that prevented you from accessing or taking HIV I mean ARV’s?

P: What?

I: Issues…

P: Yes.

I: Have you experienced that prevented you from accessing or taking ARV’s antiretroviral?

P: That I experienced eeh?

I: Yes, and that prevented you from taking your medication.

P: No, I was just to take medication eeh each and every night, and then I said no or and then I was feeling like embarasete(embarrassed) (laughing) let me say things like that, so but from there I decided that no eeh I need to take care of my wealth (health), and then yes and then now im not scared even to tell anybody that im HIV and positive things like that yes I’m free, yes so I understand that HIV. When you are HIV positive doesn’t mean you gonna die early or what if you take care of yourself you can still leave longer.

I: What do you think would happen if one continues to take medication or stop taking their ARV’s?

P: Yes, that can be dangerous because like like they say HIV HRV eeh RV (ARV) is for each and every day and you don’t need to stop it, so once you start with it eeh you take it for your whole life, for the rest of your lives eeh yes.

I: Okay, now I want to know about your behavioural change. Since accessing the facility for HIV prevention services, could you explain how your life has been impacted or affected?

P: Hai, since then never had eeh infection, I’m still well as I said, I’m still doing well. No infections, nothing yes.

I: Can you explain the HIV prevention services you think have been helpful to you?

P: For me?

I: Yes.

P: Eeh like to prevent, I think there is no more way to prevent for me to not get HIV pos… because I'm already HIV, HIV and positive you know so but to live longer eeh I can take treatment each and every day and use condoms things like that so that I can live longer.

I: So, you think taking medication is more helpful and using condoms?

P: Yes.

I: Okay, it is time for us to close this part of interview but before we doh (do), before we do, is there anything else about this topic that we haven't discussed, that you feel is important to say?

P: Aah no, I think everything everything is right nothing.

I: Now we have come to the end of our discussion. Thank you for your participation if you have any question about the study you can contact us. Thank you.

P: You are welcome.

I: End time is 12h47.
