# Supplementary material for: Understanding factors influencing utilization of HIV prevention and treatment services among patients and providers in a heterogeneous setting: A qualitative study from South Africa
Source: PLOS Glob Public Health. 2022 Feb 3;2(2):e0000132. doi: 10.1371/journal.pgph.0000132 (PMC10021737; doi:10.1371/journal.pgph.0000132)
Supplement: S1 Data — (ZIP) [file pgph.0000132.s001.zip › Supplementary information/IDI_Clinic attendee_QA024.pdf]

TYPE OT INTERVIEW: QUALITATIVE

DATE: 20 JULY 2020

INTERVIEWEE: CLINIC ATTENDEE

INTERVEIWER: Name of RA

RA: Eh good day my name is XXX (Name of RA) I will be conducting the interview. The participant ID is QA024 a female attendee type of an interview, and then she is 36 years at XXX(Name of Clinic). The todays date is 20 July 2020 and thanks again for taking your time.

QA024: Okay

RA: And as we explained again about the consent form and you agreed to sign it.

QA024: Yes.

RA: And now can I please for the, the purpose for the regulation can you please confirm that you allow us to audio record this session.

QA024: I do.

RA: Alright, and then can you please try and speak louder and clearly cause there is some noise in the background that is going on.

QA024: Alright.

RA: Eh we are interested to hear about your experiences accessing of health or providing health services related to HIV interventions in this clinic at XXX (Name of Clinic) eh you do not have to answer questions if you do not want too. The interview will take approximately 45-60 minutes, eh I want to remind you that the information you share is confidential and what you say will not be connected to you and while the information gathered during this interview will be combined with other interviews. No one will know who said it, when it was said and where it was said.

QA024: Okay.

RA: There are no wrong or wright answers.

QA024: Okay.

RA: We are interested in what in what you think, and your experiences please feel free to ask me any questions or if there something is not clear please feel free to ask so I can clarify for you neh.

QA024: Yes.

RA: Do you have any questions before we begin the interview?

QA024: No.

RA: Alright. The starting time is eh 13H01 eh can you please tell me about yourself. Tell me everything about yourself.

QA024: Everything about myself?

RA: Yes, everything about yourself.

QA024: Okay my name is XXX(Name of participant) I`m a 36-year-old woman, actually mother, mother of 4 eh, I live in XXX( Name of Area). I`m what I can say…. I`m unemployed. I`ve studied, I`ve studied until Matric and didn`t get a chance to continue with my education and yah yah.

RA: Great, and ah can you tell me about how long have you lived in this area?

QA024: About 30 years.

RA: And how long have you been visiting in this clinic?

QA024: Since 2013, that`s 7 years, I think.

RA: Have you visited other clinics in this area?

QA024: No.

RA: Could you tell me whether you are HIV infected?

QA024: I am.

RA: And how long have you known about your HIV status?

QA024: Since 2008.

RA: Are you on any treatment?

QA024: I am.

RA: And for how long have you been on treatment?

QA024: For 12 years now.

RA: Can you tell me what the major issues are affecting your health right now.

QA024: Well, its nothing much because I do take my treatment regular so, I don't have any health problem yah I`m like normal person who is not horrible.

RA: Okay.

QA024: But yah but, I think it's because I`m taking my treatment.

RA: Okay

QA024: Yah, every time.

RA: Okay, do you think these factors like your HIV status does it affect other people, you being HIV positive does it affect other people?

QA024: Does it affect like other people like, like do you mean like in my family or?

RA: Yah like the question was saying, the first one was saying factors affecting your health right now. The second one is saying do you think these factors affecting other people that you know right now.

QA024: No, no. They don`t it doesn`t.

RA: Okay so now we gonna be talking about healthcare just in general nje.

QA024: Okay

RA: Can you tell me your experiences in terms of service delivery from health care facilities.

QA024: Yah, gosh okay in my experience neh, for instance, at first when you have to come to, when its your date to come to the clinic you have to at first okay now we use that system whereby you have to book or they give you a time where you have to come on your appointments but before you could just wake up in the morning and come to the clinic be served and go home quickly. Now this thing that they have is waste of time its not working you come. It`s so horrible because the give you time and say come 8 o`clock they don`t help you at 8 o`clock they help you at 1 or 2, it doesn`t help at all, it doesn`t. The first option which was, whereby where you used to come normally in the morning you woke up you go home early and when you didn`t wake late then you will leave late cause you knew that you late. So, this thing of appointments and what not its not working, the system its not working at all. It`s not.

RA: And when has this system started?

QA024: I think 3 years back.

RA: Okay

QA024: Yah I think in 2017 it`s not working.

RA: What are some of the positive features that you have in this health facility that you have visited?

QA024: Some of?

RA: Positive features in this facility?

QA024: That I have visited?

RA: Yah.

QA024: In this facility?

RA: Yah.

QA024: No, I only come here when I`m here for treatment, I haven`t come for any other services.

RA: Okay, and is there any other positive services that you get?

QA024: Yah, the nurses that you get they are so friendly, they are so friendly they are so good, the people in general they are so nice they are friendly. They are helping, and they are very helpful.

RA: And what are the most challenging features in the facility that you have visited? The challenges or negative as we were talking about the positives.

QA024: Mmm yah the negative is the long ques yah, and we were talking about the negatives or the positive?

RA: No, we were talking about the positive.

QA024: We were talking about the positive before?

RA: Yes

QA024:Ohhh.

RA: No cause you did mention the positive about the staff being nice and helpful and all that, I just want to know about the negative. Yes.

QA024: Oh the negative is the que, long ques and the, the long time that you have to stay before you are helped and yah otherwise if they could just fix that the clinic will run smoothly.

RA: What do you causes the long ques and the long time waiting?

QA024: I think they, they are booking a lot of people in one day that cause, that what causes the delays.

RA: Okay.

QA024 I think they should maybe select a number of people that they know they can handle a day and deal with those numbers not like a whole a bigger number that they know they can`t handle. I think that what causes the delays. Yah.

RA: And then can you tell me about your experience getting HIV care.

QA024: Mmm I don`t have a bad experience at all from the get-go I was, I tested they told me I was positive and then from there it`s been a smooth ride. Yah got my medication been coming to collect my treatment, coming for my blood eh what you call my blood test yah, yah it’s a there`s no problem about that. Yah.

RA: And do they explain to you the procedure…

QA024: Yah they do..

RA: And the bloods that they run on you?

QA024: Yah they do, they do….

RA: And what is your understanding about it if I can ask, I ask you now.

QA024: Okay.

RA: What are you doing with regards to your HIV status and does your bloods work?

QA024: Okay….. Last time I did my bloods was on October and I`m going to say that my viral load has been low for the past 4 years(laughter) So yah, so yah I do my bloods in October and then I take my, my pill, my pill on 2 months bases yah, yah.

RA: What are the things that you would like to improve about the health service in Winnie Mandela?

QA024: Nothing except for the queues(laughter). The ques and the people they book for that day, they mustn`t over book cause that put a lot of strain in them as well and then we and then in our side we will think that they don`t wanna work and we don't see that the problem is that they over book people, on that day.

RA: So, have you ever like experience people saying ukuthi(that) Like this one don`t want to work?

QA024: No, no not at all. No, they do work.

RA: Okay

QA024: Yah.

RA: Now we going to be talking about, eh HIV prevention okay. What do you understand about HIV prevention?

QA024: Ahhh HIV prevention its whereby when you, okay when you test positive then you start taking your treatment. Its, it prevents you, it pro-long your, your life. And like I mean you can stay healthy as long as you….take your pills on time and you live on the bases that they tell you. Yah yes… You live healthier and longer when you take your medicine.

RA: Ehhh..

QA024: Live longer.

RA: Can you tell me types of HIV prevention services.

QA024: HIV prevention services?

RA: Mmm like before you get HIV or what services that are done before getting HIV?

QA024: Before getting HIV. Okay I know that if you use a condom yah you won`t get HIV…

RA: Mmm.

QA024: Tell me the other thing that I don`t know yah.

RA: Ehhh. The ABC(laughter).

QA024: Abstain (laughter) yah yes yah

RA: And what are some of the difficulties you may experience in accessing HIV prevention services? What could be the stumbling blocks or anything that can make it difficult to the access HIV prevention services?

QA024: I don’t think there is, our government it`s doing their best to, I mean they are doing their best for us to get it I mean if, if I think if people are in denial some of the people are in denial about the sickness of themselves so obvious if don`t help yourself who`s gonna help you?

RA: Mmm

QA024: There`s no stumbling block at all! Yah.

RA: Eh….

QA024: You have to take upon yourself that you don’t want to get it then you won`t get it. Yes.

RA: Do you use condoms?

QA024: Yes, I do.

RA: And the reason for using condoms?

QA024: Its because I wanna, get well like re-infected cause my viral load, my viral load is, is undetected yes. I wanna stay like this.

RA: How often do you use condoms?

QA024: Every day.

RA: Thank you. And where do you get, where do you get them from?

QA024: At the clinic, yah.

RA: And what other places you can get them from?

QA024: Mmm, you can get from like the spaza shops whereby the will be put by the box outside there and supermarkets as well, you can buy them if you wanna buy them at the chemist or pharmacies as well.

RA: And what would you prevent, what would prevent you from using condoms?

QA024: If you wanna have a baby.

RA: Okay.

(Laughter)

RA: What would prevent you from getting condoms?

QA024: From getting condoms?

RA: Yes.

QA024: Mmm nothing unless there`s a shortage or they stop making them(laughter). Yah

RA: Can you explain what the universal test and treat is?

QA024: Universal test and treat, I think it`s whereby you go to the clinic for your first test, for your first HIV test and then you find out that you are HIV positive you start the treatment same day. Yah I think yes.

RA: What are some of the advantages of the universal test and treat?

QA024: Ahhh it`s wow, it`s for you to lead a normal healthy life as well, the better, the sooner the better yah unlike to stay in denial and say no I`ll go, I`ll go and find out that you don`t go and 3 years down the line you find out that sick and you end up regretting that why didn`t I go when I find out that I`m HIV positive.

RA: Okay

QA024: Yah

RA: What are some of the disadvantages of the universal test and treat?

QA024: Disadvantages, mmm disadvantage no, there`s none. There`s none, there`s none, there`s none. Yah

RA: Ehhh has there be any, has there been any changes to the way health information or health services have been delivered since immediate ARVs began that have changed the way you look at your own health?

QA024: Yah. Yah there is a change cause before the was a limited information on, on the virus and how to take care of yourself but now there is gosh, there is a lot of information on how to go about at to treat yourself and how to keep yourself healthy yah. Yes.

RA: Can you share that eh, some of the information that you know, or you have accessed.

QA024: Mmm let me see like for instance, I didn`t know that when you, you like, okay I find out when I was pregnant with my first born baby and I didn`t know that for me to have another baby, my viral load has to be low with my, together with my partner. I had my second born baby when my viral load was not and I only find out then that I wasn`t supposed to fall pregnant, I was supposed to come to the clinic and consult first before falling pregnant. And then with my third born my viral load was low, I consulted with my partner and they told us yah we can go ahead yah. There won`t be any dangers yah.

RA: Okay as you have told us that with your second born your viral load was high, how has that affected you or your health or your baby's health?

QA024: Woo nothing happened hey, with God's grace and obviously me taking my treatment nothing happened. I was praying every day that nothing should happen, I was praying that nothing should happen to my baby. He was born and he was negative, and I learned I was so stressed, I learned that I need to consult with them sisters and doctors in the clinic that if I wanna do this now is it safe and all that yah.

RA: Ehhh what if any issues have you experienced that prevented you from accessing or taking ARVs?

QA024: Sorry, sorry your question again?

RA: Okay, what or if there`s any issues have you experienced that prevented you from accessing or taking ARVs? Is there anything that has…

QA024: That prevented me no nothing…

RA: Mmm

QA024: Nothing. Nothing at all no I can`t no there`s nothing at all….

RA: Okay so, you don`t run shortages of treatment here at….

QA024: No.

RA: That has never happened?

QA024: No. Never. Here in XXX (Name of Area) no, I`ve never experience such yah. We always get our medication on time and they always remind you even a week before you have to come and pick up that, I`m just say at you please come and collect, please come and collect and they say at you if you can`t send someone to come and get that for you the one that you trust. So yah.

RA: Okay, and what do you think would happen if one or if one continues to take ARVs or stop taking their medication?

QA024: What would happen to them?

RA: Yes

QA024: That’s not good, that really not good cause you can`t take your medication then you stop and expect that you will live the normal life that you were living before, it`s dangerous. You have to do as they say that you have, its, its for life yah. You have to take it for life otherwise if you, well people taking them and then stopping and then that`s the end of them passing away because they are not taking the medication so its gambling with your life. Its gambling cause they, they have said before that once you start you don’t stop, even skip you have to take it every day.

RA: Okay.

QA024: Yes.

RA: Ehhh now part four it`s about behavioural change.

QA024: Okay.

RA: Since accessing the facility for HIV prevention services could you explain how your life has been impacted.

QA024: Yah it ah….(laughter). Yah its… Okay. Not well it hasn`t changed that much but behaviour wise it has change because then you don`t care you would go out, have drinks like and weekends are for party but now, you can`t drink like what you used to drink you have to do some limitation so yah it`s fine I`m used to it now.

RA: Okay

QA024: Yes.

(Laughter)

RA: Can you explain the HIV prevention services you think have been helpful to you.

QA024: HIV prevention services?

RA: Mmm

QA024: Mmm like mmm you mean like, what do you mean like?

RA: Eh as you are saying eh you can access condoms…

QA024: Yes.

RA: How has that been helpful to you? How has that impacted your life in a positive way?

QA024: Mmm

RA: And ARVs because ARVs at some point are used as a prevention.

QA024: Yah..

RA: For when you are raped, and you are HIV negative they can give you the ARVs as a prevention.

QA024: Yah.

RA: So according to your own way or your own experience, how has that services been helpful to you?

QA024: It`s been helpful cause, I`m still alive today if it wasn`t for the pills I could not be yah and I`m healthy I mean who can say I`m HIV positive.

(Laughter)

QA024: No one and yah thank you for your treatment.

RA: So, it is time for us to close this part of the interview, before we do is there anything else of this topic that we did not discuss that you feel its important to talk about?

QA024: Mmm No on my side I`m fine. Yes, I`m fine. Yah no. Yah I`m good.

RA: Ehhh we have to come to the end of our discussion.

QA024: Okay.

RA: Thank you for participating and then if you have any questions about our study, eh you can please contact us.

QA024: Alright.

RA Thank you for your time again.

QA024 Okay.

RA: And the end time is 13H24
