# Supplementary material for: Understanding factors influencing utilization of HIV prevention and treatment services among patients and providers in a heterogeneous setting: A qualitative study from South Africa
Source: PLOS Glob Public Health. 2022 Feb 3;2(2):e0000132. doi: 10.1371/journal.pgph.0000132 (PMC10021737; doi:10.1371/journal.pgph.0000132)
Supplement: S1 Data — (ZIP) [file pgph.0000132.s001.zip › Supplementary information/IDI_Clinic attendee_QA025.pdf]

1 PARTICIPANT IDENTIFICATION NUMBER: QA025

2 RESEARCH ASSISTANT: XXX (Name of RA)

4 DATE : 21 JULY 2020

5 CLINIC NAME: XXX (Name of clinic)

6 TYPE OF THE PARTICIPANT: MALE

8 LANGUAGE: ENGLISH

9 TIME : 08:45

10 I. Thank you for taking time to participate in our study, It's a qualitative interview, participant's ID is QA025, Location XXX (Name of clinic). Participant type male, primary language English. Date it's the 21 of July 2020. Name of the interviewer is XXX (Name of RA) and start time is 08:45.

11 I. Aah thank you again for participating and for filling in the consent form. I would like the permission of record the interview with audio recorder, aah so do you allow us to record the section?

12 P. Yes i do.

13 I. Thank you.

14 I. Tell me more about yourself?

15 P. My name is (XXX Name of the person, based in XXX (Name of Area) I was born in July in 1980 in XXX (Name of Province). I relocated in XXX(Name of place) when my mother passed away in 2003, fortunately I don't have a father so I was an often and I was raised by my aunt and she couldn't afford us and staff like that. So eventually at the age of seven she decided to chase us financially she can't afford to raised, actually I can say that I was raised by my sister from the age of seven until now. Mmm (yes) I schooled at XXX(Name of place) and then I did my matric there and then I come back to XXX(Name of place) yah (yes) just started my life here again.

16 I. Okay, Aah so can you tell me how long have you lived here in XXX(Name of place)?

17 P. Approximately I can say its let's say six years cos I move to XXX(Name of place) side and I come again, its approximately there five or six years somewhere there.

18 I. Okay, and how long have you being visiting this clinic?

19 P. Mmm (yes) I started visiting last year October somewhere there.

20 I. Okay, and have you visited other clinics in this area?

21 P. Yes, yes.

22 I. Could you tell me whether you are HIV infected?

23 P. Yes I am.

24 I. And what is it that you like about this clinic?

25 P. Mmm (yes) this clinic it's a comfort zone for me as an I deputy person, like man having sex with man. As well as a gay person cos I come here to express myself and like have feeling the way I feel, and talk confidential things which I keep it this side and doesn't go outside. Regarding like explaining my situation to someone which I find it difficult but when I explain my situation as a gay person in individual person like that, which have the same thing with me. I think it's much better for me.

26 I. Okay, and what is it that you dislike about this clinic?

27 P. Unfortunately there is nothing I dislike about this clinic (laughing) cos is the most clinic that I ever visited like previous ones. And the is nothing that I can say I am still new but It's a couple of a year now. I can say I dislike this and this but is the most comfortable.

28 I. Okay, and how is lockdown experience affects you on a personal level?

29 P. Ohh its very very hard, sometimes like you can come to the clinic and then and found out that someone has tested positive for covid-19 and then you have to skip for your day. And they rearrange the other day, so its so so difficult.

30 I. Okay, so is that affects you taking your treatment properly?

31 P. It does but like the is minimum pill that they gave you. If that date arrives and you have to skip and then you just follow the next day but with me never happen but I ask if like that.

32 I. Mmm (yes) and which treatment are you taking?

33 P. Atenoff (Name of pill)

34 I. Paden?

35 P. Is something like Atenoff (Name of a pill).

36 I. And which categories fall under?

37 P. It falls under, I forget.

38 I. Okay what is it for Atenoff?

39 P. Is for HIV prevention.

40 I. Okay HIV prevention?

41 P. Yes

42 I. And for how long have you being taking the treatment?

43 P. Mmm (yes) 22 years from now, its 22 years from now.

44 I. So you are born taking the treatment?

45 P. Yes.

46 I. Okay.

47 I. Can you tell me what are the measure affecting your health right now?

48 P. Mmm (Thinking) I don't think I have the factor affecting my health but sometimes I guess sometimes I struggle to breath. I have shortness of breath and staff like that, which I use not to have before but I think it's normal because I stopped exercising.

49 I. Okay.

50 I. Do you think this factors is affecting other people which you know as well?

51 P. I haven't ask someone status I think is someone private things.

52 I. Okay.

53 I. Are you employed?

54 P. No I am not employed?

55 I. So what do you do to survive?

56 P. Mmm (yes) I am hustler, I just pump cash.

57 I. Okay. (laughing together with the participant).

58 I. So do you mind sharing with me how do you pump that?

59 P. Jah (yes) I can.

60 I. Okay.

61 P. Aah let me just say sometimes I just mobilized (Noise at the background) maybe let's just say maybe I get three people and I get them involved.

62 I. So how long has being this going on.

63 P. I started last year.

64 I. Okay. And then do you do it every day?

65 P. No not every day.

66 I. Okay.

67 P. Yes.

68 I. How many times per week?

69 P. Twice a week.

70 I. Twice a week?

71 P. Yes.

72 I. Okay, and how did you get to know the mobilizing people and what is it called?

73 P. It's called pop in its an organization that they recruit people they just give voucher so they get into everything, so it's like that and I recruit people and they recruit other, and that recruiting you get that 90 rand voucher again. So it's like that.

74 I. Interesting.

75 I. And the can you tell me your experiences of service delivery from health care facilities?

76 P. Mmm (yes) my experiences with health care facility is that amazing experience which I have experience, at the same time is just boosting myself. I am for citing my career. Which I wanted to be a nurse and a counselor at health facility, so me volunteering doing those things is like help me to know about health facility.

77 I. Okay.

78 I. And you said this facility for you it's very comfortable for you and all that, and then you have attended other facilities, and what is the different about this facility and those other facility?

79 P. Mmm (yes) actually let me say on the previously facilities I could like express my feelings, of being guy of being part of SGP part but here when I started sex with man, probably a nurse was a male. There it was a female I could like explain having sex with the male. So I thought like she can judge me. So like that you see judging someone without weather she will be harmful in that way or not. So here I just like okay someone is just like me. I am having such experience like this or I am having this thing and this and this. And how to deal with this and this. I think it's much better for me.

80 I. Okay.

81 I. With regard to treatment how are the facilities are different?

82 P. here I don't spend like more than three hours on the line, in other facility I just like the nurse do his job, and then everything it's just fine.

83 I. Okay, Great.

84 I. What are some of positive features in this facility that you have visited?

85 P. The facility features that I have visited here is that confidentiality is what matter to you I can say that. That what matter to me and everything that I look here I think it's positive, even entertainment that it's here, it's just like home.

86 I. And what are most challenging features in this facility that you have visited?

87 P. I haven't experience that.

88 I. Mmm (yes)

89 P. I haven't .

90 I. You love this clinic?

91 P. I love this clinic.

92 I. Okay , can you tell me your experiences about getting HIV care?

93 P. Mmm (yes) actually I was young neh when they told me that I am HIV positive, I say thank god cos I don't make myself, I am just like okay. It's just like okay yes that was good that was thing that I have experience, I didn't cry maybe let's say I was expecting it cos it's a long journey that I went through being sick and staff. The way I get story is the way that happen to me.

94 I. And how old are you when you find out that you are HIV?

95 P. I was 14 years.

96 I. 14 years?

97 P. Yes.

98 I. So you were just a teenager ?

99 P. Yes

100 I. Okay.

101 I. What are the things you would like to improve in this health facility?

102 P. In this facility its (Noise at the back ground) as we have various needs, what we called things in the box. I think everything is set up, that's why for everyone to come here. I guess they won't be key when come to medication and staff like that. And if they need to take the blood. Jah (yes) you won't take that long.

103 I. Okay, as you mention the mobile clinic do you know the service that they are rendering? Mobile clinic.

104 P. Services that they are 8rendaring in mobile clinic is we test doing HTS in there. And we come this side in the facility to take a prep or medication and collection box.

105 I. And then collection box what is that?

106 P. The collection box is where you put your number phone and ID and if I have the medication there you just take it and live. And you come next month and you do the same thing.

107 I. Okay. And then it is done in this facility?

108 P. Yes.

109 I. Great. And how is it making the I mean the raising time, and what's the positive thing about it?

110 P. I think it has minimize the, I think like we have start have this average number like sitting down like seeing the Doctor. I think that take time cos you have to see them one by one and then. And giving the medication some are different but we have to see them one by one and the are different patient. So that collection box I think it's very easier and not to be on the line it's just like taking the medication. Some of them they just like its mixture of people so they just like take the medication. I think if those are here for medication and they just take the medication it's much easier.

111 I. Wow interesting, so now we are going to talk about HIV prevention.

112 I. What do you understand about HIV prevention?

113 P. Mmm (yes) it's a pill that minimize, let me say that it's a pill that minimized that prevent you to go to the next stage that is AIDS. I guess it's like that.

114 I. Okay.

115 P. And its also protect viral load jah (yes)

116 I. Okay, can you tell me the different type of HIV prevention services?

117 P. Prevention services that I know?

118 I. Yes.

119 P. I know prep, condoms, yah (yes) I know prep and condoms.

120 I. Okay.

121 I. Can you please tell me about prep?

122 P. Prep is the prevention pill that you take after and before you exposed from HIV. And you take every day its more similar to ARTS.

123 I. Okay, meaning ARVS?

124 P. Yes.

125 I. Alright. And where do you get it?

126 P. Mmm (yes) each and every facility have it.

127 I. Okay.

128 P. Because it's for free and prep it's for free, HIV service regarding going to town.

129 I. Okay, and this prep it is given to everyone or the is certain criteria of people that qualify for it?

130 P. I think the is certain criteria of people who qualify for it.

131 I. Okay which criteria is that?

132 P. Which are HIV negative.

133 I. Okay, with regardless of who you are and where you come from as long as you are HIV negative.

134 P. Yes.

135 I. You qualify for it if you want it?

136 P. Yes.

137 I. And how do you take prep for?

138 P. Prep it's a everyday.

139 I. Okay.

140 P. Its protective from getting HIV. It's more similar to HIV prevention pill.

141 I. Okay. And then you mention condoms, you said prep and condoms. So with condoms what is that, that you can tell me?

142 P. Okay let me start about different prep and condom, prep it's a pill and condom it's a condom (laughing) but condom the is high chance of getting HIV while you are having sex with HIV person, but prep when you are having sex with condom so you are still protected. But I am not inversing that if you are taking prep it's just like having sex without a condom. Cos the is disease so a prep just prevent HIV only. When using condom it's much safer. But the is chances that condom may blast or broke.

143 I. Okay.

144 I. And what are some of the difficulties you may experience when accessing HIV prevention services?

145 P. Mmm (thinking) the most difficult thing when you are HIV positive it's when you have new partner, they don't understand when you get the thing as I, I was born with it they think that you get make yourself, they think that you have sex without having protection and it end up like this. It's hard for them to believe you, it's hard. It's very rear let me say half 99% and you get 1% who gonna believe you and say let's carry on and staff like that.

146 I. So for random person is it easy for them to get prep anyway in the clinic? According to your experience or your knowledge?

147 P. I am not sure about other facility but here pop in under Aurum yes you can, but pop in its meant for man only.

148 I. Okay. So hence I was asking for like just for normal person pass by how is how is not doing any voluntary work, or not collection any treatment. Let's take for instance sebongile (xxx Name of the person) your neighbor. She wants prep because she hard you taking about it, so how easy is it for her to access prep?

149 P. I am not sure about that, let's say that in Aurum we believe in pop in actually we believe that. Female are not the one who spread HIV virus. HIV virus is the man that spread HIV virus. So we believe that, that is way we are doing study that is passing in man.

150 I. Okay.

151 P. To get prevention.

152 I. Okay.

153 P. But as you said for XXX (Name of the person) that why we move around the street we treat those people who are HIV negative and HIV positive and then introduce them to prep.

154 I. Okay.

155 P. That is way we do that.

156. I. Okay, the condom what is it that can prevent a person or what is difficult for a person to access condoms?

157 P. Mmm (Thinking) I am to be honest with this question as I do it. First around you can do it with condom but second round you cant.

158 I. Mmm (yes)

159 P. So I think it's up to a person to decide whether to have a condom or not. But it's not easy.

160 I. So when it happens do you actually know the person status?

161 P. No you don't.

162 I. Okay. So you just go with the feeling?

163 P. You just go with the feeling.

164 I. Okay I hear you, and how does that feel?

165 P. For me it’s not good.

166 I. Okay.

167 I. what do you mean when you say it’s not good?

167 P. I mean as I know my status.

168 I. Yes,

169 P. For I am having sex with someone without a condom, without informing the person that I am HIV positive I am putting in a life in risk and in danger to. So I actually prefer condom.

170 I. Okay.

171 P. I wont lie if I do it, sometimes I don’t but my partner she is on prep.

172 I. Okay. So you saying usually the first around is with condom.

173 P. Yes but the second around it not.

174 I. Okay, alright. So I would like to talk about this subject that you just give. It’s very very interesting that you use a condom in a first round and then the second round you do not. And it has being happening with other people before this partner that you have?

175 P. No.

176 I. Okay. Its happening with this particular partner that you have?

177 P.Yes with this one because I stay with him, a week twice or three times a week.

178 I. Okay. Alright and you said she is on prep?

179 P. Yes.

180 I. That means she is HIV negative?

181 P. Yes

182 I. Okay, alright so how and where do you get condoms?

183 P. In the facility they offer them for free.

184 I. Okay, alright so the main reason for using condom what is it?

185 P. Mmm (Thinking) sexual transmitted you know transmitted diseases.

186 I. Okay.

187 P. I can say that.

188 I. And how often do you use them?

189 P. First around yes.

190 I. And usually how many round do you have?

191 P. Three.

192 I. Three so it means that first round it will be condom and next two rounds it will be without condom?

193 P. It depends.

194 I. Depends on?

195 P. If I use it or not.

196 I. And what’s driving to use it? And what’s driving not to use it?

197 I. Okay besides you in particular under this program and attending the clinic here and collection the treatment as an individual, where is the person get condoms?

198 P. Any clinic.

199 I. Any clinic?

200 P. Yes they provides with condoms.

201 I. Okay.

202 I. It is only clinics?

203 P. No. I think even salon they do carry condoms this days and some they cell at pick and pay.

204 I. Okay

205 I. And what will prevent you from using condoms?

206 P. From using condom?

207 I. Mmm (yes)

208 P. From me it prevent me from transmitting HIV. And it prevent me from other sexual diseases. Like gonorrhea and I forget all of this.

209 I. But is the anything that can prevent you from using condoms?

210 P. No.

211 I. Okay.

212 I. And what can prevent you from getting condoms?

213 P. What can prevent me from getting condoms the is nothing. I have them like I have benched of them .

214 I. Okay. So you not gonna run out?

215 P. No, every day when I come here I take one. I just add.

216 I. I hear you.

217 I. Can you explain what the universal Test and treat is?

218 P. Universal test and treat?

219 I. UTT.

220 P. I think its new to me.

221 I. Okay. So you never had of program were by you get to the clinic you get tested and you start the treat without the CD4 Count waiting times and adherence classes.

222 P. No its new to me, and the last time I tested is like a week I think. I tested in June and I started my treatment I think at 7 July.

223 I. Okay. I here you.

224 I. Has the be any changes the way health information or health services have being delivered since ARVS began. That have change the way that you look at your own health?

225 P. Yes it is.

226 I. Okay can you please elaborate?

227 P. When I started the treatment neh, I think I started the treatment for two years and then and they change the location for me and then I was under this (xxx Name of the shop) and from XXX(Name of shop),I changed to (xxx Name of the shop) I fetch my medication there. From there changes in the body I even gain weight I guess. I think from 48kg to 50kg.

228 I. Okay. And then within the health sector , because remember the was a longer waiting period.

229 P. Yes.

230 I. And now people start treatment faster then before, how was that change the lifes of people or the health system?

231 P. I think it change a life of people because years before you were supposed to wait but now no. like the CD4 drops faster. I think it has change a lot of life in people.

232 I. Okay it is a positive changes or negative changes?

233 P. Positive actually.

234 I. Okay in which way?

235 P. Its different than before like before like you have to wait for that period you start your treatment. You just like go with the flow.

236 I. Okay during the waiting period while you are waiting what is it that can happen?

237 P. Is either you default or you become HIV or you become where you are between life and death. I think it's like that.

238 I. Okay, eeeh what if or any issues you may have or experience that may prevent you from accessing or taking the ARVS, are the any experiences that may prevent you from accessing or taking your ARVS?

240 P. No I haven't.

241 I. Okay, so with the lockdown and all that nothing has change?

242 P. I still get my three month medication that is I say I still get.

243 I. Okay, the was a time were the was a shortage of ARVS neh nationally that has never affected you?

244 P. No

245 I. Okay and then, what do you think could happen if one continue to take ARVS or stop taking the medication?

246 P. My advice is stop taking ARVS you can be in a lot of danger because you gonna default and that gonna lead you to AIDS from there we are taking about the matter of life and death. Is either you die.

247 I. I heard you mentioning that your inspiration is XXX (Name of famous HIV activist).

248 P. Yes

249 I. In which way?

250 P. XXX (Name of famous HIV activist) is the one of the kids born with HIV positive and it was I didn't know like I will end up like him and read the story like almost like everyday. I remember in my life orientation book I think around 28 the was this story I like reading and reading and that is when I started

discovering that I am HIV positive. And I say to all my friend what if you have a friend like XXX (Name of famous HIV activist) what gonna say. And XXX (Name of famous HIV activist) my said I will take care of that. And I think like he motivated me, me and XXX (Name of famous HIV activist) are born the same so that is why.

251 I. Okay, so this behavioral change question.

252 P. Okay.

253 I. Since accessing the facility for HIV prevention service, could you explain how your life has being impacted?

254 P. Mmm (thinking) at first I didn't want to tell anyone what is happening and staff like that I just like keep the staff to myself. And people who I was staying with like my Aunt did find out about that and then she talk to me. On my mind I am like why do you want to talk about in the first place you chase me here and now you want to talk about my HIV prevention and staff like that why do you care. I think to get mad at her but still I didn't bother myself like taking about it.

255 I. Okay and the facility and your personal life how was it change? Like you being accessing this particular treatment how was that person affected you as a human being?

256 P. Actually I started my treatment in other facility I think is still the same question.

257 I. Mmm (yes)

258 P. It change me a lot because like it didn't affect me in anything, before I come to this company I think I was little bit pop in. so i knew about it so I did not have affection in that.

259 I. Okay.

260 I. Could you explain the HIV service you think has being helpful to you?

261 P. Aaah (thinking) the the(laughing) the collection box, and the service that they are rendered in here.

262 I. And which services are those?

263 P. The is this thing called outreach I think it's outreach and the box.

264 I. Mmm (yes)

265 P. follow up to the people who are far away who couldn't come and collect their medication I think it's pretty awesome and much better.

266 I. Great.

267 I. Now we have reach the end of the section, it is time for us to close this part of the interview. But before we do, is the anything about the topic we haven't discuss? That you feel it's important to talk about?

268 P. I think we have rep up everything.

269 I. Now we have to come to the end of the discussion and thank you so much for your participation, if you have any question about the study participation please contact us.

270 P. Okay thank you.

271 I. And thank you again

272 P. You are welcome.

273 I. End time is 09:21..

GROSSARY

HIV= HUMAN IMMUNE DEFICIENCY VIRUS

AIDS=ACQUIRED IMMUNODEFICIENCY SYNDROME

PREP=PRE-EXPOSURE PROPHYLAXIS

ARVS=ANTIRETROVIRAL TREATMENT
