# Supplementary material for: Understanding factors influencing utilization of HIV prevention and treatment services among patients and providers in a heterogeneous setting: A qualitative study from South Africa
Source: PLOS Glob Public Health. 2022 Feb 3;2(2):e0000132. doi: 10.1371/journal.pgph.0000132 (PMC10021737; doi:10.1371/journal.pgph.0000132)
Supplement: S1 Data — (ZIP) [file pgph.0000132.s001.zip › Supplementary information/IDI_Clinic attendee_QA026.pdf]

Full participant ID: QA026

Participant Type: Clinic Attendee

Location: XXX (Name of clinic)

Date: 21 July 2020

Language: English

Name of facilitator/interviewer: XXX (Name of RA)

I: Thank you for taking time to participate in our study. Mm where is that thing? For purpose of regulation, can you please confirm that you allow us to audio-record the session?

P: I do allow that.

I: Okay, qualitative interview, participant pid QA026, location (xxx name of clinic), participant type male, primary language English and date is the 21 July 2020, name of the interviewer (xxx name of interviewer), start time is 09h37, can you please tell me more about yourself?

P: Wow that's a bit nice, I'm I'm one of kind guys, sometimes shy, (xxx name of person) is shy is a shy guy, mmm but talkative and I am more like I knew venture to other people because I'm some people find a quite guy more intimidating and more scary so but once you get to meet me you will find me and fell in love.

I: Okay and then how old are you?

P: I'm 26 years old.

I: Okay, where are you from?

P: I'm from (name of place) zone five.

I: Okay, are you married?

P: No, not yet.

I: Okay, so do you have any children?

P: No, not yet at least.

I: Okay, can you please tell me more about your education about your education?

P: Okay, I'm I did my education in (xxx name of province) originally I'm from (xxx name of province), so I did my education in (xxx name of province), I'm I'm I was started at (xxx name of school) formal primary's and then I went to (xxx name of school) Secondary for my senior levels, where I did my grade twelve and currently I was suppose to be studying business marketing management at eeh (xxx name of college) but due to lockdown we stopped.

I: Okay, so are you currently working?

P: Currently I'm unemployed yes.

I: Okay, how are you finding ease with covid-19?

P: Ay, I find it very difficult and challenging because ahh most workplaces are shutting down, and many people who were currently employed are fi… facing themselves with unemployment. So ahh this covid-19 its very challenging to our society.

I: So, it has affected you a lot neh?

P: It has affected not me only but the country as a whole, that for us as youth you know we have been having a struggle of youth unemployment and this covid-19 comes to add on that issue.

I: So, what do you think can be done maybe to help the youth around here or in South Africa?

P: Aahm, what can help youth is that if youth can be serious about life and start things of their own, stop looking for jobs because the are no jobs and start creating jobs, as I'm talking to you right now I have my own company that I'm marketing for, so I sat down and said no you know what I was born with eeh, I was born with having this stat of youth unemployment and now I'm in the youth sector and I have to do something to change that eeh status.

I: So, when did you start this company?

P: My company, I registered my company la…in February this year yes.

I: That’s good, so how did you know about this clinic?

P: I’m I usually come to this clinic, I’m but then I’m I was on prep, I do I participate on prep here at (xxx name of clinic) Clinic and I found it very welcoming so I always spend most of my time here.(background noise)

I: So, since you mentioned prep, can you please tell me more about it, what is it and what it does?

P: Okay Prep is aah when you are going to start Prep, you are going through ei test HIV test first. Then if you are pos…negative they give you a pill that you will take for a period of uhm how long is it? Aah but they give you that pill that will prevent you from getting infected, it doesn’t actually motivate you to go on an unprotected sex but it happens sometimes you go on an unprotected sex that pill will protect you. Uhm if you are found positive then you go for treatment.

I: So, can you tell me how long you have lived in this area?

P: Aah for from 2014 December up to this far.

I: So, how long have you been visiting this clinic?

P: I will say 90% of my life in (xxx name of place) that means from 2014 December, hai no I started visiting here in 2015 then I became a regular.

I: Okay, so what bring you today here at the clinic?

P: The clinic aah what brings me is that aah I I felt like I I just became part of the family in this clinic so whenever I have time I come and say hi hi to people.

I: okay so, have you visited other clinics in this area?

P: In (xxx name of place).

I: So, can you differentiate between the two or the is no different?

P: I I will say aah this clinic with their services provides I will say it’s a mall then with the clinic that I have visited in (xxx name of place) I will say it’s a spaza shop. I don’t know whether you see the difference. The difference is aah the services offered in Winnie Mandela clinics most of them are not in that clinic like there is no (xxx name

of institute) for instance, there there is no dentist for instance, so many services are not there and even its its space is quite smaller.

I: So, I heard you mentioning (xxx name of institute), so what does (xxx name of institute) do here, do they help you with what I want to know?

P: Ooh, (xxx name of institute) is a very friendly institution I will say, uhm it it’s the one that made me feel more welcome here because its staff is very friendly, uhm they help a lot of patient I I have seen them helping other patients with HIV treatments and they don’t go about speaking about status of their clients.

I: Okay, so what do you   like about this clinic?

P: What I like about this clinic is that this clinic has a staff that is always ready to help, always ready to do their job without questioning.

I: And what do you dislike about this clinic?

P: Up to so far, I don’t know what I dislike about it because I found it very.

I: Okay, can you tell me whether you are HIV-infected or not?

P: No I’m not.

I: So, can you tell me what are major factors affecting your health right now? What are major factors?

P: Uhm major factors that are health are affecting my health was uhm ohoo! Well I can say air pollution from outside not from here. Uhm but aah I don’t have those such major factors.

I: Okay.

P: Yes.

I: So, do you think mmm, so do you think the are other factors that affect people that you know?

P: Around the clinic or?

I: Also, in general yes.

P: In general?

I: Yes.

P: In general I will say its air pollution, many people because I heard them complaining about it several times.

I: So how does it affect them?

P: It affect them because some of them uhm TB tre…TB aah patients and they inhale that bad smoke because the are many illegal dumping’s which turn to pollute the air.

I: So, what what do you do with those illegal dumping, do you report them or what happens?

P: Aah I report them and up to a point whereby uhm myself and other members of the community decided to do that we are cleaning them, we took an initiative that we are cleaning illegal dumping areas and then make something on that illegal dumping, so that we prevent the other coming dumping’s.

I: Okay, Something like what so? (participant clears throat)

P: Something like aah we have on this corner uhm we have eeh eeh a smaller nyana park and then we placed the guy there to do icar (a car) wash so the are no dumpings there, we fa… we we have conquered that one and we are still moving to others.

I: Yes okay, can you tell me your experiences in terms of service delivery from healthcare facilities?

P: Uhm its it depends on the clinic but let me concentrate on the clinic that I am currently, aah the service delivery in health its quite impressive and aah the the only challenge that the health staff its finding it might be a challenge from the upper structures but as for them they are always hands on.

I: So, what to you think can be done about that thing?

P: The?

I: That thing that the staff is facing here?

P: Ooh! What can be done I think the upper structure should come visit them and hear their their challenges atleast once a month that can help. [background noise].

I: What are some of the positive features in this, in the facility that you have visited? The positive features in the facility?

P: The … (interrupted by the interviewer)

I: In the facilities that you have visited?

P: Except this only?

I: Any yes.

P: Any okay.

I: Yes.

P: Uhm, the positive features is that uhm we do have isolations in facilities for those patients that are severe ill, uhm we do have uhm emergency rooms that you attend emergency patients then eeh we we do have staff that is really really hands on, want to help uhm we do have management that is really listening to clients. So, I think that is a positive way of uhm meeting halfway with the patients.

I: Alright, so then by isolation what do you mean?

P: Isolation uhm it's a room whereby aah if if I have a contaminating disease, I have to stay in a room that I I won't affect other patients because the are patients with that are with immune system, so if I if the facility has isolation then I it can assure their safety.

I: And what are the most challenging features in the facilities that you have visited? The most challenging ones.

P: In the facility that I visited uhm except this one because this one it has a bigger space, eeh the facility that I have mentioned earlier it's the challenge it's are a space for ambulance they don't have.

I: Here at (xxx name of place)?

P: Not not this one.

I: Oho.

P: Not this one.

I: Okay.

P: They don’t have space for ambulance, like if the patient really need emergency attendance of an ambulance they don’t have the space. They will have to move the staff cars and then the ambulance can enter but hear in (xxx name of clinic) the challenge that the the challenge that I think it can its here its its very populative this clinic it’s a very populative one, aah many clients come here not because its best clinic but because of the services they find here.

I: Ooh okay, so what kind of services do they offer here?

P: Like I said earlier there is a dentist which you don’t find in other clinics, uhm we have Aurum I have unfortunately not seen nearby clinics with (name of institute). Uhm we have aah we have some other some other departments except related to health but in other clinics they don’t have. And then also we have social workers in this facility eeh in other clinics they don’t have, we have counsellor’s who are doing counselling to patients, in other clinics they don’t have that so there are many services offered here that other clinics don’t have.

I: And what are the things you would like to improve about health services in your health facility?

P: Uhm one thing I would like to be improved its ac… li… por…maybe they if they can offer the services even during on weekend because people do need services on weekend.

I: So, in other words at weekend they close?

P: At weekend its closed.

I: Yes okay, okay now we are going to talk about HIV prevention, what do you understand about HIV prevention?

P: What I understand about HIV prevention is that uhm aah you prevent you prevent yourself from getting infected from HIV, uhm and then uhm if you are already exposed you you you pick up the treatment right on time.

I: Can you tell me the differ…the different types of HIV prevention services?

P: Its having eeh protected sex which does not always say your pre… your preventing your prevented because you mild not get HIV through sex, aah its Prep, its aah always mind what you touch, mind what aah who you who you help before and you you check the status of whether the person has cuts and so on.

I: Okay, so can you tell me more about Prep?

P: Prep?

I: Mhm.

P: Aahm! (sigh) Okay aah Prep is a very good thing aah a very good initiative that helps you prevent yourself from getting infected, uhm and it also helps you to know your status because before you, start the the process you go through testing and after you found the result you take action on the result regardless what kind of, aah result are they happy result so but Prep is is preventing from is preventing you from getting infected. Though I am quite not sure whether it protects you from other STI'S but from HIV I'm quite sure.

I: So, do they offer it here?

P: They offer it here.

I: Is it free or?

P: It's are free, you just you just have to have a gut of going and test and accept the result and then you go with the process.

I: Okay, so what are some of the difficulties you may experience in accessing HIV prevention services?

P: Uhm, I think the the the hardest thing in preventing HIV its its in a po… it's in a question of testing [background noise, people laughing], many people don't wanna test because they are afraid of the result, they are afraid of what they might find. So,

it's very difficult to to convince someone to go and test, it need someone who is talkative someone with aah a good uhm a… approach, you just cant come and tell me that I must go and test and then start the Prep, you just have to me why should I start this and that.

I: Do you use condoms?

P: Every now and then.

I: Okay.

P: I have a box of them in my room, no no its not it's not that I I do love sex its sex that loves me. (both laughing)

I: Ooh okay.

P: Yes.

I: So how many partners do you have?

P: One.

I: Okay, and then why do you use condoms? Why?

P: For I I use condom for safety, because like I said earlier on that I'm not sure that aah when I'm on Prep I'm prevented from other STI'S so I'm using it for safety. Eih! my sister safety comes first. (participant laughing)

I: So, how often do you use them?

P: Like how often do I have sex?

I: How often do you use condoms?

P: Uhm okay, I use condom let's say (clearing throat) three times a week.

I: Okay.

P: Don't ask me how many how many per day because you might get you might faint.

I: Can you please tell me how many per day please?

P: Per day?

I: Mhm.

P: It depends on the mood when I’m happy its five.

I: Okay, (laughing with the participant).

P: On a serious note, when I’m happy its happy, when I’m I’m not happy like the is something that is fazing me its three.

I: Yes! Where do you get them?

P: Here at the clinic.

I: Only here or?

P: Sometimes I buy them.

I: okay, what other places can you get them from?

P: Uhm, I do get them in some municipality facilities, I do get them in in where is it? At the shopping centre.

I: Okay.

P: Yes.

I: Mmm (thinking) what would prevent you from using condoms?

P: What would prevent me is when I’m starting to plan a baby with with knowing the status of my partner, health wise.

I: Yes and what would prevent you from getting condoms?

P: Nothing.

I: Can you explain what the universal test and treat is?

P: Sorry.

I: Can you explain what the universal trest mmm (thinking) test and treat is? Universal test and treat is. [background noise, chairs moving and people talking at the back]

P: [background noise continues] Hai its difficult, it’s difficult at school).

I: Sorry.

P: Its difficult at school uhm (laughing) aah I think universal test and (interrupted by interviewer)

I: Treat.

P: Treat its identifying your status and then after testing and got your status you start the treatment.

I: Eeh, what are some of the advantages of universal test and treat?

P: Advanta… I would say most advantages of it uhm it helps a patient to know its his or her status and then eeh start the treatment when you are positive earlier before its too late, because some of patients start their treatment in ICU, so some patient do not start treatment they just die because they are ignorant to their to health. So, that's why I always do my whole health check-up at least three times three times a year.

I: So, what do you think can be done to help those that are in denial?

P: To help those that are in denial you just have to convince them with a facts that okay HIV is there and its real, uhm the are people with HIV who are taking treatment and when they tell you that they are both positive you just say they are lying.

They are not telling the truth, so other people if you can tell them that HIV is there and is real they they just more convincing.

I: Mhm okay, and so…what are some of the disadvantages of universal test and treat? Disadvantages.

P: Disadvantages of them, their disadvantage is is I could say it's the attitude of aah the person you approach to come and test, some are very arrogant some they already know their status and they are in denial and they don't want anyone to talk about their status. They don't want anyone to tell them about HIV because they know their positive.

I: Okay, has there been any changes to the way health information ooh wrong question or health services have been delivered ooh okay this is the wrong question, I made a mistake neh. We are going to behavioural change, since accessing the facilities for

HIV prevention services, could you explain how your life has been impacted or affected?

P: It has not been affected instant it has improved because aah I know my status, thanks to health facility uhm I know if I am tested if I could test positive I know I still have life to live. Because there is a treatment so it it has not affected me in a bad way it just improved my life.

I: Can you explain the HIV prevention services you think have been helpful to you? The HIV prevention services that you think have been helpful to you.

P: Prep.

I: Okay.

P: Its Prep.

I: Only that?

P: Its Prep because if I could say other things they also offer them there.

I: Okay, now is time for us to close this interview neh…

P: Mhm.

I: But before we do is there anything else about this topic that we haven’t discussed that you feel is important to say?

P: Uhm what we didn’t discuss is that uhm have I ever come to test with my partner and yes I have I have tested with my partner, I know his status I know her status, I know my status, we know each other status so yaa we are good.

I: Ooh okay, so you are both negative?

P: Negative.

I: That’s good okay mhm, now we have come to the end of our session discussion. Thank you for your participation. If you have any questions about the study, you can contact us.

P: Okay, thank you.

I: Thank you.

P: Thank you so much.

I: End time is 10h05.
