# Supplementary material for: Understanding factors influencing utilization of HIV prevention and treatment services among patients and providers in a heterogeneous setting: A qualitative study from South Africa
Source: PLOS Glob Public Health. 2022 Feb 3;2(2):e0000132. doi: 10.1371/journal.pgph.0000132 (PMC10021737; doi:10.1371/journal.pgph.0000132)
Supplement: S1 Data — (ZIP) [file pgph.0000132.s001.zip › Supplementary information/IDI_Clinic attendee_QA027.pdf]

Full Participant ID: QA027

Participant Type: Female

Location: XXX (Name of clinic)

Date: 21 July 2020

Primary interview language: English

Name of Facilitator/Interviewer: XXX (Name of RA)

Label Key

I = Interviewer

P = Participant

I: Thank you for taking time to participate in our study, it's a qualitative interview. Participant ID is QA027 and Location is XXX (Name of Clinic). Participant type is female. Primary language is English. Date is 21st July 2020. Name of interviewer XXX (Name of RA). Do you allow us to audio record this interview?

P: Yes

I: Thank you. Err can you please tell me more about yourself?

P: My name is (XXX name of a person) I grew up in XXX (Name of former country) then I came to XXX (Name of current country) in 2016 to work. I've been working here, and everything is fine. I was tested HIV in 2015 in XXX Name of former country) then I was transferred to XXX (Name of clinic) err last year November, 15 November last year, that's when I was transferred to XXX (Name of clinic). I've been receiving my medicine here at XXX (Name of clinic), and everything has been fine.

I: Okay, and how old are you?

P: I am 35 years old

I: Okay, so tell me about your household like you are married, you have kids, do you have siblings?

P: Okay, I have been in marriage but my marriage was broken but I have kids, I have 2 kids and they stay at home in (XXX name of the place), right now I'm single, that's all.

I: Alright, err so with this COVID, how is it treating you, how do you find err how do you, like the situation that you are in and COVID how does it fit together?

P: The situation of this COVID may, to me is like caring and it's making everything going back like condemning work, like communication everything with social, how we are living with people is becoming difficult

I: Okay, you mentioned work, so how is it affecting you with regards to work?

P: As for me, for someone that I have been working for I, since this pande… this COVID started we have been sto… we have stopped went to work. They promised that the company will open in the earlier but as for now I'm just sitting at home doing nothing and I'm finding it

difficult to cater for myself like err money for food, like I find it difficult to pay my rentals and everything like that, so I'm seeing that it is a big problem.

I: So how has it err affected you because I heard you talking about communication, you can't communicate with people as much as you used to, in which way?

P: Like where I am staying, I've got friends, I've got, we used to go to like a female gatherings where we discuss some other things, we used to go to other gatherings like church, I used to go to my friend's place to place or just to enjoy so I can't have enough time like that because we have to stay at home and we have to avoid getting in contact with the many people so it is just affecting me that way

I: Okay, and now, how do you err provide for your financially since you are unemployed how are you coping?

P: As for myself I've been, since I left work, I just had small money when I was working in my account and that, from what I have saved I can only manage to buy food and pay my rent, and I don't know when, it's continuing like this I don't know maybe the, that one I saved will finish, I don't what would I do.

I: Okay

P: Yah (yes)

I: Alright, err so now can you tell me how long have you lived in XXX (Name of Area)?

P: I started living in XXX (Name of Area) in, it is almost 3 years now because I started living in XXX (Name of Area) 2017 yah (yes)

I: And how do you find XXX (Name of Area)?

P: Err for me has no stress, I've been staying well and living in good conditions

I: What do you mean when you say good condition?

P: Where I'm staying err, the atmosphere, everything, people they way we socialise I find everything good for me. The environment I find is good for me, like water everything at the facility, electricity is fine where I'm staying so I find everything fine for me

I: Okay

P: Yah (yes)

I: And how long have you been visiting this clinic?

P: I've been visiting this clinic now is like 6 months yah (yes) I've been visiting XXX (Name of Clinic), is almost 6 months now

I: Okay you said November so it would be 7 months

P: Yah (yes)

I: Oh 7 to 8 months because it's on the 15th

P: Yah (yes)

I: Okay, err have you visited other clinics in this area, in XXX (Name of Area) before you came to XXX (Name of Clinic), have you ever went to any other clinic?

P: I've never went to any other clinic. I started coming to XXX (Name of Clinic)

I: Okay, and what do you like about this clinic?

P: I can say about this clinic I like the way they; I can say the way they work with us like patients. We… I don't know, I can say (laughs) but I can see it's a proper because since I came, I came in time they handled everything like in a kind way I've never been harassed, I've never been treated bad. I can get my medication, I can speak to any nurse and they can serve me what I want I can ask for, they can help me. I find it, I find that I'm welcome and I find that err the environment here is fine. There's water, there's toilet, I find everything fine for me.

I: Okay, and what do you dislike about this clinic?

P: Err since I came here, I can't say I have anything which I disliked as for now, I never see anything which I dislike about XXX (Name of Clinic)

I: Err are you on any treatment?

P: Yah (yes) I'm getting err I'm on ARVs

I: Is it the only treatment that you are taking?

P: Yes

I: Okay, and err is for how long, how long have been taking?

P: I've been taking ARVs from 2015, November 2015

I: Can you tell me what are major factors affecting your health right now?

P: As foe now I'm feeling that I'm healthy. I don't have any problems

I: Okay so do you think as err you are infected with HIV err do you… are any other people infected or affected by you being HIV?

P: Sorry?

I: As you are infected with HIV, do you think there are other people that are affected by you being infected with HIV? Like for instance you kids, how are they affected by you?

P: Okay, for me, yes I discovered that my son, my last born was affected that's when I discovered that I'm HIV positive, so my son got affected through me during labour or breastfeeding, and also I don't know about the father because that's the time when we were, we broke up but for him he was also affected so it's me and my son and the father of my kids. I think these are only people who have been affected

I: Okay, and how old is your son?

P: My son now is 10 years old

I: Okay

P: Yes

I: And is he on treatment?

P: Yes, he's getting treatment. He is staying at home, but he is getting treatment that side

I: Okay

P: Yah

I: How is it treating him?

P: As for now, he is, everything is going well

I: Okay

P: Yah (yes)

I: And who takes care of your son at home?

P: My son is staying with my mother

I: Okay, so can you tell me in your experience in terms of service delivery from health care facilities? How is the service delivery in health care facilities not this one in particular, you can also mention about XXX (Name of Clinic), how is their service delivery and if you have any other experience like you said you were taking treatment in XXX (Name of former country) before so you can like tell me about both clinics?

P: Err since I started taking my medication in XXX (Name of former country), that clinic, everything was going well, and their services were fine. Same here when I come to XXX (Name of clinic), I never faced any challenge. The services were fine, I've been getting my medication, and everything was going well.

I: Okay, what are some of the positive features in these facilities that you have visited? Anything positive or anything good about the facilities that you have visited?

P: Yah (yes) I like the environment here and the services are being like I said the way we are, we are getting our treatment yah (yes)

I: Okay, and err what are the most challenging features in these facilities that you have visited?

P: I don't have any

I: There are no challenges?

P: Yah (yes)

I: Okay, err so what time did you get here in the morning?

P: In the morning I arrived at 7

I: At 7?

P: Yah (yes)

I: And what time is it now?

P: Now is…

I: So is around 12

P: Around 12 yah (yes)

I: So, from 7 to 12, you've been here for 5 hours

P: Yes

I: So, it is not a problem for you?

P: Yah (yes) as for today I understand the situation because the sisters, they explained that they are having some problems like during this period of corona they have to take some measures, they have to take some like err they have to take time with the patient trying to make things so I just understand that it is only for this period because sometimes when I come here I’ve never been here for 5 hours but as for today I did understand because they were trying to make things like clear. As for my side they said they, they were talking about treatment what, what problems shortages. So, they were trying to, they were taking time with the patient trying to explain how you can manage so for my side I understand that there's a slight inconvenient, so I think is fine

I: Alright, so I heard you talking about shortages of treatment and all that, can you just tell me what was actually said?

P: For me I think, they just said err we have shortage of treatment but anytime form now you can come back to check so I don’t know, they didn’t explain what the cause of the problem was

I: Okay,

P: Yah (yes)

I: And then the management, how do you manage because you said they also told you about how to manage with the shortage of treatment, how will you manage?

P: Err as for me I can say I think, as for me I’ve got enough treatment for this month but I can come to check then if I can’t get any other treatment from there maybe I don’t know what I can do. (laughs)

I: So, did you receive treatment today?

P: Today I didn’t receive any treatment, they said they don’t have any medicine, any medication today but they said I have to come and check earlier or maybe they will send an email if there's medication

I: Okay

P: Yah

I: So, don’t you think that is a challenge?

P: Yah (yes) I can say it’s a challenge because me as a patient I know that err I have to take my medicine everyday so if I can’t take my medicine every day I see that it is going to affect me, it’s going to affect my health so I can see it as a challenge

I: And then can you tell me err about your experience getting HIV care?

P: Err at first it was scary, like I was, I was not, I was scared at first I received the, when they said I was HIV, at first I was scared but as time goes on I was getting used and the time I started using my medicine I started seeing my health changing, I was getting healthier. When I get tested the blood was, my body and my CD4, everything was going well. I discovered that my health even other diseases like flu and headache and the normal pain, I never, I never had them like I used before started using treatment and I think everything is going well and as for now I got confidence and I’m not scared anymore of staying with HIV. Only what I, only what I, I, I want just to maintain, and I don’t want to spread it to other people yah (yes)

I: Err what are the things you would like to improve about the health services in your health facility? Is there anything that you think should be improved?

P: I don’t know, I don’t think

I: Okay, let’s say what if you were owning this clinic how would like it to be like?

P: Okay, for me I think I would wanted like the, to add some help to people with chronic diseases and disable people like they can have a something, some benefits from the society like food, like clothes and something like that, that’s my opinion.

I: Okay

P: Yah (yes)

I: Alright, err now we are going to talk about HIV prevention

P: Okay

I: What do you understand about HIV prevention?

P: About HIV prevention, I understand err when you are HIV positive you have to make sure that you use the, like precautions like when you are having your partner you have to condomise or to protect, to prevent the spread of HIV then like when you are staying with other people or like when you are using other utensils like needles and you have to be careful with those utensils to prevent the spread of HIV to other people

I: Okay, and err can you tell me the different types of HIV prevention services?

P: Err, HIV prevention like, services?

I: Services yah (yes) what are the services that are being offered to prevent HIV?

P: I’m no sure (giggles), I’m not sure

I: Okay, what are some the difficulties you may experience in accessing HIV prevention services? Like the services that err the clinic is giving, what is it that you can use to prevent HIV?

P: Okay, before you get HIV

I: Yes before you get HIV, because you need to prevent it so that it doesn’t get to you, so what are those services or what are the things that can be done so that a person cannot get HIV?

P: Like err the things that, I said condomising and like abstaining. Abstaining is another fact

I: Is there any medication that you can take to prevent HIV?

P: I don’t know any medicine for preventing HIV

I: Okay

P: Yes

I: Alright, err do you use condoms?

P: I do, I use condoms

I: Okay, alright, and err why do you use condoms?

P: I use condoms in order to prevent a spread, conducting the disease to other people and also to avoid the, myself from getting infected with more viruses

I: Okay, and how often do you use condoms?

P: I use condom every time I get sexual intercourse

I: And where do you get condoms from?

P: Me, I get condoms when I come here at the clinic, I get condoms. There are free condoms here, we get condoms here from the clinic. Then also err at the location, in the public areas there's a, like those people who test people in the streets sometimes they have condoms, they provide, they can give us condoms

I: Okay, and err what would prevent you using condoms? What can stop you or prevent you from using condoms, is there anything that can prevent you from using condoms?

P: As for me I don't think have anything that can prevent me from using condoms

I: Okay, is there anything that can prevent you from getting condoms?

P: Err no

I: Err can you explain what the Universal Test and Treat is?

P: Test and Treat?

I: Yes, Universal Test and Treat

P: I'm not sure

I: You are not sure. Err has there been any changes to the way health information or health services have been delivered since immediate ARV began that have changed the way you look at your own health?

P: As for now I don't think any changes that I have seen which has affected or which has changed my health but I don't know, since today they said there's no medication I what will happen from there but as for now I think everything is fine, so I don't know from now

I: Okay, let me explain the Universal Test and Treat, it is called UTT. It is a system that was introduced by the government in 2018 that when you come and get tested for HIV, and you are found to be HIV positive err you start treatment if you feel that you are ready to start treatment unlike at first you had to wait, they have to take the CD4 count then the result of CD4 count come that is when you will do the other blood, baseline and then you start the treatment. So now when you get tested and you become HIV positive, they take the baseline blood, but they will start you on treatment

P: Okay

I: Yah (yes) so that is what we call UTT, Universal Test and Treat. So, you no longer have to wait

P: Okay

I: They are taking away the waiting period. So now with the experience of you when you got test, did you have to wait for a certain period before you could start treatment?

P: Yah as for me when I tested for HIV, they tested my CD4 count and it was above 500 and they, in Zimbabwe they gave me (not audible 28: 06) they said it will help me to, err just to

help me prevent getting affected with diseases like flue, err like running stomach, diarrhoea and like that. Then when I, after that I was sick for a while like I take (28:44 name of medication), it's almost for one year then after that I was sick then I went to the hospital again, then they tested my CD4 and my CD4 was below 300 copies then they introduced me to ARVs. That's when I started

I: Okay, so how do you think it's different from you than a person who comes now, get tested and then take ARVs, where else you waited for certain period of time and then up until you started not feeling okay? Do you think then versus now err how is the system, is it better and how can a person benefit if they give them treatment sooner than to wait?

P: Yah (yes) as for now I can see that is better to, is better and is going to help to get a treatment sooner because when you get your treatment sooner and you take it err well, you can, your chance of getting sick or your health deteriorating is going to be less

I: Okay, err what are some of the advantages of UTT, Universal Test and Treat?

P: Advantages?

I: Yes

P: Err advantages is that err, the advantages that you can get treated early and you can stay healthy that's all I can say

I: Okay, and err what are some of the disadvantages of UTT?

P: Err as for me I don't think there's a disadvantage (laughing)

I: Okay, alright err has there been on, you have answered this, the negative changes on ARVs. And then what, if there are any issues you have experienced that prevents you from accessing or taking ARVs? Are there any issues that you have experienced that preventing you from accessing or taking ARVs?

P: No

I: The situation of today

P: Oh the situation of today, yes err I can say it will affect me from taking my ARVs if they didn't, if I didn't get it in time since they promised that we have to check, I don't know how long it will take, so if it is going to take time without getting ARVs is going to affect

I: Okay, Err, what do you think would happen if one continues to take ARVs?

P: I think if you continue to take ARVs you will stay healthy and then you, your, your, what can I say…

I: Immune system

P: Yah (yes), your system will remain healthy for a long time

I: And then err what do you think would happen if one stops taking their medication?

P: According to the knowledge I have I that if someone stop taking the medication, he can die, or the health will go down actually the person will die

I: Aright, with behavioural changes, since accessing the facility for HIV prevention services could you explain how your life has been impacted by accessing HIV prevention services?

P: Err, I'm not sure

I: So, you have been attending the clinic?

P: Yes

I: Has that impacted your life in a negative way or in a positive way? Is there any good from accessing the services in this clinic for your treatment?

P: Okay, for me it’s, I can say it’s good for me

I: In which way?

P: Like since I have started taking my medication I can feel that my health is improving from the time I got tested and I’m getting well, I’m gaining weight and my health is improving, and I’m just feeling that it’s fine for me

I: Okay, can you explain the HIV prevention services you think have been helpful to you, what are the things that the service has been doing that you think it has been helpful to you?

P: I think getting my medication and also getting err, checking for CD4 and a viral load, it keeps me knowing, it keeps me knowing an updated that how healthy I am and it makes me, my mind and my, me feel good and I think that one it can help me on my health to feel that the disease is, the virus is getting (no audible 36:01) in my body. It makes me feel happy and my mind is happy, and is helping my health and also about the… that’s all I can say

I: Okay, err it is time for us to close this part of the interview but before we do, is there anything else err about this topic that we haven’t discussed that you feel that is important for us to talk about?

P: No

I: Okay, now we have to come to an end of our discussion. Thank you for your participation. If you have any question about the study participation, please contact us.

P: Mmm okay

I: Thank you

Time ended: 12:39
