# Supplementary material for: Understanding factors influencing utilization of HIV prevention and treatment services among patients and providers in a heterogeneous setting: A qualitative study from South Africa
Source: PLOS Glob Public Health. 2022 Feb 3;2(2):e0000132. doi: 10.1371/journal.pgph.0000132 (PMC10021737; doi:10.1371/journal.pgph.0000132)
Supplement: S1 Data — (ZIP) [file pgph.0000132.s001.zip › Supplementary information/IDI_Clinic attendee_QA028.pdf]

PARTICIPANT IDENTIFICATION NUMBER: QA028

RESEARCH ASSISTANT: XXX (Name of RA)

DATE: 21 July 2020

CLINIC NAME: XXX (Name of clinic)

TYPE OF THE PARTICIPANT: Clinic Attendee

LANGUAGE: English

I: Okay thank you for taking time to participate in our study.

P: Yes

I: Do you allow us to record you?

P: (participant laughing) yes, I do.

I: Qualitative Interview, participant pid QA028, location (xxx name of clinic), participant type female, primary language English, date 21 July 2020, name of interviewer (xxx name of interviewer). Can you please tell me about yourself?

P:I’m (xxx name of person) , I’m from (xxx name of place and province), I’m a cool guy and I’m shy normally, so the only thing I will say ((participant laughing)) so I will listen to you guys when they (referring to the interviewer) gonna ask me some questions and I will follow the rules. So, the only thing I will say I don’t have much words yes.

I: How old are you?

P: 31 (years old).

I: Okay, can you tell me about your educational level?

P: Educational level, I didn’t complete matric, I failed matric.

I: Where are you staying currently?

P: Currently, for now?

I: Yaa.

P: I’m staying at (xxx name of place) but normally at (xxx name of place) zone one.

I: Okay, are you currently working?

P: Yaa, I’m working at (xxx name of company).

I: Okay, so how did you know about this clinic?

P: I know this clinic because I come to take treatment for high blood.

I: Okay, so today you came here to collect your…?

P: To collect yes.

I: Okay, did you get good service from the clinic?

P: Yes.

I: Mhm, okay, can you tell me how long you have lived in this area?

I: how long have you…?

P: How long I have lived here?

I: Yes.

P: I think 2010.

I: yes, how long have you been visiting this clinic?

P: I think mmm (thinking) 2019 because on 2019 I was pregnant and I was come and they assist me here.

I: Okay, have you visited other clinics in this area?

P: No it’s only this one.

I: okay and what do you like about this clinic?

P: It’s a service normally.

I: So today how how long did you spend in the clinic today?

P: Today?

I: Yes.

P: Its been four to five hours.

I: Yes, then is there anything that maybe the clinic can to do improve the services or?

P: To improve?

I: yes.

P: Yes, maybe they change the container to build the more houses, because this container is cold normally and we are are sick.

I: And how was the service, how was the service like, the service that they gave to you today, how was it?

P: No, it was good, they didn't even give me attitude.

I: Yes, and then is there anything that you dislike about this clinic?

P: The thing that I like?

I: Dislike, that you don't like.

I: Don't like?

I: Yes.

P: Eeh Its attitude, some other people other I don't like normally the old person they are suppose to talk nicely Yes.

I: The staff or patients?

P: No the staff they are suppose to tel…they are suppose to talk nicely with the with the patient.

I: Okay, can you tell me whether you are HIV-infected?

P: No I'm negative.

I: Can you tell me what are major factors affecting your health right now? The things that affect your life right now.

P: Which one? To me? For now I’m not effected is only high bloods pressure.

I: So do you think this factors affect other people that you know?

P: This one? Normally I think its HIV, normally people are affected with HIV, TB yes.

I: So, do you that, let’s say that High Blood also affect other people?

P: High blood?

I: Yes.

P: Iyoo!! I don’t   know   that they are affecting other people and besides its normally HIV, TB because we are communicating each other yes.

I: Okay so then when you mean’t   that the mmm(thinking)   the staff don’t talk nicely then what happened?

P: They end up arguing, it’s the way that we see.

I: They argue with the….the…

P: With the older when they come to take treatment.

I: So...

P: Sometimes when you find out you didn’’t   get   your   file example…

I: Yes.

P: When you go there, he he   didn’t told you to the older people nicely yes   they shouting people and that thing is not nice, even you, you won’t feel right.

I: (silence for a little bit and people whispering), does it usually happen or?

P: Sorry.

I: Does it usually happen were they argue with the other with the patient? Do it normally happen or?

P: Sometimes.

I: It happen sometimes?

P: Yes.

I: Okay, can you tell me your experience in terms of service delivery from this healthcare facility?

P: Yes, repeat again.

I: Can you…can you tell me your experience…

P: Experience.

I: Yes, in terms of service delivery from this healthcare facility?

P: Service delivery is good yes.

I: What are some of the positive features in the facility that you have visited?

P: (Thinking), I don’t understand the question, (phone vibrating) you beat me.

I: Ooh so you said this is the only clinic you have visited or there are other clinics?

P: No is only this one.

I: This one only?

P: Yes.

I: Okay, (silence for a little bit) okay according to you in this clinic…

P: Yes.

I: What are things that you think are suitable or right for you in this clinic?

P: Yes.

I: Yes.

P: The things that are good for me in this clinic is that every time, when I come to the clinic, I always get my treatment, there is no day that they said there is no treatment.

I: And what are the most challenging features in the facility that you have visited? Things that are more difficult or most challenging to you since you came here?

P: The challenges that I encounter here like maybe I will say its time, they don’t work on time, they don’t start on time, yes those are the challenges that bothers me. Coz (because) if you can look at the time they start at until now, when I leave the clinic you can see its not the right thing.

I: So how much time do you normally spend at the clinic? You come what time and you leave what time?

P: They start at 07h30, normally I can’t say its 07h00, I can say 08h00. Then I will leave at 13h00 or 12h00).

I: Yes.

P: Yes.

I: So Maybe its not or do you think maybe the is shortage of staff or maybe they are just lazy to do their job?

P: If they have shortage of staff they are suppose to tell us, they must tell us that we must be patient or they(nurses) must tell us that they have a meeting. Coz (because) Some other times I used to come on Wednesday and on those Wednesday, they (nurses) used to tell us to be patient and we used to be patient coz(because) they were telling us.

I: So, it means these days they are no longer telling you…

P: No like today they they didn’t tell us anything.

I: Yes.

P: Yes.

I: So, what are the things you would like to improve about health services in this facility, health facility?

P: What they must improve, they must improve time, yes even if they can have sta…enough staff.

I: Okay, now we are going to talk about HIV prevention, neh?

P: Yes.

I: Okay, what do you mean when you say nako (time)?

P: I mean time, they must start to work on time, time. Yes, time to start to work.

I: Okay, what do you understand about HIV prevention?

P: Mhm! (laughing) say again.

I: What do you understand about HIV prevention?

P: HIV prevention, can you please explain.

I: Ooh okay, what do you understand about prevention… (interrupted by participant)

P: To prevent?

I: Yes, HIV.

P: HIV.

I: Yes.

P: We prevent HIV by a condom, yes and we must make sure that we test, I must know my status. Yes so that I teach others, yes so that I can teach them.

I: Can you tell me the different types of HIV prevention services?

I: Okay, can you tell me the things that are used to prevent others from getting HIV?)

P: HIV?

I: Yes, what things are used to prevent HIV?

P: HIV? firstly, I will say it's a condom, what can I say the second one is? You will help me (laughing), help me.

I: Can you tell me the different types of HIV prevention services?

P: Eeh its pills that I get from the clinic even though I don’t know their name, but they (nurses) say I must take them for twenty-eight days yes.

I: And then what else do you know?

P: Another preventative method?

I: Yes.

P: Yes, the other one I said it’s a condom yes.

I: Okay, eeh what are some of the difficulties you may experience in accessing HIV prevention services? What things can make it difficult for you to get help so that you can prevent HIV?

P: (people talking at the back) Mmm!! (surprised) please repeat again.

I: Okay, I will repeat the question, what are some of the difficulties you may experience in accessing HIV prevention services?

P: You are saying what are the things that are difficult to prevent HIV?

I: That prevent you from getting… (interrupted by participant)

P: Get help.

I: Yes, to prevent HIV.

P: Mmhh!! (surprised) I don’t know that one.

I: ooh you don’t know.

P: Yes.

I: Okay, do you use condoms?

P: Yes.

I: Why do you use them?

P: I’m preventing HIV.

I: How often do you use them?

P: You are saying “how do I use them’’?

I: How often do you use them?

P: How often?

I: Yes.

P: Maybe once a week.

I: Where do you get them from?

P: In a box at the clinic.

I: What other places can you get them?

P: Mmhh there is no other clinic that I visit, I only come to this clinic (xxx name of clinic).

I: What would prevent you from using condoms? (silence for seconds) What can stop you from using condom?

P: Something that can stop me?

I: mhm.

P: The is nothing that can stop me from using it because I must use a condom as a female.

I: And what could prevent you from getting condoms?

I: What can stop you from getting condoms?

P: Unless if I can’t get them at the clinic because, I will never take condoms from the street. Eeh   I will never trust condoms that I get from the street, I must come here   to collect.

I: Can you explain what the universal test and treat is?

P: (laughing).

I: Okay universal test and treat it was introduced I think “2008 or 2009’’ neh?

P: Yes.

I: At the government where you test now and get medication at the same time.

P: Same time.

I: Yes, it’s what we call universal test and treat.

P: And treat.

I: Yes.

P: Okay.

I: Okay, so now we are going to talk about behavioural change?

P: Yes.

I: Since accessing the facility for HIV prevention services, could you explain how your life has been impacted or affected?

P: Mmm!! (surprised) you want to know how to I notice a person or how do I look?

I: Since you came to the clinic for services where they teach you how to prevent HIV okay?)

P: Yes.

I: So how has your life changed or how is it affected?

P: Yes, my life is fine since I started to test, I had never had a problem, because even when I come for family planning, I test. Yes, I make sure that I know my status so that I can’t stay without knowing my status mhm mhm.

I: Okay, can you explain the HIV prevention services you think have been helpful to you?

I: Okay, can you explain HIV services that have been helpful you).

P: Yes.

I: Ee.

P: As well as that the sister was talking about…

I: Yes.

P: Yes like I said you can prevent it by using a condom, even when you come to the clinic they give you a pill even though I don’t know its name, and then that pill I must take it for twenty eight days, after twenty eight I must come back to the clinic to consult so that they can check my status.

I: So, do you think those thing have helped you, those things of preventing HIV, do you think they were helpful in changing your life, so that you can prevent yourself from getting HIV?

P: Yes, they can help us.

I: So, were you happy about the time you spend in this clinic clinic today?

P: Mmm (thinking) I’m not happy at all coz (because) they took too much time, and when I have to come here for interview, I could have arrived at pass eleven, so because of eeh that side.

I: So when you came to test did you come with your partner or you only came alone?

P: I’m alone.

I: Yes.

P: Yes.

I: {(paper flipping and background noise)} Okay now is the time for us to close this interview okay?

P: Yes.

I: But before we do, is there anything else about this topic that we haven’t discussed, that you feel is important to say?

P: The most important thing is that I happy for communicating with you, because I did not know other things but for now, I think maybe I will improve there and there.

I: Now we have come to the end of our discussion. Thank you for your participation.

P: Yes.

I: If you have any questions about the study…

P: Yes.

I: You can contact us.

P: Thank you.

I: Thank you and then the end time is 13h09.

P: Okay.
