# Supplementary material for: Understanding factors influencing utilization of HIV prevention and treatment services among patients and providers in a heterogeneous setting: A qualitative study from South Africa
Source: PLOS Glob Public Health. 2022 Feb 3;2(2):e0000132. doi: 10.1371/journal.pgph.0000132 (PMC10021737; doi:10.1371/journal.pgph.0000132)
Supplement: S1 Data — (ZIP) [file pgph.0000132.s001.zip › Supplementary information/IDI_Clinic staff_QS001.pdf]

Full Participant ID: QS001

Participant Type: Female

Location: XXX (Name of Clinic)

Date: 20 July 2020

Primary interview language: English

Name of Facilitator/Interviewer: XXX (Name of RA)

Label Key

I = Interviewer

P = Participant

I: Can you tell me more about yourself?

P: Okay I’m a professional nurse for 15 years and yah, I have been working different services, I have done HAST and currently I’m doing chronic

I: Alright, can you tell me about your role in this facility?

P: I’m a professional nurse like said and then most of the time I’m the acting in charge. I’m the deputy to our which is XXX (name of the person) and yah I’m a haste pioneer whereby we, each and every service has a pioneer. Pioneer is somebody who is responsible for that service so yah

I: I heard you talking about that you worked for 15 years and then how long have you been working in this facility?

P: This facility can be 5 years

I: Alright and how long have you been working in the current role?

P: 15 years

I: and in this facility?

P: 5 years

I: you just came and started working as

P: As a transfer in. I was at the civic centre clinic for 10 years, I was at XXX (Name of hospital ) hospital for 4 years. I didn’t count that one because 4 years I was a student, I only worked for 8 years as a registered nurse. The whole 15 years I was just at Ekurhuleni

I: Oh, the whole district?

P: Yes, that’s why it’s, coming here is just transfer they needed somebody who could act when the in charge goes for leave that’s when I came here.

I: Oh okay, based on your experience, what do you feel are the major issues affecting service delivery in this facility?

P: Mmm waiting time, filing system

I: Can you please speak up so that you can be audible on the recorder?

P: Okay, the things that are hindering service delivery in our institution, one will be waiting time , okay because the patient has to wait longer for the files to be retrieved and then for them to get to be done at the observation room. Secondly will be the retrieval of the files which also delays. Thirdly will be our observation room is too small, it is not conducive. Thirdly the waiting area is also not conducive, is not well ventilated like I said the building is small. So those are the some of the things that are hindering service delivery. Shortage of staff as well and then lack of resources, sometimes you find out that tablets are out stock, essential tablets, for instance, the psych – mental health tablets were out of stock for so long

I: okay

P: and currently we have a problem with ARVs, the ARVs are not enough it's another thing. Those are the things.

I: Okay, since the covid started is the anything like covid contributed to the issues that you are facing?

P: No. it actually makes our lives easier because now we no more do observations on each and every patient, so they just wait outside, they are screen outside, we pack their medicine a day before. We just issue medicine especially the chronic ones. Only people that have to queue, draw the file and be seen at the observation room which is small are ones who are sick

I: Okay you mentioned many things such as space, filing system so I understand that in this facility there are also health systems so can you please explain or describe in your own understanding, how this standard of health system works?

P: I'm not sure I understand, what is the health system?

I: Mmm health system includes medical stuff, supply of medication and other resources

P: Remember we are actually depending on government *akere* (right), we are XXX (Name of District) clinic, but we are depending on government. So, things like those *bo di* budget and the resources we gather them from them. Even material, even the stationary we get it from government so far we haven't had anything out of stock major that we are using every day that hinders us to render the services, so in my opinion the health service is not that bad

I: Alright

P: As much as there's covid there's also some test kit, we've never run out of test kit let me just give an example, we've never run out of PPEs in our institution *rena* (us) we always have them. As much as we have the positive cases, we've never had the problem with the PPE or the test kits or anything that is related to health care services like you said.

I: Okay. So, any other than what you just explained to me, do you have any other difficulties providing health services in this facility?

P: No, except for those ones that are I have given you; no. remember we are a small clinic, so we don't experience maybe most of the issues that other clinics are experiencing. The only thing is that most of the time is difficult for us to reach the target since our premises is out of, it is not that easily accessible it needs your own transport if people are using public transport it is not easy for them to access us unlike civic centre where I used to worked

before that's where the taxis travel where the rank is. So, it's easy for people to get to that side.

I: Okay. Here people

P: Struggle especially when there's taxi strike because we are far from railway, they can't come here but civic centre because there's a railway station even if there's a taxi strike, they still use the train to go there

I: Alright. Can you please discuss some of HIV prevention and treatment available in this clinic?

P: Prevention *ke di* (is) condoms. We have condoms: males and females, and then treatment we got ARVs both for children and adults, another prevention PCR we test the kids that side. Let me start with the babies, prevention no, let me start with antenatal

I: Alright

P: Prevention there by the women who are pregnant we test each and every pregnant woman in this clinic

I: okay

P: on their first visit and I think now it's every two months, I am not good with ANC but we test them like 4 times or 5 times before they deliver and they, we used to test them each and every visit then the you know the policies they change and then they said we no more going to test them every visit, I think is now twice or three times I don't know. The reason being to prevent mother to child transmission because the mother is pregnant by there so to detect as early as possible if the woman is pregnant that is why in ANC, we encourage them to come before 20 weeks. Then we test them let's say is a first visit we test in first visit, even in subsequent visit she has to test if she is positive, we start the ARVs same day we all them fast tracking. We start the ARVs same day that's how we prevent and treat the HIV at the antenatal side. Coming to the babies' side after giving birth, the mother. And education ne (sort of question)? The mother is busy being educated ore (that) if the husband is also positive they must use condom, if the husband hasn't tested must encourage a *bai betsang kana* (what do they call it) whereby they go and test everybody at the family. I forgot the name of that thing

I: is it couple testing?

P: No, they call it, these days they call it index

I: Oh, index

P: Yes,

I: I heard about it

P: Yes, so that's also what is happening at antenatal. Then we come this side, the baby is now born. If the baby was not tested PCR at birth if the mother is positive. The minute the baby comes for 3 days the baby is being tested PCR. If the baby was tested PCR at the hospital when he or she comes for 3 days we check the results, we lab track the results.

I: Okay

P: If the results are negative then all is good, the baby is going to test again at 10 weeks. Then if the results are positive ARVs must be initiated immediately, is the same as the pregnant woman, we calling it fast track le *yona* (also). And then le (also) index testing, we see if the mother is positive and the father, what's happening the family.

I: Okay

P: Fine, we moving where are we going? Err our family planning also encourage to test we have the counsellor's room where we test every day and then no wait, at the babies I'm not yet finished. The babies if the child after 10 weeks tested negative, we are going to repeat the test at 18 months. Or if the baby is negative at 10 weeks but the mother is breastfeeding, we are going to test this same child six weeks postisation of breastfeeding.

I: Okay

P: and then if the results are negative, we discharge if they are positive, we start the treatment immediately. Then at 18 months; 1 year 6 months. When the baby comes for immunisation *ya* (of) 18 months we test again whether all these tests were negative *akere ka birth ke* (Because at birth is) PCR and it was negative, 10 weeks PCR, it was negative. Then maybe the child was not breastfeeding we never tested them we are going test them at 18 months. If the baby was breastfeeding until 9 months' six weeks postisation we test again *hora ore ke* (it means is) 11 months

I: Okay

P: Then at 18 months we test again to check if the baby is still negative then now, we that baby negative because we have done all the tests that were necessary.

I: Okay, okay

P: We are moving family planning, yes, we encourage the women to test. They mustn't just come. Our family planning, we do as fast queue we treat it as totality. They take out the files we don't just use the…

I: The card…

P: Yes, appointment card. We open the files, they screen, they do their weigh, their BP and HIV then they do their family planning

I: Okay

P: Coming this side room 8. Room 8 that's where we do our TB positive and the ones who are on treatment already and the ones who need to go for CCMDD, CCMDD is that,

I: What is that?

P: Whereby all patients who are on chronic medication ARVs being one of them, they register them on the system they go collect at Clicks.

I: Oh okay

P: Mm-uh (yes) so those ones we do viral loads *tse itwo* (Collect blood 2 times for viral load) if both the viral loads are supressed and the patients are asymptomatic is then when we transfer, we check the kidneys as well

I: Okay

P: If the creatinine is right then we transfer the client to CCMDD, to clicks. Then it’s another service *ya* (of) HIV that we render. We are coming this way. okay then this room is also ART where we do the same services as room 8 where they do their routine blood every month and all our HIV patient, they go on the Tier.net. The tier.net is the system that helps us to establish who’s due for blood, who’s due for viral load, who’s supposed to be doing what and when, and the transfer out. When we transfer a patient out (door opening and door closing). When we transfer a patient out. Okay then err the next room is chronic room, we combine everything if a patient is on ART medicine plus mental health they come to my side because I’m doing mental psych, I’m doing mental health. If the patient is on High Blood Pressure treatment, diabetic treatment plus ART they come this side but if it’s only ART then they go that side – no mental health, nothing. Then ka *mo* (here) this is our Doctor’s room then the last room that’s where we do acute. Acute is everybody who’s sick. We encourage everybody to test, that is why their room is next to testing room. We do our testing in the rooms plus there by the by the counsellors. Yah (yes) that’s how we do it

I: okay err you spoke about the condoms

P: Mm-uh (yes)

I: As part of prevention, HIV prevention

P: Yes

I: Do you think there's a lot of condom use on the clinic attendees?

P: No

I: Okay and around here I can see that there are condom dispensers

P: Yes

I: How often do you refill them?

P: Hah maybe twice a week or once a week it depends on seasons, sometimes in winter. You know when covid was starting, they were being used so much it showed that people were indoors but now it has gone down.

I: Okay let me

P: Maybe also that summer is coming

I: Alright are there any specific difficulties that you experienced providing HIV prevention services at this facility?

P: No we don’t have, it is just, you know the young ones we have youth service friendly we are offering that one as well we normally start doing that after 2 and another thing, students in uniform, they don’t queue with people without uniform. ( door opening, oh sorry from interruption, door closing) so we trying to encourage them it is just that you’d find that these young ones are shy or are afraid to take the condoms with because they are afraid their colleagues are looking at them and things like that. They sometimes come willingly especially in the afternoon to come and get information on HIV, so we’ve never experienced any problem. We don’t have actually. It can only be when there's a shortage of staff that week then there's no one to offer that service but we don’t turn them away, we try to squeeze them *mo* di (on the) adult queue and then we attend to them. So, we don’t have.

I: Okay in your own perspective can you tell me what you think about UTT?

P: Universal Testing and Treating

I: Mmhh (Yes)

P: uhm-mm it’s good (door opening, interruption)

I: Okay before the interruption you were explaining what you think about UTT.

P: Oh, UTT, I don’t see anything, I don’t see a problem with UTT *nna* (me) personally

I: mmmhhh (okay)

P: and then it depends on type of clients that we get. Some of them they don’t believe in UTT. They will tell you that I still want to go home and tell my husband or tell my partner or I still want to think before I start treatment (door opening, interruption). So like you know people are different, others, they are gladly accepting it that I’m happy you are treating me today especially those who came at the later stage you know the type of clients that we have some they knew their status 5 years ago but they were sitting at home not doing anything about it now they realised that is like they are getting sick, there's also covid and so many things that are happening then they come acting innocent as if they’ve never heard of HIV before.

I: Okay

P: Then you test them then they are positive and then you tell them these are the ARVs this is how it works I can set you ARVs today then she gladly accepts because she knows that she’s been long knowing her status but didn’t want to come and test and start treatment.

I: Okay

P: and then I like the fact that it accommodates everybody it tells you, you cannot start on this patient if there's any indication that allows you not to start, for instance, if the patient is TB we cannot start today, if the patient is TB positive or on TB treatment you cannot start today. If you suspect any medical condition that will put the client on jeopardy you don’t start the very same day. You give the information you take the blood you wait for the blood results. So, I don’t see anything wrong now with UTT, I think it’s also a good suggestion.

I: Okay so did providing UTT affect your ability to carry out other duties.

P: No, it doesn’t, no it doesn’t. it is even better than, remember previously we used to have wellness programme whereby you test the client today then you will be repeating the viral load every 6 months and the CD4 count see where they are if the CD4 counts becomes below than this number is then when you start treatment, others they end up not coming back or defaulting or doing something so with UTT is fine because now he or she can decides right there if she needs the treatment or not

I: Alright

P: The sooner the better

I: Okay I understand. So, what are some of the operational issues that you experience on providing UTT?

P: Staff. Knowledge deficit, others they don’t want to render the service because they don’t understand how it works. So I think, unfortunately because we don’t have enough staff, there's no one designated SR who’s going to do UTT it has to fall under those SRs who are doing everything concerning haste room 12 and room 8 so that’s when it becomes a problem

because after the patient is being tested let’s say in the counsellors’ room then they have to refer the patient to this room where there's already a queue and that side they queued maybe so if it was possible if there was a designated nurse who’s only doing UTT, you know that after testing or you refer those clients who wants to test, the nurse will test find the results that are positive then suggest the UTT on the clients then give the UTT there and there,

I: Okay I was also about to ask you how these issues can be resolved but I heard you talking about you need another SR there. Can you also explain all these issues that are hindering you from providing UTT that are operational issues can be resolved?

P: I think it is going to be human resource problem. If we can get a SR like I said or if you can get a person who is designated to do UTT every single day, 5 days a week it will be much better even if it is 3 days a week, patients will get used to it but it would be more effective it will be okay if it is every day and then this SR or this nurse or this person will be responsible to test these patients even he or she doesn’t test them she can work together with the counsellors of which is not going to be hectic for the counsellors remember currently the counsellors are testing everybody who is going to test, all the patients that are seen by some of the nurses especially acute and ANC so their work load is going to be a lot. Remember that they are allowed to test 6 or 8 per day, there's a number and we only have 2 counsellors, so it is going to be a problem. So, if they can get somebody who is a professional nurse like myself. This person will be able to do his or her own testing, interpret the results, do the blood, assess the patient (21:47 not audible), assess the patient, initiate ART and then it will be much better. Can you see that everything will be done in one room? The client will be asking maybe for example, lets the client comes and say I just need to test I’m not sick, he/she will be referred to that nurse. The nurse will be able to assess the patient in (22:02 not audible) okay fine I’m offering testing but let’s say you see the links glance are enlarge or something (review 22:05) the n you start asking okay, then are you on any TB treatment or do you have any medical conditions, any surgical problem anything, you are excluding things that you know ore (that) will hinder you rendering UTT. If there's anything you educate the patient ore (that) I’m going to take your blood and then you will come back maybe after 2 days to come and get your results and then we’ll see from there. When the patient comes back is still going to see the same person is not like today, she to see another person. The very same nurse who established rapport with the client is able to give the client the results, interpret those results, tell the client way forward. If they are initiating that day they initiate. If they postpone the nurse will explain to the client why we are postponing, why are we not testing, and the client will also get the information from the same SR about the index testing. So that SR is doing everything. It is the only way that we can resolve

I: Okay thank you. Now it is time for us to close this part of the interview, but before we do so, is there anything that you feel like is important to say that we haven’t discussed?

P: No, I think we have covered everything especially concerning HIV.

I: Okay, okay

P: There’s this new thing PrEP

I: PrEP?

P: Mmhh (yes) whereby they say if I tested positive, my partner is negative then my partner will be coming to the clinic then will be tested if her results are negative then my partner will be given 2 kinds of ARVs TDF and something and then he will be taking that treatment I think for a month or 2 and then after 3 months they come and repeat the test if they are still

negative, my opinion I feel like that one is encouraging the clients not to use condom because they will know that they will neglect their lives. People will start dating people who are positive, they will not use condoms knowing that the PrEP is there. It is my personal opinion.

I: Is it being offered in this clinic?

P: Yes

I: Okay

P: It is something new, but it has already started on the 1s of July

I: Is there anyone who is designated to do so?

P: No, that’s still another thing. It was pushed to those people who are already doing the ARVs.

I: Alright.

P: Mmhh (yes)

I: Thank you very much, and if you have any questions…

P: No, I don’t

I: About your participation, you can just contact us.

P: No, I don’t have

I: Okay. Thank you very much

End Time: 16:01
