# Supplementary material for: Understanding factors influencing utilization of HIV prevention and treatment services among patients and providers in a heterogeneous setting: A qualitative study from South Africa
Source: PLOS Glob Public Health. 2022 Feb 3;2(2):e0000132. doi: 10.1371/journal.pgph.0000132 (PMC10021737; doi:10.1371/journal.pgph.0000132)
Supplement: S1 Data — (ZIP) [file pgph.0000132.s001.zip › Supplementary information/IDI_Clinic staff_QS005.pdf]

Full participant ID: QS005
Participant Type: Female
Location: XXX (Name of clinic)
Date: 21 July 2020
Primary interview language: English
Name of facilitator/interviewer: XXX (Name of RA)

I: Eh..good day, good day
P: Good Day sir
I: how are you?
P: Iam well and yourself?
I: I am good, my name is (XXX Name of an interviewer) and I will be interviewing you today.
P: owk.
I: yes… eh participant is QS2005 time is 14:12, 2:12 so eh before we continue do you have any questions?
P: I would like to know what this interview is about.
I: This interview is about accessing the HIV prevention, HIV intervention in this in this facility at Alberton clinic.
P: OK
i so they want to know about how this clinic intervenes when it comes to HIV prevention and other services that you offer in this clinic
P: Alright
I: Yes, before we continue could you please tell me more about yourself?
P: I am a clinician in this facility.
I: Yes
P: My name is (XXX Name of a person)
I: Alright
P: Yeah I am allowing you to ask as much information that you need
I: Alright
P: and then I will just answer diligently as far as I can remember or whatever I know about my job

I: Alright, thank you, before we continue do you give us permission to record this conversation?
P: Yes you can record this conversation provided you gonna hold my identity
I yes, yes
P: information and giving you
I: alright thank you, everything that will be discussing here will remain confidential
P: ok
I: Yes
P: Ok
I: so how long have you worked here in this clinic?
P: About 13 years
I: 13 Years…
P: Yeah
I: so in this current role how long have you worked in this current local role? and what is your current role?
P: I am a clinician, over three years
I: Over 3 years.
P: Yeah 3 years plus
I: and then back then what were you doing?
P: I was still a professional nurse
I: alright
P: But it’s an upper rank over the professional nurse
I: alright
Yeah
I: based on your experience what are other measure issues that are affecting the services around here?
P: Staff shortage.
I: Staff shortage.
P: Staff shortage we got a lot of challenge; poor communication among ourselves; The influx coming from other townships or I don't know whether to say location
I: Owk

P: because already in albertinia servicing a lot of small areas around us but a lot of people but also there's a lot of movement from the locations e.g (XXX Name of local areas)

I: Alright

P: wherever everybody wants to see them at this clinic please say the service is good but remember this staff number is still exactly as it was some other years ago when they were influx

I: Okay

P: Yeah

I: so which which criteria are you using to select patients?

P: There isn’t

I: alright

P: we just take all the patients because we are doing all the services

I: Aleight

P: we are doing well services nobody cares Turned back unfortunately they one that are that are coming maybe 4:30 at the ones that will not taking care of

I: Alright

P: Yeah

I: so when you talking about staff shortage which staff are you referring to?

P: Clinicians

I: Clinician?

P: Yes, The professional nurses

I: Alright

P:Yeah, the ones assess, prescribe and diagnose

I: alright now we will talk about their experiences with provision of HIV prevention services

P: Yes

I: Yes, eh how how is this system works the Healthcare system.

P: The?

I: How do they access…

P: They’ve got full access because we as much as we see patients every day any patient that you come across you offer

I: Alright

P: we have to offer the service and then unfortunately most of the patients are still in denial some know themselves
I: Yeah
P: they've tested somewhere else and then they would like to confirm is it still the same here.
I: Alright
P: Some have been tested some more than five years ago they never continued care and then then come but they will restart care when they come to the facility others are still in denial they will tell you straight I'm still afraid I'm still scared I'm not doing it so some of the problems that we encounter but for any patient that we see we try and offer
I: alright
P: We try as much any any patient yeah
I: so which people are qualified to access the HIV testing.
P: all.
I: All of them?
P: Kids, every breastfed baby qualify, even breast feeding mother qualify for antenatal (… not audible) qualify all sick patience that comes to the clinic qualify. chronic will refer; family planning , any any type of patient that comes in the clinic
I: Yes
P: has…Is offered and HIV testing doesn't matter that look like everybody needs to know everybody needs to get tested okay thank you and then how is the supply of medication it was fine before they covid-19
I: Alright
P: now we are getting into a struggle because remember some of the clinics when they are closed their people are coming to (XXX Name of clinic)
I: Alright
P: so remember we order according to the clinic so our numbers now have increased we are running out we are running out, currently there is a big challenge.
I: Alright
P: Yes
I: So back then things, before covid19…   how was it…?
P: No… no There was enough

I: Enough?
P: there was enough
I: Alright
P: So we don't know because there were people were in the country before lockdown and now they can travel back
I: Yeah
P: They're stuck here they don't have medication we are supplying.
I: Alright
P: There’s visitors that are coming from where ever they also need medication you can't not offer if.. a patient medication and also there's people that belong to our Clinic that don’t have and theres newly tested that needs to be done or given treatment
I: Yes
P: you understand?
I: Yes
P: so the numbers are increasing maybe there's pressure with the pharmaceutical I don't know but medication is not sufficient.
I: Alright, and then you spoke about counselling
P: Yes
I: which which which which criteria are using to offer counseling do you have specific people?
P: Weve got both from the clinic and we have got those that are offered from   (XXX Name of institution).
I: Alright
P: But also before I I for myself before I refer.
I: Yes
P: I try and cancel the patient as much as possible for myself
I: Ok
P: and then send to The Counselor to re-emphasise on whatever I said or cover what I didn't believe it something that I've missed
I: Alright
P: but unfortunately we will do that problem if I expect according to my own research that I made wicked couple that haven't disclosed to each other they they will they will get a

woman in the room maybe with an STI in whatever you offer and then they say no I know my status and then you say does your partner know. She will tell you straight no I'm not going to tell him because he's never told me so I'm just taking my medication it stays by a friend

I: Mmmh (alright)

P: and then that is the problem so the child the biggest challenge for me is disclosure.

I: Alright.

P: I don't know if we are not giving them enough information but we are missing out on the disclosure thing because yesterday. I did… I got a 14 year old.

I: Yes

P: Its an example she comes she's got a painful legs painful lower legs and feet obviously you thinking neuropathy.

I: Mmmh (alright)

P: But you also doing a urine test is negative TB sugar there's nothing just to make sure the kidneys and diabetes are not there

I: Yes

P: And then work left is HIV offering.

I: Yes

P: Its a 14-year old is a minor and fortunately the parent of one of the parents was here so I went out to talk to the parent. The parent gave me a consent only to find that the parents are on treatment but they never told the children and unfortunately the result of the total came back positive. So in those cases…

I: Yes

P: I feel just disclosure is not done is not is not encouraged not done enough but there is somewhere we are missing the point because we shouldn't be having kids that don't know, and fortunately our government has said musi neh (right)

I: Yes

P: That we have to test any child between 0 and 15 years

I: Yes

P: So all those children I think if we can continue doing this you're going to catch them but unfortunately how do you explain to a child about testing when the parents hasn't said anything.

I: …Anything, yes
P: Its still stigmatized HIV, It’s a problem.
I: It’s a big problem. what are the strength of the system that you are using what are the strength of this system? Judging from things I mean...
P: but part of the system, Part of the system working but with knowledge I think people who are knowledgeable.
I: Mmm (alright)
P: Give the the system strength but those that are still in the dark and still believes in old things or traditional medicine…
I: Yes
P: We're losing them we are losing.
I: Do you still have patients that are still believing in tradition when it comes to HIV?
P: Yes, we still have.
I: How…?
P: Some other time they were to be healed from shingles.
I: Mmm (alright)
P: Remember the treated traditionally but they don't believe that they needed to do an HIV test and because sometimes you can persistently from us
I: Mmm (ok)
P: We push them to get tested and fortunately they have started treatment. So I would say somewhere along the line we need to teach our… we were lacking information that promotion,
I: Mmm (ok)
P: We not targeting enough out there they don't understand I still believe because ori(they say) shingles was treated three years ago but I still have pain somewhere I feel that these pain then you are assessing but through the history you saying “have you tested for HIV?’’ no I haven’t, now what do you do?
I: Yeah
P: Yeah, so at least we got them like that but how many of the drilling traditional medication and not not knowing their stages there's three people out there
I: Yes. And then do you have any awareness of HIV awareness?
P: Our…unfortunately our health promoters right now she is not in but she does you know.

I: Ok

P: On maybe twice a month or three times a month will be conversing in the clinic and then on the 1st of December when we celebrate the world AIDS day then it's a big thing.\

I: Ok

P: we do we do but she can't be alone I think she needs assistance like right now when she's not on inside she's on the gra… Shes on the ground there, somebody needs to be continuing think we need continuity of service on the ground.

I: Mmm (ok). When talking about being on the ground there, what do you mean by that?

P: I mean shes on the field visiting teaching the public about COVI19 isn’t it.

I: alright

P: shes teaching the public about covid19; the importance of wearing masks blah blah blah.

I: Alright

P: Yes

I: and then before Covid19 how were how was the clinic communicating with their communities do you have any linkage?

P: Yeah there are community leaders, we have got (XXX Name of a person)

I: Yes

P: from the squartercamp. We liaise with the Ward councillor (XXX Name of a place).

I: Ok

P: Those are the people that bring their people and then we have monthly meetings

I: Alright

P: Normally, because we have a communication box were by the public writes their demands, and monthly its open we discuss and then we look for way forward we look for way forward. This is how we improve our services.

I: Good. Good , ok right so do you also offer prevention? HIV prevention services

P: Not yet, it was just introduced. It was just introduced like a month ago, so the stock hasn't come yet.

I: Ok,

P: Yeah but we were referring referring them to (XXX Name of hospital).

I: Alright

P: so that they can get treatment and review.

I: Alright

P: Yeah
I: And then how is the access of condoms in this clinic?
P: 100% there
I: 100%.
P: Yeah, we’ve got each room. Each office keeps condom but there’s also a box at the front.
I: Alright
P: So that those that, you know sometimes people shy away from taking condom they get them from the office because one patient will say I am not going to that box , so you give in the office is easy to access them
I: Oh alright. and then which which condoms is it for males or females?
P: Both
I: Alright
P: We even teach on how to use them; they are struggling but we still teaching.
I: Ok, Do you have any familiar cases of condom burst?
P: A lot
I: A lot…
P: Yeah
I: When, are they… are they telling you about the causes?
P: You know…
I: Did you find the reasons behind?
P: Yeah I found most of the time it's poor storage; eh expired condoms. Our people are still somewhere.
I: Mmmh (alright)
P: They don't understand the storage but after you've thoughts that person. I promised you they are not coming there if you teach them about storage how to store condom where to keep it.
I: Yes
P: Don’t put them in the pocket, walk around the mall; keeping the pocket in the sun.
I: Mmmh (yes)
P: You know they do understand
I: Yes
P: Yeah

I: Alright

P: And the reusing of condoms because some would say we had only one condom so..

I: Yeah

P: And we had incidences were I have removed two actually, I have never referred to the hospital but they had to be removed from the woman deep deep deep.

I: Yeah

P: Yes, so you teach them, if you teach you can't go wrong.

I: Yeah

P: knowledge is power, we need more health promoters to be able to cut in and teach.

I: Yes

P: Because where a sister cant reach we need somebody who will do.

I: That's true

P: Yes.

I: Are there any specific difficulties that you are experiencing providing HIV Prevention services?

P: No

I: Specifically, in this facility?

P: No.

I: Ok, what changes would you like to addressed, to see addressed?

P: You know, I would like our counsellors to be empowered. you know they are missing certain things like I would wish they'll be taught about few conditions that are common, common conditions whereby they are able to pick up if a patient need remember its not about testing only

I: Mmm (yes)

P: If they screen more, they can find the STI that the patient never said anything about because some of the patients they are just coming from outside straight to testing.

I: Yes

P: Volunteer testing but then I will be testing then not asking anything about their health or anything, we missing STIs; we missing a lot of problems; we missing pregnancies; we missing eh our less than weeks pregnant women that are coming in for testing but they are going out.

I: Alright

P: If our counsellors or lay counsellors they can be taught more on how to screen, the screen.
I: Yes
P: The screen then we would be somewhere
I:Ok
P: Because we missing many people
I: Yes
P: You are here for what ok testing, you test. Meanwhile the same patient has blood pressure.
I: Mmm (yes)
P: They got headache, you ar no reporting back to the sister to say that I have go this patient she complaining about headache that started when, and let that patient do blood pressure and teach them to be able to read that is that blood pressure of yours abnormal. Some patients are going there for family planning. After family planning they test
I: Mmm (yes)
P: Nobody is asking about ‘’no but your blood pressure is high ‘I think if we have screening tool where they know this are the abnormalities these are the right things; these are the referrals, refer back to the sister.
I: Mmm (yes)

P: Not referred to the sister because the patient has to take treatment but referred because I have identified a problem.
I: Yes
P: Like we still have social worker, we have psychologist remember.
I: Mmm (yes)
P: It might not be the results that are shocking or making the patient cry at that particular time, you may find that there is a social need.
I: Mmm (yes)
P: And is not attended to because I am a counsellor, so the sister is going to miss it and then the next thing the patient is going.
I: Yes
P: We`ve missed that patient, and the the patient goes out with that stress.

I: Mmm (yes)
P: Commit suicide, we are complaining
I: Mmm (yes)
P: Do you understand ?
I: I understand
P: Yeah, more more emphasis on the screening teach our Lay counsellors enough.
I: Yeah
P: So that they are able, to identify a problem about that la(here). There is that mother instinct and that medical instinct
I: Mmm (yes)
P: That theres gonna be a problem if I am not talking to this now there’s a big problem coming, especially with males.
I: Mmm (yes)
P: who looks after males’ version, in the room they bring headache, if you know how to screen you come up with more, they you are able to refer but If they come and test and go the problem left with them.
I: Mmm (yes) alright
P: Yeah
I: Thank you very much. eh, what do you understand about universal test and treat.
P: I.. I understand that everybody needs to get tested. everybody needs to get treatment if they are positive instantly, no denials for treatment. everybody, not anybody by choice. everybody needs to get, if they test positive they need to get treatment, they need to be offered treatment...ready or not, then if it means they are not ready re-emphasis and counselling… that's what I understand.
I: oryt
P: because we are trying to combat HIV for the nation, we need an HIV free nation in the coming years.
I: yes. does UTT universal test and treat affect any other duties?
P: Jah because we are short staffed. remember, every clinician here has a specific service they render. so if you are busy doing that and then there's a client that needs to get tasted it takes longer, and then it affects waiting time for the client that's been here in the morning. you understand?

I: Yes.

P: so we need man power, we need man power.

I: more staff?

P: more staff.

I: okay.

P: to provide the UTT. then we know we are going to refer directly to these 5 people.so those 5 people don't concentrate on our job, so we continue as normal. we screen, we trace, we find, we refer.

I: Alright. any other issues that you think need to be addressed on UTT?

P: so far, no... so far, no

I: and then how often do you refill the condom tray. I just want to understand the demand of condoms.

P: it could be twice or 3 times a week. if they run-out we fill. we refill. there's no specific day where we say on Monday, stock is always available. so as long as the tray goes... runs out, they refill. the guys that's sitting by the table there, every time you will out in the offices. and then we are fine, am sorted.

I: and then you are using the FIFO method to distribute the condoms?

P: eheh.. first in first out.

P: good. Good. okay. do you have anything or anything important that you think we didn't mention that you would like us to discuss?

P: no.

I: any question?

P: no.

I: Alright... and then..

P:no questions, no editions.

I: no editions no questions?

P: jah.

I: Alright.. eh we. we have come to the end of our discussion.

P: okay.

I: yes. and then if there's nothing that you would like us to talk about, then I can only ask you one more question .

P: no problem.

I: yes. eh, since we are experiencing this covid19 and you also have HIV patience that you need to do follow up on, are there any difficulties that you are facing when it comes to linking the 2?
P: yes
I: since.
P: because some present. Especially like HIV, you still eh.. patience will still present with pneumonia, you remember.
I: yes.
P: and now with the covid19 the symptoms are similar to pneumonia symptoms. so sometimes it's a bit confusing, but like I would say a thorough investigation with thorough screening, even if they don't have a temperature you have to be able to separate to two. do you understand?
I: yes.
P: but there's no a stop to say HIV patients are neglected because of covid19, no. no.
I: then when it comes to follow ups, are you able to track them during lockdown?
P: jah.. they are, they are traceable.
I: owoh.
P: and because we have got a tracker, they phone them a week before to remind them, and if they come up, a day before we find out what was their reason, if they are somewhere and then we refer them, go to your nearest clinic and please get back to us and tell us if they provided you with medication or not, failing which you have to make a plan and come back. so far I don't think we are missing... the people that are not... that we are losing are the people that are not following themselves... yes.
I: and then.
P: 90 percent of the patients in our clinic are responsible...
I: okay, good good good.. and then then rate of defaulters is in increasing or decreasing?
P: it's fair.
I: it's fair?
P: mhmm.
I: okay. that's good.
P: there will always be in and outs. there will always be in and outs.
I: are you able to retain them?

P: jah
I: okay.
P: yes.
I: which system are you using?
P: the tracking
I: Alright. thank you very much, thank you for you time. we have come to an end of our discussion. do you still... you are still sorted? no questions no editions?
P: am still sorted
I: Alright. thank you very much.
P: no problem... pleasure
