# Supplementary material for: Understanding factors influencing utilization of HIV prevention and treatment services among patients and providers in a heterogeneous setting: A qualitative study from South Africa
Source: PLOS Glob Public Health. 2022 Feb 3;2(2):e0000132. doi: 10.1371/journal.pgph.0000132 (PMC10021737; doi:10.1371/journal.pgph.0000132)
Supplement: S1 Data — (ZIP) [file pgph.0000132.s001.zip › Supplementary information/IDI_Clinic staff_QS010.pdf]

Full participant ID: QS010

Participant Type: Female

Location: XXX (Name of Clinic)

Date: 20 July 2020

Primary interview language: English

Name of facilitator: XXX (Name of RA)

(NB: There is background noise during the entire course of the session).

I: [Flipping page] Ehh thank you for taking time to participate in our study, this is a qualitative interview and then participant ehh ID is S… QS010. Ehh we are in XXX (Name of Clinic) and the participant type is female and primary language it will be English and date is the 27th of July and name of the interviewer is XXX (name of RA) and Starting time is [sound of unzipping something] 09:22. Do you allow us to record this interview?

P: Yes.

I: Thank you. Ehh can you please tell me about yourself.

P: My name is XXX Name of participant) [sound of a chair] I was born in XXX (Name of place) in 1992, ehh I went to XXX (Name of primary school) based in XXX(Name of place) and then my High School I attended at XXX (Name of high school) mnca ehh until grade 12 and then did my nursing at XXX (Name of nursing school)in 2011 until 20 four… 2014 it's a four year course, mnca then I came to work at XXX (Name of clinic). On 2018 I did my speciality in Primary Health Care at XXX (Name of tertiary institution) mnca ehh yah, and then until today.

I: Mnca, Thank you and then ehh can you please tell me about your role.

I: In this facility…

P: I… in this facility?

I: Yes.

P: I’m a primary Health care worker, uhmm now I’m doing chronic services but more… more focused on TB at the moment. Uhhh I see TB patients, we initi… we ini… we initiate TB treatment mnca, we also do the HIV, we initiate, we do the follow ups, the Diabetes, HIV, Hypertensive, Asthma, Epilepsy mnca and what did i…?, yah and Arthritis. Mprr.

I: Okay.

P: Mhmm.

I: Ehh since there’s a load shedding ehh, how long have you worked in this ehh health facility?

P: It’s six years.

I: Six years.

P: Yah.

I: And then ehh how long have you worked in your current role?

P: Ehh it’s an… an… now it’s one year, how many months? *Kana* it’s August, one year eight…

I: One year eight…

P: One year eight months.

I: Okay. Based on your experiences, what do you feel are the major issues affecting service delivery in this facility?

P: Mhmmm (thinking) mnca I think seeing the whole…whole…whole population of XXX (Name of place) in one clinic cause, we over crowded, clinic is over crowded ehh we are enable (unable) to see patients in time. Ehh the other thing, the issue of drug supply. Sometimes there’s a shortage of medication like now, we’re having shortage of ehh FDC so those are the things, the drugs, the over crowding.

I: Okay, and then ehh since you say you have shortage of FDC, how do you deal with that?

P: We go and ask at XXX (Name of Hospital) they give us maybe… (wondering) how many? Sixty ehh tablets, ha-aa (no) sixty hha (sigh)

I: Containers.

P: Kere containers of FDC’s and then ehh we… we count seven tablets per patient that do take t… FDC but then others we try to mnca initiate them on the new drug based on their blood results of viral load. The TLD.

I: And how does that affect the patients?

P: It affects them so bad cause they… some of them sometimes they come, XXX (Name of Hospital) couldn’t supply us with that much, we give them one one and they should come back again. So its more or less we are working… we can see one patients three to four times in a… in a… in a week.

I: Mhm (okay)

P: So its bad, cause depending on the medication. They can promise you to give you this ehh medication this today, tomorrow they saying they have a problem, than you to [noise of something outside] tell the patients to go back home, come back tomorrow and see if there is a medication yahh it’s really bad.

I: And how..

P: Its frustrating.

I: And how does ehh the patient responds to, if you tell them to come back tomorrow, are they okay with it?

P: Some they are okay, some they are not. They get angry, they swear at us but then there’s nothing we can do. There’s nothing. [Background noise]

I: I hear you. Ehh now we gonna be talking about experiences with provision of HIV prevention services. The health system it includes a medical staff, supply of medication and other health resources, commodities, information and access to health services. Overall can you please describe your understanding of how this standard health care system works.

P: Ehh… repeat that question again.

I: Okay, mnca. The health system includes medical stuff, ehh supply of medication and other health resources slash commodities, information and access to health services. Overall can you please describe your understanding of how this standard of health care system works.

P: I think ehh it's us the… the… the health workers ehh seeing the patients or the clients and then if they have another mnca other problems which we cannot be able to assist with we, we refer them to the aligned health care workers and then there's… in aligned *akere* (isn't it) we have physio, our pharmacist that will… which will follow (fall) under the supply of medication and the social workers, yah I think that's how the… the… the referral of patients in… in one facility (softly) yah I guess.

I: Okay, and what are some of the strengths and weaknesses of this system?

P: Ehh [people talking outside] *herere di* (when we say) weaknesses, weaknesses of those things ehh lets say the patient has a psychological matter, we refer the patient to the psychological… psychologist and then it takes more days, cause they don't… they are not based at the facility. So the patients have to wait and then some of them they… they become angry and not ehh mnca [noise outside] *kereng?* (what should I say?) [more noise] ehh they lose… they tend to lose hope that they going to be helped cause that person maybe she won't be able to come, the aligned health care worker wont be able to come due to ehh sick, the leaves and the patients have to be postponed. So its, the time frame its bad and then the strength kegure (its that) patients are being helped by the….kereng (what can I say) by the are helped by oh hoh siyile seguwa (English is gone)

I: Buwa ka Sotho, ka sethu (Talk in vernacular) I`ll translate it.

P: Bathuswe kebathu balekureng, bayi thutile (By being helped by the people who have studied for) that thing yah. So the professional barrier about it, its good on handling their matters. Yah

I: Okay, so you, you made an example with regards to the psychologist, so how is the system with regards to the social worker or a dietitian do you have such?

P: Dietitian we have a problem now she's on leave, we only have one there`s no one standing on her behalf our patients are not being helped and then

there were, before she left we didn`t have the some of the nutrition’s like the, those soft porrage that they, the diet of people with low weight and then we having, we don’t have anything so they have to wait for her to come back after 3 weeks that’s a problem le (even) social worker leyone(as well)its not based here everyday, she only come here two days or one day and goes to other facilities most of the time she is at XXX (Name of Hospital).

I: Okay, and is there any difficulties providing health service, services at this facility?

P: No there are no difficulties, not at all, everything its okay.

I Okay, please discuss some of the HIV prevention and treatment interventions available in this clinic.

P: Eh not yet available because they were saying, the were talking that they will introduce ternimine as a prevention for the youth people who are testing negative and then they are sexually active but then the system has not yet rolled out, we are still waiting for enrolment date.

I: What is ternimine?

P: Ternimine is…

I: It`s ternimine?

P: It`s ternemine, it’s a combination of tablets imetersevene tablet and tenofovir tablet.

I: And what is it used for?

P: We use, we use it for patient who are negative, HIV negative to prevent them from getting HIV or being exposed to HIV when they are sexually active. So we issue it out eh.., the research that was done by ANOVA, they said that its working for those people so even if they don’t use condom, they don’t condomise with people who are HIV positive, with a suppressed viral load, they are at a low risk of getting it.

I: So if I hear you correctly its like PreP?

P: Its like it. Yah but then they don`t say its..

I: PreP?

P: Its PreP.

I: Okay.

P: But they say its Pre-Exposure Proselytises.

I: Okay

P: Of HIV but they say there’s a difference.

I: Okay

P: Yah theres a difference cause PreP we use to give people who are, after post exposure.

I: Yes. Ke(It`s) PeP le(and) PreP.

P: Yah its PreP.

I: Oka and how about condom use, do you think there is a lot of condom use among the clinic attendees?

P: No there`s not. We give them each and every patient in the consulting rom we give them ama-condoms but then when they get home they don`t use those condoms. You can decant the patient for six months, before they come back eh after six months they are, most of them they are pregnant, so they don’t use condom. They just say they do but then in actions its not, its not happening.

I: I heard you say you decant patient, what does the word decant mean?

P: When we say we decanting a patient we, we mean the HIV patient her viral load its less than 50 to or more of them with a six months apart we take them to the program called CCMDD and then they get their medication as Sphephelo box, they collect there each and every two months.

I: I heard you say CCMDD, what does that stand for?

P: It`s chronic there other one I don’t have, it`s a abbreviation…..

I: Uyilibetsi(Have you forgot)?

P: Yah.

I: Oh it`s fine.

P: Yah keyi lebetsi kesayi tlakansa(I forgot it let me not mix it up).

I: Okay does the decanting system work for only HIV patients?

P: No. We also do it for other chronic eh conditions like diabetics, hypotension and those ones they have to be controlled for 2 years.

I: Okay.

P: Yah, otherwise if its not 2 years then we don`t take them out cause if ever our patients especially diabetes we don`t even have more that 20 outside cause diabetic, their blood sugar is not controllable at all.

I: Okay, so with this whole ehh Covid-19, how this has affected the facility?

P: Yoh the Covid -19 is bad, its bad, it`s just another work. It`s overload on its own cause now we have to take one, another one professionals to do the Covid-19 test that makes shortage and then the ques of the patients has to be long than they used to be and they there has to be, yah its overloading. We having more people coming in, they don`t even go to demarcate, their demarcated areas. They come to any facilities, its real really bad and we have more positive cases of our client but then amazing part is that they are not our demarcated areas as I`m saying we testing everyone cause they can say company in XXX (Name of place) test positive they all come here from XXX (Name of place) to XXX (Name of place). When all those people they are all HIV positive but they are our positive but they are not for our demarcation area.

I: And how has it affected your participants?

P: Eh its bad…. Its bad but then we trying our level best even though sometimes we knock of at 16:30pm but then we have to go around 19:00pm just for the sake of patients cause they have been here for a longer time and then the patients they do understand cause they see that there’s shortage of created by this Covid. There`s always a long que because of this thing of Covid.

I: Okay. And has the facility had to close down because of the Covid?

P: Yes, that was around May on the 26 ahaa(no) on the 15 it was the 15 and we closed down for two days then for fumigation and to clean yah.

I: And then as you mention that eh participants do not use condoms and then come back pregnant, how often do you refill the condom dispenser?

P: every day, every day we having many condoms at the facility like right now at the reception, at the counselling room each and every room. Every day in the morning we issue out a new box but then its bad its really bad.

I: So they do take?

P: They do take but then they don`t utilise them. (noise)

P: They can tell you ahh sister I took, I do have them but then my husband because we are married his refusing to use condom, then it’s a problem. You bring them both and then they do agree and then after next week they change, they go back so….

I: So, if I hear you correctly you say if a participant say they can`t use a condom due to their male partner refusing to use a condom. You call the partner in?

P: Yes.

I: And then you do have a talk with both the couple?

P: Yes.

I: And then they agree?

P: They agree.

I: And then when they get home they story changes?

P: The story changes. They will tell you that they cannot be told by somebody on how to maintain their bedroom issues, so…

I: Alright, are there any specific difficulties that experienced when providing HIV prevention services at this facility?

P: That one I will say, as I`ve said we are not yet doing that service so I cannot say there are difficulties or not because we don`t, we have not started yet.

I: Okay.

P: Yah.

I: Beside the PreP, before there was PreP if you had to talk to me about the prevention of HIV, which are the issues that you would touch on?

P: Prevention we would talk about the use of condom and then the encouraging your partner to come and test because your partner might not know their status so its very important that when you are get to have a new partner, you come with your partner you do the test together so that you both know the status and the we encourage the use of condom at all the time.

I: Alright.

P: And then even this eh telling them if they wish to have a baby maybe the other partner is negative the other one is positive then we say to the negative one if they are now ready to have a partner, they must come and then we do the ELISA and then we give them 2 months that they must try if they. The viral load of other partner must be suppressed which is less than 50. If they didn`t catch during that period 2 months, they come back and we do the test to the negative one and that positive we check the viral load.

I: Okay so, during that process of us maybe lets say me and my boyfriend, we wanting to have a child is there anything that you are giving us, any information on how to check if I`m ovulating are there any systems?

P: We, we do check, we do tell them about the ovulation thing but then some of them you get that they don`t understand, the community that we are in is not more literate so we try to explain that before you know huri(that) you going for you menstrual its where you more fertile than when you went and then yah we do try, about the ovulation we do try but then we don`t go deeper cause you are, you are loosing them there you just, most of the time you see you just if the patient is not literate you just say just go and try at home yah.

I: Okay. What changes if any would you like to see addressed to these challenges?

P: The one ya(that) prevention?

I: Yes.

P: Yoh.. What can you say is the challenges? The challenge I don`t think even the government can address it cause its within a person. If a person told themselves I`m not going to use a condom there’s nothing we can do as health workers but then as long as we try the most of our best to give health education give the advantages and the disadvantages and then I think if we focus more on the baby. More on the baby that if you are not using condom, you not checking your HIV status or your viral load then you know that the baby will be affected. Then in that regard more of them they tend to listen the baby story cause if you focus on them the they say nah will see when we get there but if you tell them about the baby, you show them pictures they tend to get that fear.

I: Okay. And part 3 of our session we will be discussing experiences with providing or provision of universal test and treat. Can you tell me what you think about UTT from your own perspective.

P: UTT to me its bad, why is it bad? Ah you test a person today, you give the drug, you don`t know their kidney status yet and when the results come back the creatinine of the person its bad, its high so you have take out the tenofovir treatment out the person was already on that regiment so I don`t know why is it wise to start people on treatment yet you don`t know their em kering(what can I say) the results of their kidneys cause kidneys its a vital for me so most of them we loose them there whether they are young or not, cause most of them are young people we`ve seen them 23, 21 the usually their creatinine is not okay cause they don`t drink water those people you know they are more on form fizzies so I think you are going to lose more patients there hence now days the people on kidney failure its  going up because of the UTT I don`t like it.

I: So you say they take more of fizzies, what do you mean when you say fizzies?

P: Its soft drinks like your Coke, they don`t drink water at all, they don`t drink water. They will tell you, I only take a sip of water during my tooth brush, after taking the tooth brush then they sip some water then during the day no water at all so, the kidney gets to be affected then when you issue then, when you give them on tenofovir, tenofovir regimen then its another story.

I: Mmm.

P: Its more like we are killing patients yah than saving their lives so we, we usually use our own discretion cause you cannot tell by looking the patient physically by looking at the patient you cannot see if their kidney result are okay.

I: Mmm.

P: So usually we check with age cause we had cases with ages.

I: Mmm.

P: So we don`t issue drugs until we wait for the results to come back. We use our discretion then we don’t follow the UTT guidelines.

I: So if you say you tend to look at the age, what is the criteria that you use?

P: Eh 1996 to 2003 we don`t give them same day and then the old ages 1954 to 1967 we don`t give them until we get the results.

I: Ohh. You wait for the baseline?

P: Yes we wait for the baseline results and then we do give them after 3 days of those results.

I: And eh, did providing UTT affect your ability to carry out any of your other duties?

P: No not at all. Not at all cause we had another, we had other staff from Aurum, lucky us so they help us with the initiations most of the time but then we do when they are not there and then when we do have more man power we do help so, it didn’t affect us at all.

I: So when you don`t have man power, how do you?

P: Meaning that day we only utilise the Aurum people yah.

I: Okay. What are some of the operational issues that you experience when providing UTT and how cane these be addressed to resolve these issues?

P: Professionally are the issue of the new drug yah. The issue of the new drug we have the new TLD, the teletocravir, tenofovir and the lamivudine drug is a

new drug TLD that’s our combination and then the operational part the TEE the shortage. The TLD we are not supposed to give to the child bearing people age so we have the shortage of TEE.

I: Mmm.

P: And most HIV positive people are at child bearing age, so it’s a problem, it’s a real challenge so we tend to that shortage story where you have to go to other facilities and borrow yah.

I: And what is TEE?

P: TEE its tenofovir, efivarenz and emtricitabine combination, it’s a combination.

I: Alright. And then how do you resolve these issues?

P: Ah for now they will tell you no we have this Covid issue its bad, no one want to take responsibility for this child bearing ages cause when we say should we give then they say yah hey give and write it down they don`t want to they just want to tell you by the mouth but then not in writing but then that you cannot do cause we need something in writing. You know as nurses you cannot just do as you want so, it just giving us problems everyone from the top there you know Dr. XXX (Name of doctor) doesn’t want to take accountability, we call our pharmacist, she will tell you no tell the patients to go back home let them ask friends who have those medication to let them take one, one will try to get to sort something. Its just a stress and then it create overcrowding at the clinic cause they will come back and come back, its just the TEE story its just bad.

I: So how has this affected the adherence?

P: The adherence obviously it will be bad, I`m thinking that the patients that the chording of June people, when we going to take their viral load it going to be a little high cause of the drug compliance that we are facing now. I thing the government has lost it there cause they are focusing on the Covid and lost focus on HIV. We don’t have enough stock of TEE especially in places that we are at XXX (name of place) its bad, I think in other facilities it will be more better cause more of their people will be suppressed, it goes by communities

yah cause Winnie its more like rural yah they still need that health education. More people are not working so hoppy at homes its sexual things.

I: So if you were the government, how would you put this whole thing or how would you make it work?

P: Ah, I think I will try to balance the both sides.

I: Mmmm

P: Focus, yes we know there is Covid, is according to me is nothing new before we had pneumonia in all the years just started more of it so, more of the health talk on the Covid. You cannot force people to wash hands, to wear mask and not to go around but the HIV people, these people they need drugs too in order to live too so, focus on both balance the two of it make sure that the ARVs are still being supplied. The more you want to supply the with the issues of Covid, with the social relief and whatever, but then don`t forget to also giving the chronic people and then what if those people, those patients get infected with the Covid the more the deaths come to increase yah so the losing focus more on chronic medication.

I: And how has this Covid affected cause I hear you talking more about the HIV, the TB patients how has it affected them?

P: TB patients ah lebona(also with them) we don’t have Rafinah 300/500 mg. So drug compliance its, its bad cause nower days I have patients who are on their 23rd sputum it comes back positive cause they tend to say “no but then my rafinah used to be two tablets now I`m taking four cause I have 150 mg. and 75mg of rafinah so I have to double the dose in order to make that dose.” So they tend to loose, their compliance is not good so hence we get more positive. So we have to re-initiate them.

I: Mmm.

P: So the drug supply is an issue, it’s a big issue cause patients you tell them now you on the initiation phase you`re going to take two tablets and then out of the blue you don`t have enough stock you going to be taken out of tablets again, they get angry, angry and then they tend, they tend to lose hope and say “no you meaning I`m not getting okay you don`t want to tell me the truth. Why

am I going back to the fourth tablet?”. And then hance from last week I have been getting had, I was supposed to be discharging the results are coming back positive cause we have been having ke(its) 3 months now not having that rafinah and nothing is being done.

I: And what is the reason for it when you try…

P: They will tell you that the supply where they are getting it cannot be transported due to this corona thing, that’s the story that we get. But then they can supply things of Covid there`s no transport issues there so you see hence I`m saying both needs to being balanced well. They don’t, they just less focus the, on our chronic`s and then its causing havoc at the facilities and that’s another thing they need to be focusing on.

I: Eh, now it is time for use to close this part of the interview but before we do, is there anything else about the topic that we haven’t discuss that you feel that its important that we didn’t mention?

P: Mmm no.

I: Pardon.

P: Nothing

I: Okay. Eh now we come to the end of our discussion. Thank you for your participation, if you have any questions about our study participation please feel free to contact us. Thank you.

P: Thank you.
