# Supplementary material for: Understanding factors influencing utilization of HIV prevention and treatment services among patients and providers in a heterogeneous setting: A qualitative study from South Africa
Source: PLOS Glob Public Health. 2022 Feb 3;2(2):e0000132. doi: 10.1371/journal.pgph.0000132 (PMC10021737; doi:10.1371/journal.pgph.0000132)
Supplement: S1 Data — (ZIP) [file pgph.0000132.s001.zip › Supplementary information/IDI_Stakeholder_QSH001.pdf]

1. PARTICIPANT ID NUMBER: QSH001
2. RESEARCH ASSISTANT: XXX (Name of RA)
3. CLINIC NAME : XXX (Name of Clinic)
4. TYPE OF THE PARTICIPANT: Female
5. LANGUAGE: English
6. TIME: 09:22
7. I. Can you tell me more about yourself?
8. P. Okay, myself I am a person who like working with people. A person who likes to spend more of my time assisting and a person who is not afraid to meet with challenges and I am a person who is allergenic I can even work under pressure. Than I am someone who is full of jokes but when comes to my work I concentrate. Then I always make sure that I do the best, so that whoever I assist won't forget what I did to that person.
9. I. Okay thank you, can you please tell me about your roll in this clinic?
10. P. Okay ahhh (thinking) in this clinic I am doing HIV counseling and I am also doing HIV self-screening testing, than my role is to teach people about HIV ahhh (thinking) to communicate with whoever who come to the clinic. And tell them more about testing HIV (Noise at the background) and then ahhh (Thinking) and recruit the patient who are coming to the clinic whether they are coming for chronic diseases, acute stream mostly those who are coming for family planning. We recruit them for HIV testing and those mothers those bring their children for immunizations we also recruit them, for doing HIV testing.
11. I. Okay thank you, How long have you being working in this area and how long clinics did you support in this area?

12. P. Mmm in this area I started last year October in this area, than I can say I don't have a permanent place where I can say I am supporting I can say probably I am supporting all the clinic of the north. Most of the time last year I was based this side.
13. I. Okay, what sort of services do provide for the clinics operating in this area.
14. P. I am providing education, HIV counseling I am also providing testing service for HIV.
15. I. Okay thank you, please describe your relationship in your area of operations?
16. P. I can say the relationship with the clinics are good course ahhh (Thinking) we manage to work with the patients of different problems, we manage to engage with the clinic staff we respect each other we teach each other we learn from each other, if I don't know something that must be needed I ask from one of the staff so that the person can help me. So we also learn a how the clinic channels working form the reception to where the person can find help either in treatment or get the right information.
17. I. Okay, based on your experience what do you feel are the major issues affecting service delivery in this area?
18. P. Ahhh the major issues is that most of the clients they come to the clinic looking at how the people in the facility well come them, or how the facility operates. Because each and every facility operates differently. You can find that someone of that side of (XXX (Name of place) come this side of (XXX (Name of place) for to get help here either because they don't want to disclosed their status at their nearest facility because they are lot if people that they know that side. So you find that people they come this side and they don't stay here they fake addresses so it's a little bit challenging.
19. I. So what did you do if that particular person fakes the addresses?
20. P. Ahhh (thinking) most of the time we ask them to come with the prove of address of where they are staying. And we check if they gave us the right number so that if the is someone who

default treatment we can trace and call if we don't find that person because some of the people they relocate so we cannot deny someone to get treatment because they don't stay around. Some of them they work around here but they don't stay here. They come to this clinic because they work around because its near their work place.

21. I. So in most cases what did you do if the patients don't take treatment at all, and what is your role on that?
22. P. If the person is not taking the treatment we try to give that person counseling so that we explain and teach the importance of taking medication. So that we also understand the challenges or the fears of that person, why the person is not taking the medication. We try to understand his or her situation because some of them they have serious challenges. If the problem is bigger the way we can see then we can also refer the patient to the psychology or to the social worker.
23. I. Okay thank you, describe your understanding of how the standard healthcare works in this clinic?
24. P. Ahhh (thinking) about that I can say what I understand is ahhh that a very patient that comes here in the clinic is treated equally, the staff doesn't discriminate their patient. Every patient who is in treatment has being taught or explained how to take that medication.
25. I. Okay thank you.
26. P. Yes.
27. I. What are the some of strength and weak point of this service?
28. P. Some of the I can start with the weakness is that we are unfortunate whenever the patient is giving us wrong information cos we are trying to help if you give the wrong information about yourself, maybe we are asking that you are taking medication on time or are you taking medication everyday if the person admit or doesn't even ahhh tell us the difficulty of taking

medication we won't be able to know or to help the person further, cos some other time we see the person come back and he is very sick or we trace the person and we don't find that person because of the information that the person gave us.

29. I. What can be done to ensure that the current healthcare in XXX (Name of District) is strengthened?
30. P. Ahhh (thinking) I think ahhh the current situation must be changed in the way that the we allocate people to the clinic, a person must be allowed to go to whatever clinic the feels that want to go. So that the person must be free, if the person see that she or he will be able to fetch the medication that side then the person must be given the access to medication.
31. I. Okay thank you, in your experience can you please describe the HIV prevention intervention available in this area?
32. P. Okay (thinking) for what I experience the preventions that has being assisting condoms. We give condoms every room in the clinic, you will find that the is condoms so that each and every one who is coming to the clinic must have access of condoms. Then the other thing that is preventing HIV ahhh this thing of educating people who have HIV cos whenever we teach them about taking their medication at the right time and then regularly correctly, that help your HIV people to be viral suppressed. And if person is viral suppressed is not easy to spread the HIV. I think that thing it really works cos most of our people after educated they take their medication accordingly. You find that their partner they are negative and you find that they gave birth to healthy children.
33. I. Okay, from your experience could you please describe the different uptake of HIV prevention intervention in this area, what is the considered high and low uptake coverage?
34. P. Can you repeat the question.

35. I. From your experience could you please explain the uptake and coverage of the different HIV prevention intervention in this area?

36. P. Ahhh (thinking) I think the one that I have mention earlier on of condom use sage cos mostly we also educate the teenagers how comes to the clinic for family planning. Then we educate them about using a condom, so that they grow up knowing how to be responsible and how to protects themselves.

37. I. Okay thank you. What can be done to improve uptake and coverage of this intervention in area where indicators are low?

38. P. Ahhh (thinking) I think we should ahhh we must give each and every body who comes to the clinics especially the acute stream give them a counseling section so that they can be openly discuss whatever they want to discuss. About their health so that they can be taught how to protect themselves. So that it will the other platform to show them about the risk, how to protect themselves.

39. I. Okay, it time for us to close this part of interview, before we do this there is anything thing else you would like to add in this discussing. Please feel free to say anything, maybe there is something you want to add in this discussion we have discussed.

40. P. Okay, the other thing that I can add is that is the clinic we are still waiting for prep medication, we are busy educating people about prep I think prep is the other this that is going to help in our community to prevent HIV. Especially to young woman and to the ahhh (thinking) and man how have sex with man.

41. I. Okay, do you have man who have sex with man in this area?

42. P. Yes, yes.

43. I. So how do you handle that if you come across that?

44. P. I think most of the time we just treat them like other people, and we also educate them how to protect themselves from getting HIV, We don't discriminate them. We well come them so that they can feel free to ask whatever they want to ask. So that they also live a healthy life.

45. I. Okay. Thank you for your input in this interview, now we have come to the end of our section, name of interviewer XXX (Name of RA), thank you very much.

46. P. Thank you…
