# Supplementary material for: Understanding factors influencing utilization of HIV prevention and treatment services among patients and providers in a heterogeneous setting: A qualitative study from South Africa
Source: PLOS Glob Public Health. 2022 Feb 3;2(2):e0000132. doi: 10.1371/journal.pgph.0000132 (PMC10021737; doi:10.1371/journal.pgph.0000132)
Supplement: S1 Data — (ZIP) [file pgph.0000132.s001.zip › Supplementary information/IDI_Stakeholder_QSH003.pdf]

Full participant ID: QSH003

Participant Type: Female

Location: XXX (Name of Clinic)

Date: 20 July 2020

Primary interview language: English

Name of facilitator: XXX (Name of RA)

I: Participant number is QSH003 and the gender is female and we are, the type of an interview is the stake holder and how old are you ma’am?

P: 39

I: 39 and todays date is the 20th of July 2020 [Chair moving], ehh ma’am as we approached you about the interview. Ehh and then you have signed the consent form giving us the permission to interview you, so I would like a verbal agreement or a verbal consent, do you allow us to interview you?

P: Yes

I: Okay, ehh my name is (XXX Name of the interviewer) and then thank you for agreeing ehh to be interviewed by us. Ehh we are interested to hear about experience accessing the and providing health services related to HIV interventions in this clinic *mo* (at) (XXX Name of the clinic). Ehh do you have…, you do not have to answer the questions that you feel *gore* (that) you are not comfortable in answering. The is no right and wrong in this interview and then the interview ehh duration will take 30 to 45 minutes. [People talking outside] Ehh mca, Before we start, do you have any questions?

P: No I don’t have. ((laughing while talking)). [People laughing from outside]

I: Can you please ehh tell me your role in this clinic.

P: My role oh, I am a HIV counsellor, lay counsellor.

I: Okay and the starting time is 10:43am, so ehh how long have you been working in this ehh area? and how many clinics did you support? [People laughing outside]

P: No, I’ve been working here since from… yoh ((wondering)) 2012 and then

I: And then, how many clinics have you supported? Or you’ve been based…

P: I’ve been based here

I: Okay

P: Yes

I: And then ehh.. what sort of clinic services do you provide for the clinic operations in this area?

P: Sorry what?

I: The services that you provide

P: Oh HIV counselling and Index

I: Okay and what does in intail *haore* (when you say) HIV counselling and index, what is it that you do? In details

P: Ahh ((thinking)) in details?

I: Yes

P: I do HIV counselling testing

I: Yes

P: Mhm (yes) and then

I: Index, what is index?

P: Index… hha ((thinking)) index… mchaah ((thinking)) if i…i… I find a person HIV positive I asked him or her to come with her partner and her chil… and her children to come and test. If they are positive we ask that partner *na* (that) if, if he or she have another partner so its how we work

I: Interesting. Based on your experience what do you feel are the major issues affecting service delivery in this area? Or in (XXX Name of the Clinic)

P: Issues that’s affecting…

I: Service delivery

P: In (Name of clinic) clinic or…

I: Yah, or in this area and in (XXX Name of clinic) since *wena* (you) you are based in (XXX Name of clinic) but then if you know external out of ehh (XXX Name of clinic) but in this area *mo* (in) (XXX Name of the place) what could be the major issues that are affecting service delivery?. ((Someone interrupted the session by peeping in the room)) ehh (thinking) okay, I'm gonna rephrase my question or im gonna ask my question again since we were interrupted. Ehh ((preparing to speak)) based on your experience, what do you feel are the major issues affecting service delivery in this area.

P: Okay ehh… mhmm… ((preparing to speak)) lockdown, I think is lockdown. Ehh…mhmm…eish ((laghing softly)) in Mar.. April, March – April we… we didn't work. We stayed at home and there were no service delivery because we were at home, so its only that, that I can say.

I: Okay,

P: Mhm (yes)

I: And how has that impacted your patients?

P: Ahh…(thinking)

I: How did it affect your patients? By you guys not being able to deliver service to them.

P: Kekopa ome hape (can you please pause again) ((participant was attending attending a collegues phone message))

I: Okay, as I was saying, how did the ehh lockdown affect your participants?

P: Okay ehh…it affecte… it affected our participants because a person told him or herself that he or she is going to clinic to test and then she found out that the clinic is… or the counsellors are not there, so the person doesn't wanna come again. He wants to… to… to… to make a time wo… I don't know when but for now they are… they are not coming as usual as… yah (yes).

I: Okay. Alright so ehh will be going to the part two of our interview ehh existing health system in the area. So overall can you please describe your understanding of how the standard of health care system works? According to your own understanding.

P: How?

I: How the standard of health care system works, the health care system on in this facility, how does it work? [People talking outside]

P: ((participant was silent for 10 seconds)) mhm… eish… ((Unsure))

I: Remember there is no right and wrong. Yah so can you explain in your own, own understanding there is no right and wrong, its your own understanding, it is your own experience. Can you please just explain to me according to your own understanding, how does the standard of health care system works.

P: Okay uhmm… health care system here at (XXX Name of the facility) its fine but sometimes ehh… people are waiting from four o'clock. They are here from four o'clock and they… they leave here at four o.. p… they are here at 4H00am and they leave 4H00 pm so I where why why where is the problem there? Because they are sitting the whole day here.

I: Okay

P: And yah, it's just a the problem.

I: What are some of strengths and the weaknesses of those system? How…how… how is it working in a negative way and how is it working in a positive way?

P: Ahh… can you…

I: You said ehh the problem the the health care system ehh, they're they… the… the participant they suffer

P: Mhmm (yes)

I: Because they come in the morning

P: Mhmm (Yes)

I: And then they leave in the afternoon

P: Mhmm (Yes)

I: So you said 4H00am and then till 4H00pm, so how does that affect them positively? and how does that affect them negatively?

P: Positively, they don't affect them positively because they… they go home angry, they go home angry and then negatively, ((participant laughing softly)) negatively they… they… they fight here, swear at nurses and yah, they fight.

I: Okay. So don't they get the help at the end of the day?

P: Yes, they get… they get help.

I: Okay.

P: Mhmm (yes)

I: So if they come for treatment they get it even though it takes longer.

P: Yes.

I: Okay, ehh what can be done to ensure that the current system in (Name of the district) is strengthened. Like as you said cause they stay for longer time and they fight even though you say they do get help at the end of the day. The major question here is, how can that be improved? How can it… what is it that can be done to ensure that ehh the participant don't experience the negative things that you just said.

P: Maybe if they can add another stuff, maybe theres a sorta…shortat…sorta… *keng* (what) shortage of stuff. Yah (yes) if they… they hired another nurses maybe things will go right.

I: So the… the only thing is the staff?

P: The staff, Mhmm (yes)

I: Okay. And then in your experience can you please describe the HIV prevention intervention avail… available in this area.

P: HIV prevention?

I: Yes, Intervention, wha… what are the interventions that are available for HIV prevention?

P: Condom, to abstain, yah (yes). To use condom and to abstain, that’s the risk, those are the….,

I: Those are that mhm (yes).

P: Okay. And then ehh so according to you, do, like your participants, do they come for condoms?

P: Yes they do.

I: And do they use them? if so, how… how will you ensure that they are using them? Or what is it or what evidence that *wena* (you) as a health care worker have you seen or experienced to say that the participants are actually using condoms.

P: (Small laughter) A person cannot come from home and want condom if they are not using it, I am sure… I am sure *hore* (that) they are using it because they come from home and asking for… for the condoms.

I: Okay.

P: So that makes me sure *hore* (that) they are using condoms.

I: And then which types of condoms do you provide? Cause, okay, how many types of condoms do you at the facility?

P: We have two.

I: You have two.

P: Yah (yes)

I: And then…

P: Male condoms and female condoms.

I: And then do you… which ones do you provide or do you provide both?

P: Both

I: And which ones are being ehh asked more or which ones are being requested more?

P: Male Condoms

I: Male condoms compared to female condoms and what can be the cause of that?

P: They are used to ((small laughter)) man’s condom, they say… females mxca… famales they say that female condom they are not used to, they are used to female… male condoms

I: Okay.

P: So they are afraid of this ones, I don’t know how but they say the want this male… males condom.

I: Okay. And in what ways do you think the delivery of health care services is enabled in this facility? In which, in what way do you think the delivery of health care services is enable? (silence for 5 seconds) would you like me to repeat the question for you? Okay, In what way… ways do you think that delivery of health care service is enabled?

P: Ehh here at the clinic we have wobots, they go yoh, or *aka andestenda* (maybe I didn’t understand)? They go to the community serve them the… the… ehh mprr (lips sound, Thinking) *kana ke eng* (whats this)?

I: Service delivery

P: Service delivery *eya, eya* (yes, yes) we have… and then we have the mobiles that are in the field deliver *ethwena* (the thing)

I: So how do they deliver the service? Like I hear you saying they go to the community, what services that they render in while they are doing their work as wobots and then what are the services that the mobile clinic is delivering?

P: They are testing HIV, with the mobiles, all mobiles they are testing HIV and then the wobots also they are giving them test kits to test people who are not able to come to the clinic. They give them the test kits so that they can test them at home.

I: And how do they get the stats?

P: How do they get stats?

I: Yes if they are tesing at home, do they have a way of collecting data?

P: I don’t know how they… they are working but I know that they are testing at home but I don’t know how, how they

I: Okay

P: They collect the… the data.

I: Alright. Ehh from your experience, could you please explain the uptake and coverage of the different HIV preventions interventions in this area. What is considered a high and low uptake coverage?

P: Mhm (thinking) can you repeat the question.

I: Okay, Mnca ehh (preparing to speak) according to your own experience, can you please explain the uptake, like how do you get ehh the patients or the participants ehh of different HIV prevention interventions in this area and what is considered a high or a low uptake coverage.

P: How do I get participants?

I: Yes like in this area *ka yo* (with your) own experience the uptake *ya* (of) HIV…different infect HIV prevention interventions.

P: (Participant whispered) *Hei, aketsebe kereng* (I don’t know what to say). (door opening)

I: So im gonna repeat the question, ehh before we were interrupted, from your experience could you explain… could you please explain the uptake and coverage of the different HIV prevention interventions in this area. [Sound of a car in motion]

P: Ahh (thinking) ke stakile (I’m stuck) I don’t know how to explain.

I: Are you comfortable with answering this question?

P: No, I’m not.

I: You’re not

P: Hhmh (No)

I: So can we skip it? Okay. Ehh what can be done to improve uptake and coverage of these interventions in th… in areas where these indicators are low?

P: I can’t answer that.

I: Ohk, now we have gotten to the end of our session, ehh it is time for us to close it, this part of the interview but before we do, is there anything else about the topic that we haven’t discussed that you feel it is important that you want to talk about or there’s something that you we…you feel gore (that) we left out or something that you feel gore (that) we should have added, any additions or any comments with regards to our interview.

P: No, the are no comments

I: Alrihgt, Now we have come to the end of our discussion and thank you for participatin, if you have any questions about the study participation, please contact us, ehh remember ehh in the begging we… we told you before you signed the consent form about who we are and then where can you get hold of us, so if you have any questions then you can contact us. Ehh the time is now 11H09 when we finished our session, thank you.

P: Thank you
