# Supplementary material for: Understanding factors influencing utilization of HIV prevention and treatment services among patients and providers in a heterogeneous setting: A qualitative study from South Africa
Source: PLOS Glob Public Health. 2022 Feb 3;2(2):e0000132. doi: 10.1371/journal.pgph.0000132 (PMC10021737; doi:10.1371/journal.pgph.0000132)
Supplement: S1 Data — (ZIP) [file pgph.0000132.s001.zip › Supplementary information/IDI_Stakeholder_QSH005.pdf]

TYPE OT INTERVIEW: QUALITATIVE

DATE: 20 JULY 2020

INTERVIEWEE: CLINIC STAKEHOLDER

INTERVEIWER: XXX (Name of RA)

RA: Okay ah… Time eh 11:55 date 21 July 2020 interview with a female stakeholder eh qualitative interview. Eh thank you once again for your time eh we would like you to just confirm that you we, eh you agree to doing this interview, a recorded one with you.

QSH005: Yes

RA: Yes, you agree. Oh okay. We are interested to hear about your experiences neh in providing healthcare services related to HIV interventions in this clinic neh, eh you do not have to answer questions if you do not want too, the interview will approximately take 20-30 minutes, ey I want to remind that the information that you share here with us is confidential and what you say cannot be related to you and while the information is being gathered during this interview will be combined with other interviews neh.

QSH005: Mmm(yes)

RA: No one will know where, when, who said it and when it was said, where it saw said. There are not wright or wrong answers. We are interested in what you think and your experiences, please feel free to ask any questions if something is unclear neh.

QSH005: Mm(yes)

RA: So before we start do you have any questions?

QSH005: Mm not yet.

RA: Not yet.

QSH005: Mm

RA: Oh okay. Okay I`m just gonna ask few questions just about yourself just to break the ice yabo…

RA: Just tell me more about yourself mem.

QSH005: Professionally or personally?

RA: Mm let either or the one that you are comfortable with.

QSH005: Okay ah my name is (###### #####) well I hope you won`t use my name yah.

RA: No.

QSH005: I`m a case facilitator. I was hired by Aurum in early this year, so yah I started working here in around February. So, you wanna know what I`m doing?

RA: Yes, what you are doing in the clinic.

QSH005: Okay as a case facilitator what I`m doing ah I`m offering psycho-social support to new patients that are diagnosed with HIV and those who are already taking treatment for example if maybe as I`m monitoring the viral load also, if I see the viral load of the patient maybe who started the medication let me say last year and the viral load is still high. I do call that patient, try to find out what`s the issue maybe there is anything, something that wrong he or she is doing. Okay it either I do it telephonically or the patient come here in the clinic, we meet and we address the issue and hear what he or she is doing not taking or skipping the dose or taking traditional medicine whatever then yah I do adherence…

RA: Okay.

QSH005: To those who missed their appointment ah, I also what is it. I also do the follow ups, like with the new patient, when the new patient start let me say today ah within 48 hours I have to call the patient checking whatever ah he or she is feeling ah any side effects and give some advice and all those things yah. And also, with the appointments, monthly appointments I do remind them and also do thank them after they came to the clinic

RA: Okay so, where are you from before you came here?

QSH005: Originally, I`m a Sotho girl from XXX (Name of place), well yah it’s in XXX (Name of Province) but I don`t agree, it`s next to XXX (Name of neighbouring country) and also next to XXX (Name of province) so. I don`t know they say we fall under XXX (Name of province). Yah…

RA: So where are you currently staying?

QSH005: (Breathing) Currently I`m staying here in XXX (Name of place). I just moved in like two months ago.

RA: Okay as you are working here you moved in as you are working in this clinic?

QSH005: Yes

RA: How are you finding ease with this Corona thing, this Coronavirus thing? How are you finding your daily routine with this new normal of corona?

QSH005: Ah its hectic in a way that the patient eh don`t come to the clinic for their appointment when you call they will tell you that, they are in XXX (Name of province) and with the lockdown they can`t come here and they are defaulting yah. The challenge that we are facing now is the defaulters because of this Covid. However, some we do advise them to go to their nearest clinics and if they need to take bloods, they do take bloods and we do call that the clinic and give them the information of the patient and they give us the barcode so yah the patient is…

RA: Oh, okay so you advise them to go to their nearest clinic if they are in XXX (Name of province) they go to XXX (Name of province) clinic?

QSH005: Yah where they are.

RA: Oh, okay.

QSH005: Yah if they are in XXX (Name of province) they go to XXX (Name of province) clinic and if their cohort for instance it's time to take their 6 months blood and you know some clinics they don`t, if you just come with a card they don`t yah…

RA: They don`t help them?

QSH005: Yah so, we give their numbers, our numbers so the clinic call us and we confirm that is our patient and they, he`s there to take the blood and all the stuff yah..

RA: Okay so, that is your role?

QSH005: Yah, the other thing since this, the lockdown yah you can see the clinic is always full patient don`t want to fallow the que, they always calling, especially when I call them to remind. They want to go in.

RA: They just want to come in and jump the que?

QSH005: Mm(yes) so, everyday I have to go out because they are calling my, hey (XXX Name of participant) your people are here and I have to explain them and say wena(you) have to follow the que, wena(you) okay you can come in and you see people, they are starting to hate you, you know

all those things yah because you take others and leave them yah and they expect me to give their medication, I`m not a nurse so they don`t understand.

RA: So you just case managing them?

QSH005: I`m relaying on the nurses.

RA: As well?

QSH005: Yah. Especially if Mr. XXX (Name of person)is not here it is very difficult because you know yah. Nurses here the staff, they`ve got that thing that your patient, your Aurum patient so yah.

RA: Oh, okay so, we have heard about your services. Please describe for us eh your relationship with clinics in this area of operation.

QSH005: The clinics?

RA: Yes, the other clinics maybe.

QSH005: Ahh I can`t say much but ah, now that there is this corona, we do see the patient s from other clinics and help them to top up their medication maybe their clinic is closed. Even us they do go to the nearest, XXX (Name of place) for top up only for his blood they have to wait come when the clinic is open. So, I would say the relationship is yah, it`s good.

RA: Okay, with about, with the staff here in the clinic.

QSH005: Uzongibophisa wena(you will get me arrested).

RA: (Laughter) No no no…

QSH005: Some staff neh, yah some staff we get along together, they do assist but with the most of the staff, I don`t know they`ve got that thing Aurum is overtaking the clinic, where else we are working with the same patient, they are not Aurum patient they are XXX (Name of clinic) clinic patient. So I don`t know sometimes for example I had about in April neh, I had about 3 patient, they were tested HIV positive so they were needed to be initiated so, unfortunately Mr.XXX (Name of person) was on leave. I had to book those patients until Mr. (Name of person) came back because no one wanted to initiate and this thing is happening when Mr. (Name of person) is in XXX Name of place) they don`t initiate until…

RA: He comes back???

QSH005: Yah until he comes back.

RA: So in your view, how is the relationship between you and the clinic staff?

QSH005: The Aurum and the clinic?

RA: Yes.

QSH005: Ay (No) siya fosta (we are forcing). In my view the way Aurum ngathi iyafosta (like forcing) to work with the clinic.

RA: Mmm…

QSH005: Because even with the facility manager there are times when he will call the staff meeting but she will say not Aurum Staff. I only need my staff. You know that thing might….

RA: Oh its separates like.

QSH005: It discriminate like, you know we don`t belong here but we are also working here.

RA: Mmm…

QSH005: So when you give the, first time when you give the PPEs, she only said for my staff luna (you), Aurum will give yours. So you know but we are working together endlini eyodwa (in the same house) with the same patients so I don`t know.

RA: Oh…

QSH005: Yah so the relationship I can say angazi iba nalokho (I don't it has that)..

RA: Okay

QSH005: Its on and off cause some of the amanye (other) sister, they do understand they do treat us equally as DOH staff yeah I can give you an example; Makuqalwa ukuthesta (At the beginning of testing) this corona thing the facility manager said the only staff that will be tested is her staff and one of the nurses fought about this and said no nna (I) am going to test everyone here osebenzala (who works here) clinic. So there was that tension between. But at the end sagcina sithestile ke (we ended up testing)

RA: Based on your experience and what I have heard from you what do you feel are the major issues that affects the service delivery in your facility?

QSH005: Mmm… Is this thing of Aurum abantu base (people from) Aurum you are your patient they are not our patient.

RA: So separating patients?

QSH005: Yah yah because if you ask for assistance maybe the patient came and then like I said maybe #### is not here and then I go to a sister maybe the patient I call the patient for blood only.

RA: Mmm…

QSH005: The patient needs the date for bloods not because of the patient because of the date they gave the patient.

RA: Mmm…

QSH005: So I will call the patient so you were due for bloods in May then the patient will say no I was there in May but they didn`t take bloods and say please come you will be coming only for bloods so if #### is not here I will go for assistance to another nurse. They will tell me that tell the patient to go start the que so you know eish yah those are the issues so now patient will say hey wena (You) called me and said for the bloods I can`t go start the que there by the robots only for bloods and I still have medication. Yah so that the relationship for me. The DOH staff or the, Alberton staff sometimes they give us hard time especially when we are dealing with patients.

RA: Okay. Okay thank you for that. So now we gonna go to the part two the existing health systems in this area. Overall can you describe your understanding on how standard health system works in this area.

QSH005: Mm. Yah no I don`t know I will be lying but with the HIV I know when the patient is tested positive today you need to be initiated today and start taking medication today.

RA: Okay

QSH005: So yah…

RA: Okay so with what you know what are some of the strength and weakness with what you know?

QSH005: Mmm… Okay the strength….. I can`t think of any strength.

RA: Okay…

QSH005: Because most of the time they are relying to Aurum to initiate the new patients so I don`t know. That is not the strength.

RA: So you can simply say that the strength is Aurum is helping.

QSH005: Yes yah yah I can say that Aurum is very helping the clinic even with the capturing. If you can go there in the filing ehhh sometimes the files are missing especially if Aurum staff are not there it’s a chaos in the filing room, so yah Aurum, I will say Aurum is the strength.

RA: Okay the weaknesses?

QSH005: E… (Laughter) yah the weaknesses I don`t know, I don`t know.

RA: Mmm…

QSH005: I can`t think of any right now.

RA: What can be done to ensure the current healthcare systems in XXX (Name of District) strengthen, what do you think can be done to health system of XXX (Name of District)?

QSH005: Nna (I) my opinion I will say if i-Aurum can have all, the other thing is the lack of space if Aurum can have la ema (here at) the clinics can have I don’t know something like a mobile clinic or a caravan or something so that the clinicians or the enroll nurses can have space to take bloods.

RA: Mmm…

QSH005: I think that can affect a lot and also sometimes I do understand the pressure, the stuff nurses the nurses here, staff of here are feeling because he has a long que and take lunch very late and its tiring so if they can, Aurum maybe can place that thing outside not a gazebo but the caravan or something.

RA: Mobile..

QSH005: Yah so that every patient who come for bloods goes this side whether its three months, six months whatever. They should be this side there should be assistant nurse of staff nurse who will be taking the bloods and the clinician can look only the initiations because there is a long process because it takes about an hour, you start by counselling the patient and they will start crying and all that and give you the issues and you start to attend and all that so yah if Aurum can assist with that in every clinics I think cause the space is not enough here.

RA: Okay so now we gonna be going to part three neh your experiences with provision of HIV prevention services neh. In your experience can you please describe the HIV prevention interventions available in this area…

QSH005: Yoh okay before this corona thing there were having campaigns going to the community, give ama health talks and issuing the condoms.

RA: Mmm…

QSH005: Now because of the corona they are no longer campaigning however the patient when come in and tested negative neh.

RA: Mmm…

QSH005: And the patient, you ask the patient what’s the reason you came here for , why do you test and they will tell you maybe last night I had sex with someone I don`t know or maybe the partner or the condom bust or then we do give PreP for prevention.

RA: Okay so in the clinic they do provide PreP?

QSH005: Yah yah yah that’s it that’s what I know.

RA: In what ways do you think the delivery of healthcare system is enabled?

QSH005: In what ways?

RA: Yes is enabled, is the delivery of healthcare system is enabled?

QSH005: Can you clarify that I don`t understand.

RA: Mmm the question. Okay in what ways are the health services being given, like given to people..?

QSH005: Ahh the patient come to the clinic they get attended, they get the service they are looking for yah.

RA: From your experience could you please explain the uptake and coverage of the HIV prevention in this area and what is considered high or low up to an average.

QSH005: Ay lemi buzo yakho angisayi (these questions of yours I don`t) understand.

RA: Okay so in your experience neh can you tell us when the prevention services are high like when the services are high given in high numbers to people ehh does that happen in this area? Whereby prevention majors are given to people and in high numbers and whereby they cover a large number of people?

QSH005: Yah it does happen as you can see there is a lot of patients outside, through ut the day until 4 o`clock, until they close and say no its cut off and the cut off is around 4 so yah they do, yah it does happen and also with PreP there are lot of patient that are coming for PreP. As now they also know

that PreP is also a service here. There are lot of patient that are coming and request the PreP yah.

RA: Alright. Okay.

QSH005: Yah….

RA: Okay, so what can be done to improve the uptake and coverage of these interventions in this area and the indicators, where these indicators are low?

QSH005: Ahh I think more staff.

RA: Mmm…

QSH005: There`s a shortage of staff not only Aurum, but the whole clinic the nurses.

RA: Oh the clinic staff?

QSH005: Yah there`s a shortage of clinic staff and also shortage of space and the clinic is small.

RA: Mmm oh. So that you can improve the number of people that are seen?

QSH005: Yes and the other thing that I think they should do…

RA: Mmm..

QSH005: I think this will come to the weaknesses, remember that question ….

RA: Yah the question about the weaknesses.

QSH005: Yah XXXX (Name of clinic) is accommodating people outside it due restriction, there are people from XXX, XXXX (Names of places) you know all those townships.

RA: Townships around here?

QSH005: Yah and then those people when you call them, they miss their appointments. When you ask them what happened, what is happening? The tell you they don`t have imali (money).

RA: Transport money?

QSH005: Exactly, then you ask them then okay how about we do transferee then to your nearest clinic where you won`t use transport? They don`t want to.

RA: They don’t want use their nearest clinic?

QSH005: Yah so now sisala sinama (we are left with) defaulters alekude (that are far). We can`t even

trace them cause they are outside of…

RA: Demarcated area?

QSH005: Yes. So that is the weaknesses zase(of) XXX (Name of Place). Is because of abantu(people) from outside so I wish they could like when the person, okay I know some people are working here so they prefer to come here because they are working here but there those who just they are not even working here they just come here just because they say XXX (Name of suburb). No one will see them you know those things.

RA: Because of stigma that is attached to HIV?

QSH005: Yah.

RA: Oh okay.

QSH005: And the other weaknesses the nurses here they don`t want to decent patients here I don`t know whether they don't have time or they don't check ama(the) results or they don't know when to decent a patient. I don`t know but I`ve got a long list of patients their viral load is undetectable but they still come to the clinic and I`ve been issuing that list. I think out of 48.

RA: Mmm…

QSH005: Not even half were decanted so ahh whether they don`t have time to do that process or angazi(I don`t know).

RA: Do they know the processes though?

QSH005: They know cause Mr.XXX (Name of person) did went to them and tell them even the facility manager and send that I don't know whether is a memo or what.

RA: Yes memo

QSH005: Yah that was saying the, explaining the..

RA: The procedure.

QSH005: The procedure the patient, which patient are eligible for decanting.

RA: Mmm…

QSH005: We all did that. As you can see the clinic still has a lot of patient. Most of those patient are ART and their viral load are suppressed but they are still here.

RA: Oh okay we are about to end the session neh.

QSH005: Oh thanks.

RA: We are about to end the interview but before we do so is there anything about the topic that we discussed that you feel its important to say?

QSH005: All I can say is please please please, you need to do something as you are doing these studies gaining experiences I`m sure you got the experiences of the patients and other things do something with those experiences we have shared with you.

RA: Okay now we have come to the end of our discussion, thank you for your participation.

QSH005: Siyabonga(Thank you)

RA: Thank you.
