# Supplementary material for: Understanding factors influencing utilization of HIV prevention and treatment services among patients and providers in a heterogeneous setting: A qualitative study from South Africa
Source: PLOS Glob Public Health. 2022 Feb 3;2(2):e0000132. doi: 10.1371/journal.pgph.0000132 (PMC10021737; doi:10.1371/journal.pgph.0000132)
Supplement: Attachment — Submitted filename: Response to reviewers.docx [file pgph.0000132.s002.docx]

16 August 2021

*PGPH-D-21-00055*

*Understanding factors influencing utilization of HIV prevention and treatment interventions among patients and providers in a heterogeneous setting: a qualitative study from South Africa*

**Here is a point-by point response to the comments raised by the reviewers and the exact change in the manuscript (italicized).**

**Editor**

I appreciated the opportunity to review this well-written manuscript. I encourage you to address all of the reviewers' comments below, particularly concerning the methods and discussion section. In addition, Reviewer #2's point about how setting/sampling seems to disappear from the paper after the methods is important. You spend some time setting up the sampling approach in the methods, but then do not reference it again.

We have revised the results and discussion sections to show the differences and similarities in the settings. We have provided this summary in Table 3.

The authors should clarify their use and definition of phenomenology. Typically, phenomenology requires more than one-time in-depth interviews with participants. There is usually much deeper involvement with participants (e.g. multiple interviews, participant observation, participatory methods), as well as more iterative and inductive coding and memo-ing. If this was the case for this study, then please revise the methods to reflect this, and raise this in the discussion. If not, then I would drop the phenomenological label.

Thank you for this comment. During literature review, I came across an article that utilised this method despite not conducting a series of interviews with the same individuals but in light of this comment, the reference to phenomenological approach has been dropped to avoid misleading the targeted audience (see Page 4 Line 65).

Although I appreciated the concision of the discussion, I agreed with the reviewers that a little more engagement with the literature would be helpful. In addition to their recommendations, how do your findings relate to the recent large-scale treatment as prevention/UTT/test and treat trials that have been conducted in South Africa and other countries?

We thank you for the comment that requires more engagement with existing literature. We also appreciate the suggested articles. The discussion has been revised to relate the findings with the large-scale UTT programmes and studies conducted in South Africa and other countries.

**Reviewer 1**

1. Abstract, line 2: progress” or advances/innovations?

In Line 2, I have replaced “progress” with “advances”

2. Abstract line 16: “inadequate know-how on effectively using condoms”. I’m not convinced this is the best or appropriate phrasing

In Line 16-17, I have revised “inadequate know-how on effectively using condoms” with “sub-optimal condom use”

3. Page 2, line 31: “proponents” does not seem the best choice of word here – they provide the package, but aren’t necessarily active advocates of it

In Line 32, I have replaced “proponents” with “providers”

4. Page 5, line 98: Sentence doesn’t make sense. Revise

This is not a sentence but a caption for Fig 2: In line 98 – I have revised the caption to Fig 2 to read “Flow diagram showing the recruitment strategy and enrolment of participants for the study

5. Page 5, line 100: more detail on study languages would be helpful. Provide some context of the socio-cultural setting, ethno-linguistic characteristics of participants etc

The study languages (English, Sesotho, Setswana and IsiZulu) have been included on Page 5, Lines 105 &106)

The socio cultural setting of Ekurhuleni district is discussed under study setting, Page 4, Lines 85-87.

*…EMM has a predominantly black male population (51.2%), and almost 25% live below the poverty line (earn less than ZAR 992 per month) in overcrowded informal settlements on the urban periphery with limited access to job opportunities and adequate social infrastructure”*

6. Page 6, line 132: explain the term “finer coding”

Finer coding referred to as grouping the initial codes into categories/key dimensions and subsequently organize them into themes. This revision is made on Page 6, lines 147 and 148

*“…to group the initial codes into themes and subsequently organize into key dimensions and identify patterns across groups.”*

7. Page 7, line 155: How was gender of participants determined? Were they presented with binary categories to self-select? Were there any other gender categories available? Was this self-identified gender?

Gender was self-identified when participants were asked to “tell us more about yourself?” During the interviews, we did not use a demographics questionnaire used to collect this data. We however asked the question “Tell us more about yourself”, in the interview guide and we were able to get the demographic information from the responses provided during discussions. This has been elaborated with the inclusion of line 165-166 on Page 7.

8. Page 7, line 155: define the term “single” as a characteristic – how was this defined for participants and how were people characterised?

The term single was referred to as those not married. This has been corrected in line 177 of Page 7.

9. Page 7, line 156: how was HIV status determined? Self-reported?

HIV status was self-reported. This has been corrected in Table 1.

10. Page 7, Line 60: how did the participant self-identify as MSM? Did he disclose having sex with other men, or did he self-identify with the label “MSM” ?

The participant self-identified with the label “Deputy-person” which he explained it as being an “MSM” during the interviews.

11. Discussion: more engagement with the dynamics of condom use and condom decision making might be helpful, particularly when discussing the gendered aspect of condom use and motivations – see suggested literature below.

Thank you for this comment and the suggested literature. We have discussed the dynamics of condom use or lack of from a male and female perspective and suggested recommendations to increase condom use frequency based on findings from existing literature. The revisions are on Page 20; 435-443 and Page 21; Lines 447-454.

12. Discussions of condom use also lack acknowledgement of different types of condoms, male/female, and government provided, bought condoms etc.

The revisions are on Page 20; 435-443 and Page 21; Lines 447-454.

13. What about patient perspectives of PrEP side effects? Fears o around PrEP use, stigma of PrEP being seen as ART etc -engage more with the current literature on PrEP

None of the participants mentioned about side-effects from taking PrEP and this was not probed but we have highlighted that in other studies, side effects were a major concern when using PrEP for HIV prevention (Page 20; Lines 431-437).

14. Demonstrate awareness of complexity of HIV risk perception, how this links to HIV cascade – i.e. fear, lack of concern, belief that getting HIV is not an issue since there are ARVs. Complexity of risk perceptions and motivations around HIV prevention.

This comment has been addressed on Page 19; Lines 406-409. It is one of the main findings of this study. HIV risk perception motivated engagement in care but to sustain this engagement, personal, relational dynamics need to be examined to avoid potential infection or poor health outcome along the cascade

*“ … In high uptake settings, there was high HIV risk perception which seemed to motivate engagement in HIV services. Findings highlight examining personal and relational dynamics which are likely to influence disengagement along the HIV care continuum.”*

15. Discussions around social support could benefit from acknowledgment of need for holistic interventions addressing psycho-social aspects related to HIV as well. What about mental health and wellbeing, links to treatment adherence and HIV cascade?

Thank you for this suggestion. Reference to incorporation of mental health/well-being programmes into existing community-based programmes for good HIV outcomes has been included on Page 21; Lines 459 - 462.

*“….Expanding programs that address the lack of social support from a psychosocial perspective are likely to improve health outcomes. Individual and family-centred counselling, and peer support while implementing mental health services have improved linkage to and retention in care among PLHIV.”*

16. Discussion around complexity of HIV related stigma, and efforts to reduce stigma- i.e. resulting in lack of care about getting infected with HIV? HIV stigma in South Africa is not static – it is ever changing and adapting to new HIV prevention and treatment landscape. Authors need to demonstrate an awareness of the fast changing context, and how HIV stigma is situated in this. Framing of “need to reduce stigma” is too simplistic, and needs to be unpacked.

Thank you for pointing this out. In this paper, we suggest the use of community based approaches which have been effectively used to address stigma at a community level (Page 22 489-492)

*“…..Community-based interventions including integration of HIV services into existing programs can reduce stigma through sensitization by community healthcare or peer workers and is likely to improve uptake of HIV services, thus leading to higher ART coverage and viral load suppression*.”

17. Authors could demonstrate a closer engagement with the literature. Some suggested readings below:

- Atujuna, M., Newman, P. A., Wallace, M., Eluhu, M., Rubincam, C., Brown, B., & Bekker, L.-G. (2018). Contexts of vulnerability and the acceptability of new biomedical HIV prevention technologies among key populations in South Africa: A qualitative study. PLoS ONE, 13(2), e0191251–17. <http://doi.org/10.1371/journal.pone.0191251>
- Duby, Z., Jonas, K. McClinton Appollis, T., Maruping, K., Dietrich, J. & Mathews, C. (2021) “Condoms Are Boring”: Navigating Relationship Dynamics, Gendered Power, and Motivations for Condomless Sex Amongst Adolescents and Young People in South Africa, International Journal of Sexual Health, 33(1). DOI: 10.1080/19317611.2020.1851334
- Duby, Z., Jonas, K. McClinton Appollis, T., Maruping, K., Dietrich, J., Vanleeuw, L. & Mathews, C. (2020) “There is no fear in me … well, that little fear is there”: dualistic views towards HIV testing among South African adolescent girls and young women, African Journal of AIDS Research, 19(3). DOI: 10.2989/16085906.2020.1799232
- Gause, N. K., Brown, J. L., Welge, J., & Northern, N. (2018). Meta-analyses of HIV prevention interventions targeting improved partner communication: effects on partner communication and condom use frequency outcomes. Journal of Behavioral Medicine, 41(4), 423–440. <http://doi.org/10.1007/s10865-018-9916-9>
- Hartmann, M., McConnell, M., Bekker, L.-G., Celum, C., Bennie, T., Zuma, J., & van der Straten, A. (2018). Motivated Reasoning and HIV Risk? Views on Relationships, Trust, and Risk from Young Women in Cape Town, South Africa, and Implications for Oral PrEP. AIDS and Behavior, 22(11), 3468–3479. <http://doi.org/10.1007/s10461-018-2044-2>
- Hendrickson, Z. M., Naugle, D. A., Tibbels, N., Dosso, A., Van Lith, L. M., Mallalieu, E. C., et al. (2019). “You Take Medications, You Live Normally”: The Role of Antiretroviral Therapy in Mitigating Men’s Perceived Threats of HIV in Côte d’Ivoire. AIDS and Behavior, 23(9), 2600–2609. <http://doi.org/10.1007/s10461-019-02614-5>
- Irungu, E.M, Ngure, K., Mugwanya, K.K., Awuor, M., Dollah, A., Ongolly, F., Mugo, N., Bukusi, E., Wamoni, E., Odoyo, J., Morton, J.F., Barnabee, G., Mukui, I., Baeten, J.M. & O'Malley, G. & the Partners Scale-Up Project Team. (2020). “Now that PrEP is reducing the risk of transmission of HIV, why then do you still insist that we use condoms?” the condom quandary among PrEP users and health care providers in Kenya. AIDS Care. <http://doi.org/10.1080/09540121.2020.1744507>
- Lambert, R. F., Orrell, C., Bangsberg, D. R., & Haberer, J. E. (2018). Factors that Motivated Otherwise Healthy HIV-Positive Young Adults to Access HIV Testing and Treatment in South Africa. AIDS and Behavior, 22(3), 733–741. <http://doi.org/10.1007/s10461-017-1704-y>
- Legemate, E. M., Hontelez, J. A. C., Looman, C. W. N., & de Vlas, S. J. (2017). Behavioural disinhibition in the general population during the antiretroviral therapy roll-out in Sub-Saharan Africa: systematic review and meta-analysis. Tropical Medicine & International Health, 22(7), 797–806. <http://doi.org/10.1111/tmi.12885>
- Ng’eno, B., Rogers, B., Mbori-Ngacha, D., Essajee, S., Hrapcak, S., & Modi, S. (2020). Understanding the uptake of prevention of mother-to-child transmission services among adolescent girls in Sub-Saharan Africa: a review of literature. International Journal of Adolescence and Youth, 25(1), 585–598. <http://doi.org/10.1080/02673843.2019.1699124>

Thank you for these examples of previously written work. We have synthesized findings from these articles to augment our findings, expand the arguments and suggest additional recommendations and relate findings to the recent large-scale treatment prevention/UTT/test and treat trials that have been conducted in many settings.

1. About our finding on adherence to PMTCT, further engagement with the literature from a systematic review highlighted led us to understand that despite the favourable finding (uptake of this HIV service), there is a population (adolescents and young women which is often delayed into care due inadequate knowledge to navigate the health system and stigma from consequences of HIV positive test result by healthcare workers, family and community (Page 20: Lines 423-430). Psychosocial support from healthcare workers and peer education is encouraged to increase utilization of services by these population (Page: 21; Lines 460-463).
2. More discussion on PrEP use and lack of use and the reasons including pill burden and side effects has been included on Page 20: Lines 432-438.
3. Dynamics of condom use has been unpacked on Page 20 from Lines 438-444.
4. Recommendations on improving uptake have been explained on Page 21 Lines 448-455, Page 22; Lines 490-493, and Page 23; Lines 530-534.

**Reviewer 2**

How was the actual recruitment of participants done? When the researcher approached participants at the queue in the HIV clinic, what was their reaction? Were there feelings of stigma? Were there difference between recruiting participants through the health providers versus by directly approaching them as they queued for their routine visits? Why were two strategies used to recruit participants?

The recruitment was done at the clinics, and RAs approached patients as they queued for their visits as not all participants were allowed to be in the clinic due to COVID-19 protocols. The participants referred to us by HCWs were mainly those newly initiating on ART after testing HIV positive on the day of recruitment (See Page 5; line 108). There were no feelings of stigma shown by the participants during recruitment.

What do the authors mean by “final sample of 30 participants was diverse enough”? How diverse was this final sample? What do you mean by diverse? Diversity based on what? Please explain.

The diverse sample consisted of HIV-positive and negative patients, both male and female, stable on ART and newly initiating. The sample also included health care providers at the clinic and community level. This diversity has been explained in lines 116-122.

*“…Consenting participants were assigned a unique study number for confidentiality. The final sample of 30 participants included male and female PLHIV, patients who were newly initiated on ART, HIV negative patients, and HCWs providing different HIV services at the clinic and community-level. The variedness of this ultimate sample would enable obtaining a fairly comprehensive picture about experiences and perceptions related to uptake and delivery of HIV interventions.”*

How did the authors develop the coding framework?

Coding framework was developed by adapting the Social Cognitive theory to explore perception of patients seeking and HCWs providing HIV services in routine healthcare settings (Pg. 6 Lines 146-148)

Were there any difference between the HIV positive and negative participants in terms of a particular perspective as presented in the themes? How about differences in self-esteem and utilization of services? Were there differences in motivations for using HIV services by HIV status or by setting?

Table 3 below shows that there were no differences by setting in terms of utilizing HIV services. The motivations from HIV positive participants was to reduce their viral load to an undetectable level and not to transmit to sexual partners or babies. Those HIV negative utilized HIV services to prevent infection. We did not probe on self-esteem. This has been explained on Pages 19 and 20 in Lines 413 to 433.

The authors mention that the married women find it difficult to use condoms and other HIV services. Did the partners of these women know that they were HIV positive? How about the status of the partners for the married women? Was the status for the men known to the couple?

Our findings show that married women find it difficult to use condoms due to the stigma attached to it. The female participants with stable partners in our study knew their partners HIV status and would often visit the healthcare clinic together for counselling (Page 17; lines 341-342). The partners would agree to use them during counselling sessions but changed their minds when they got home.

It seems like the participants interviewed had good access to HIV services and as mentioned in the manuscript had a regular supply of ART. What implications do these findings have beyond the study settings, especially in places where supplies may be irregular?

Thank you for this observation. These findings cannot be generalized to settings with irregular supply and on Page 23; Lines 530-534, we highlight the need for the healthcare system to include HIV prevention supplies as part of their essential commodity security plans in expanding access to these services. On-going monitoring and evaluation is needed to plan for further improvements in implementation of HIV services.

In addition to working with health providers and those infected with HIV to reduce stigma, there is also stigma experienced within the community. Based on their findings, what do the authors suggest should be done to reduce stigma at the community level?

Community-based interventions including integration of HIV services into existing programs can reduce stigma through sensitization of community members. This is likely to improve uptake HIV services such as higher levels of ART coverage and viral load suppression. Page 22; Lines 490-493.

The differences in setting sampled from did not reflect clearly in the findings and the discussion. It would be useful for the authors to present similarities and differences in accounts by settings so that readers can clearly see how the findings are a reflection of the heterogeneous settings as mentioned in the methods and discussion sections.

 Thank you for this observation. We have included Table 3 in this revised manuscript that provides a summary of the similarities and differences in themes and key dimensions across the three different settings.

Table 3: Overview of similarities and differences in themes across geographical setting

| **Theme and key dimensions** | **HL** | **LH** | **LL** |
| --- | --- | --- | --- |
| **Theme 1: Benefits from adopting HIV prevention or treatment services** | | | |
| Being in HIV care while pregnant for positive pregnancy outcomes including HIV negative children |  |  |  |
| Suppressed viral load as a result of consistently taking ART |  |  |  |
| Remaining HIV negative by using PrEP or PEP in case of unprotected sexual acts and protected sexual acts by condom use |  |  |  |
| **Theme 2: Potential negative sequelae from utilising HIV prevention or treatment services** | | | |
| Fear of testing HIV positive which may lead to anticipated or enacted stigma |  |  |  |
| Accessing and using condoms especially for female patients which may lead to anticipated or enacted stigma or violence |  |  |  |
| Concerns from providers when patients do not use condoms in favour of PrEP or when patients fail to seek healthcare due to proximity to healthcare facilities or prolonged waiting times |  |  |  |
| Side effects from ART that may interrupt or stop utilisation of HIV treatment |  |  |  |
| **Theme 3: Well-informed about available HIV prevention or treatment services** | | | |
| Having knowledge on HIV and available interventions, which affect utilization of HIV prevention or treatment interventions such as PrEP and PEP. |  |  |  |
| Using information and skills obtained during pre and post-test HIV counselling, health promotion activities or campaigns to remain in HIV care |  |  |  |
| Attitudes towards utilizing HIV prevention or treatment services |  |  |  |
| **Theme 4: Ability to correctly use the available HIV prevention or treatment services** | | | |
| To correctly and consistently use condoms for HIV prevention |  |  |  |
| Taking ARVs to sustain an undetectable viral load and prevent death |  |  |  |
| **Theme 5: Social or environmental dynamics and conditions supporting utilization of HIV prevention or treatment services** | | | |
| Drawing inspiration from peers who have lived positively despite being HIV positive |  |  |  |
| Social support from family members to continue utilizing the available HIV prevention or treatment interventions |  |  |  |
| Ensuring supply of condoms in healthcare facilities and in the communities |  |  |  |
| Structural support by friendly clinic staff who encourage utilization of interventions including to key populations |  |  |  |
| Differentiated care delivery of interventions for stable ART patients to overcome barriers associated with staff shortages and lengthy queues |  |  |  |

- Denotes similarities across settings
