# Supplementary material for: Understanding factors influencing utilization of HIV prevention and treatment services among patients and providers in a heterogeneous setting: A qualitative study from South Africa
Source: PLOS Glob Public Health. 2022 Feb 3;2(2):e0000132. doi: 10.1371/journal.pgph.0000132 (PMC10021737; doi:10.1371/journal.pgph.0000132)
Supplement: Attachment — Submitted filename: Response to reviewers.docx [file pgph.0000132.s003.docx]

06 November 2021

Editor

PLOS Global Public Health
[1160 Battery Street
Koshland Building East, Suite 225
San Francisco, CA 94111, USA](https://goo.gl/maps/SNG0D)

Dear Dr. Marie A. Brault, PhD,

Thank you for the opportunity to revise our submitted manuscript *‘Understanding factors influencing utilization of HIV prevention and treatment interventions among patients and providers in a heterogeneous setting: a qualitative study from South Africa’.*

The suggestions offered by the reviewers have been immensely helpful. We have addressed the reviewer comments and responded to them individually. The revisions have been approved by all authors and I have again been chosen as the corresponding author. The changes are marked in red in the response to reviewers’ document as requested, and the revised manuscripts are uploaded.

We have taken the following into consideration:

1. We used PACE to ensure that the figures meet journal requirements.
2. We have checked that the reference list is correct and accurate and no changes have been made.

Please do not hesitate to contact me if you require any additional detail.

Sincerely,

Lucy A Chimoyi, B.Sc. (Biochem), MSc. (Epi)

Senior Research Manager, Implementation Research Division

The Aurum Institute

29 Queens Road

Parktown, Johannesburg

South Africa

Telephone: (+27) 11- 590 1360

Email: [LChimoyi@auruminstitute.org](mailto:LChimoyi@auruminstitute.org)

*PGPH-D-21-00055*

*Understanding factors influencing utilization of HIV prevention and treatment interventions among patients and providers in a heterogeneous setting: a qualitative study from South Africa*

**Here is a point-by point response to the comments raised by the reviewers and manuscript changes.**

Reviewer #1: This manuscript is much improved. The authors have made a great effort at engaging with the literature, and have made some important clarifications around terminology employed and methodology. Additional comments:
P2
-line 8 & 17: Clarify what you mean by “areas”

We clarify that “areas” in this context refers to “geographical areas.” This word has been replaced in lines 8 and 18. This clarification has been made in other the text in lines 93,354, 362 and 370.

-line 26: human resources – rephrase to “staff”/ “health care providers”?

“Human resources” in lines 27 has been replaced with “healthcare providers”.

P4
-line 81: Gauteng Province, “of South Africa”

The words “of South Africa” have been added at the end of the sentence in line 82.

-line 85: provide US$ equivalent for international readers

The equivalent for ZAR 992 is ≈ US$64 and this has been provided in line 87.

P5, line 104: perhaps just say that data were collected in July 2020?

Thank you for the correction. The words in “July 2020” have replaced “between July 4 and July 27 2020” in line 105-106

P6, line 138: surely not “back translated” into English as English was not the original language?

Thank you for the observation. The statement in line 139 reads “were translated into English”.

P21, line 442: in addition to being good quality, provided condoms should “desirable” – government issued condoms are often viewed as undesirable as they “smell bad” / too thick etc.

Thank you for this suggestion. We have included in addition to good quality that condoms need to desirable to the end-user in lines 445 and 446.

P22, Line 485: clarify what “this misinformation” refers to
Misinformation has been replaced with the following phrase in line 489 “…misconceptions about existing HIV prevention strategies.”

Re. my previous comment:
- How was gender of participants determined? Was this self-identified gender?
authors responded:
“Gender was self-identified when participants were asked to “tell us more about yourself?” During the interviews, we did not use a demographics questionnaire used to collect this data. We however asked the question “Tell us more about yourself”, in the interview guide and we were able to get the demographic information from the responses provided during discussions". - I am still troubled by this – interviewers categorised participants into genders by their own interpretation of what participant said, or assumption about the person’s gender? Unless ppt clearly stated when asked to “tell about yourself” – that “as a woman” / “as a man”. Methods section should include statement about how gender was categorised, by whom

Re response: “participant self-identified with the label “Deputy-person” which he explained it as being an “MSM” during the interviews.” -Did the participant state that “deputy-person” meant a “gay man” or a man who has sex with other men? Be specific and careful using these categories.

Thank you for raising this comment again. In the methods section lines 160-162, we have included a statement about the categorization of gender as suggested.

*“We categorized gender based on the responses from the question: “Tell us more about yourself,” When the participant explicitly mentioned and voluntarily mentioned their gender as either male or female without probing.*

In this case, the participant who described himself as a “deputy-person” indicated that he was MSM. In line 351, the acronym LGBTQ has been replaced with MSM to reflect this explanation.
