# Supplementary material for: Understanding factors influencing utilization of HIV prevention and treatment services among patients and providers in a heterogeneous setting: A qualitative study from South Africa
Source: PLOS Glob Public Health. 2022 Feb 3;2(2):e0000132. doi: 10.1371/journal.pgph.0000132 (PMC10021737; doi:10.1371/journal.pgph.0000132)
Supplement: Attachment — Submitted filename: Response to reviewers.docx [file pgph.0000132.s004.docx]

09 November 2021

Editor

PLOS Global Public Health
[1160 Battery Street
Koshland Building East, Suite 225
San Francisco, CA 94111, USA](https://goo.gl/maps/SNG0D)

Dear Dr. Marie A. Brault, PhD,

Thank you for the opportunity to revise our submitted manuscript *‘Understanding factors influencing utilization of HIV prevention and treatment interventions among patients and providers in a heterogeneous setting: a qualitative study from South Africa’.*

The suggestions offered by the reviewers have been immensely helpful. We have addressed the reviewer comments and responded to them individually. The revisions have been approved by all authors and I have again been chosen as the corresponding author. The changes are marked in red in the response to reviewers’ document as requested, and the revised manuscripts are uploaded.

We have taken the following into consideration:

1. We used PACE to ensure that the figures meet journal requirements.
2. We have checked that the reference list is correct and accurate and no changes have been made.

Please do not hesitate to contact me if you require any additional detail.

Sincerely,

Lucy A Chimoyi, B.Sc. (Biochem), MSc. (Epi)

Senior Research Manager, Implementation Research Division

The Aurum Institute

29 Queens Road

Parktown, Johannesburg

South Africa

Telephone: (+27) 11- 590 1360

Email: [LChimoyi@auruminstitute.org](mailto:LChimoyi@auruminstitute.org)

*PGPH-D-21-00055*

*Understanding factors influencing utilization of HIV prevention and treatment interventions among patients and providers in a heterogeneous setting: a qualitative study from South Africa*

**Here is a point-by point response to the comments raised by the reviewers and manuscript changes.**

Reviewer #1: This manuscript is much improved. The authors have made a great effort at engaging with the literature, and have made some important clarifications around terminology employed and methodology. Additional comments:
P2
-line 8 & 17: Clarify what you mean by “areas”

We clarify that “areas” in this context refers to “geographical areas.” This word has been replaced in lines 8 and 18. This clarification has been made in other the text in lines 93,354, 362 and 370.

-line 26: human resources – rephrase to “staff”/ “health care providers”?

“Human resources” in lines 27 has been replaced with “healthcare providers”.

P4
-line 81: Gauteng Province, “of South Africa”

The words “of South Africa” have been added at the end of the sentence in line 82.

-line 85: provide US$ equivalent for international readers

The equivalent for ZAR 992 is ≈ US$64 and this has been provided in line 87.

P5, line 104: perhaps just say that data were collected in July 2020?

Thank you for the correction. The words in “July 2020” have replaced “between July 4 and July 27 2020” in line 105-106

P6, line 138: surely not “back translated” into English as English was not the original language?

Thank you for the observation. The statement in line 139 reads “were translated into English”.

P21, line 442: in addition to being good quality, provided condoms should “desirable” – government issued condoms are often viewed as undesirable as they “smell bad” / too thick etc.

Thank you for this suggestion. We have included in addition to good quality that condoms need to desirable to the end-user in lines 445 and 446.

P22, Line 485: clarify what “this misinformation” refers to
Misinformation has been replaced with the following phrase in line 489 “…misconceptions about existing HIV prevention strategies.”

Re. my previous comment:
- How was gender of participants determined? Was this self-identified gender?
authors responded:
“Gender was self-identified when participants were asked to “tell us more about yourself?” During the interviews, we did not use a demographics questionnaire used to collect this data. We however asked the question “Tell us more about yourself”, in the interview guide and we were able to get the demographic information from the responses provided during discussions". - I am still troubled by this – interviewers categorised participants into genders by their own interpretation of what participant said, or assumption about the person’s gender? Unless ppt clearly stated when asked to “tell about yourself” – that “as a woman” / “as a man”. Methods section should include statement about how gender was categorised, by whom

Re response: “participant self-identified with the label “Deputy-person” which he explained it as being an “MSM” during the interviews.” -Did the participant state that “deputy-person” meant a “gay man” or a man who has sex with other men? Be specific and careful using these categories.

Thank you for raising this comment again. In the methods section lines 160-162, we have included a statement about the categorization of gender as suggested.

*“We categorized gender based on the responses from the question: “Tell us more about yourself,” When the participant explicitly mentioned and voluntarily mentioned their gender as either male or female without probing.*

In this case, the participant who described himself as a “deputy-person” indicated that he was MSM. In line 351, the acronym LGBTQ has been replaced with MSM to reflect this explanation.

Reviewer #2:

1. I would suggest to add the appropriate key words at the end of your abstract, unless the journal requires to put them elsewhere.

Thank you for this comment. We have followed the instructions to authors provided by the journal. The key words are included during the submission process.

2. Page 2, line 30: Interventions including HIV testing services….., I would suggest you add ART initiation for those who test positive as the major response. The comprehensive package includes HIV testing and ART initiation as components that must go together.

Thank you for this suggestion; We have added the words *“…and ART initiation for those who test positive”* on page 2, line 31 and 32.

3. Page 2, line 32: primary healthcare clinics. Introduce its known abbreviation of PHC, if you subsequently use it.

Thank you for this suggestion, primary healthcare clinics in line 33 of page 3 has been abbreviated as (PHCs) and has been subsequently used throughout the manuscript.

4. Page 3, lines 40-50: are these factors documented worldwide? If so, clarify that.

The word “worldwide” has been included to give the extent of the factors affecting uptake of HIV services

5. In the background, it would be great if you start with global facts, then what is known for Africa before introducing South Africa.

Thank you for the comment. We have moved paragraph two (lines 40-51) which explains the global facts to non-utilization of HIV services to paragraph 1and introduced South Africa in paragraph 2.

6. In your study design (Page 4, line 65), introduce that you used in-depth interviews following your declaration of study design. You will then develop that data collection strategy in the recruitment section…

The word in-depth interviews (IDIs) has been introduced following the declaration of the study design (line 75; Page 4)

7. Page 4, line 80. Clearly mentioned whether you used all clinics in the district or you sampled them.

On page 4, line 90, we have clearly mentioned that we sampled three clinics in the district where we conducted the in-depth interviews

8. Page 5, line 107. Use 12 o’clock or 12 PM instead of 12noon. Just for formal writing reasons!

12noon has been replaced with 12pm (Page 5, line 119)

9. If you used thematic analysis, clearly state it. Was this deductively or inductively applied or you moved between both?

On pages 5 and 6, we have clearly stated that we used thematic analysis and developed codes using both inductive and deductive approaches. The following sentence has been added.

*“To analyse the qualitative data, we used thematic analysis and inductively and deductively developed codes”.*

10. I’m happy for the way you used and linked your themes to their relevant SCT domains!

Thank you for this observation

11. Put all your quotes within quotation marks.

All the quotes have been put within quotation marks.

12. Though previously used in data collection section, I would suggest you remind HL, LH and LL as a note under the table/figure for the general public to remember.

Thank you for this observation. A footnote has been added under 3 to define the areas

13. Page 19, line 384, it seems you forgot the detail regarding districts in Ekurhuleni Metropolitan Municipality (EMM) and how you classified them in your study setting (You later wrote about high & low HIV services uptake but this was not linked to the district, unless I missed that detail). This would have clarified this line of discussion.

We had included the definition of a high priority district in South Africa in the Methods section page 4 lines 91 and 92. Ekurhuleni is one such district. In the discussion section, we have added the words “…such as EMM” to confirm that the focus on this district.

14. Page 21, lines 451-452, maybe you missed a word. It was could be health services/care/programs utilization instead of health utilization.

On page 21, lines 472: we have added the word “care”.

15. Page 22, lines 487-488, you stated that a purposive sampling was used but this information was supposed to be brought up in the methods. it’s worth mentioning the reason of this disconnect of design/planning or implementation in few lines or in a single paragraph.

Thank you for pointing out this oversight. I have removed the reference to purposive sampling to prevent this confusion.

**General comment**: Utilisation of HIV services reads much better than HIV intervention. If you prefer to stick to “interventions”, just consider to alternatively use HIV services/care/programmes in the body of your manuscript. If I missed something, others may have picked it and I urge the authors to consider those comments to improve this useful paper before its publication.

Thank you for this comment. We have revised the manuscript and used *“Utilisation of HIV services”* interchangeably with *“HIV services/care/programmes.*
